# Genetic variation in cervical preinvasive and invasive disease: a genome-wide association study

**DOI:** 10.1016/S1470-2045(21)00028-0

**Published:** 2021-04

**Authors:** Sarah J Bowden, Barbara Bodinier, Ilkka Kalliala, Verena Zuber, Dragana Vuckovic, Triada Doulgeraki, Matthew D Whitaker, Matthias Wielscher, Rufus Cartwright, Konstantinos K Tsilidis, Phillip Bennett, Marjo-Riitta Jarvelin, James M Flanagan, Marc Chadeau-Hyam, Maria Kyrgiou, FinnGen consortium

**Affiliations:** a Department of Metabolism, Digestion and Reproduction, Faculty of Medicine, Imperial College London, London, UK; b Department of Surgery and Cancer, Faculty of Medicine, Imperial College London, London, UK; c Department of Epidemiology and Biostatistics, School of Public Health, Faculty of Medicine, Imperial College London, London, UK; d West London Gynaecological Cancer Centre, Hammersmith Hospital, Imperial College Healthcare NHS Trust, London, UK; e Department of Obstetrics and Gynaecology, University of Helsinki and Helsinki University Hospital, Helsinki, Finland; f Department of Urogynaecology, London North West Hospitals NHS Trust, London, UK; g Department of Hygiene and Epidemiology, University of Ioannina School of Medicine, Ioannina, Greece; h Center for Life Course Health Research, Faculty of Medicine, University of Oulu, Oulu, Finland; i Unit of Primary Health Care, Oulu University Hospital, Oulu, Finland; j Department of Life Sciences, College of Health and Life Sciences, Brunel University London, London, UK

## Abstract

**Background:**

Most uterine cervical high-risk human papillomavirus (HPV) infections are transient, with only a small fraction developing into cervical cancer. Family aggregation studies and heritability estimates suggest a significant inherited genetic component. Candidate gene studies and previous genome-wide association studies (GWASs) report associations between the *HLA* region and cervical cancer. Adopting a genome-wide approach, we aimed to compare genetic variation in women with invasive cervical cancer and cervical intraepithelial neoplasia (CIN) grade 3 with that in healthy controls.

**Methods:**

We did a GWAS in a cohort of unrelated European individuals using data from UK Biobank, a population-based cohort including 273 377 women aged 40–69 years at recruitment between March 13, 2006, and Oct 1, 2010. We used an additive univariate logistic regression model to analyse genetic variants associated with invasive cervical cancer or CIN3. We sought replication of candidate associations in FinnGen, a large independent dataset of 128 123 individuals. We also did a two-sample mendelian randomisation approach to explore the role of risk factors in the genetic risk of cervical cancer.

**Findings:**

We included 4769 CIN3 and invasive cervical cancer case samples and 145 545 control samples in the GWAS. Of 9 600 464 assayed and imputed single-nucleotide polymorphisms (SNPs), six independent variants were associated with CIN3 and invasive cervical cancer. These included novel loci rs10175462 (*PAX8*; odds ratio [OR] 0·87, 95% CI 0·84–0·91; p=1·07 × 10^−9^) and rs27069 (*CLPTM1L*; 0·88, 0·84–0·92; p=2·51 × 10^−9^), and previously reported signals at rs9272050 (*HLA-DQA1*; 1·27, 1·21–1·32; p=2·51 × 10^−28^), rs6938453 (*MICA*; 0·79, 0·75–0·83; p=1·97 × 10^−17^), rs55986091 (*HLA-DQB1*; 0·66, 0·60–0·72; p=6·42 × 10^−28^), and rs9266183 (*HLA-B*; 0·73, 0·64–0·83; p=1·53 × 10^−6^). Three SNPs were replicated in the independent Finnish dataset of 1648 invasive cervical cancer cases: *PAX8* (rs10175462; p=0·015), *CLPTM1L* (rs27069; p=2·54 × 10^−7^), and *HLA-DQA1* (rs9272050; p=7·90 × 10^−8^). Mendelian randomisation further supported the complementary role of smoking (OR 2·46, 95% CI 1·64–3·69), older age at first pregnancy (0·80, 0·68–0·95), and number of sexual partners (1·95, 1·44–2·63) in the risk of developing cervical cancer.

**Interpretation:**

Our results provide new evidence for the genetic susceptibility to cervical cancer, specifically the *PAX8, CLPTM1L,* and *HLA* genes, suggesting disruption in apoptotic and immune function pathways. Future studies integrating host and viral, genetic, and epigenetic variation, could further elucidate complex host–viral interactions.

**Funding:**

NIHR Imperial BRC Wellcome 4i Clinician Scientist Training Programme.

## Introduction

Despite the introduction of screening and vaccination programmes, invasive cervical cancer remains one of the most common malignancies in women globally. Persistent infection with oncogenic high-risk human papillomavirus (HPV) subtypes is necessary for the development of cervical cancer and its precursor cervical intraepithelial neoplasia (CIN).^1^ HPV infection is common, with a lifetime incidence over 70%.^2^ Most infections are transient and cleared through an incompletely understood immune response, but a fraction of infected women develop a persistent HPV infection, which ultimately progresses to CIN or invasive cervical cancer.^3^

A number of factors, both host and viral, have been reported to affect HPV clearance and the risk of progression to cervical cancer. HPV genotype, HPV genetic and epigenetic variation,^4^ and viral load have been shown to affect disease outcome. Host behavioural and environmental factors that might influence exposure to HPV or immune response to infection have been reported, including tobacco smoking,^5^ hormonal contraceptives, and socioeconomic, reproductive, and sexual factors.^6^ Family-based studies have reported family aggregation of cervical cancer, but were not able to discriminate between the effect of genes and shared environmental factors.^7^, ^8^ Population-based studies provide evidence for a genetic contribution (27% attributable fraction) to the development of cervical tumours,^9^ and report common variant-based heritability at 36%.^10^

**Research in context**

**Evidence before this study**

The impact of genetic variance on predisposition to cervical cancer has long been debated and there remains uncertainty regarding the hereditary component of cervical malignancy. Family aggregation studies and heritability estimates suggest an inherited genetic contribution to disease risk of up to 27–36%. We searched PubMed and the GWAS Catalog with the terms “cervical cancer” OR “cervical intraepithelial neoplasia” OR “CIN” in combination with “genome-wide association” OR “GWAS”, for articles of any language, published from Jan 1, 1990, to April 16,2020, in which genes previously associated with risk of cervical precancer or cancer were identified. To our knowledge, seven previous genome-wide association studies of cervical preinvasive and invasive disease have been performed worldwide, with the most consistently identified signals in the *HLA* region. However, these studies were limited by modest sample sizes and by the low number of invasive cases included, with case phenotypes mainly including preinvasive disease. Because of the lack of formal replication, results have thus far been inconsistent in the literature and further work is required to characterise the genetic predisposition to cervical cancer.

**Added value of this study**

To our knowledge, this study presents results from the largest genetic association study to date, which explores both cervical preinvasive and invasive phenotypes and includes the largest analysis of invasive cervical cancer phenotypes. We expanded the study of genetic variants outside of the *HLA* region with a broad imputation and identified novel variants at genome-wide significance in the *PAX8* and *CLPTM1L* genes. Results were validated in a large independent replication set. Through mendelian randomisation, we were able to triangulate evidence independently linking cervical cancer risk to earlier age at first pregnancy, higher number of sexual partners, and exposure to tobacco smoking.

**Implications of all the available evidence**

Our results provide new evidence for genetic susceptibility to cervical cancer, suggesting disruption in apoptotic and immune function pathways at the *PAX8, CLPTM1L,* and *HLA* genes, and provide further insight into high-risk behavioural factors. Studies integrating analysis of host susceptibility and viral genetic variation, along with epigenetic behaviour, will further elucidate the differential risk associated with the complex host–viral interaction.

Several candidate gene studies have explored genetic regions related to plausible immunological and carcinogenic pathways (including the *HLA* region^11^), but have shown inconsistent results that frequently could not be replicated in larger cohorts. To our knowledge, seven genome-wide association studies (GWASs) have examined genetic variation in cervical precancer or cancer, including European,^10^, ^12^ Chinese,^13^ and Japanese populations.^14^ These studies varied in size and reported a total of 112 single-nucleotide polymorphisms (SNPs) associated, at genome-wide significance level, with cervical disease. The most consistently identified allelic variation is at the 6p21.3 locus, within the *HLA* region,^10^, ^12^, ^13^ where 44 variants have been reported across six studies, and the MHC class I polypeptide-related sequence A (*MICA*) gene.^10^, ^12^

Our study aimed to perform a genome-wide exploration of variants associated with invasive cervical cancer or high-grade preinvasive lesions (CIN grade 3 [CIN3]) using data from the UK Biobank; and to validate candidate associations in an independent population (FinnGen). Using a two-sample mendelian randomisation approach, we explored the role of the most established risk factors in the genetic risk of cervical cancer.

## Methods

### Study design and participants

We did a GWAS in female UK Biobank participants of European ancestry and with available genotyping data after quality control. The UK Biobank is a population-based cohort of approximately 500 000 volunteers (n=273 377 women with genotyping data available), aged 40–69 years at recruitment between March 13, 2006, and Oct 1, 2010 (appendix p 2).^15^

UK Biobank has approval from the North West multicentre Research Ethics Committee, which covers the UK. In Scotland, UK Biobank has approval from the Community Health Index Advisory Group.

The FinnGen study was used as a replication cohort. The FinnGen cohort (release 5) is a public–private partnership combining gentoyping data from Finnish Biobanks and digital health record data from Finnish health registries. Samples are made up of nine participating biobanks (Auria Biobank, Biobank Borealis of Northern Finland, Biobank of Eastern Finland, Central Finland Biobank, Finnish Clinical Biobank Tampere, Helsinki Biobank, Terveystalo Biobank, and THL Biobank). The cohort consists of a total of 218 792 individuals at the latest data release on July 15, 2020.

The Coordinating Ethics Committee of the Hospital District of Helsinki and Uusimaa (HUS) approved the FinnGen study, protocol number HUS/990/2017.

### Procedures

We used existing genotype calls on DNA samples from female UK Biobank participants who were genotyped for 820 967 variants using a custom Affymetrix UK Biobank Axiom array (Affymetrix Reseacher Services Laboratory, Santa Clara, CA, USA) and 807 411 variants using the BiLEVE Axiom array (Affymetrix Reseacher Services Laboratory) from the UK BiLEVE study. Imputation was done centrally according to HG37 Haplotype Reference Consortium and UK10K and 1000 Genomes project reference panels by UK Biobank, resulting in 9 600 464 imputed common variants for analysis.

Samples that failed genotyping quality control, had high heterozygosity for autosomal chromosomes or missingness (samples identified as outliers in UK Biobank data field 22027), genetic sex discordance, high guanine–cytosine content, missing covariates, and duplicates were excluded from the present analysis. The resultant 235 716 samples were retained for analysis.

To reduce bias due to high degree of relatedness within the UK Biobank, we used a graph representation of the kinship between participants from UK Biobank kinship coefficient data to define a set of unrelated individuals of all ancestries (n=180 224; appendix p 3). To avoid bias from transethnic linkage disequilibrium pattern heterogeneity, data were restricted to the largest ethnic group, which was European ancestry (n=150 314). Ancestry was identified by genetic information, as extracted from UK Biobank field 22 006 (appendix p 3).

The primary disease traits studied consisted of diagnoses of both cervical cancer and carcinoma in situ (CIN3). Cervical cancer case samples were identified as either CIN3 or invasive cervical cancer using diagnoses from a series of predefined International Classification of Diseases codes via linkage to UK cancer registries (followed up to Dec 14, 2016) and hospital episode statistics (followed up to March 31, 2017; appendix pp 10–11). Controls (n=145 545) were identified as women with no record of or reported history of any cervical abnormality from nurse-administered questionnaires at recruitment and linkage to hospital episode statistics.

### Genetic association analysis

The association study was done using an additive univariate logistic regression model analysing cervical cancer status as the outcome against each of the imputed SNPs using PLINK v1.9b3.3. To maximise statistical power, we adopted in our primary analyses a broad definition of cervical cancer including both CIN3 and invasive cervical cancer outcomes. To account for disease heterogeneity and progression, we subsequently analysed case samples from each subtype separately.

Regression models were adjusted for established confounders including age, smoking status (classified as never, former, or current, as obtained from UK Biobank field 20 116), social deprivation score (UK Biobank field 189), and in order to model technically induced nuisance variation, the analytical batch (UK Biobank field 22 000). Effect size estimates were expressed as odds ratio (OR) and 95% CI measuring risk change per copy of the minor allele. To control for population structure and covert relatedness, we adjusted our analyses for the first ten principal components as estimated by UK Biobank and capturing the study population latent genetic structure. We only considered SNPs with a minor allele frequency (MAF) greater than 1% (n=656 284 genotyped; 9 600 464 imputed). To control for multiple testing, we considered the established genome-wide significance level of 5 × 10^−8^

We did a sensitivity analysis only considering genotyped variants (ie, discarding imputed genotypes). We did a series of conditional analyses to identify genetic variants that were independently associated with cervical cancer outcomes and complementarily contributed to the outcome explanation. For each chromosome separately, an iterative procedure was done that identified the SNP with the strongest association and conditioned the logistic regression model on that SNP. This procedure was repeated by sequentially conditioning on the strongest SNP in the chromosome and was continued until there were no remaining SNPs associated with the outcome. Conditional p values (p_cond_ ) are defined for each SNP as the p value for the model adjusted for all other associated SNPs in the chromosome. We evaluated the novelty of our findings based on reported SNPs from the GWAS Catalog (accessed May 20, 2020) and accounted for a 1 Mb window. LocusZoom software (Original LocusZoom version) and visual inspection of heatmaps (generated in linkage disequilibrium based on the 1000 Genomes project) were used to explore linkage disequilibrium blocks of SNPs identified in conditional analyses.

We did an independent validation of the conditional SNPs identified in our discovery dataset using the FinnGen release 5 dataset (replication cohort), in which a total of 655 973 genetic markers were assayed and imputed against a Finnish population-specific backbone (appendix p 4). These data include 4246 case samples from a Finnish population. Replication in the FinnGen release 5 dataset was sought for the SNPs and insertion–deletions (indels) found to be genome-wide significant (p≤5 **×** 10^−8^) with a MAF of 1% or greater following conditional analysis in our discovery set, using a nominal significance level of p=0·05.

### Functional analyses of variants and genes

To explore the phenotypic and functional effects of detected genetic loci, we used publicly available databases (Phenoscanner, UCSC genome browser, dbSNP, and ENCODE) to annotate variants. We searched for previously reported associations with other disease traits in the GWAS Catalog. We subsequently used publicly available datasets to explore potential carcinogenic pathways through the examination of gene expression in cervical cancer tissue, normal cervical tissue, and other tumour tissues (including expression quantitative trait loci [eQTL] ratios in the PanCanQTL database and the GTEx Portal). To establish if any gene already had a known role in cancer, we further searched somatic mutations (COSMIC) in cervical and other cancers. Intronic variants were further investigated for possible indirect effects on gene expression through transcription regulation including histone marks and motifs in HaploReg v4.1 (appendix p 5).

### Mendelian randomisation

To quantify the contribution of established environmental risk factors to the risk of cervical cancer, we did a two-sample mendelian randomisation using independent (squared coefficient of correlation [r^2^]<0·001) genetic variants with known effects on the risk factors, as instruments for the exposure (at genome-wide significance).

We used the largest available datasets to seek genetic instruments of the most established risk factors: tobacco smoking, age at first pregnancy, and number of sexual partners (appendix pp 12, 13). In our two-sample mendelian randomisation, we quantify the linear contribution of these instruments to the genetically predicted risk of cervical cancer, as measured by log odds (log OR), using inverse variance weighting mendelian randomisation to estimate effect size expressed as OR and 95% CI. For exposures with significant effects in the main analysis (using Benjamini and Hochberg's false-discovery rate below 5%), we used a range of robust mendelian randomisation approaches to account for possible violations of the instrumental variable assumptions including weighted-median and mendelian randomisation-presso (appendix p 5). We subsequently did two multivariable mendelian randomisation models (appendix p 5) to account for potential pleiotropy and attenuation when adjusting for age of first pregnancy or risky behaviours (ie, lifetime smoking index and number of sexual partners). For multivariable mendelian randomisation, we used genetic variants associated with either of the two exposures as instrumental variables at genome-wide significance.

### Role of the funding source

The funders had no role in the study design; in the collection, analysis, or interpretation of data; in the writing of the report; or in the decision to submit the paper for publication.

## Results

**Figure 1:**
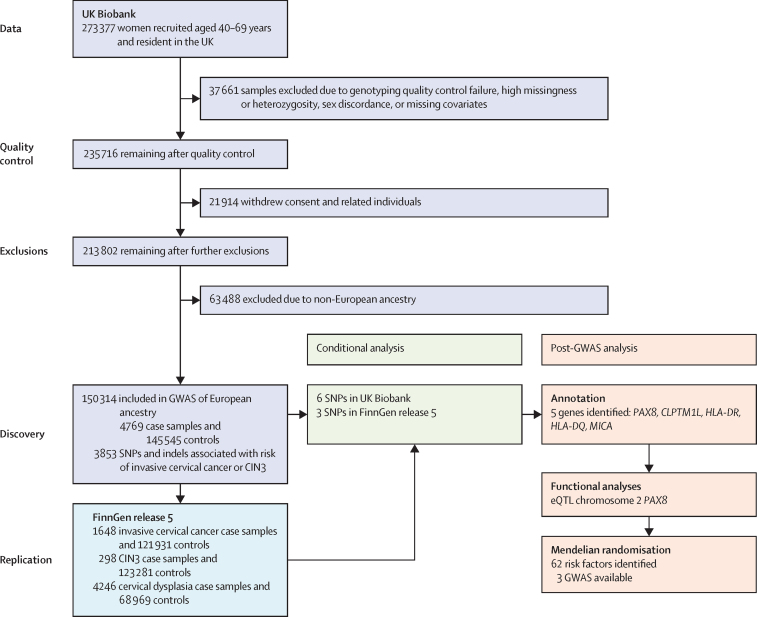
Analysis plans for the discovery (UK Biobank) and replication (FinnGen release 5) datasets GWAS=genome-wide association study. CIN3=cervical intraepithelial neoplasia grade 3. SNP=single-nucleotide polymorphism. eQTL=expression quantitative trait loci.

From the original 235 716 female participants with genotyping and phenotypic data available in UK Biobank (after quality control), 85 402 were excluded, leaving 150 314 women for subsequent analyses (4769 case samples [764 invasive cervical cancer and 4005 CIN3] and 145 545 controls; figure 1; appendix p 8).

The GWAS identified 3853 SNPs and indels associated with the risk of invasive cervical cancer or CIN3. Functional analysis (appendix p 6) revealed that these associations targeted three genetic regions: the *HLA* region in which 3815 of the 3853 detected associations were located (chromosome 6: base pairs 31 324 647–32 623 713), *PAX8* (2q14.1; lead SNP rs10175462 [OR 0·87, 95% CI 0·84–0·91]; p=1·07 **×** 10^−9^), and *CLPTM1L* (5p15.33; lead SNP rs27069 [0·88, 0·84–0·92]; p=2·51 **×** 10^−9^; appendix pp 14 and 118). There was minimal evidence of genomic inflation (appendix p 119).

When restricting the phenotype to invasive cervical cancer case samples only (n=764), signals in the *PAX8* and *HLA* region*s* were attenuated and did not reach the genome-wide significance level, and a novel locus was detected at 12q24.11 (rs117960705; OR 2·41 [95% CI 1·81–3·23]; p=2·76 **×** 10^−9^) located in the *ACACB* gene (appendix p 120). In a comparative random subset of 764 CIN3 cases, previously detected *PAX8* signals were no longer apparent, while *HLA* loci remained associated with CIN3 at a genome-wide significance level (appendix p 120).

In the 9 600 464 assayed and imputed variants, conditional analyses identified six loci independently associated with CIN3 or invasive cervical cancer (table). This included one signal in chromosome 2 (driving SNP rs10175462 and indel 113992800_TCC_T; *PAX8*), one in chromosome 5 (driving SNP rs27069; *CLPTM1L*), and four independent signals in the *HLA* region: three common non-coding variants rs9272050 (*HLA-DQA1;* OR 1·27 [95% CI 1·21–1·32]; p=2·51 **×** 10^−28^), rs6938453 (*MICA*; OR 0·79 [0·75–0·83]; p=1·97 × 10^−17^), and rs55986091 (*HLA-DQB1*; 0·66 [0·60–0·72]; p=6·42 **×** 10^−22^), and one low-frequency missense variant in the *HLA-B* gene rs9266183 (0·73 [0·64–0·83]; p=1·53 × 10^−6^; OR_cond_ 0·68 [0·59–0·77]; p_cond_=6·20 × 10^−9^ MAF 0·04 in Europeans). Conditional analyses identified rs117960705 (chromosome 12) as the only variant independently associated with the invasive cervical cancer outcome, and rs150406145 (chromosome 2) and rs9272245 (chromosome 6, *HLA* region) as independent markers of CIN3.

**Table:** Results from logistic models predicting CIN3 or invasive cervical cancer case-control status from each single-nucleotide polymorphism

	**Chromosome**	**Base pairs**	**Allele 1**	**MAF**	**Info**	**Variant type**	**Nearest gene**	**CIN3 or invasive cervical cancer**			**CIN3**			**Invasive cervical cancer**		
								N	OR (95%CI)	p (p_cond_)	N	OR (95%CI)	p (p_cond_)	N	OR (95%CI)	p (p_cond_)
**CIN3 and invasive cervical cancer**																
rs10175462	2	113 988 492	A	0·362	1·000	Intron	*PAX8*	150 314	0·87 (0·84–0·91)	1·07 × 10^−9^ (1·07 × 10^−9^)	146 313	0·88 (0·79–0·98)	1·55 × 10^−2^ (..)	146 309	0·91 (0·82–1·02)	9·26 × 10^−2^ (..)
rs27069	5	1 347 128	T	0·431	0·986	Intergenic	*CLPTM1L*	146 530	0·88 (0·84–0·92)	2·51 × 10^−9^ (2·51 × 10^−9^)	142 644	0·86 (0·77–0·95)	4·64 × 10^−3^ (..)	142 641	0·86 (0·77–0·95)	3·61 × 10^−3^ (..)
rs9266183	6	31 324 647	C	0·037	1·000	Missense	*HLA-B*	150 314	0·73 (0·64–0·83)	1·53 × 10^−6^ (6·20 × 10^−9^)	146 313	0·75 (0·55–1·03)	7·73 × 10^−2^ (..)	146 309	0·63 (0·45–0·89)	7·88 × 10^−3^ (..)
rs6938453	6	31 377 793	A	0·228	0·982	Intron	*MICA*	146 033	0·79 (0·75–0·83)	1·97 × 10^−17^ (1·35 × 10^−15^)	142 125	0·79 (0·69–0·91)	7·42 × 10^−4^ (..)	142 121	0·76 (0·66–0·87)	7·78 × 10^−5^ (..)
rs9272050	6	32 599 071	G	0·385	0·999	Intron	*HLA-DQA1*	150 211	1·27 (1·21–1·32)	2·51 × 10^−28^ (2·51 × 10^−28^)	146 212	1·42 (1·29–1·58)	9·56 × 10^−12^ (..)	146 209	1·20 (1·08–1·33)	6·69 × 10^−4^ (..)
rs55986091	6	32 623 713	A	0·091	0·995	Intergenic	*HLA-DQB1*	149 757	0·66 (0·60–0·72)	6·42 × 10^−22^ (1·02 × 10^−11^)	145 773	0·68 (0·56–0·84)	3·22 × 10^−4^ (..)	145 771	0·72 (0·59–0·89)	1·84 × 10–3 (..)
**CIN3**																
rs150406145	2	6 371 190	A	0·012	0·929	Intron	*PAX8*	149 360	1·33 (1·12–1·56)	8·54 × 10^−4^ (..)	145 385	2·37 (1·74–3·22)	4·14 × 10^−8^ (4·14 × 10^−8^)	145 380	1·05 (0·66–1·65)	8·51 × 10^−1^ (..)
rs9272245	6	32 602 872	C	0·364	−	Intergenic	*HLA-DQA1*	149 283	1·26 (1·21–1·31)	1·59 × 10^−26^ (..)	145 318	1·44 (1·30–1·60)	4·60 × 10^−12^ (4·60 × 10^−12^)	145 323	1·21 (1·09–1·34)	4·23 × 10^−4^ (..)
**Invasive cervical cancer**																
rs138446575	1	2 811 427	T	0·013	0·953	Intergenic	*TTC34*	149 651	1·24 (1·04–1·48)	1·66 × 10^−2^ (..)	145 674	1·03 (0·64–1·64)	9·12 × 10^−1^ (..)	145 671	2·39 (1·75–3·27)	4·97 × 10^−8^ (4·97 × 10^−8^)
rs117960705	12	109 645 816	G	0·090	1·000	Intron	*ACACB*	150 314	1·22 (1·04–1·44)	1·71 × 10^−2^ (..)	146 313	0·78 (0·47–1·28)	3·21 × 10^−1^ (..)	146 309	2·41 (1·81–3·23)	2·64 × 10^−9^ (2·64 × 10^−9^)

Models are adjusted for age, smoking status, Townsend index, batch, and the first ten principal components. Results are reported for genome-wide association study significant single-nucleotide polymorphisms following conditional analysis (p_cond_<5×10^−8^) for both CIN3 and invasive cervical cancer, CIN3 only, and invasive cervical cancer only. CIN3=cervical intraepithelial neoplasia grade 3. MAF=minor allele frequency. OR=odds ratio. p_cond_=p value after conditional analysis.

**Figure 2:**
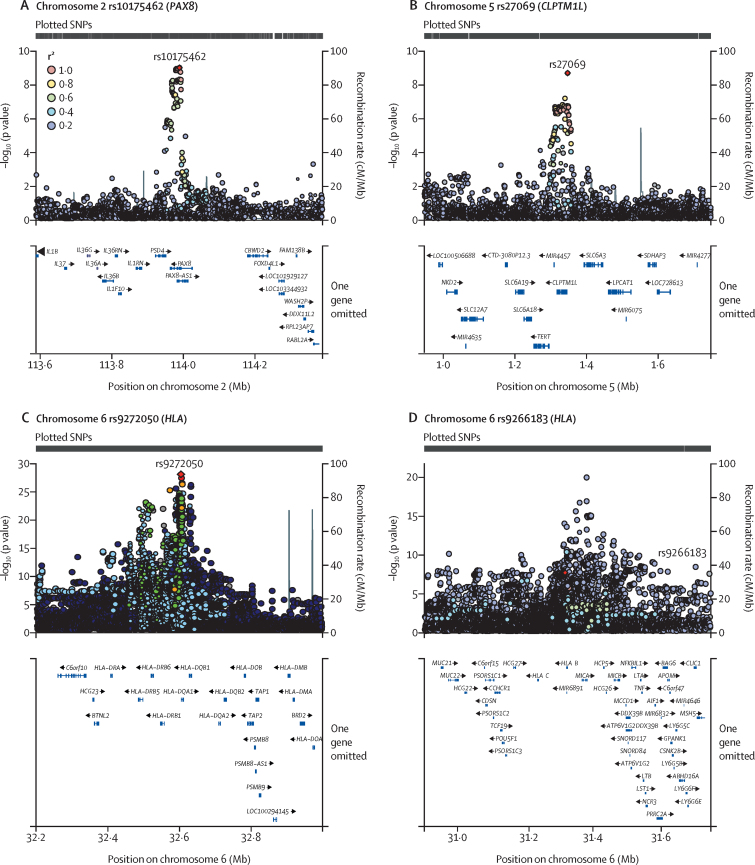
Regional plots of sentinel SNPs from conditional analysis associated with CIN3 and invasive cervical cancer in *PAX8* (chromosome 2; A), *CLPTM1L* (chromosome 5; B), and *HLA* (chromosome 6; C and D) The p value of the association between variant and the cervical cancer status is represented by the log_10_ scale (y-axis) in function of the genetic location. SNPs are colour coded in relation to their correlation (as measured by their recombination rate [r_2_]) with the sentinel SNP (shown in red) in the region. In the lower panel of each plot, gene annotations are provided according to the 1000 Genomes project 2014. SNP=single nucleotide polymorphism. CIN3=cervical intraepithelial neoplasia grade 3.

Regional plots of sentinel SNPs further showed single signals at the *PAX8* and *CLPTM1L* genes (figure 2A, B), whereas associations in the *HLA* were tightly clustered (figure 2C, D). Exploration of the linkage disequilibrium structures of conditional signals showed single blocks in chromosomes 2 and 5, and a complex linkage disequilibrium structure in the *HLA* region (appendix p 121). Comparison of the three remaining conditionally associated SNPs in the *HLA-DQA1, HLA-DQB1*, and *MICA* with GWAS Catalog suggests they had not been previously identified, but were in high linkage disequilibrium with reported variants (r^2^>0·8; appendix p 97). The *HLA-B* low-frequency missense variant rs9266183 was not identified (to our knowledge) by any previous study (appendix p 97).

Analysis restricted to genotyped variants revealed the same pattern of association as the imputed and genotyped variants in *HLA* and *PAX8* regions, but the signal in *CLPTM1L* no longer reached the genome-wide significance level (appendix p 122).

In FinnGen release 5, in the absence of detailed information on disease subtypes, we did three separate GWASs of cervical precancer and cancer phenotypes including a total of 128 123 participants. We investigated separately the cervical dysplasia outcome (n=4246, including mainly CIN3 but also CIN1–2 case samples, appendix p 4), CIN3 outcome (n=298), and invasive cervical cancer outcome (n=1648).

Of our six conditional loci, three were assayed in the FinnGen release 5 dataset (*PAX8* rs10175462 [and indel rs35724515]; *CLPTM1L* rs27069; and *HLA-DQA1* rs9272050). Replication was achieved for the *PAX8* SNP rs10175462 (and indel rs35724515) for invasive cervical cancer (p=0·015) and cervical dysplasia (p=0·0002) phenotypes with concordant direction of effect. No effect of rs10175462 on the CIN3 phenotype was observed (p=0·069; OR 0·86, 95% CI 0·73–1·01; appendix p 112). The significant association of *HLA-DQA1* lead SNP (rs9272050) was replicated in invasive cervical cancer (p=7·90 × 10^−8^), CIN3 (p=0·0005), and cervical dysplasia (p=5·33 × 10^−10^) phenotypes. Lead SNP rs27069 in the *CLPTM1L* gene was also replicated in invasive cervical cancer (p=2·54 × 10^−7^), CIN3 (p=0·04), and cervical dysplasia (p=0·0004) phenotypes.

**Figure 3:**
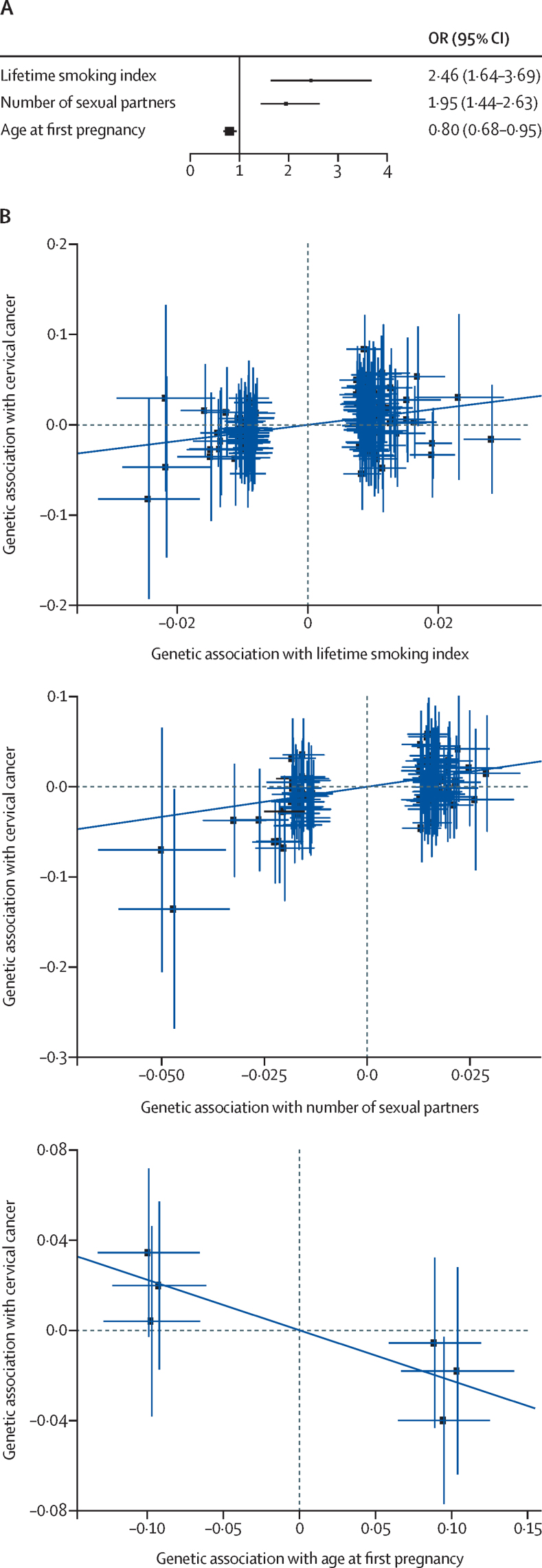
Mendelian randomisation analysis of exposures associated with CIN3 and invasive cervical cancer (A) Forest plot shows inverse variance weighted mendelian randomisation for all identified known environmental risk or protective factors for cervical cancer with available GWAS, to determine effect sizes by OR and 95% CI (x-axis; n=3). (B) Three mendelian randomisation exposures (x-axis, lifetime smoking index; number of sexual partners; age of first pregnancy) found to have a significant association with cervical cancer (y-axis). Each blue point represents a genetic variant used as an instrumental variable in the mendelian randomisation analysis and error bars show standard errors of genetic associations. The dark blue trendline represents the causal effect estimate from the inverse variance weighting mendelian randomisation on the β scale. CIN3=cervical intraepithelial neoplasia grade 3. GWAS=genome-wide association study. OR=odds ratio.

In the mendelian randomisation, the strongest associations with risk of cervical cancer were observed for smoking (OR 2·46, 95% CI 1·64–3·69) and number of sexual partners (1·95, 1·44–2·63; figure 3). We additionally identified a protective effect for older age at first pregnancy (0·80, 0·68–0·95; figure 3; appendix p 114). Sensitivity analyses using weighted-median and mendelian randomisation-Presso confirmed consistent effect sizes with the main analysis based on inverse variance weighting (appendix p 115). In attenuation analyses to account for potential pleiotropic effects, we found that the effect of age at first pregnancy was independent of the number of sexual partners as there was no attenuation for age at first pregnancy when accounting for number of sexual partners (appendix p 116). In the multivariable mendelian randomisation model including both smoking and number of sexual partners, the effect of smoking did attenuate when accounting for the number of sexual partners, but remained significant (OR 1·74, 95% CI 1·08–2·81; appendix p 160).

## Discussion

In this study, we present results from the largest GWAS to our knowledge so far on this subject, analysing cervical cancer phenotypes in more than 150 314 women of European ancestry and over 9 million imputed and genotyped genetic variants. We provide evidence for a strong association at six independent loci in the *PAX8, CLPTM1L*, and *HLA* regions in the UK Biobank. We were able to replicate three SNPs in *PAX8* (rs10175462), *CLPTM1L* (rs27069), and *HLA-DQA1* (rs9272050) in an independent Finnish dataset of 4246 cervical dysplasia cases, 298 CIN3 cases, and 1648 cervical cancer cases (FinnGen).

Previous GWASs have been limited by the modest sample sizes and rarity of invasive cervical cancer; cervical disease phenotypes have been largely dominated by precancerous disease with no separate analyses of cancers. Imputations to permit expansion of studied loci have rarely been done, with the exception of the *HLA* region.^10^, ^13^, ^16^ Furthermore, the gene–environment interaction has not to date been adequately explored in cervical cancer, and our mendelian randomisation supported a risk-increasing effect of smoking, and number of sexual partners, and a protective effect of older age at first pregnancy on cervical cancer.

Our results in the *HLA* region are consistent with previous GWASs in cervical cancer, where allelic variation has been reported for *HLA-*DRB1,^10^, ^12^, ^14^, ^16^ *HLA-*DQA1,^12^ *HLA-*DQB1,^10^, ^16^ *HLA-*DPB1,^13^ *HLA-*DPB2,^13^ and *HLA-*B,^16^ and both protective and risk haplotypes have been described. Associations at the nearby *MICA* gene have also been replicated.^12^ *HLA-DRB, HLA-DQA1*, and *MICA* genes have important roles in the adaptive immune response to infection—coding for MHC class I and II alleles, which are responsible for antigen processing and exogeneous peptide presentation. Such responses are particularly vital in the mediation of T-cell mediated activity and cytotoxicity, which are known to have key roles in immune responses to both HPV infection and tumour cells.^17^ The *HLA* region is notorious for its complex linkage disequilibrium structure. Although we present four independent SNPS that have not been previously reported, linkage disequilibrium patterns from our analysis suggest high linkage with known variants. Our results therefore probably provide a replication of allelic variation for *HLA-DQA1, HLA-DQB1, HLA-B*, and *MICA*. Notably, gene annotations for non-coding variants are not certain. Of interest is our novel report of the association at a rarer SNP rs9266183 (MAF 0·037; *HLA-B*), which represents a missense variant resulting in a codon shift and amino acid change. Alteration of this allele might result in a shift in peptide presentation properties, distorting the T-cell response to HPV infection.^18^ Owing to the lower MAF, this association has probably gone undetected in previous smaller studies. Despite the growing number of *HLA* associations reported for cervical cancer, minimal laboratory validation has been done; given an anticipated coding effect, rs926618 would be one of the most interesting targets for further functional analyses.

To our knowledge, no SNPs outside of the *HLA* region have been validated in European populations, before this study. Although previous candidate gene studies of cervical cancer have explored regions related to immune function, cell cycle control, DNA repair, and other carcinogenic processes and identified genetic associations including *CTLA4, FANCA, OAS3, SULF1, IFNG, DUT, DMC1, GTF2H4* and *EVER1/2*,^19^ *ERAP1, LMP7* and *TAP2, TP53, TERT, IL10*,^20^ and *IL17* and *TNF*,^21^ these findings have not been replicated in larger cohorts. In Chinese populations, GWAS-detected loci in *EXOC1* and *GSDMB* genes are suspected to influence immune response to viral infection.^13^ Whereas a Japanese GWAS reported an association with *ARRDC3*, and further evidence from gene knockdown in HeLa cells that genetic variation might affect HPV entry to cells.^14^

We identified novel variants in *PAX8* and *CLPTM1L* intronic regions in UK Biobank samples, which were replicated in the FinnGen dataset of cervical cancer phenotypes. Our functional analysis showed how these associations might represent carcinogenic or immune function pathways. The strongest association at the *PAX8* locus was an indel (del:rs35724515), which was in perfect linkage disequilibrium with the lead SNP rs10175462. Enrichment for rs10175462 in cervical cancer tissue was colocalised with an eQTL, and the strongest effects were observed at LOC654433, suggesting long-range epigenetic regulation silencing might be controlling expression.^22^ Specifically, decreased expression of the whole region was associated with a protective effect in the presence of the alternative T allele. Variant rs10175462 might be tagging the deletion variant rs35724515, resulting in decreased *PAX8* expression leading to a protective effect due to reduced uncontrolled cell growth and anti-apoptosis associated with *PAX8* overexpression.^23^, ^24^

Genetic variation detected at the *CLPTM1L* locus offers additional insight into potential carcinogenic pathways in HPV-driven cancers. *CLPTM1L* codes for a transmembrane protein and has been linked to cell growth promotion in non-cervical tumours,^25^ and altering cisplatin-mediated apoptosis when overexpressed.^26^ *CLPTM1L*'s previous association with multiple mucosal and HPV-driven cancers suggests a role in regulation of viral transmission across epithelial barriers. Additional investigations of anti-*CLPTM1L* monoclonal antibodies in chemoresistant lung and pancreatic cancers suggest that *CLPTM1L* is a potential novel target for cancer therapeutics in various cancers.^27^

Our main GWAS was based on a broad definition of cervical cancer including both invasive cervical cancer and CIN3 case samples. Conditional analyses of the CIN3 or invasive cervical cancer phenotype identified four associated SNPs that could be viewed as independent genetic markers of cervical cancer that complementarily contribute to the explanation of the disease outcome. Although CIN3 is considered the immediate cancer precursor, it is estimated that 30% of women with CIN3 develop invasive cervical cancer within 10–15 years.^28^ In the subgroup of invasive cervical cancer cases alone, there was a strong attenuation of the associations in *HLA* and *PAX8* regions, which were not found to be genome-wide associated with the outcome. Similar attenuation was observed for the associations linking genetic variants in the *PAX8* gene with the CIN3 outcome, both in our UK Biobank participants and in the replication cohort. Conversely, a strong and broad signal was observed in the chromosome 6 *HLA* region in relation to the CIN3 only outcome. Meanwhile, only one SNP (rs9272245, *HLA*) remained significant after our conditional analyses, hence supporting the existence of a single strong genetic signal associated with CIN3. This is the first study with adequate power to separately explore variation in the invasive cancer phenotype alone. These results provide some evidence of histological heterogeneity, with the *HLA* region being more strongly associated with non-progressive preinvasive lesions, rather than invasive cervical cancer, whereas *PAX8* signals might only be detectable in larger sample sizes as shown in the invasive cervical cancer phenotype of the replication cohort. In our replication cohort, due to the original phenotype coding, the analyses pooling all cases included less severe phenotypes (CIN1 and CIN2). Inclusion of these phenotypes might have increased the heterogeneity of the cases and diluted the estimated effects. Longitudinal study of CIN lesions could reveal valuable information on genetic variance associated with more aggressive phenotypes.

Results from our mendelian randomisation models confirmed previous observations that smoking and an increased number of sexual partners are risk factors for CIN and cervical cancer. Indeed, smoking cessation advice is routinely offered at colposcopy clinics. We did find, however, that when applying a multivariable mendelian randomisation analysis, the effect of smoking attenuated when accounting for the number of sexual partners, suggesting that the strong associations seen with smoking might in part be related to increased lifetime HPV exposure from other risky behaviours. We further confirmed that the effect of young age at first pregnancy was not attenuated when controlling for number of sexual partners, suggesting a possible independent effect of young pregnancy on cancer risk, potentially through mechanisms of early cervical trauma or early hormonal changes. We were unable, however, to confirm that risk associated with younger age of first pregnancy was not a surrogate for young age of HPV exposure and age at first sexual intercourse, or related to higher parity. Previous studies have shown a disappearance in effect after controlling for parity,^29^ although in a large pooled analysis the effect of early pregnancy on cervical cancer remained after adjusting for sexual habits (lifetime sexual partners and age at first sexual intercourse).^6^

HPV status was not available in the UK Biobank due to the invasive nature of sampling healthy volunteers and the absence of HPV-based screening data, which has only recently been introduced in most countries. Furthermore, HPV status at a given timepoint would be an unreliable surrogate of exposure or clearance, and serum antibodies tests have variable accuracy and are not commercially available. To reduce potential for this complex confounding, our control group comprised only women with no previous abnormal screening history—because this population was likely to have been exposed to HPV without developing any cervical lesion. Future adequately powered studies should investigate differential genetic variation in the presence of a known screening history of HPV clearance or persistence. The UK Biobank only had a sufficient number of case samples to study European ancestry. Given the high burden of cervical malignancy in non-European populations, future studies should further explore genetic variation in other ethnicities, particularly Black and south Asian populations. Although this was the first mendelian randomisation, the analysis was based on publicly available summary-level data—we used female sex-specific data on genetic associations where possible, but such data were not available for all exposures considered (smoking and number of sexual partners). For some of the exposures, there was overlap in samples for the two-sample mendelian randomisation design. However, since we based our analysis on genetic variants that are strongly associated with the exposure, representing strong instrumental variables, the induced bias can be considered negligible.

Our findings offer exciting insights into the genetic predisposition of the cervical cancer trait. This study supports a complex neoplastic model related to both altered immune response and carcinogenic processes in the presence of high-risk HPV. Investigations that integrate the study of host susceptibility with viral genetic variation,^30^ along with epigenetic behaviour, could further elucidate the differential risk related to complex host–viral interactions. Meanwhile, further investigation of the functional effects of loci, within and outside of the *HLA*, is needed to better understand the pathogenesis of cervical neoplasia, and implications for potential therapeutic targets in women with cervical preinvasive and cancerous lesions.
