# Supplementary material for: Genetic variation in cervical preinvasive and invasive disease: a genome-wide association study
Source: Lancet Oncol. 2021 Apr;22(4):548–57. doi: 10.1016/S1470-2045(21)00028-0 (PMC8008734; doi:10.1016/S1470-2045(21)00028-0)
Supplement: Supplementary appendix [file mmc1.pdf]

THE LANCET
Oncology

Supplementary appendix

This appendix formed part of the original submission and has been peer reviewed. We post it as supplied by the authors.

Supplement to: Bowden SJ, Bodinier B, Kalliala I, et al. Genetic variation in cervical preinvasive and invasive disease: a genome-wide association study. *Lancet Oncol* 2021; **22:** 560–69.

CONTENTS

1 SUPPLEMENTARY METHODS

1.1 UK Biobank data

1.2 FinnGen data

1.3 Mendelian randomisation

1.4 Bioinformatic packages and databases

2 FUNCTIONAL ANALYSIS

3 SUPPLEMENTARY TABLES

4 SUPPLEMENTARY FIGURES

5 FINNGEN CONSORTIUM AUTHOR LIST

6 REFERENCES

1 SUPPLEMENTARY METHODS

1.1 UK Biobank (UKB) data

An application (ID: 21146) was made to the UK Biobank on April 15th 2016, approved on September 22nd 2016; full genetic and phenotypic data were downloaded to the Imperial College high performance computer (HPC) network July 1st 2018.

The UK Biobank cohort is a prospective longitudinal cohort of ~500,000 volunteers aged 40-69 at enrolment ascertained in a clustering approach through NHS registers and recruited from 13/03/2006-1/10/2010[1]. Participants who consented to enrolment in the study were asked to attend for a baseline interview with a registered nurse and required to complete a standardised questionnaire with characteristics, life course exposures, medical history, treatments and family history. They consented to lifelong linkage to health data including cancer registries and Hospital Episode Statistics and cause of death data. Patients also underwent standardised anthropometric physical measurements such as BMI and blood pressure at enrolment and donated urine and serum plasma.

Serum plasma was genotyped using specifically designed UK Biobank and UK BiLEVE genotyping arrays, which share 95% variant content[2]. Imputation was undertaken centrally by the UKB in a merging of three reference panels, namely the UK10K, 1000 genomes Phase 3 panel and the Haplotype reference consortium (HRC)[3]. This resulted in ~9million SNPS with a minor allele frequency (MAF) >1% available for GWAS. Unexpected duplicates were later removed in R v4.02.

Definition of the Phenotype

Cases and controls were identified from the UKB release for our project, which was coded in a series of fields as listed in supplement table 1. To identify cases, we used ICD9 and 10 codes (supplement table 2) for cervical cancer and carcinoma in situ, and searched UKB fields linking to UK cancer registries and hospital episode statistics (HES). To define controls, we identified women who were not cases but also with no history of any cervical abnormality, including mild or moderate dysplasia according to participant UKB questionnaires, UKB interviews, and HES.

Quality control

Several Quality Control (QC) steps were undertaken centrally by UKB prior to data release including examination for deviation from Hardy-Weinberg Equilibrium (HWE), batch and plate effects, sex effects, array effects and sex chromosome discordance across control replicates. These SNPS were set to missing for the corresponding batch in the genetic data files provided by UKB. Samples were tested for heterozygosity and missing rates resulting in genotyping data for 488,377 individuals after QC. We removed all individuals with high missingness, sex discordance or high heterozygosity for autosomal

chromosomes. We excluded women that withdrew consent. Further information on the full QC performed by the UKB are available[3,4].

Analysis Model

We restricted the main analysis to European ancestry, based on genetic ancestry provided by UK Biobank (field 22006 available from the UK Biobank showcase at https://www.ukbiobank.ac.uk/data-showcase). A high degree of relatedness has been noted in the UK Biobank population and therefore to reduce confounding we excluded related individuals. We used a graph representation of the kinship between participants to define a set of unrelated individuals, as described elsewhere[3]. In this graph, women participants retained for analysis are the vertices and there is an edge between them if they are 3rd degree relatives or closer. Families correspond to connected components, i.e. sets of nodes that are inter-connected but isolated from the rest of the graph. For each family, we used the algorithm implemented in the igraph package in R to identify the largest set of unrelated individuals[5], i.e. vertices that are not connected to any other vertex. These sets are combined to form the largest possible set of unrelated participants in the population under study. To adjust for covert relatedness we adjusted for ten principle components.

1.2 FinnGen release 5 (r5) data

Replication was performed in the FinnGen r5, through application to the FinnGen team. Patients and control subjects in FinnGen provided informed consent for biobank research, based on the Finnish Biobank Act. Alternatively, older research cohorts, collected prior the start of FinnGen (in August 2017), were collected based on study-specific consents and later transferred to the Finnish biobanks after approval by Fimea, the National Supervisory Authority for Welfare and Health. Recruitment protocols followed the biobank protocols approved by Fimea. The Coordinating Ethics Committee of the Hospital District of Helsinki and Uusimaa (HUS) approved the FinnGen study protocol Nr HUS/990/2017.

The Biobank Access Decisions for FinnGen samples and data utilized in FinnGen Data Freeze 5 include: THL Biobank BB2017_55, BB2017_111, BB2018_19, BB_2018_34, BB_2018_67, BB2018_71, BB2019_7, BB2019_8, BB2019_26, Finnish Red Cross Blood Service Biobank 7.12.2017, Helsinki Biobank HUS/359/2017, Auria Biobank AB17-5154, Biobank Borealis of Northern Finland_2017_1013, Biobank of Eastern Finland 1186/2018, Finnish Clinical Biobank Tampere MH0004, Central Finland Biobank 1-2017, and Terveystalo Biobank STB 2018001.

Genotyping was performed for ~600,000 samples on the FinnGen ThermoFisher Axiom custom array through the ThermoFisher genotyping service in San Diego. The array consists of 736,145 probes for 655,973 markers. In addition to the core GWAS markers it contains 116,402 coding markers enriched in Finland, specific markers for the HLA region, ClinVar markers, pharmacogenomic markers and

selected markers that were of special interest for partners. Legacy samples from other biobanks have previously been genotyped over the years using various generations of Illumina GWAS arrays. All GWAS data is imputed against a Finnish population specific whole genome sequence (WGS) backbone. More details can be found at: https://www.finngen.fi/en/researchers/genotyping. Cervical cancer phenotypes cases were identified from Finnish Hospital Discharge register, Finnish Cancer Registry, and Cause of Death registry according to ICD10, ICD9 and ICD8 codes: “cervical dysplasia”, including cases of CIN 1-3 (ICD10: N87.0-9; ICD8: 621), “cervical carcinoma in situ”, including both endo- and ectocervical carcinoma in situ / CIN3 cases (ICD-10: D06), and malignant neoplasm of the uterine cervix (ICD10: C53; ICD9 & ICD8: 180). CIN3 cases are in Finland invariably coded either as D06 or N87.2, but most of CIN3 cases can be assumed to be included in the “cervical dysplasia” phenotype (https://risteys.finngen.fi/phenocode/N14_DYSPLACERVUT#endpoint-definition). All other women were included as controls. Covariates such as smoking and social deprivation score were not available.

**FinnGen Phenotype**	**Cases/Controls**
Malignant neoplasm of cervix uteri (ICC)	1648/121931
Carcinoma in situ of cervix uteri (CIN3)	298/123281
Dysplasia of cervix uteri (cervical dysplasia CIN1-3)*	4246/68969

* The cervical dysplasia phenotype in FinnGen is based on data from both the Cause of Death and Hospital Discharge Registry (HDR) data. HDR records includes information on inpatient episodes since 1967, day surgical procedures in all hospitals since 1994 and all hospital outpatient visits in public hospitals since 1998. HDR data can therefore include CIN1 cases as well as histological high-grade squamous intraepithelial lesions (HSIL) cases. However, since the majority of treatments are for HSIL (CIN2 or more), the phenotype here will consist mainly of high-grade disease (estimated to be greater than two-thirds of cases). The number of women contributed from the death registry can be considered minimal. In summary, the FinnGen “cervical dysplasia”, phenotype mainly consists of CIN3 (dysplasia gravis, carcinoma in situ), but does include some CIN1-2.

1.3 Mendelian randomisation

Systematic review: We performed a systematic review of the literature and identified the following search strategy to identify publications on risk and protective factors associated with cervical cancer phenotypes, the full details are published at PROSPERO [ID:189995]. The search strategy used to identify meta-analyses and pooled studies was as follows:

1. Uterine Cervical Neoplasms [MeSH Terms] OR Uterine Cervical Dysplasia [MeSH Terms] OR female genital tract cancer OR uterine cervix cancer OR Cervical Intraepithelial Neoplasia [MeSH Terms] OR CIN [tw] OR CINII* [tw] OR CIN2* [tw] OR CINIII* [tw] OR CIN3* [tw] OR SIL [tw] OR HSIL [tw] OR H-SIL [tw] OR LSIL [tw] OR L-SIL [tw] OR ASCUS [tw] OR AS-CUS [tw]) OR (papillomaviridae [MeSH:NoExp] OR alphapapillomavirus [MeSH Terms] OR "DNA, viral" [MeSH Terms] OR Papillomavirus Infections [MeSH Terms] OR Tumor Virus Infections [MeSH Terms] OR "Cervix Uteri/virology" [MeSH Terms] OR HPV [tw] OR "human papillomavirus" [tw] OR papillomaviridae [tw]

2. (cervix[Tw] OR cervical[Tw] OR cervico*[Tw] OR Cerv*[tw]) AND (cancer*[Tw] OR carcinoma[Tw] OR adenocarcinoma[Tw] OR neoplas*[Tw] OR dysplas*[tw] OR dyskaryos*[Tw] OR squamous[Tw])

3. (female genital tract cancer[tw])

4. 2 OR 3

5. 1 OR 4

6. Meta-analysis[pt] OR systematic[sb] OR (Meta-analysis[tw] OR systematic review[tw] OR meta-analysis as topic[mh] OR meta-analysis[mh] OR meta analy*[tw] OR metanaly*[tw] OR metaanaly*[tw] OR met analy*[tw] OR systematic review*[tiab]

7. 5 AND 6

8. animal[mh] NOT human[mh]

9. 7 NOT 8

Analysis Model: For the main analysis we used a two-sample mendelian randomisation approach, we quantify the linear contribution of these instruments to the genetically-predicted risk of cervical cancer, as measured by log-OR, using inverse variance weighting to estimate effect size expressed as OR and 95%CI. For exposures with significant effects in the main IVW MR analysis (multiple testing adjusted for using Benjamini and Hochberg's correction controlling the false discovery rate below 5%), we performed sensitivity analyses using a range of robust MR approaches to circumvent possible violations of the instrumental variable assumptions (weighted median[6] and MR Presso[7]). We subsequently performed two multivariable MR[8] models to account for potential pleiotropy and attenuation. First, we investigated whether age of first pregnancy effect attenuates when accounting for number of sexual partners, using the 10 genetic variants associated with age at first pregnancy[9]. Secondly, we performed a multivariable MR analysis for risky behaviours, where we include the lifetime smoking index[10] and number of sexual partners[11].

1.3 Bioinformatic packages and databases

PLINK v1.9b3.3[12].

GWAS Catalog [accessed 2020-05-20][13]

Phenoscanner[14]

UCSC genome browser[15]

dbSNP[16]

ENCODE[17]

PanCanQTL database[18]

GTEx Portal[19]

COSMIC[20]

HaploReg v4.1[21]

2 Functional Annotation

The strongest association on chromosome 2 was an INDEL (rs35724515), which was tagged to rs10175462 (LD r^2=1, distance 4.3 kb). When investigating the eQTL we detected that the whole region showed allele-specific long-range epigenetic silencing in cervical cancer tissues (Supplementary Figure 4). Meanwhile examination of gene expression in healthy tissues suggested *PAX8* has a limited expression across tissue types and is mostly limited to cervix, thyroid and kidney tissues. For this locus, histone marks were also noted in HeLa (cervical cancer) cell lines, suggesting the SNP may have regulatory effects. Interrogation of the GWAS catalog revealed this SNP has been previously associated with a phenotype of reduced lymphocyte counts[22]. This SNP has not previously been associated with cervical or any other cancers, however increased *PAX8* gene expression has been associated with the mucinous ovarian cancer trait[23] and in epithelial ovarian cancer tumour tissue[24]. Overall there appears to be evidence of a regulatory function of this locus in predisposition to altered immune or carcinogenic pathways.

Annotation of rs27069 revealed an intergenic variant located 2029bp downstream from the *CLPTM1L* (also known as cisplastin-resistance gene), commonly co-expressed with the TERT gene. This SNP had a reported co-localised eQTL locus in oesophageal mucosal tissue and skin in GTEx however, no data were available for cervical tissue. HaploReg reported this SNP as motif-altering for YY1, a transcription factor which has been reported to be correlated with expression of the HPV16 oncogene E6 and progression from CIN to cancer[25]. Previous SNP associations have been reported for this gene and HPV-driven plantar warts[26], as well as oral cavity[27] and nasopharnageal[28] cancers – a significant proportion of which are HPV-driven. Sequence variation at the *TERT-CLPTM1L* locus has been previously associated with cervical cancer as well as other squamous and adeno-carcinoma types including skin and lung cancers[29].

We detected *HLA* associations including protective and risk variants in proximity of *HLA-DRB1*, *HLA-DQA1* and *MICA* genes. The protective association observed at rs9266183 in the *HLA-B* region (6p21.3) has not previously been associated to cervical cancer, and according to the dbSNP reference report, is a predicted missense variant located in the *MHC Class I* gene; this causes an amino acid change from Aspartate to Glycine. Upstream (3'UTR) from the variant is a co-localised microRNA (MIR6891), which has been reported to affect local RNA stability including transcripts for the IgA heavy chain[30]. Cervical mucosal IgA responses to HPV are recognised to be important in preventing infection, re-infection, spread to adjacent cells and HPV clearance[31].

3 SUPPLEMENTARY TABLES

Supplement Table 1. Individual characteristics of the 150,314 unrelated UK Biobank participants of European ancestry in main analysis

	Controls (N=145,545) n	Median (IQR)/ Proportion	CIN3 cases (N=4,005) n	Median (IQR)/ Proportion	ICC cases (N=764) n	Median (IQR) / Proportion
Age at recruitment	145,545	57 (13)	4,005	53 (12)	764	57 (11)
Smoking status						
Never	88,213	60.61	1,729	43.17	348	45.55
Former	45,214	31.07	1,505	37.58	287	37.57
Current	12,118	8.33	771	19.25	129	16.88
Townsend score+	145,545	-2.37 (-3.75/-0.04)	4,005	-1.65 (-3.37/1.20)	764	-1.86 (-3.47/1.00)

Supplement Table 2. Individual characteristics of the 180,224 unrelated UK Biobank (UKB) participants of all ethnicities, as identified by UKB genotyping data

		**Controls** (*N*=174,717) n	Mean (s.d.) / Proportion	**CIN3 cases** (*N*=4,620) n	Mean (s.d.) / Proportion	**Cancer cases** (*N*=887) n	Mean (s.d.) / Proportion
Age at recruitment		174,717	55.83 (8.00)	4,620	53.05 (7.69)	887	56.16 (7.26)
Ethnic background							
	European*	**145,545**	**83.30%**	**4,005**	**86.69%**	**764**	**86.13%**
	White (Other)	19,987	11.44%	487	10.54%	99	11.16%
	Asian	3,517	2.01%	22	0.48%	11	1.24%
	Black	3,516	2.01%	51	1.10%	6	0.68%
	Mixed	1,353	0.77%	40	0.87%	5	0.56%
	Chinese	799	0.46%	15	0.32%	2	0.23%
Smoking status							
	Never	106,244	60.81%	1,981	42.88%	411	46.34%
	Former	53,492	30.62%	1,746	37.79%	330	37.20%
	Current	14,981	8.57%	893	19.33%	146	16.46%
Townsend score**		174,717	-1.38 (3.02)	4,620	-0.79 (3.17)	887	-0.91 (3.24)

*Main analysis restricted to European ancestry as largest ethnic group
**Townsend score is a relative deprivation index used to measure material deprivation based on four variables: unemployment, non-car ownership, non-home ownership, household overcrowding, where increasing score implies increased deprivation.

Abbreviations. n: number of participants with covariate trait, N: Total number of participants in disease phenotype; s.d: standard deviation.

Supplement Table 3. UKB fields requested and accessed to identify cases and controls and genetic quality control

Full_name	Pheno
X1239	Current tobacco smoking
X20001	Cancer code, self-reported
X20006	Interpolated Year when cancer first diagnosed
X20007	Interpolated age of participant when cancer first diagnosed
X20008	Interpolated year when non-cacner illness first diagnosed
X20009	Interpolated Age of participant when non-cancer illness first diagnosed
X20010	Interpolated year when operation took place
X20011	Interpolated age of participant when operation took place
X20012	Method of recording time when cancer first diagnosed
X20013	Method of recording time when non-cancer illness first diagnosed
X20014.0.1	Method of recording time when operation occurred
X20160	Ever smoked
X21000	Ethnic background
X21003	Age when attendde assessment centre
X21022	Age at recruitment
X22001	Genetic Sex
X22002	CEL
X22003	Heterozygosity
X22004	Heterozygosity, PCA corrected
X22005	Missingness
X22006	Genetic ethnic grouping
X22007	Genotype measurement plate
X22008	Genotype measurement well
X22009.0.1	Genetic prinicpal components
X22011	Genetic relatedness pairing
X22012	Genetic relatenedness factor
X22013	Genetic relatedness IBS0
X22018	Genetic relatedness exclusions
X22021	Genetic kinship to other participants
X22100	Chromosome XY genotype results
X22101	Chromosome 1 genotype results
X22102	Chromosome 2 genotype results
X22103	Chromosome 3 genotype results
X22104	Chromosome 4 genotype results
X22105	Chromosome 5 genotype results
X22106	Chromosome 6 genotype results
X22107	Chromosome 7 genotype results
X22108	Chromosome 8 genotype results
X22109	Chromosome 9 genotype results
X22110	Chromosome 10 genotype results
X22111	Chromosome 11 genotype results
X22112	Chromosome 12 genotype results
X22113	Chromosome 13 genotype results
X22114	Chromosome 14 genotype results
X22115	Chromosome 15 genotype results
X22116	Chromosome 16 genotype results
X22117	Chromosome 17 gentoype results
X22118	Chromosome 18 genotype results
X22119	Chromosome 19 genotype results
X22120	Chromosome 20 genotype results
X22121	Chromosome 21 genotype results
X22122	Chromosome 22 genotype results
X22123	Chromosome X gentoype results
X22124	Chromosome Y genotype results
X22125	Mitochondrial genotype results
X22182	HLA imputation values
X22801	Chromosome 1 imputation and haplotype results
X22802	Chromosome 2 imputation and haplotype results
X22803	Chromosome 3 imputation and haplotype results
X22804	Chromosome 4 imputation and haplotype results
X22805	Chromosome 5 imputation and haplotype results
X22806	Chromosome 6 imputation and haplotype results
X22807	Chromosome 7 imputation and haplotype results
X22808	Chromosome 8 imputation and haplotype results
X22809	Chromosome 9 imputation and haplotype results
X22810	Chromosome 10 imputation and haplotype results
X22811	Chromosome 11 imputation and haplotype results
X22812	Chromosome 12 imputation and haplotype results
X22813	Chromosome 13 imputation and haplotype results
X22814	Chromosome 14 imputation and haplotype results
X22815	Chromosome 15 imputation and haplotype results
X22816	Chromosome 16 imputation and haplotype results
X22817	Chromosome 17 imputation and haplotype results
X22818	Chromosome 18 imputation and haplotype results
X22819	Chromosome 19 imputation and haplotype results
X22820	Chromosome 20 imputation and haplotype results
X22821	Chromosome 21 imputation and haplotype results
X22822	Chromosome 22 imputation and haplotype results
X2453	Cancer diagnosed by doctor
X40000	Date of death
X40002	Contributiory (secondary) causes of death
X40005	Date of cancer diagnosis
X40007	Age at death
X40008	Age at cancer diagnosis
X40011	Histology of cancer tumour
X40012	Behaviour of cancer tumour
X40018	Death report format
X41078	Episodes containg "Diagnoses - secondary ICD10" data
X41080	Episodes containing "Operative procedures - secondary OPCS" data
X41082	Episodes containing "Dates of operations" data
X41083	Episodes containing "episode start date" data
X41084	Episodes containing "Episode end date" data
X41096	Episodes containing "Date of admission to hopsital" data
X41101	Episodes containing "Date of discharge from hospital" data
X41142	Episodes containing "Diagnoses - main ICD10" data
X41146	Episodes containing "operative procedure - main OPCS" data
X41148	Episodes containing "Date of operation" data
X41202	Diagnoses - main ICD10
X41203	Diagnoses - main ICD9
X41204	Diagnoses - secondary ICD10
X41205	Diagnoses - secondary ICD9
X52	Month of birth
X53	Date attendance assessment centre
X84	Cancer year/age first occurred
X22200	Year of birth
X54	UK biobank assessment centre
X87	Non-cancer illness year/age first occurred
X92	Operation year/age first occurred
X189	Townsend deprivation index at recruitment
X1249	Past tobacco smoking
X20002	Non-cancer illness code, self reported
X20004	Operation code
X20116	Smoking status
X22506	Tobacco smoking
X40001	Underlying (primary) cause of death
X40006	Type of cancer: ICD10
X40013	Type or cancer: ICD9
X41273	Operative procedures - main OPCS
X41256	Operative procedures - secondary OPCS

Supplement Table 4. ICD9 and 10 codes from UK Biobank field IDs 40006 (ICD10), 40013 (ICD9) and linkage to the Hospital Episode Statistics used to identify cases annd exclude controls

**Inclusion criteria for cases**			
**ICD9**		**ICD10**	
180.0	Malignant neoplasm of endocervix	C53	
180.1	Malignant neoplasm of exocervix	C53.1	
180.8	Malignant neoplasm of other specified sites of cervix	C53.9	
180.9	Malignant neoplasm of cervix uteri, unspecified site	D06.0	CIS endocervix
233.1	Carcinoma in situ (CIN3)	D06.1	CIS ectocervix
		D06.7	CIS other parts of cervix
		D06.9	CIS cervix, unspecidfied
**Exclusion criteria for controls (plus not a case)**			
**ICD10**			
N87.0	Mild dysplasia		
N87.1	Moderate dysplasia		
N87.2	Severe dysplasia		

Abbreviations:ICD9-10: International classification of diseases versions 9 and 10

Supplement Table 5. Exposures identified for cervical cancer and submitted to search for GWAS in preparation for Mendelian randomisation analysis November 2020

Exposure	PMID
1. Combined oral contraceptive pill	12686037;28759170;17993361
2. Injectable contraceptives	12686037
3. Intruterine device	21917519;29112647
4. Active smoking	12854514;14682438
5. Passive smoking	22938442,27321059;16206285;24935346
6. Household air pollution	25890249
7. Chlamydia trachomatis infection	27043670;31175391
8. Herpes simplex virus type 2	25030659
9. EBV infection	29021084
10. HIV and antiretroviral treatment	29107561;29369827
11. HIV infection	17617273;29369827;29873885;30602038
12. HIV, CD4 count	29369827;29873885
13. U.urealyticum infection	29520664
14. U.parvum ingection	29520664
15. M.hominis infection	29520664
16. M.genitalium infection	29520664
17. Coinfection of HPV and C.trachomatis	27043670
18. Vaginal dysbiosis	30550767
19. Bacterial vaginosis	21223574
20. Vaginal microbiota	29729331;31242505
21. Trichomonas vaginalis infection	9240115
22. Vaginal douching	9240115
23. Human papillomavirus vaccine	29740819
24. Age at full term pregnancy	16570271
25. Parity	16570271
26. In vitro fertilisation treatment	23211423
27. Number of sexual partners	25987056
28. Pregnancy	24667102
29. Condom use	12438912
30. Vit B12	15912536
31. Vit C	26731169;15912536
32. Vit E	28829815;15912536
33. Vit A	22005522
34. Folate levels	15912536;21749626
35. Homocysteine levels	15912536
36. Beta-carotene	15912536
37. Alpha carotene	15912536
38. Exposure to trichloroethylene	22005522
39. Lycopene	15912536
40. Lutein	15912536
41. Cryptoxanthin	15912536
42. Retinol	15912536
43. Alpha tocopherol	15912536
44. Intake of fruits	15912536
45. Intake of vegetables (carrots, pumkin, papaya, spinach, broccoli, oranges)	15912536
46. Intake of juices	15912536
47. Carotenoids intake	22005522
48. Zinc levels	30370809
49. Copper levels	30370809
50. Systemic lupus erythematosus	24657969
51. Rheumatoid arthritis	26271620
52. Transplant recipient	17617273
53. Inflammatory bowel disease on immunosuppressive medication	25895005
54. Multiple sclerosis	25533302
55. Eating disorders	33025526
56. T2DM	32349989
57. Maternal reproductive factors	32226046
58. Fruits and vegetables	32156167
59. GDM	32097717
60. Serum iron	31852398
61. Candida albicans	31673281
62. BMI	WCRF CUP database

Supplement Table 6. GWAS studies identified as exposures available for MR analyses

Exposure	N total
Cigarette smoking including duration and heaviness and cessation	462690 individuals
Reproductive behaviour in women as measured by age at first birth and number of children	189656 women
Number of sexual partners as part of a study on risky behaviour measured in individuals	414343 individuals

Supplement Table 7. All SNPs reaching P<5e-8 in the UKB discovery cohort for CIN3 or ICC phenotypes

CHR	RSID	BP	A1	TEST	NMISS	OR	STAT	P
2	rs28679654	113967617	T	ADD	145745	0.8622	-5.769	7.97E-09
2	rs112692022	113970182	T	ADD	147202	0.8623	-5.794	6.88E-09
2	rs7578633	113978650	T	ADD	148698	0.874	-6.02	1.74E-09
2	rs11123170	113978940	G	ADD	148783	0.8735	-6.046	1.48E-09
2	rs12472361	113980645	G	ADD	148533	0.8631	-5.765	8.15E-09
2	rs6707386	113981022	A	ADD	148719	0.8739	-6.025	1.69E-09
2	rs6755040	113981137	C	ADD	148718	0.8732	-6.062	1.35E-09
2	rs4849177	113982584	C	ADD	148716	0.8733	-6.061	1.36E-09
2	rs4849178	113982608	A	ADD	148644	0.8624	-5.797	6.74E-09
2	rs11123172	113984303	C	ADD	148724	0.8734	-6.057	1.39E-09
2	rs4849179	113985170	T	ADD	148714	0.8734	-6.054	1.41E-09
2	rs4849180	113985186	G	ADD	148657	0.8623	-5.802	6.54E-09
2	rs35222158	113986629	T	ADD	148665	0.8626	-5.788	7.13E-09
2	rs10175462	113988492	A	ADD	148783	0.8724	-6.099	1.07E-09
2	rs5833488	113989122	AG	ADD	148709	0.8727	-6.088	1.15E-09
2	rs2863242	113989236	T	ADD	148783	0.8619	-5.822	5.82E-09
2	rs2863243	113989267	C	ADD	148749	0.8729	-6.082	1.19E-09
2	rs7563094	113989327	C	ADD	148750	0.8729	-6.081	1.19E-09
2	rs2166421	113990242	C	ADD	148462	0.8628	-5.774	7.76E-09
2	rs7421852	113990261	A	ADD	148555	0.8746	-5.992	2.08E-09
2	rs10206269	113990393	A	ADD	148581	0.8745	-5.993	2.06E-09
2	rs4849181	113991970	G	ADD	148783	0.8747	-5.984	2.18E-09
2	rs4849182	113992201	A	ADD	148415	0.8632	-5.755	8.67E-09
2	2:113992800_TCC_T	113992800	T	ADD	148130	0.8715	-6.13	8.76E-10
2	rs7576384	113993385	G	ADD	145311	0.8767	-5.814	6.08E-09
5	rs27069	1347128	T	ADD	145041	0.8781	-5.961	2.51E-09
6	rs114115115	30989692	G	ADD	147978	0.7514	-5.741	9.42E-09
6	rs116071718	30990081	A	ADD	147972	0.7573	-5.747	9.06E-09
6	rs3094672	30993377	T	ADD	148783	1.161	6.442	1.18E-10
6	rs3130519	31195097	T	ADD	148669	1.155	6.39	1.65E-10
6	rs3130947	31195365	G	ADD	148766	1.151	6.192	5.95E-10
6	rs9264023	31195796	A	ADD	148761	1.151	6.196	5.78E-10
6	rs3130469	31196336	T	ADD	148773	1.151	6.191	5.96E-10
6	rs3130472	31198205	G	ADD	148778	1.15	6.189	6.06E-10
6	rs3130939	31198507	A	ADD	148779	1.151	6.189	6.05E-10

6	rs3130527	31198987	T	ADD	148783	1.15	6.176	6.59E-10
6	rs3132532	31201202	A	ADD	148708	1.145	5.953	2.64E-09
6	rs2394888	31202246	A	ADD	148708	1.145	5.953	2.64E-09
6	rs2394889	31202305	C	ADD	148706	1.145	5.954	2.61E-09
6	rs3130701	31202686	C	ADD	148709	1.145	5.952	2.64E-09
6	rs3134770	31204986	T	ADD	148751	1.146	5.974	2.32E-09
6	rs9265189	31289588	A	ADD	148198	1.141	5.941	2.84E-09
6	rs9265258	31291276	G	ADD	146279	1.141	5.911	3.40E-09
6	rs9265459	31296680	A	ADD	148534	1.142	5.976	2.28E-09
6	rs9265572	31298681	G	ADD	147373	1.141	5.902	3.60E-09
6	rs9265597	31299280	T	ADD	142946	1.141	5.869	4.38E-09
6	rs9265658	31300571	T	ADD	148758	1.14	5.928	3.08E-09
6	rs9265683	31301240	C	ADD	148743	1.141	5.96	2.52E-09
6	rs9265729	31302236	T	ADD	148543	1.139	5.911	3.40E-09
6	rs4959060	31304535	A	ADD	146123	1.168	5.76	8.41E-09
6	rs9295981	31308524	A	ADD	129045	0.8633	-6.055	1.40E-09
6	rs4637682	31312237	A	ADD	148679	0.8749	-5.775	7.71E-09
6	rs2508002	31312863	C	ADD	147158	0.8648	-6.784	1.17E-11
6	rs12175330	31314066	T	ADD	148358	0.8766	-5.778	7.58E-09
6	rs3016018	31314106	G	ADD	148654	0.8648	-6.849	7.43E-12
6	rs2507999	31314646	A	ADD	148695	1.14	5.924	3.14E-09
6	rs2507996	31314921	A	ADD	148706	1.136	5.824	5.75E-09
6	rs9265951	31315270	C	ADD	148349	1.137	6.087	1.15E-09
6	rs9265953	31315312	A	ADD	146780	1.139	6.132	8.70E-10
6	rs9265956	31315363	T	ADD	148056	1.14	5.929	3.04E-09
6	rs9265962	31315520	A	ADD	148607	1.136	5.851	4.90E-09
6	rs9265975	31315891	A	ADD	148505	1.183	7.686	1.51E-14
6	rs1053726	31322047	G	ADD	148349	0.797	-6.523	6.89E-11
6	rs3819306	31322133	G	ADD	147638	0.8427	-7.564	3.90E-14
6	rs2308655	31322303	G	ADD	148783	0.8606	-6.618	3.64E-11
6	rs3819294	31322487	A	ADD	148770	0.7497	-6.317	2.67E-10
6	rs3819292	31322522	A	ADD	148480	0.8143	-6.491	8.50E-11
6	rs3819288	31322694	C	ADD	148700	0.7894	-6.144	8.04E-10
6	rs17193012	31322703	T	ADD	148765	0.7492	-6.329	2.46E-10
6	rs1130992	31322996	T	ADD	148566	0.8791	-5.916	3.31E-09
6	rs17881225	31323065	C	ADD	148637	0.789	-6.154	7.54E-10
6	rs12526858	31323721	C	ADD	148652	0.7467	-6.357	2.06E-10
6	rs12528645	31323725	C	ADD	148648	0.7468	-6.353	2.11E-10

6	rs4999717	31324348	T	ADD	148743	0.7478	-6.344	2.24E-10
6	rs17881210	31324448	A	ADD	148738	0.7443	-6.333	2.40E-10
6	rs1131161	31324891	T	ADD	147131	0.7472	-6.335	2.37E-10
6	rs9266224	31325164	T	ADD	145455	0.8702	-6.07	1.28E-09
6	rs34265426	31325684	A	ADD	148729	0.7493	-6.306	2.87E-10
6	rs35791140	31325795	T	ADD	148708	0.7478	-6.343	2.25E-10
6	6:31326150_CG_C	31326150	C	ADD	146325	0.7567	-6.101	1.06E-09
6	rs35840551	31326151	C	ADD	147337	0.7385	-6.48	9.20E-11
6	rs199724218	31326153	C	ADD	146324	0.7567	-6.101	1.06E-09
6	rs9405085	31326292	G	ADD	148742	0.7491	-6.31	2.78E-10
6	rs9378249	31327701	G	ADD	148634	0.7389	-6.265	3.74E-10
6	rs9380248	31328475	T	ADD	148682	0.7519	-6.221	4.95E-10
6	rs9366782	31328545	T	ADD	148681	0.7519	-6.221	4.95E-10
6	rs9266329	31330788	A	ADD	148507	0.8468	-5.911	3.40E-09
6	rs2523559	31330969	C	ADD	148457	1.163	7.041	1.90E-12
6	rs2523557	31331257	G	ADD	148468	1.163	7.037	1.97E-12
6	rs4959062	31331500	G	ADD	148260	0.8712	-6.247	4.20E-10
6	rs9266344	31331516	C	ADD	147598	0.8697	-6.307	2.84E-10
6	rs9266345	31331517	A	ADD	147594	0.8696	-6.31	2.78E-10
6	rs9266361	31332919	T	ADD	148018	0.8706	-6.273	3.55E-10
6	rs2523545	31333499	G	ADD	148783	1.163	7.048	1.82E-12
6	rs7761068	31333939	G	ADD	148710	0.8695	-6.345	2.23E-10
6	rs144847276	31335416	GA	ADD	148615	0.771	-6.536	6.33E-11
6	rs9295986	31338528	T	ADD	148783	0.8266	-6.987	2.82E-12
6	rs9266520	31341493	C	ADD	146722	0.8604	-7.02	2.21E-12
6	rs9266635	31346979	C	ADD	148763	1.153	5.858	4.69E-09
6	rs9266637	31347045	G	ADD	147732	0.8587	-6.323	2.56E-10
6	rs9266638	31347057	T	ADD	148330	0.8575	-6.383	1.73E-10
6	rs9266642	31347201	T	ADD	148747	0.777	-6.417	1.39E-10
6	6:31347390_CCATT_C	31347390	C	ADD	148752	0.8293	-8.153	3.55E-16
6	rs2244020	31347451	G	ADD	148288	0.8294	-8.139	3.97E-16
6	rs2844541	31347542	T	ADD	148272	1.153	5.863	4.54E-09
6	rs2523491	31347546	C	ADD	148777	0.8339	-6.986	2.82E-12
6	rs2244026	31347550	T	ADD	148756	0.8339	-6.987	2.81E-12
6	rs2244027	31347566	G	ADD	148783	0.8326	-7.051	1.78E-12
6	rs9266658	31347644	A	ADD	148783	0.8355	-5.942	2.82E-09
6	rs4713460	31347798	A	ADD	148780	0.8623	-5.95	2.68E-09
6	rs4713462	31347816	A	ADD	148783	0.862	-5.948	2.72E-09

6	rs9266663	31347982	G	ADD	148782	0.7769	-6.421	1.35E-10
6	rs9266688	31348543	A	ADD	148777	0.777	-6.416	1.40E-10
6	rs9266701	31349030	T	ADD	148728	0.7756	-6.455	1.08E-10
6	rs9266703	31349120	A	ADD	148719	0.7771	-6.408	1.48E-10
6	rs9266722	31349922	T	ADD	148783	0.778	-6.391	1.65E-10
6	rs4293988	31350380	T	ADD	148767	0.7782	-6.384	1.72E-10
6	rs9266729	31350463	G	ADD	148768	0.779	-6.359	2.03E-10
6	rs3016014	31351153	G	ADD	148783	1.191	7.823	5.18E-15
6	rs9266749	31351313	T	ADD	148781	0.7779	-6.394	1.62E-10
6	rs9266766	31351675	C	ADD	148655	0.7769	-6.4	1.56E-10
6	rs34258436	31352722	ATC	ADD	148774	0.7788	-6.366	1.94E-10
6	rs9266774	31352880	T	ADD	148783	0.7781	-6.389	1.67E-10
6	rs9266775	31353417	G	ADD	148765	0.7779	-6.391	1.65E-10
6	rs3128983	31357916	T	ADD	117845	0.8408	-7.35	1.98E-13
6	rs9266788	31358284	C	ADD	140463	1.2	8.444	3.07E-17
6	rs9266790	31358450	A	ADD	146970	1.198	6.201	5.62E-10
6	rs2442734	31358749	C	ADD	100767	0.8581	-5.841	5.18E-09
6	rs9348866	31358955	T	ADD	146977	0.8656	-6.566	5.17E-11
6	6:31359428_AG_A	31359428	A	ADD	148535	0.7676	-5.739	9.51E-09
6	rs2523468	31362891	C	ADD	127389	1.158	6.485	8.90E-11
6	rs9394070	31366295	A	ADD	148699	0.747	-6.019	1.76E-09
6	rs2523455	31367077	A	ADD	148663	1.157	6.031	1.63E-09
6	6:31367318_AAAAC_A	31367318	A	ADD	113161	0.8064	-6.512	7.40E-11
6	rs3763287	31370329	A	ADD	148769	0.7463	-6.043	1.52E-09
6	rs3763288	31370367	A	ADD	148783	0.746	-6.046	1.48E-09
6	rs2507968	31372718	G	ADD	145404	1.168	6.086	1.16E-09
6	rs12174396	31374579	G	ADD	148617	0.7764	-5.879	4.12E-09
6	rs9366783	31375493	C	ADD	148604	0.7771	-5.858	4.68E-09
6	rs114202986	31375917	C	ADD	148616	0.7415	-6.103	1.04E-09
6	rs2523496	31377719	C	ADD	148061	0.819	-9.277	1.75E-20
6	rs6938453	31377793	A	ADD	144547	0.7887	-8.495	1.97E-17
6	rs28436034	31377975	G	ADD	144008	0.8355	-6.284	3.30E-10
6	rs9266806	31378022	C	ADD	146476	0.8082	-7.456	8.95E-14
6	rs9380254	31378335	C	ADD	148783	0.7427	-5.943	2.80E-09
6	rs1063631	31378387	C	ADD	148767	0.8604	-5.988	2.13E-09
6	rs1051786	31378425	G	ADD	148783	0.8632	-5.867	4.43E-09
6	rs2853981	31378768	A	ADD	148689	1.184	7.967	1.62E-15

6	rs1051792	31378977	A	ADD	148772	0.8616	-5.938	2.89E-09
6	rs1051794	31379109	A	ADD	148783	0.8605	-5.986	2.16E-09
6	rs2853977	31379304	A	ADD	148709	1.184	7.969	1.60E-15
6	rs3828878	31379377	G	ADD	148770	0.8615	-5.942	2.82E-09
6	rs3828879	31379391	T	ADD	148718	0.7828	-6.977	3.01E-12
6	rs3828880	31379403	G	ADD	148772	0.8615	-5.943	2.80E-09
6	rs3828881	31379549	T	ADD	148773	0.861	-5.967	2.42E-09
6	rs3828882	31379620	A	ADD	148773	0.861	-5.967	2.42E-09
6	rs1051796	31379773	T	ADD	148055	0.8613	-5.924	3.15E-09
6	rs1131898	31379795	G	ADD	148755	0.8614	-5.946	2.75E-09
6	rs1051798	31379807	T	ADD	148755	0.8614	-5.946	2.75E-09
6	rs1051799	31379823	G	ADD	148755	0.8614	-5.946	2.75E-09
6	rs147915864	31380091	C T	ADD	148612	0.8606	-5.978	2.26E-09
6	rs748049953	31380160	T	ADD	138309	0.7807	-6.343	2.26E-10
6	rs2256174	31380422	A	ADD	148695	1.184	7.962	1.70E-15
6	rs2256183	31380529	A	ADD	148783	1.183	7.923	2.31E-15
6	rs6933734	31380587	A	ADD	148743	0.861	-5.965	2.45E-09
6	rs6933621	31380710	T	ADD	148734	0.8613	-5.954	2.62E-09
6	rs6934311	31380854	T	ADD	148624	0.8617	-5.921	3.20E-09
6	rs9357133	31380897	T	ADD	148735	0.7563	-5.785	7.26E-09
6	rs3132467	31380943	A	ADD	148529	0.8614	-5.932	3.00E-09
6	rs6934187	31380974	G	ADD	148563	0.8614	-5.93	3.03E-09
6	rs6934403	31381130	C	ADD	148587	0.8615	-5.925	3.12E-09
6	rs9266813	31381321	G	ADD	148583	0.8617	-5.916	3.30E-09
6	rs62395263	31381611	T	ADD	148751	0.7743	-6.201	5.61E-10
6	rs3749950	31382585	G	ADD	148752	0.8623	-5.909	3.44E-09
6	rs78470849	31382675	T	ADD	147284	0.8379	-5.732	9.92E-09
6	rs9266823	31382690	G	ADD	148728	0.8617	-5.933	2.97E-09
6	rs9266824	31382770	G	ADD	148765	0.8621	-5.918	3.26E-09
6	rs1882	31382911	G	ADD	148783	0.8657	-6.848	7.47E-12
6	rs9266829	31382964	G	ADD	148723	0.8609	-5.967	2.42E-09
6	rs2596528	31385485	T	ADD	148693	1.183	7.94	2.02E-15
6	rs2596529	31385782	T	ADD	148765	0.7725	-6.255	3.96E-10
6	rs2596530	31387373	G	ADD	148783	1.183	7.95	1.86E-15
6	rs2596531	31387557	C	ADD	148783	1.184	7.993	1.32E-15
6	rs2596532	31387871	C	ADD	148783	0.7785	-6.387	1.69E-10
6	rs2848716	31387967	C	ADD	148783	0.782	-6.286	3.26E-10
6	6:31388175_GT_G	31388175	G T	ADD	146880	1.181	7.796	6.39E-15

6	rs2844511	31389784	A	ADD	148681	1.183	7.943	1.98E-15
6	rs2516448	31390410	T	ADD	148653	1.184	7.98	1.46E-15
6	rs2596468	31392308	G	ADD	148763	0.771	-6.304	2.91E-10
6	rs3093959	31392805	T	ADD	148729	1.159	6.33	2.45E-10
6	rs2524279	31392906	G	ADD	148783	0.7553	-5.835	5.39E-09
6	rs2516451	31397658	G	ADD	148533	0.7738	-6.217	5.06E-10
6	rs2524278	31398784	T	ADD	148755	0.7713	-6.289	3.19E-10
6	6:31400305_AGCTGGCAAGGCCAG_A	31400305	A	ADD	148634	0.7681	-6.363	1.98E-10
6	rs2263323	31401466	T	ADD	148783	0.7516	-5.908	3.46E-09
6	rs3130483	31402356	A	ADD	148507	1.159	6.345	2.22E-10
6	rs55957433	31402383	G	ADD	144909	0.8361	-6.286	3.26E-10
6	rs3128980	31402837	A	ADD	147891	0.8754	-6.125	9.07E-10
6	rs2516449	31404061	G	ADD	148764	1.158	6.32	2.62E-10
6	rs138614581	31404170	AT	ADD	148635	0.78	-5.794	6.87E-09
6	rs2596466	31405617	C	ADD	148756	0.7526	-5.886	3.95E-09
6	rs2844528	31406525	T	ADD	148756	1.158	6.318	2.65E-10
6	rs3749939	31407247	C	ADD	147748	0.8671	-5.763	8.29E-09
6	rs9469008	31409102	G	ADD	148717	0.8607	-5.977	2.28E-09
6	rs9267084	31409605	A	ADD	147953	1.216	7.075	1.49E-12
6	rs2266161	31416025	A	ADD	148774	0.7684	-6.359	2.03E-10
6	rs2524280	31416990	A	ADD	148783	0.7492	-5.98	2.23E-09
6	rs3131624	31418562	T	ADD	148673	1.166	6.745	1.54E-11
6	rs2516455	31420572	G	ADD	148708	1.139	5.743	9.28E-09
6	rs2105957	31421640	G	ADD	148308	0.7616	-6.428	1.29E-10
6	rs3099847	31423060	T	ADD	145087	0.7831	-5.795	6.81E-09
6	rs368333960	31423465	C	ADD	135623	0.8103	-7.029	2.09E-12
6	rs3128984	31424528	G	ADD	147443	0.7565	-5.784	7.31E-09
6	rs3093971	31424607	A	ADD	148660	0.7528	-7.267	3.68E-13
6	rs2523684	31426399	G	ADD	148783	0.7508	-5.943	2.79E-09
6	rs2523650	31449022	T	ADD	148783	1.153	6.451	1.11E-10
6	rs2905752	31450716	A	ADD	148747	1.16	6.828	8.59E-12
6	rs2904789	31452432	C	ADD	148496	1.131	5.818	5.97E-09
6	rs2905739	31452850	G	ADD	148744	1.16	6.813	9.54E-12
6	rs2428501	31453547	A	ADD	148783	1.164	6.992	2.70E-12
6	rs9267246	31455767	A	ADD	145662	1.142	6.146	7.95E-10
6	rs9267247	31455834	A	ADD	148228	1.131	5.742	9.34E-09
6	rs9267257	31456262	A	ADD	148650	1.161	5.777	7.60E-09

6	rs9267260	31456398	A	ADD	148026	1.133	5.821	5.85E-09
6	rs2904786	31456587	C	ADD	146970	1.136	5.937	2.90E-09
6	rs2905732	31456937	G	ADD	147938	1.133	5.826	5.68E-09
6	rs2904782	31457385	T	ADD	144050	1.147	6.273	3.54E-10
6	rs2905729	31457742	A	ADD	148208	1.131	5.763	8.26E-09
6	rs9267293	31458508	G	ADD	148657	1.16	5.763	8.27E-09
6	rs7450417	31459254	G	ADD	148392	1.131	5.755	8.65E-09
6	rs9267306	31459373	A	ADD	148394	1.131	5.755	8.66E-09
6	rs2904787	31459768	A	ADD	148531	1.133	5.823	5.79E-09
6	rs2904783	31459972	C	ADD	148473	1.133	5.851	4.88E-09
6	rs2395038	31460451	T	ADD	148431	1.133	5.844	5.11E-09
6	rs3094006	31461558	T	ADD	148382	1.143	5.853	4.84E-09
6	rs3132461	31480668	G	ADD	148332	1.167	6.105	1.03E-09
6	rs2516403	31480874	C	ADD	148783	1.139	5.979	2.25E-09
6	rs2516401	31480906	G	ADD	148761	1.157	6.536	6.32E-11
6	rs3131633	31480923	A	ADD	148783	1.163	5.894	3.78E-09
6	rs3132458	31481113	T	ADD	148698	1.168	6.157	7.41E-10
6	rs2246871	31481146	C	ADD	148783	1.135	5.906	3.51E-09
6	rs2516398	31481526	G	ADD	148639	1.139	5.996	2.03E-09
6	rs2516493	31482714	T	ADD	148590	1.14	6.02	1.74E-09
6	rs2516492	31482727	A	ADD	148590	1.14	6.02	1.74E-09
6	rs2844494	31483415	C	ADD	148783	1.14	6.032	1.62E-09
6	rs3094001	31484032	T	ADD	148674	1.169	6.237	4.47E-10
6	6:31484731_TCTC_T	31484731	TCTC	ADD	134706	1.166	5.81	6.26E-09
6	6:31486158_GT_G	31486158	GT	ADD	148208	1.16	6.244	4.27E-10
6	rs3130638	31487540	T	ADD	148666	1.181	6.393	1.63E-10
6	rs3093982	31497244	A	ADD	148755	1.18	6.367	1.92E-10
6	6:31498379_CA_C	31498379	CA	ADD	145494	1.168	6.023	1.71E-09
6	rs9267480	31498384	C	ADD	145533	1.168	6.016	1.78E-09
6	rs3093977	31500226	C	ADD	148753	1.181	6.398	1.57E-10
6	6:31514972_CT_C	31514972	CT	ADD	148606	1.182	6.417	1.39E-10
6	rs2516383	31519653	C	ADD	148740	1.157	6.276	3.47E-10
6	rs2516396	31521913	A	ADD	148717	1.157	6.284	3.30E-10
6	rs2857605	31524851	C	ADD	148783	1.171	6.359	2.03E-10
6	rs2516479	31528326	G	ADD	148674	1.134	5.863	4.54E-09
6	rs2516392	31529334	C	ADD	148684	1.133	5.829	5.58E-09

6	rs2516391	31529335	T	ADD	148684	1.133	5.829	5.58E-09
6	rs2516390	31529883	C	ADD	148778	1.133	5.807	6.37E-09
6	rs3094595	31530574	G	ADD	148780	1.133	5.807	6.38E-09
6	6:31530636_CAA_C	31530636	CAA	ADD	147209	1.141	5.934	2.96E-09
6	rs928815	31531215	T	ADD	148783	1.133	5.809	6.29E-09
6	rs3135043	31532193	G	ADD	148781	1.133	5.807	6.35E-09
6	rs3135042	31532254	G	ADD	148781	1.133	5.807	6.35E-09
6	rs2857710	31532553	C	ADD	148773	1.133	5.814	6.10E-09
6	rs2857602	31533378	G	ADD	148783	1.132	5.805	6.45E-09
6	rs2844486	31533870	A	ADD	148769	1.133	5.813	6.12E-09
6	rs2844485	31534206	A	ADD	148769	1.133	5.813	6.12E-09
6	rs1121800	31535074	A	ADD	148757	1.133	5.834	5.42E-09
6	rs2844484	31536224	A	ADD	148783	1.133	5.822	5.80E-09
6	rs2844483	31536796	T	ADD	148710	1.133	5.817	5.99E-09
6	rs746868	31540429	C	ADD	148737	1.133	5.804	6.46E-09
6	rs2736190	31561211	T	ADD	147503	1.151	6.057	1.39E-09
6	rs2844481	31563429	T	ADD	147548	1.151	6.052	1.43E-09
6	rs2857596	31567422	G	ADD	147329	1.149	5.987	2.14E-09
6	rs805284	31682029	A	ADD	148783	1.26	5.827	5.63E-09
6	rs2844456	31864674	C	ADD	148783	1.264	5.894	3.77E-09
6	rs750497811	31866846	GA	ADD	147954	1.281	5.973	2.32E-09
6	rs2844454	31873292	T	ADD	148449	1.265	5.854	4.79E-09
6	rs34562262	32020961	C	ADD	148783	1.267	5.909	3.45E-09
6	rs41268924	32135137	A	ADD	148783	1.263	5.796	6.80E-09
6	6:32147044_AAG_A	32147044	A	ADD	148778	1.264	5.831	5.52E-09
6	rs41268928	32147157	C	ADD	148783	1.262	5.793	6.91E-09
6	rs9391855	32149801	T	ADD	148719	1.27	6.137	8.39E-10
6	rs2070600	32151443	T	ADD	148783	1.273	6.255	3.98E-10
6	rs41268932	32154711	G	ADD	148783	1.265	5.745	9.19E-09
6	rs2022059	32156489	C	ADD	148768	1.274	6.221	4.93E-10
6	rs2856437	32157364	A	ADD	148783	1.272	6.179	6.46E-10
6	rs8192574	32169145	A	ADD	148770	1.269	5.893	3.80E-09
6	rs3096702	32192331	A	ADD	148783	1.168	7.338	2.17E-13
6	rs767016638	32193303	C	ADD	125673	0.8309	-7.552	4.28E-14
6	rs9267853	32195251	T	ADD	147987	1.166	7.172	7.38E-13
6	rs3132949	32195935	C	ADD	148518	0.8748	-5.766	8.11E-09
6	rs9267858	32196575	T	ADD	148186	1.135	5.943	2.80E-09

6	rs7739432	32198474	T	ADD	148293	1.133	5.866	4.47E-09
6	rs9267873	32199352	C	ADD	148783	1.131	5.785	7.27E-09
6	rs9267897	32202817	C	ADD	148763	1.13	5.755	8.68E-09
6	rs9267898	32202935	T	ADD	148755	1.133	5.868	4.41E-09
6	rs9267907	32204926	T	ADD	148765	1.13	5.757	8.57E-09
6	rs6900833	32205863	C	ADD	148737	1.133	5.868	4.42E-09
6	rs9267919	32206103	C	ADD	146302	1.146	6.276	3.47E-10
6	rs6906174	32206399	T	ADD	146017	1.131	5.74	9.46E-09
6	rs113547322	32206619	T	ADD	146844	0.8156	-6.08	1.20E-09
6	rs9267947	32211218	G	ADD	148783	0.863	-6.933	4.13E-12
6	rs9267948	32212233	G	ADD	148783	1.147	6.511	7.46E-11
6	rs9267949	32212237	T	ADD	148212	1.151	6.612	3.80E-11
6	rs9267950	32212241	T	ADD	148175	1.151	6.607	3.93E-11
6	rs768701081	32212462	CTT	ADD	145099	1.142	6.195	5.82E-10
6	rs6913664	32295874	T	ADD	148693	0.8702	-6.546	5.92E-11
6	rs1076712	32300152	C	ADD	148783	0.8777	-5.889	3.88E-09
6	rs1474727	32300638	T	ADD	148756	0.8781	-5.869	4.39E-09
6	rs2273019	32306419	T	ADD	148783	0.8797	-5.807	6.35E-09
6	rs3129902	32310194	A	ADD	148781	0.8774	-5.907	3.48E-09
6	rs3117136	32310811	C	ADD	148782	0.8774	-5.907	3.49E-09
6	rs3132954	32311459	A	ADD	148783	0.8507	-7.411	1.25E-13
6	rs7383143	32312351	T	ADD	148783	0.8774	-5.907	3.49E-09
6	rs4576281	32312650	C	ADD	148782	0.8793	-5.824	5.73E-09
6	rs3117135	32313531	C	ADD	148782	0.8773	-5.912	3.38E-09
6	rs3117134	32313550	C	ADD	148782	0.8773	-5.912	3.38E-09
6	rs35406322	32314182	CG	ADD	148782	0.8773	-5.912	3.38E-09
6	6:32314306_CCTGTCTTTTAAAATA_C	32314306	C	ADD	145994	0.8782	-5.779	7.52E-09
6	rs2395147	32314484	A	ADD	148781	0.8773	-5.913	3.37E-09
6	rs3117131	32314594	T	ADD	148781	0.8773	-5.913	3.37E-09
6	rs3117130	32314877	G	ADD	148730	0.8776	-5.874	4.26E-09
6	rs910050	32315654	C	ADD	148783	0.8772	-5.917	3.27E-09
6	rs969893	32316081	A	ADD	148783	0.8773	-5.912	3.38E-09
6	rs969891	32316415	C	ADD	148766	0.8773	-5.908	3.47E-09
6	rs3117122	32316604	T	ADD	148783	0.8786	-5.841	5.20E-09
6	rs3117121	32316615	G	ADD	148783	0.8773	-5.911	3.40E-09
6	rs3117120	32316804	C	ADD	148783	0.8773	-5.912	3.38E-09

6	rs2076539	32317467	C	ADD	148783	0.8773	-5.909	3.44E-09
6	rs2076537	32317635	A	ADD	148783	0.877	-5.924	3.14E-09
6	rs761189	32317859	C	ADD	148783	0.877	-5.927	3.08E-09
6	rs761188	32317973	G	ADD	148783	0.8777	-5.893	3.79E-09
6	rs2223636	32331209	A	ADD	148777	0.8793	-5.826	5.68E-09
6	rs62404579	32376019	T	ADD	148581	0.7183	-6.566	5.18E-11
6	rs3129963	32380208	G	ADD	148476	0.849	-5.839	5.26E-09
6	rs3129965	32380830	C	ADD	148420	0.8496	-5.811	6.21E-09
6	rs3129967	32381461	T	ADD	146725	0.8717	-5.862	4.56E-09
6	rs3129968	32381472	C	ADD	146734	0.8716	-5.868	4.41E-09
6	rs743862	32381939	C	ADD	146237	0.8388	-7.706	1.30E-14
6	rs143325653	32382272	AAAAGAAGG	ADD	146597	0.8496	-7.652	1.97E-14
6	rs9268543	32384801	T	ADD	147278	1.173	6.174	6.65E-10
6	rs3135369	32387221	A	ADD	148778	0.8602	-5.827	5.63E-09
6	6:32387373_GGAAA_G	32387373	G	ADD	148781	1.155	5.891	3.84E-09
6	rs2395163	32387809	C	ADD	148783	1.155	5.889	3.88E-09
6	rs149900446	32392268	AT	ADD	147800	0.8523	-5.768	8.04E-09
6	rs2157333	32392591	C	ADD	148694	0.8703	-6.038	1.56E-09
6	rs732162	32394913	A	ADD	148715	0.8703	-6.041	1.54E-09
6	rs3129844	32395835	G	ADD	148774	0.8421	-7.272	3.55E-13
6	rs3129845	32396277	G	ADD	148782	0.8455	-7.129	1.02E-12
6	rs3135343	32396313	T	ADD	148782	0.8455	-7.129	1.01E-12
6	rs3129846	32396475	G	ADD	148783	0.8454	-7.13	1.01E-12
6	rs3129847	32396506	G	ADD	148783	0.8421	-7.273	3.51E-13
6	rs3135342	32396615	T	ADD	148783	0.8421	-7.273	3.51E-13
6	rs9268581	32396930	A	ADD	148709	1.15	5.737	9.61E-09
6	rs3129848	32397049	A	ADD	148776	0.842	-7.277	3.41E-13
6	rs3129851	32397712	C	ADD	148775	0.842	-7.278	3.40E-13
6	rs3129853	32398648	A	ADD	148783	0.8402	-7.366	1.75E-13
6	rs3135340	32398872	G	ADD	148781	0.842	-7.28	3.34E-13
6	6:32398917_GTATACACATATACCATATGACA_G	32398917	G	ADD	148496	0.8411	-7.31	2.66E-13
6	rs4988822	32398975	T	ADD	148779	0.842	-7.277	3.41E-13

6	rs4988821	32399022	G	ADD	148777	0.8425	-7.254	4.05E-13
6	rs4988820	32399158	T	ADD	148779	0.8424	-7.258	3.93E-13
6	rs3135339	32399261	G	ADD	148783	0.842	-7.276	3.45E-13
6	rs3129857	32399785	G	ADD	148782	0.8419	-7.281	3.31E-13
6	rs2395172	32399842	C	ADD	148782	0.8419	-7.283	3.27E-13
6	rs3129858	32400520	A	ADD	148783	0.8403	-7.356	1.90E-13
6	rs3129859	32400939	C	ADD	148783	0.8521	-6.922	4.44E-12
6	rs3135337	32401534	T	ADD	148781	0.8419	-7.281	3.32E-13
6	rs5875370	32403240	GT	ADD	148757	0.8416	-7.295	2.99E-13
6	rs983561	32403655	G	ADD	148783	0.8419	-7.281	3.31E-13
6	rs3135398	32403934	T	ADD	148781	0.842	-7.28	3.35E-13
6	rs3129864	32404043	G	ADD	148781	0.842	-7.28	3.33E-13
6	rs3129865	32404048	G	ADD	148781	1.175	5.748	9.02E-09
6	rs5000563	32404135	G	ADD	148783	0.842	-7.28	3.35E-13
6	rs3135396	32404213	T	ADD	148782	0.842	-7.279	3.36E-13
6	rs2395174	32404878	G	ADD	148781	0.842	-7.28	3.35E-13
6	rs2395176	32405062	T	ADD	148781	0.842	-7.279	3.37E-13
6	rs2395177	32405076	C	ADD	148781	0.842	-7.279	3.37E-13
6	rs3129870	32406100	T	ADD	148709	0.8406	-7.341	2.12E-13
6	rs9268632	32406412	G	ADD	148780	0.8408	-7.334	2.23E-13
6	rs9268641	32406887	T	ADD	148777	0.8408	-7.334	2.24E-13
6	rs3129872	32407153	T	ADD	148783	0.8401	-7.365	1.77E-13
6	rs2395179	32407302	G	ADD	148774	0.8441	-7.193	6.35E-13
6	rs2395180	32407310	G	ADD	148766	0.8445	-7.174	7.26E-13
6	rs2395181	32407404	C	ADD	148783	0.8443	-7.184	6.78E-13
6	rs3129873	32407433	C	ADD	148743	0.8411	-7.321	2.47E-13
6	rs3129874	32407440	C	ADD	148724	0.8408	-7.332	2.27E-13
6	rs3129875	32407468	C	ADD	148706	0.844	-7.197	6.18E-13
6	rs3129876	32408012	A	ADD	148783	0.8445	-7.17	7.50E-13
6	rs3129877	32408597	A	ADD	148783	0.8359	-7.575	3.60E-14
6	rs3129878	32408735	C	ADD	148783	0.8381	-7.562	3.97E-14
6	rs3129879	32408907	A	ADD	148782	0.8449	-7.174	7.27E-13
6	6:32409188_GA_G	32409188	G	ADD	148752	0.8358	-7.562	3.96E-14
6	rs3135392	32409242	A	ADD	148783	0.851	-7.349	2.00E-13
6	rs3129881	32409484	T	ADD	148783	0.8357	-7.567	3.83E-14
6	rs7197	32412580	T	ADD	148749	1.178	6.169	6.89E-10
6	rs3129891	32415080	A	ADD	148783	0.8556	-5.762	8.30E-09
6	rs9268805	32423399	G	ADD	147408	0.8378	-8.075	6.72E-16

6	rs9268833	32428062	T	ADD	148783	1.137	5.797	6.75E-09
6	rs9268834	32428079	A	ADD	148761	1.136	5.76	8.39E-09
6	rs9268835	32428115	A	ADD	148783	1.137	5.777	7.61E-09
6	rs9268838	32428715	A	ADD	148780	1.137	5.79	7.06E-09
6	rs9268926	32433067	G	ADD	133591	1.199	7.112	1.15E-12
6	rs2894257	32433276	T	ADD	100141	0.8426	-6.185	6.23E-10
6	rs9268999	32435874	C	ADD	142612	0.8585	-5.824	5.75E-09
6	rs9391873	32436350	G	ADD	122572	1.282	7.469	8.08E-14
6	rs17209747	32441392	T	ADD	125635	1.286	6.707	1.99E-11
6	rs6901903	32442436	C	ADD	130404	0.8598	-5.821	5.85E-09
6	rs9269108	32443223	A	ADD	148736	0.8396	-7.764	8.25E-15
6	rs9269146	32445530	G	ADD	147599	0.8418	-7.654	1.95E-14
6	rs10947290	32446269	A	ADD	146284	0.6939	-7.285	3.22E-13
6	rs373088803	32448052	AG	ADD	136744	1.215	7.449	9.42E-14
6	rs72508409	32451073	G	ADD	114592	0.7598	-6.494	8.36E-11
6	rs9269232	32451760	T	ADD	144249	0.8505	-7.235	4.65E-13
6	rs71565332	32451822	T	ADD	135092	1.197	7.338	2.16E-13
6	rs71565338	32452110	T	ADD	124071	1.226	6.657	2.80E-11
6	rs9269237	32453583	T	ADD	138538	0.8658	-6.245	4.24E-10
6	rs9269250	32454269	T	ADD	111498	0.8448	-6.833	8.30E-12
6	rs9269252	32454293	A	ADD	122840	0.8441	-7.15	8.69E-13
6	rs9269254	32454327	T	ADD	126865	0.8563	-6.599	4.13E-11
6	rs72508413	32454597	G	ADD	140165	0.8359	-7.544	4.55E-14
6	rs73731419	32454694	C	ADD	141631	1.172	5.789	7.10E-09
6	rs148967059	32454755	A	ADD	111315	1.232	6.763	1.35E-11
6	rs181133225	32454764	C	ADD	122078	1.258	5.977	2.27E-09
6	rs73401326	32454783	G	ADD	76547	0.7983	-7.403	1.33E-13
6	rs182965163	32454827	A	ADD	76441	0.7975	-7.423	1.15E-13
6	rs202162787	32454828	G	ADD	76441	0.7975	-7.423	1.15E-13
6	rs142972412	32454837	T	ADD	130102	1.176	5.871	4.33E-09
6	rs145092566	32454838	A	ADD	75474	0.8031	-7.102	1.23E-12
6	rs139029897	32454882	G	ADD	86760	0.8052	-7.756	8.74E-15
6	rs114141268	32454899	G	ADD	137499	1.177	5.92	3.23E-09
6	rs76483802	32454913	G	ADD	130851	0.8674	-5.787	7.19E-09
6	rs139465672	32454920	C	ADD	137542	1.176	5.897	3.71E-09
6	rs149234868	32454921	A	ADD	85396	1.227	7.329	2.32E-13
6	rs73401330	32454968	A	ADD	105312	1.188	5.882	4.06E-09
6	rs75310737	32454993	C	ADD	138531	1.178	5.964	2.45E-09

6	rs75495772	32455718	GC	ADD	123952	1.242	6.188	6.10E-10
6	rs550730214	32455719	T	ADD	123952	1.242	6.188	6.10E-10
6	rs553071835	32455724	T	ADD	123952	1.242	6.188	6.10E-10
6	rs77497538	32455725	G	ADD	123892	1.241	6.167	6.95E-10
6	rs55723336	32457250	G	ADD	94009	1.249	6.678	2.42E-11
6	rs75620297	32457324	C	ADD	90528	1.247	8.009	1.16E-15
6	rs78845660	32457325	T	ADD	89813	1.252	8.126	4.45E-16
6	rs76883199	32457539	C	ADD	143889	1.176	5.935	2.93E-09
6	rs75301863	32457566	T	ADD	144183	1.176	5.95	2.68E-09
6	rs78583156	32457599	T	ADD	90022	1.232	6.877	6.10E-12
6	rs113174592	32457619	C	ADD	89913	1.232	6.855	7.15E-12
6	rs77312051	32457665	A	ADD	96021	1.222	6.732	1.67E-11
6	rs58937009	32457727	A	ADD	89397	1.243	7.932	2.15E-15
6	rs58095553	32457733	G	ADD	89397	1.243	7.932	2.15E-15
6	rs77964335	32457866	T	ADD	140916	1.172	5.77	7.91E-09
6	rs80133970	32457880	A	ADD	140968	1.172	5.778	7.57E-09
6	rs113133909	32457928	T	ADD	109893	0.8398	-6.892	5.48E-12
6	rs534859262	32457931	T	ADD	109893	0.8398	-6.892	5.48E-12
6	rs542536566	32457959	T	ADD	109402	0.8392	-6.899	5.23E-12
6	rs186217224	32457967	C	ADD	109137	0.8389	-6.904	5.04E-12
6	rs180756398	32458010	A	ADD	107659	0.8272	-7.41	1.26E-13
6	rs73401349	32458094	C	ADD	98812	0.8341	-6.949	3.67E-12
6	rs111890942	32458131	C	ADD	92603	0.8281	-7.06	1.66E-12
6	rs78947187	32458169	A	ADD	120457	1.165	5.99	2.09E-09
6	rs72849265	32458208	T	ADD	92918	0.8255	-7.179	7.00E-13
6	rs62405571	32458228	T	ADD	143337	1.171	5.793	6.92E-09
6	rs62405572	32458232	A	ADD	76799	1.27	7.781	7.19E-15
6	rs62405573	32458239	G	ADD	76769	1.27	7.778	7.39E-15
6	rs62405575	32458259	A	ADD	83212	1.264	7.774	7.60E-15
6	rs200402299	32458349	A	ADD	89069	0.8071	-8.01	1.15E-15
6	rs369376349	32458425	G	ADD	72558	1.198	5.885	3.98E-09
6	rs111458169	32458991	A	ADD	137451	1.174	5.79	7.03E-09
6	rs186920750	32459175	T	ADD	140261	1.172	5.788	7.11E-09
6	rs191818300	32459176	A	ADD	140265	1.172	5.785	7.25E-09
6	rs557676328	32459223	T	ADD	140123	1.174	5.841	5.18E-09
6	rs573113955	32459233	G	ADD	140266	1.171	5.748	9.00E-09
6	rs560518806	32459292	A	ADD	136217	1.177	5.909	3.45E-09
6	rs549250780	32459294	T	ADD	136217	1.177	5.909	3.45E-09

6	rs191593025	32459304	T	ADD	136219	1.177	5.91	3.41E-09
6	rs182144635	32459309	T	ADD	136222	1.177	5.911	3.40E-09
6	rs76803697	32459456	A	ADD	140403	1.17	5.742	9.34E-09
6	rs75371622	32459470	T	ADD	140318	1.171	5.759	8.45E-09
6	rs116611418	32459595	G	ADD	101523	0.8396	-6.816	9.35E-12
6	rs79238108	32459807	C	ADD	140387	1.171	5.732	9.94E-09
6	rs116587947	32459889	T	ADD	111063	0.8327	-7.433	1.06E-13
6	rs114146939	32459890	G	ADD	111063	0.8327	-7.433	1.06E-13
6	rs74507717	32459917	G	ADD	140678	1.171	5.753	8.77E-09
6	rs76782015	32459937	T	ADD	134397	1.174	5.792	6.97E-09
6	rs144441217	32460025	G	ADD	140357	1.171	5.739	9.51E-09
6	rs147402766	32460027	G	ADD	140358	1.171	5.74	9.49E-09
6	rs73401361	32460045	G	ADD	140373	1.173	5.745	9.18E-09
6	rs75127169	32460082	G	ADD	140035	1.172	5.748	9.03E-09
6	rs111499705	32460116	A	ADD	140030	1.171	5.734	9.82E-09
6	rs74945164	32460447	A	ADD	140192	1.171	5.756	8.61E-09
6	rs78767260	32460491	A	ADD	140193	1.172	5.776	7.65E-09
6	rs80221542	32460512	T	ADD	140164	1.171	5.762	8.31E-09
6	rs75498325	32460514	C	ADD	140164	1.171	5.762	8.31E-09
6	rs77089552	32460533	A	ADD	140164	1.171	5.762	8.31E-09
6	rs77707664	32460541	G	ADD	140164	1.171	5.762	8.31E-09
6	rs79705649	32460549	T	ADD	140166	1.171	5.746	9.14E-09
6	rs75236519	32460670	C	ADD	90683	0.8273	-6.986	2.82E-12
6	rs76753608	32460766	G	ADD	140073	1.17	5.734	9.81E-09
6	rs190915926	32464521	A	ADD	80691	1.258	6.089	1.14E-09
6	rs375383446	32468702	G	ADD	141995	1.175	5.888	3.92E-09
6	rs111543935	32468718	A	ADD	142033	1.176	5.877	4.17E-09
6	rs540780180	32468744	T	ADD	141989	1.174	5.877	4.18E-09
6	rs139032160	32468894	T	ADD	145403	1.173	5.817	5.98E-09
6	rs73731427	32468906	T	ADD	145403	1.173	5.816	6.04E-09
6	rs115084652	32468978	T	ADD	114175	0.8552	-6.294	3.10E-10
6	rs116048339	32468982	T	ADD	114615	0.856	-6.265	3.74E-10
6	rs116380301	32468983	T	ADD	114615	0.856	-6.265	3.74E-10
6	rs74372324	32468996	A	ADD	143691	1.175	5.872	4.31E-09
6	rs144975609	32468997	A	ADD	115003	0.8546	-6.341	2.28E-10
6	rs141020324	32469001	A	ADD	115003	0.8546	-6.341	2.28E-10
6	rs74197151	32469035	T	ADD	110728	0.8493	-6.519	7.09E-11
6	rs74211958	32469040	G	ADD	110598	0.851	-6.446	1.15E-10

6	rs112457394	32469073	C	ADD	99745	0.8505	-6.292	3.13E-10
6	rs72849276	32469075	C	ADD	143118	1.175	5.872	4.30E-09
6	rs116691706	32469222	C	ADD	142742	1.17	5.766	8.11E-09
6	rs77693147	32469247	C	ADD	142519	1.171	5.777	7.62E-09
6	rs150643306	32469276	AG	ADD	96562	0.8248	-7.231	4.80E-13
6	rs545379090	32469285	T	ADD	107678	0.8503	-6.447	1.14E-10
6	rs72849282	32469335	T	ADD	107957	0.8513	-6.392	1.64E-10
6	rs76317804	32469375	T	ADD	112161	0.853	-6.402	1.53E-10
6	rs75140942	32469389	G	ADD	142561	1.171	5.734	9.83E-09
6	rs75519123	32469404	G	ADD	109275	0.848	-6.584	4.58E-11
6	rs571525080	32469429	A	ADD	109716	0.8497	-6.51	7.53E-11
6	rs532050307	32469431	C	ADD	109715	0.8497	-6.509	7.54E-11
6	rs75073083	32472471	C	ADD	118304	1.215	6.83	8.50E-12
6	rs75153822	32472472	A	ADD	118304	1.215	6.83	8.50E-12
6	rs75393164	32472553	T	ADD	118467	1.214	6.807	9.97E-12
6	rs79601710	32472593	A	ADD	142880	1.175	5.918	3.26E-09
6	rs75176490	32472604	C	ADD	142982	1.174	5.886	3.96E-09
6	rs75427248	32472605	C	ADD	142982	1.174	5.886	3.96E-09
6	rs79265816	32472664	A	ADD	90117	1.194	6.159	7.33E-10
6	rs75424998	32472830	A	ADD	140317	1.171	5.739	9.53E-09
6	rs139038289	32472940	T	ADD	72262	1.226	5.963	2.48E-09
6	rs77930301	32472943	T	ADD	116239	1.181	5.865	4.50E-09
6	rs113299215	32473089	T	ADD	104623	1.18	5.941	2.84E-09
6	rs116274877	32473099	C	ADD	140694	1.172	5.776	7.63E-09
6	rs115428791	32473101	A	ADD	140694	1.172	5.776	7.63E-09
6	rs116758859	32473104	T	ADD	140694	1.172	5.776	7.63E-09
6	rs114561193	32473120	T	ADD	99584	1.183	5.922	3.19E-09
6	6:32473157_TTTAC_T	32473157	TTTAC	ADD	142457	1.176	5.921	3.21E-09
6	rs111894799	32473167	C	ADD	142457	1.176	5.921	3.21E-09
6	rs142633963	32473248	C	ADD	140568	1.17	5.742	9.38E-09
6	rs140656634	32473259	G	ADD	140568	1.17	5.742	9.38E-09
6	rs72849287	32473321	G	ADD	142600	1.174	5.888	3.92E-09
6	rs73403198	32473419	T	ADD	140580	1.173	5.756	8.63E-09
6	rs73729750	32473503	G	ADD	143367	1.172	5.806	6.39E-09
6	rs113310401	32473554	A	ADD	143368	1.173	5.82	5.89E-09
6	rs111344982	32473588	T	ADD	85730	1.23	6.599	4.13E-11

6	rs116001816	32473686	G	ADD	140428	1.172	5.782	7.37E-09
6	rs60993360	32473694	A	ADD	140470	1.174	5.805	6.42E-09
6	rs112365225	32473740	G	ADD	140529	1.173	5.805	6.44E-09
6	rs187933457	32473756	C	ADD	140529	1.173	5.805	6.44E-09
6	rs189917865	32473758	G	ADD	140530	1.173	5.805	6.45E-09
6	rs529539073	32473776	T	ADD	140785	1.171	5.763	8.24E-09
6	rs111570293	32473792	T	ADD	140765	1.171	5.76	8.43E-09
6	rs75796665	32473799	T	ADD	140765	1.171	5.76	8.43E-09
6	rs74588093	32473810	G	ADD	140756	1.171	5.763	8.28E-09
6	rs74569570	32473816	C	ADD	140756	1.171	5.763	8.28E-09
6	rs77932560	32473832	C	ADD	140695	1.171	5.752	8.82E-09
6	rs74696032	32473866	A	ADD	140660	1.171	5.75	8.93E-09
6	rs79747029	32473889	C	ADD	140647	1.171	5.756	8.59E-09
6	rs79184871	32473972	A	ADD	140320	1.171	5.751	8.86E-09
6	rs74930081	32474088	C	ADD	141743	1.179	6.039	1.55E-09
6	rs76659340	32474090	G	ADD	142549	1.176	5.944	2.78E-09
6	rs77017079	32474095	C	ADD	142549	1.176	5.944	2.78E-09
6	rs75800703	32474096	T	ADD	142549	1.176	5.944	2.78E-09
6	rs188738201	32474550	T	ADD	139847	1.171	5.77	7.92E-09
6	rs113601089	32479182	T	ADD	139020	1.177	5.899	3.66E-09
6	rs186804046	32479321	C	ADD	73477	0.8166	-5.939	2.86E-09
6	rs569725476	32479351	T	ADD	73425	0.8188	-5.867	4.43E-09
6	rs558533142	32479366	C	ADD	73425	0.8188	-5.868	4.41E-09
6	rs72508421	32479491	T	ADD	140844	1.17	5.732	9.95E-09
6	rs114278921	32479544	A	ADD	72822	0.8166	-5.839	5.24E-09
6	rs74428884	32479872	C	ADD	142888	1.172	5.808	6.34E-09
6	rs72849291	32479924	C	ADD	143014	1.172	5.815	6.07E-09
6	rs533688361	32480016	T	ADD	87550	0.8509	-5.84	5.22E-09
6	rs185087225	32480024	C	ADD	87446	1.178	5.925	3.13E-09
6	rs114340096	32480262	T	ADD	143175	1.174	5.886	3.95E-09
6	rs112918203	32480282	A	ADD	142935	1.174	5.874	4.27E-09
6	rs112886967	32480285	C	ADD	143177	1.173	5.866	4.46E-09
6	rs58769847	32481122	T	ADD	112365	0.8183	-8.323	8.60E-17
6	rs59447698	32481125	G	ADD	107638	0.8227	-7.641	2.16E-14
6	rs112582516	32481392	T	ADD	109136	0.8128	-8.377	5.45E-17
6	rs72508424	32481590	G	ADD	142493	1.174	5.84	5.23E-09
6	rs112740757	32481965	A	ADD	88937	1.266	8.74	2.34E-18
6	rs113749865	32481973	T	ADD	88413	1.26	8.493	2.01E-17

6	rs12665158	32482007	T	ADD	92948	0.7994	-8.483	2.19E-17
6	rs73729756	32482020	G	ADD	95554	0.7957	-8.789	1.51E-18
6	rs111823263	32482045	G	ADD	95457	0.7974	-8.706	3.15E-18
6	rs111251172	32482056	C	ADD	140785	1.173	5.796	6.78E-09
6	rs148831934	32482067	G	ADD	140784	1.173	5.796	6.78E-09
6	rs192090456	32482101	C	ADD	108033	1.162	5.928	3.06E-09
6	rs146618240	32482102	G	ADD	106645	0.7886	-9.693	3.22E-22
6	rs552461365	32482115	C	ADD	113198	0.8212	-8.194	2.52E-16
6	rs113147593	32482176	C	ADD	140788	1.17	5.739	9.53E-09
6	rs111874278	32482181	C	ADD	140781	1.17	5.737	9.66E-09
6	rs562851322	32482216	A	ADD	140594	1.173	5.811	6.22E-09
6	rs533509110	32482220	C	ADD	140594	1.173	5.811	6.22E-09
6	rs113506020	32482261	T	ADD	140648	1.171	5.742	9.36E-09
6	rs114716760	32482279	A	ADD	140649	1.171	5.74	9.46E-09
6	rs116360287	32482303	G	ADD	85925	1.279	8.695	3.46E-18
6	rs181549666	32482352	A	ADD	123154	0.8444	-7.05	1.79E-12
6	rs556568526	32482353	T	ADD	127567	0.8479	-6.951	3.63E-12
6	rs578020847	32482368	C	ADD	127595	0.8479	-6.952	3.61E-12
6	rs116473597	32482374	T	ADD	127595	0.8479	-6.952	3.61E-12
6	rs115830407	32482484	T	ADD	82075	1.233	7.258	3.94E-13
6	rs112281380	32482990	C	ADD	138648	1.179	5.985	2.16E-09
6	rs112100135	32483024	A	ADD	138648	1.179	5.985	2.16E-09
6	rs145893158	32483045	T	ADD	138648	1.179	5.985	2.16E-09
6	rs112388251	32483060	A	ADD	138656	1.179	5.985	2.17E-09
6	rs79394030	32484625	T	ADD	138440	1.176	5.918	3.26E-09
6	rs76153169	32484651	G	ADD	138446	1.176	5.916	3.30E-09
6	rs62405576	32484706	A	ADD	140633	1.181	6.105	1.03E-09
6	rs113169828	32484743	A	ADD	140630	1.182	6.106	1.02E-09
6	rs190771556	32485893	T	ADD	66117	0.7706	-6.115	9.64E-10
6	rs139188913	32485921	A	ADD	83959	0.8194	-6.616	3.68E-11
6	rs556705917	32486092	T	ADD	136780	1.176	5.918	3.26E-09
6	rs145238941	32486179	T	ADD	135680	1.18	6.005	1.91E-09
6	rs115328397	32486181	A	ADD	135679	1.18	6.006	1.91E-09
6	rs112269347	32486197	G	ADD	98042	1.188	5.921	3.21E-09
6	rs113242918	32486202	G	ADD	135705	1.181	6.051	1.44E-09
6	rs139672306	32486447	G	ADD	130850	1.177	5.849	4.94E-09
6	rs555503640	32486477	T	ADD	102802	0.8048	-8.674	4.17E-18
6	rs114548292	32486491	T	ADD	137930	1.179	5.996	2.02E-09

6	rs188122191	32486511	T	ADD	137660	1.173	5.763	8.26E-09
6	rs116721417	32486517	T	ADD	137686	1.173	5.773	7.81E-09
6	rs111501833	32486545	T	ADD	138415	1.173	5.765	8.19E-09
6	rs547755130	32486547	T	ADD	90181	1.238	7.845	4.33E-15
6	rs565956329	32486548	G	ADD	90192	1.238	7.851	4.13E-15
6	rs116708952	32486590	T	ADD	90881	1.244	8.077	6.66E-16
6	rs142829465	32486625	G	ADD	138617	1.176	5.905	3.52E-09
6	rs147404658	32486632	T	ADD	138617	1.176	5.905	3.52E-09
6	rs116577630	32486639	T	ADD	138614	1.176	5.905	3.52E-09
6	rs187058069	32486665	C	ADD	84562	1.241	7.779	7.33E-15
6	rs115109434	32486667	G	ADD	138605	1.176	5.901	3.62E-09
6	rs535866271	32486668	C	ADD	88594	1.25	8.186	2.70E-16
6	rs113440750	32486682	A	ADD	138601	1.176	5.902	3.58E-09
6	rs143742029	32486683	A	ADD	92410	1.248	8.125	4.47E-16
6	rs150860760	32486709	T	ADD	81987	1.25	7.773	7.67E-15
6	rs539638110	32486760	A	ADD	135057	1.274	6.301	2.95E-10
6	rs138130801	32486866	T	ADD	74160	0.8214	-6.318	2.64E-10
6	rs116011937	32486884	G	ADD	74732	0.8223	-6.302	2.95E-10
6	rs182976814	32486900	T	ADD	117877	1.273	6.391	1.65E-10
6	rs77052909	32487023	A	ADD	118067	1.188	6.027	1.67E-09
6	rs201958319	32487094	T	ADD	97501	1.202	6.289	3.20E-10
6	rs199855998	32487096	A	ADD	97098	1.205	6.37	1.90E-10
6	rs201850026	32487100	T	ADD	97426	1.2	6.237	4.46E-10
6	rs200254403	32487102	G	ADD	91193	1.221	6.731	1.68E-11
6	rs112872773	32487174	G	ADD	119318	1.228	6.002	1.95E-09
6	rs114293611	32487353	T	ADD	77086	1.188	5.784	7.32E-09
6	rs77180119	32487390	T	ADD	136361	1.173	5.759	8.46E-09
6	rs115198947	32487398	C	ADD	136361	1.173	5.759	8.46E-09
6	rs142795234	32487593	T	ADD	81690	1.184	5.744	9.25E-09
6	rs141114903	32487814	C	ADD	94739	1.279	9.385	6.29E-21
6	rs547797725	32487827	G	ADD	98337	1.262	9.133	6.69E-20
6	rs375309052	32487908	A	ADD	56571	0.7745	-5.969	2.39E-09
6	rs73726164	32487937	A	ADD	56582	0.7779	-5.866	4.46E-09
6	rs71547391	32488685	A	ADD	99649	1.259	8.175	2.97E-16
6	rs541218925	32488717	A	ADD	81141	1.244	7.419	1.18E-13
6	rs185236820	32488776	T	ADD	99574	0.8048	-8.64	5.63E-18
6	rs190526848	32488781	A	ADD	99571	0.8047	-8.642	5.52E-18
6	rs140747244	32488784	C	ADD	98310	0.7995	-8.849	8.83E-19

6	rs34265294	32489118	A	ADD	98637	0.8148	-8.086	6.15E-16
6	rs117090280	32489265	A	ADD	94207	1.226	7.857	3.92E-15
6	rs371747477	32489266	C	ADD	88325	1.232	7.71	1.26E-14
6	rs150598903	32489395	A	ADD	86476	1.201	6.695	2.16E-11
6	rs543993279	32489575	G	ADD	102871	1.255	5.897	3.70E-09
6	rs117108573	32489679	T	ADD	138142	1.286	6.371	1.88E-10
6	rs41559714	32489707	C	ADD	130258	1.277	6.284	3.30E-10
6	rs1136778	32489711	G	ADD	138697	1.276	6.189	6.06E-10
6	rs72508440	32489908	G	ADD	85585	1.209	6.501	7.95E-11
6	rs112326475	32489980	G	ADD	85803	1.222	6.862	6.79E-12
6	rs144901015	32489992	G	ADD	86689	1.221	6.923	4.41E-12
6	rs188812628	32489994	G	ADD	86689	1.221	6.923	4.41E-12
6	rs532143340	32490078	C	ADD	84873	1.189	5.918	3.27E-09
6	rs181494824	32490081	G	ADD	84873	1.189	5.918	3.27E-09
6	rs187653175	32490127	C	ADD	74647	1.215	6.401	1.54E-10
6	rs572264676	32490134	T	ADD	88419	1.211	6.912	4.80E-12
6	rs185320691	32490292	C	ADD	125614	1.263	6.18	6.41E-10
6	rs528723549	32490420	A	ADD	87697	1.209	7.083	1.41E-12
6	rs80177780	32490421	C	ADD	98386	1.186	5.861	4.60E-09
6	rs182191708	32490480	A	ADD	86343	1.234	7.73	1.08E-14
6	rs139910587	32490483	A	ADD	87837	1.231	7.717	1.19E-14
6	rs528638684	32490600	G	ADD	90423	1.238	7.823	5.15E-15
6	rs140849564	32490607	A	ADD	89546	1.246	7.817	5.41E-15
6	rs72508441	32490752	G	ADD	79992	1.204	6.255	3.96E-10
6	rs73726176	32490760	A	ADD	88049	1.22	7.262	3.81E-13
6	rs73726177	32490762	C	ADD	88049	1.22	7.262	3.81E-13
6	rs112140494	32490767	C	ADD	101354	1.306	7.012	2.35E-12
6	rs563226460	32490851	T	ADD	102462	0.8082	-8.402	4.37E-17
6	rs551839031	32490868	C	ADD	140777	1.171	5.757	8.59E-09
6	rs566929862	32490870	G	ADD	140777	1.171	5.757	8.59E-09
6	rs534289980	32490871	A	ADD	140777	1.171	5.757	8.59E-09
6	rs116670844	32490925	T	ADD	89786	0.8184	-7.478	7.57E-14
6	rs114795116	32491112	T	ADD	68484	0.8073	-6.429	1.28E-10
6	rs116787876	32491139	G	ADD	68218	0.8055	-6.473	9.59E-11
6	rs143972840	32491242	T	ADD	84456	1.22	6.904	5.07E-12
6	rs115881023	32491453	G	ADD	139068	1.175	5.836	5.36E-09
6	rs73726187	32491461	A	ADD	139034	1.176	5.86	4.62E-09
6	rs112851809	32491633	T	ADD	70032	0.7626	-8.236	1.78E-16

6	rs113854881	32491732	G	ADD	130477	1.279	6.375	1.83E-10
6	rs141142229	32491805	C	ADD	84956	0.8182	-7.04	1.92E-12
6	rs139195061	32491813	A	ADD	84957	0.8183	-7.036	1.97E-12
6	rs115254515	32491895	C	ADD	98514	1.236	8.215	2.12E-16
6	rs116328963	32491938	C	ADD	84328	1.287	8.624	6.48E-18
6	rs145606733	32491959	A	ADD	98583	1.244	8.506	1.79E-17
6	rs111779823	32491965	C	ADD	98557	1.244	8.504	1.83E-17
6	rs549879919	32491969	G	ADD	92294	0.7779	-9.181	4.28E-20
6	rs113091576	32491986	C	ADD	92310	0.7774	-9.204	3.44E-20
6	rs116711613	32492041	C	ADD	94267	1.232	7.964	1.66E-15
6	rs115814063	32492093	T	ADD	92480	1.247	8.211	2.19E-16
6	rs74223623	32492102	C	ADD	93740	1.244	8.188	2.65E-16
6	rs17210637	32492176	A	ADD	61653	1.236	5.988	2.12E-09
6	rs17204294	32492179	T	ADD	83142	1.223	6.679	2.40E-11
6	rs73726194	32492199	G	ADD	83197	1.222	6.654	2.85E-11
6	rs114780493	32492249	G	ADD	133216	1.287	6.548	5.84E-11
6	rs73726198	32492262	T	ADD	83792	1.226	6.782	1.19E-11
6	rs76784864	32492453	C	ADD	140740	1.174	5.849	4.95E-09
6	rs111823185	32492457	T	ADD	84468	1.205	6.442	1.18E-10
6	rs74655967	32492468	G	ADD	140739	1.174	5.849	4.95E-09
6	rs76415507	32492589	T	ADD	94865	1.287	9.599	8.11E-22
6	rs73726200	32492592	A	ADD	140755	1.173	5.827	5.63E-09
6	rs201844955	32492907	T	ADD	110916	1.205	6.324	2.54E-10
6	rs532180052	32492929	C	ADD	110915	1.208	6.386	1.70E-10
6	rs140209363	32492971	C	ADD	104643	0.8423	-6.786	1.16E-11
6	rs570284595	32493008	A	ADD	141134	1.175	5.882	4.06E-09
6	rs537372552	32493009	A	ADD	141134	1.175	5.882	4.06E-09
6	rs58427731	32493017	C	ADD	141051	1.178	5.91	3.42E-09
6	rs570494819	32493038	A	ADD	141112	1.174	5.872	4.31E-09
6	rs371337007	32493146	G	ADD	82234	1.195	5.792	6.95E-09
6	rs543726509	32493208	A	ADD	95887	0.8368	-6.75	1.48E-11
6	rs531043234	32493428	C	ADD	88612	0.7753	-8.388	4.96E-17
6	rs199973717	32493600	A	ADD	87253	1.197	6.472	9.69E-11
6	rs34328528	32493811	C	ADD	122428	1.309	7.157	8.23E-13
6	rs112861810	32494029	T	ADD	88689	1.215	7.087	1.37E-12
6	rs112560257	32494038	C	ADD	86752	1.238	5.906	3.51E-09
6	rs574714830	32494119	G	ADD	81050	1.226	7.002	2.52E-12
6	rs182336149	32494269	C	ADD	84743	1.187	5.935	2.94E-09

6	rs117616320	32494704	A	ADD	81977	0.8136	-6.322	2.59E-10
6	rs200786989	32494716	C	ADD	76299	1.207	6.063	1.34E-09
6	rs200248344	32494937	C	ADD	142826	1.174	5.847	5.01E-09
6	rs530613608	32495889	T	ADD	71316	0.8176	-6.061	1.35E-09
6	rs115711695	32495994	A	ADD	113798	0.8107	-8.591	8.60E-18
6	rs73727909	32496036	T	ADD	110898	0.8253	-7.748	9.33E-15
6	rs112507860	32496083	T	ADD	119305	0.8115	-8.984	2.61E-19
6	rs202075303	32496326	T	ADD	109370	0.8409	-6.6	4.12E-11
6	rs561942189	32496432	G	ADD	116713	0.8068	-9.113	7.99E-20
6	rs374248993	32496534	C	ADD	111131	0.8348	-7.286	3.20E-13
6	rs554443954	32496557	T	ADD	117403	0.8361	-7.448	9.46E-14
6	rs141631850	32496670	A	ADD	83945	1.221	6.892	5.49E-12
6	rs140028862	32496684	C	ADD	83369	1.234	7.012	2.35E-12
6	rs145528327	32496685	A	ADD	83369	1.234	7.012	2.35E-12
6	rs373964410	32497086	C	ADD	87075	1.201	6.503	7.87E-11
6	rs368127976	32497090	T	ADD	99253	1.212	6.427	1.30E-10
6	rs572123865	32497098	G	ADD	87090	1.201	6.503	7.89E-11
6	rs554894601	32497146	C	ADD	95071	1.244	8.248	1.62E-16
6	rs112138727	32497169	G	ADD	89467	0.8328	-6.459	1.05E-10
6	rs76270534	32497232	G	ADD	119345	1.168	6.309	2.82E-10
6	rs552507937	32497354	A	ADD	136807	0.8568	-6.716	1.87E-11
6	rs570739482	32497356	C	ADD	136807	0.8568	-6.716	1.87E-11
6	rs535043779	32497358	A	ADD	136807	0.8568	-6.716	1.87E-11
6	rs148451580	32498626	A	ADD	108088	0.8072	-8.371	5.73E-17
6	rs61433897	32498787	T	ADD	108903	1.239	7.593	3.12E-14
6	rs7771596	32498881	G	ADD	145875	1.176	5.9	3.63E-09
6	rs78569932	32498885	T	ADD	145876	1.176	5.901	3.62E-09
6	rs183703563	32498913	A	ADD	126469	0.8608	-5.824	5.75E-09
6	6:32498931_G_A	32498931	A	ADD	114104	0.8409	-7.176	7.19E-13
6	rs546033671	32498932	G	ADD	111743	0.8532	-5.921	3.20E-09
6	rs148612288	32498953	G	ADD	111669	0.8537	-5.896	3.72E-09
6	rs115724954	32498958	G	ADD	141952	1.175	5.916	3.30E-09
6	rs114164610	32498960	G	ADD	144445	1.17	5.787	7.18E-09
6	rs113620044	32498980	G	ADD	144438	1.171	5.837	5.30E-09
6	rs147478643	32499006	G	ADD	144382	1.173	5.899	3.65E-09
6	rs140006648	32499015	T	ADD	142635	1.172	5.858	4.69E-09
6	rs111359734	32499033	A	ADD	144517	1.176	5.968	2.41E-09
6	rs149189956	32499047	C	ADD	144516	1.176	5.968	2.40E-09

6	rs146130478	32499213	C	ADD	115278	1.174	6.528	6.66E-11
6	rs78247055	32499214	A	ADD	146722	1.172	5.822	5.83E-09
6	rs138961230	32499244	C	ADD	146865	1.175	5.878	4.16E-09
6	rs186325882	32499990	A	ADD	135448	0.8517	-6.744	1.55E-11
6	rs182771482	32500178	C	ADD	130904	0.843	-6.927	4.29E-12
6	rs187316544	32500462	C	ADD	109223	0.8122	-6.605	3.97E-11
6	rs190614023	32500626	G	ADD	106733	0.826	-6.254	4.00E-10
6	rs149589923	32500818	A	ADD	133901	0.852	-7.016	2.28E-12
6	rs184358099	32500832	G	ADD	133894	0.852	-7.019	2.24E-12
6	rs11965298	32500923	G	ADD	133782	0.8522	-7.014	2.32E-12
6	rs113528607	32500928	G	ADD	132611	1.156	6.331	2.44E-10
6	rs78745522	32500941	A	ADD	146675	1.17	5.764	8.23E-09
6	rs115654776	32501015	T	ADD	146666	1.17	5.774	7.73E-09
6	rs79076469	32501124	A	ADD	146473	1.172	5.815	6.07E-09
6	rs76209082	32501127	C	ADD	146470	1.176	5.884	4.01E-09
6	rs2157337	32501144	T	ADD	110846	0.835	-6.25	4.11E-10
6	rs113972770	32501169	C	ADD	145643	1.178	5.964	2.47E-09
6	rs71549248	32501196	C	ADD	145464	1.174	5.891	3.83E-09
6	rs2187821	32501204	T	ADD	140968	0.8534	-7.029	2.08E-12
6	rs111379218	32501211	A	ADD	145558	1.172	5.856	4.75E-09
6	rs73727927	32501214	G	ADD	139256	0.8073	-9.953	2.45E-23
6	rs74205903	32501249	C	ADD	143575	0.8525	-7.139	9.37E-13
6	rs115303308	32501296	A	ADD	133515	0.8534	-6.929	4.25E-12
6	rs80029685	32501300	G	ADD	146587	1.177	6.021	1.73E-09
6	rs114831840	32501301	C	ADD	133583	0.8522	-6.98	2.95E-12
6	rs115202951	32501311	T	ADD	133399	0.8521	-6.988	2.79E-12
6	rs67851010	32501347	G	ADD	128196	1.166	5.77	7.91E-09
6	rs72851020	32501393	C	ADD	146788	1.175	5.95	2.69E-09
6	rs112155110	32501417	T	ADD	116229	0.8605	-6.186	6.16E-10
6	rs74738572	32501420	G	ADD	147641	1.172	5.843	5.13E-09
6	rs74485644	32501421	T	ADD	132576	0.8526	-6.965	3.30E-12
6	rs72851025	32501459	C	ADD	130661	0.8578	-6.642	3.09E-11
6	rs112709345	32501541	T	ADD	116917	0.8689	-5.809	6.27E-09
6	rs72851028	32501615	A	ADD	132792	0.8532	-6.944	3.80E-12
6	rs72851029	32501639	T	ADD	114325	0.864	-5.982	2.21E-09
6	rs66583884	32501652	G	ADD	147668	1.172	5.836	5.34E-09
6	rs34481656	32501664	G	ADD	131236	1.153	6.177	6.53E-10
6	rs115712701	32501737	C	ADD	132946	0.8525	-6.984	2.88E-12

6	rs28453305	32501842	G	ADD	147680	1.172	5.843	5.12E-09
6	rs72851044	32501886	A	ADD	132890	0.8514	-7.038	1.95E-12
6	rs66738149	32501898	C	ADD	132942	0.8519	-7.015	2.30E-12
6	rs74221689	32501936	A	ADD	141043	0.8561	-6.936	4.04E-12
6	rs72851046	32501957	G	ADD	141054	0.8563	-6.927	4.31E-12
6	rs72851047	32501962	T	ADD	141054	0.8563	-6.927	4.31E-12
6	rs5016923	32502028	G	ADD	145576	0.8562	-7.01	2.38E-12
6	rs74937819	32502037	A	ADD	145574	0.8563	-7.008	2.42E-12
6	rs116316731	32502097	T	ADD	145343	0.8545	-7.086	1.38E-12
6	rs114987387	32502131	G	ADD	145361	0.8545	-7.089	1.36E-12
6	rs72851052	32502249	T	ADD	148783	0.8569	-7.016	2.28E-12
6	rs72851053	32502250	G	ADD	147636	0.857	-6.991	2.73E-12
6	rs111626267	32502273	T	ADD	147368	0.8566	-7.005	2.48E-12
6	rs115573660	32502277	A	ADD	147368	0.8566	-7.005	2.48E-12
6	rs75802832	32502295	C	ADD	147020	1.17	5.766	8.13E-09
6	rs115910196	32502304	T	ADD	143732	0.8565	-6.896	5.35E-12
6	rs78182729	32502322	T	ADD	143120	1.137	5.988	2.13E-09
6	rs79881121	32502324	A	ADD	143358	0.8119	-9.846	7.11E-23
6	rs75509903	32502337	A	ADD	147010	1.17	5.774	7.74E-09
6	rs7765211	32502346	A	ADD	133068	0.839	-6.021	1.73E-09
6	rs115104630	32502387	A	ADD	143963	0.8526	-7.081	1.43E-12
6	rs74816744	32502447	A	ADD	147088	1.171	5.81	6.26E-09
6	rs79076719	32502455	C	ADD	147088	1.171	5.81	6.26E-09
6	rs75310651	32502513	A	ADD	147051	1.17	5.777	7.59E-09
6	rs75085141	32502522	T	ADD	147063	1.17	5.761	8.35E-09
6	rs74202712	32502546	G	ADD	141352	0.8569	-6.832	8.38E-12
6	rs74211571	32502566	T	ADD	140060	0.8555	-6.892	5.49E-12
6	rs74929336	32502585	A	ADD	138961	1.137	5.843	5.13E-09
6	rs76940575	32502590	C	ADD	140042	0.8556	-6.885	5.78E-12
6	rs72508463	32502617	A	ADD	147211	1.171	5.817	6.01E-09
6	rs74207695	32502631	C	ADD	142207	0.8543	-6.942	3.87E-12
6	rs75872455	32502638	C	ADD	141963	1.133	5.794	6.86E-09
6	rs562045282	32502681	T	ADD	141924	0.8579	-6.738	1.61E-11
6	rs74197584	32502717	T	ADD	141820	0.8563	-6.818	9.26E-12
6	rs550643609	32502794	A	ADD	142316	0.8527	-7.017	2.27E-12
6	rs72851055	32502814	C	ADD	141013	0.8532	-6.969	3.19E-12
6	rs79338946	32502822	T	ADD	129547	0.8385	-5.866	4.45E-09
6	rs545949191	32502900	A	ADD	135873	0.8558	-6.82	9.10E-12

6	rs113787140	32502901	T	ADD	135533	1.23	9.49	2.32E-21
6	rs72851056	32502944	A	ADD	131005	0.8617	-6.459	1.06E-10
6	rs112349327	32502948	A	ADD	130996	0.8619	-6.448	1.13E-10
6	rs114198395	32502961	T	ADD	129769	0.8663	-6.201	5.60E-10
6	rs201439475	32502968	A	ADD	129774	0.8673	-6.155	7.52E-10
6	rs115663137	32502992	C	ADD	129624	0.8652	-6.25	4.10E-10
6	rs115380393	32503020	T	ADD	130129	0.8553	-6.721	1.81E-11
6	rs71549270	32503039	A	ADD	146854	1.171	5.811	6.21E-09
6	rs71549271	32503050	G	ADD	146854	1.171	5.811	6.21E-09
6	rs116609655	32503059	C	ADD	129980	0.8551	-6.676	2.46E-11
6	rs111247469	32503071	T	ADD	120932	1.189	6.776	1.23E-11
6	rs544527733	32503091	T	ADD	131172	0.8515	-6.884	5.82E-12
6	rs71549272	32503154	A	ADD	128128	1.165	5.775	7.69E-09
6	rs113096063	32503161	T	ADD	126920	1.159	6.217	5.08E-10
6	rs113351668	32503167	A	ADD	126937	1.159	6.211	5.26E-10
6	rs115726960	32503171	A	ADD	130448	0.8521	-6.932	4.14E-12
6	rs111897614	32503177	T	ADD	126967	1.159	6.209	5.32E-10
6	rs115077688	32503287	T	ADD	130200	0.8509	-6.977	3.01E-12
6	rs116132127	32503328	A	ADD	133753	0.8515	-6.95	3.66E-12
6	rs531982544	32503334	C	ADD	133264	0.8519	-6.907	4.94E-12
6	rs115878723	32503335	A	ADD	133260	0.852	-6.9	5.20E-12
6	rs78294251	32503336	A	ADD	133453	1.146	6.045	1.49E-09
6	rs547409849	32503365	T	ADD	132247	0.8519	-6.873	6.31E-12
6	rs75658193	32503375	A	ADD	132472	1.14	5.754	8.74E-09
6	rs116225018	32503380	C	ADD	132372	0.8517	-6.884	5.83E-12
6	rs78806801	32503403	C	ADD	132379	0.8515	-6.894	5.44E-12
6	rs116133469	32503492	G	ADD	132506	0.8551	-6.768	1.31E-11
6	rs115095329	32503497	A	ADD	132701	0.8541	-6.82	9.11E-12
6	rs76034941	32503498	C	ADD	132701	0.8541	-6.82	9.11E-12
6	rs114653292	32503516	G	ADD	139578	0.8574	-6.768	1.31E-11
6	rs114821537	32503522	G	ADD	139597	0.8566	-6.809	9.84E-12
6	rs115296342	32503526	C	ADD	139563	0.8561	-6.831	8.42E-12
6	rs72851060	32503560	G	ADD	138788	0.8563	-6.829	8.55E-12
6	rs79293578	32503561	T	ADD	137864	1.137	5.792	6.95E-09
6	rs115524254	32503565	C	ADD	139349	0.8541	-6.961	3.39E-12
6	rs77339454	32503567	A	ADD	138051	1.14	5.914	3.34E-09
6	rs75744841	32503575	C	ADD	147312	1.172	5.836	5.35E-09
6	rs77734842	32503641	A	ADD	147317	1.171	5.81	6.24E-09

6	rs112177836	32503658	A	ADD	127600	1.161	6.347	2.20E-10
6	rs72851061	32503672	C	ADD	128453	0.8478	-7.093	1.31E-12
6	rs80141235	32503689	A	ADD	146106	1.171	5.791	6.99E-09
6	rs75730517	32503697	A	ADD	130267	1.158	6.385	1.72E-10
6	rs538879571	32503698	A	ADD	129716	0.8505	-6.952	3.61E-12
6	rs114682516	32503702	C	ADD	130617	0.85	-6.985	2.85E-12
6	rs79591888	32503704	A	ADD	131165	1.156	6.316	2.69E-10
6	rs116103612	32503716	A	ADD	140439	0.8573	-6.774	1.25E-11
6	rs114629945	32503717	G	ADD	140438	0.8573	-6.775	1.25E-11
6	rs73727940	32503738	T	ADD	140598	0.8513	-7.113	1.13E-12
6	rs35874713	32503771	G	ADD	146912	1.17	5.758	8.51E-09
6	rs78992700	32503806	A	ADD	146945	1.171	5.797	6.73E-09
6	rs75802100	32503825	T	ADD	146919	1.17	5.767	8.08E-09
6	rs80235202	32503831	G	ADD	123335	1.194	7.078	1.46E-12
6	rs113017160	32503834	G	ADD	134061	0.851	-7.003	2.51E-12
6	rs114302967	32503839	C	ADD	132925	0.8541	-6.812	9.62E-12
6	rs76054694	32503840	T	ADD	134031	0.8513	-6.983	2.89E-12
6	rs76662624	32503880	A	ADD	134175	0.8499	-7.09	1.34E-12
6	rs113900769	32503887	T	ADD	134178	0.8511	-7.033	2.02E-12
6	rs72851063	32503901	T	ADD	130148	0.8493	-7.049	1.80E-12
6	rs74323244	32503921	C	ADD	129000	1.157	6.253	4.03E-10
6	rs72851064	32503928	C	ADD	129677	0.8474	-7.135	9.71E-13
6	rs77048791	32503986	T	ADD	129979	1.153	6.138	8.36E-10
6	rs67894469	32504015	A	ADD	147222	1.17	5.774	7.74E-09
6	rs71549276	32504030	A	ADD	147220	1.17	5.772	7.82E-09
6	rs71549277	32504035	T	ADD	145089	1.173	5.844	5.09E-09
6	rs114840252	32504071	T	ADD	144140	0.8581	-6.819	9.19E-12
6	rs79604868	32504073	A	ADD	143951	0.8609	-6.664	2.66E-11
6	rs79364663	32504085	C	ADD	144269	0.8584	-6.808	9.87E-12
6	rs75174683	32504101	G	ADD	144436	0.8574	-6.86	6.88E-12
6	rs112815590	32504110	T	ADD	144594	0.8577	-6.854	7.20E-12
6	rs67342079	32504133	G	ADD	146778	1.17	5.755	8.66E-09
6	rs66514134	32504179	A	ADD	146771	1.169	5.746	9.12E-09
6	rs72851065	32504213	T	ADD	131998	0.8523	-6.926	4.32E-12
6	rs72851066	32504216	T	ADD	131998	0.8523	-6.926	4.32E-12
6	rs72508464	32504341	T	ADD	132692	0.8517	-6.98	2.96E-12
6	rs78994392	32504362	T	ADD	132732	0.8515	-6.988	2.78E-12
6	rs116405199	32504367	T	ADD	132732	0.8515	-6.988	2.78E-12

6	rs115744566	32504372	T	ADD	147306	1.173	5.866	4.46E-09
6	rs72851070	32504395	G	ADD	147301	1.173	5.865	4.49E-09
6	rs546636941	32504421	T	ADD	130495	0.8518	-6.887	5.70E-12
6	rs116520213	32504424	T	ADD	130495	0.8518	-6.887	5.70E-12
6	rs557288207	32504452	T	ADD	131105	0.8542	-6.775	1.25E-11
6	rs115696138	32504460	C	ADD	130714	0.8541	-6.773	1.26E-11
6	rs116422126	32504500	G	ADD	130266	0.856	-6.666	2.63E-11
6	rs114362560	32504503	A	ADD	130266	0.856	-6.666	2.63E-11
6	rs76509141	32504529	C	ADD	128316	0.8533	-6.766	1.33E-11
6	rs71549279	32504585	T	ADD	147305	1.172	5.821	5.83E-09
6	rs530662953	32504587	C	ADD	130037	0.8528	-6.86	6.88E-12
6	rs72851077	32504624	C	ADD	130119	0.8541	-6.796	1.08E-11
6	rs67388151	32504700	T	ADD	147223	1.17	5.761	8.35E-09
6	rs199506689	32504701	T	ADD	132988	0.8489	-7.11	1.16E-12
6	rs73727944	32504713	T	ADD	132779	0.8499	-7.057	1.71E-12
6	rs67529500	32504757	C	ADD	140285	0.8534	-6.906	5.00E-12
6	rs62405584	32504801	T	ADD	147320	1.172	5.845	5.06E-09
6	rs75088009	32504884	G	ADD	140266	0.8549	-6.929	4.25E-12
6	rs62405587	32504965	C	ADD	147284	1.171	5.797	6.75E-09
6	rs73727946	32504967	C	ADD	140414	0.8636	-6.442	1.18E-10
6	rs72508465	32505141	T	ADD	139841	0.8547	-6.888	5.67E-12
6	rs72508466	32505166	G	ADD	141860	0.8571	-6.798	1.06E-11
6	rs200819822	32505189	G	ADD	141668	0.8573	-6.786	1.15E-11
6	rs114327439	32505202	A	ADD	141540	0.8574	-6.781	1.19E-11
6	rs114084676	32505216	C	ADD	134970	0.8577	-6.683	2.34E-11
6	rs75876652	32505224	T	ADD	126242	1.149	5.842	5.16E-09
6	rs115010442	32505229	C	ADD	134979	0.8579	-6.683	2.34E-11
6	rs74498391	32505267	C	ADD	128543	1.145	5.782	7.40E-09
6	rs67515384	32505271	A	ADD	136513	0.8578	-6.709	1.97E-11
6	rs72508468	32505288	C	ADD	136842	0.8574	-6.736	1.63E-11
6	rs79136305	32505292	G	ADD	128765	1.145	5.773	7.80E-09
6	rs72508469	32505309	C	ADD	144581	0.8636	-6.541	6.11E-11
6	rs571341495	32505326	G	ADD	143913	0.8643	-6.487	8.76E-11
6	rs538727527	32505332	T	ADD	144013	0.8635	-6.525	6.80E-11
6	rs553897781	32505335	A	ADD	144024	0.8634	-6.53	6.59E-11
6	rs77413629	32505374	G	ADD	141818	0.8575	-6.745	1.53E-11
6	rs183777110	32505375	T	ADD	141818	0.8575	-6.745	1.53E-11
6	rs544548886	32505381	T	ADD	141853	0.8585	-6.698	2.11E-11

6	rs150117381	32505388	C	ADD	141850	0.8579	-6.729	1.71E-11
6	rs115673628	32505419	G	ADD	141541	0.8571	-6.757	1.41E-11
6	rs72851099	32505508	C	ADD	147280	1.171	5.791	6.99E-09
6	rs72851100	32505511	C	ADD	135943	0.8536	-6.917	4.60E-12
6	rs72853904	32505575	T	ADD	124310	0.8424	-7.377	1.62E-13
6	rs7743846	32505600	C	ADD	135668	0.8517	-7.003	2.51E-12
6	rs72853905	32505609	A	ADD	146787	1.171	5.794	6.87E-09
6	rs376217655	32505666	T	ADD	140453	0.8609	-6.529	6.61E-11
6	rs79010350	32505668	G	ADD	146656	1.17	5.772	7.83E-09
6	rs28568890	32505767	G	ADD	147175	1.174	5.827	5.66E-09
6	rs114034298	32505797	C	ADD	138917	0.858	-6.684	2.33E-11
6	rs193201719	32505807	T	ADD	138837	0.859	-6.634	3.26E-11
6	rs550051341	32505851	C	ADD	139712	0.8591	-6.622	3.55E-11
6	rs571586981	32505852	A	ADD	139712	0.8591	-6.622	3.55E-11
6	rs185520080	32505872	C	ADD	140524	0.8572	-6.739	1.59E-11
6	rs72853910	32505875	C	ADD	146715	1.17	5.763	8.28E-09
6	rs78912960	32505908	G	ADD	146625	1.169	5.749	8.97E-09
6	rs77375484	32505909	T	ADD	146625	1.169	5.749	8.97E-09
6	rs142526177	32505944	G	ADD	143996	0.8194	-6.551	5.73E-11
6	rs113333416	32506627	C	ADD	146840	1.169	5.755	8.68E-09
6	rs76612829	32506695	G	ADD	146848	1.169	5.734	9.79E-09
6	rs116241189	32506751	A	ADD	137158	0.855	-6.841	7.89E-12
6	rs72853911	32506759	G	ADD	137149	0.8555	-6.817	9.29E-12
6	rs75924712	32506761	A	ADD	136057	1.137	5.75	8.90E-09
6	rs78273580	32506779	T	ADD	135926	1.139	5.829	5.58E-09
6	rs74729326	32506796	T	ADD	133217	1.143	5.93	3.03E-09
6	rs141961165	32506799	T	ADD	137029	0.8094	-9.778	1.40E-22
6	rs74949117	32506846	T	ADD	146854	1.175	5.875	4.23E-09
6	rs189968693	32506863	A	ADD	137348	0.8566	-6.693	2.18E-11
6	rs182987687	32506870	C	ADD	137338	0.8567	-6.687	2.28E-11
6	rs188426982	32506878	A	ADD	137338	0.8567	-6.687	2.28E-11
6	rs191423596	32506888	C	ADD	137347	0.8566	-6.694	2.18E-11
6	rs116715716	32506910	T	ADD	137341	0.8148	-9.455	3.22E-21
6	rs76539678	32506942	C	ADD	147111	1.17	5.771	7.87E-09
6	rs79468266	32506982	A	ADD	145989	1.171	5.779	7.53E-09
6	rs74500484	32506993	G	ADD	145989	1.171	5.779	7.53E-09
6	rs150423066	32506995	A	ADD	138168	0.8554	-6.858	6.96E-12
6	rs148940091	32507101	C	ADD	131130	0.8132	-9.262	2.01E-20

6	rs536387398	32507125	A	ADD	128933	0.8533	-6.389	1.67E-10
6	rs113128948	32507129	T	ADD	114509	0.8131	-8.721	2.77E-18
6	rs573273966	32507131	C	ADD	128928	0.8531	-6.397	1.58E-10
6	rs115562562	32507200	T	ADD	146666	1.173	5.879	4.14E-09
6	rs77687660	32507226	G	ADD	146683	1.171	5.815	6.08E-09
6	rs113795374	32507296	G	ADD	147070	1.169	5.754	8.69E-09
6	rs534024500	32507307	G	ADD	141893	0.862	-6.513	7.38E-11
6	rs112959132	32507310	T	ADD	147114	1.171	5.801	6.59E-09
6	rs529484704	32507478	C	ADD	139886	0.8617	-6.559	5.41E-11
6	rs569509899	32507490	G	ADD	139862	0.8617	-6.561	5.34E-11
6	rs566710022	32507505	T	ADD	141809	0.8599	-6.7	2.08E-11
6	rs547631481	32507531	A	ADD	142448	0.8616	-6.624	3.49E-11
6	rs114580448	32507538	T	ADD	139761	1.134	5.742	9.35E-09
6	rs67542250	32507548	G	ADD	147115	1.169	5.741	9.43E-09
6	rs374408836	32507582	C	ADD	133873	0.8592	-6.603	4.04E-11
6	rs544353633	32507596	G	ADD	134605	0.8596	-6.591	4.36E-11
6	rs77077499	32507652	T	ADD	140306	0.8582	-6.709	1.96E-11
6	rs76325796	32507653	A	ADD	140306	0.8582	-6.709	1.96E-11
6	rs74197204	32507695	G	ADD	132151	0.8625	-6.361	2.01E-10
6	rs79740987	32507712	G	ADD	139280	0.8612	-6.552	5.67E-11
6	rs68009841	32507731	T	ADD	138903	0.8608	-6.556	5.53E-11
6	rs199636459	32507758	A	ADD	140335	0.8579	-6.708	1.97E-11
6	rs540194362	32507768	G	ADD	140341	0.8577	-6.718	1.85E-11
6	rs555383676	32507771	A	ADD	137565	0.8615	-6.477	9.36E-11
6	rs74786163	32507780	T	ADD	127982	0.8583	-6.479	9.24E-11
6	rs66781242	32507812	G	ADD	147662	1.175	5.869	4.39E-09
6	rs72853919	32507852	C	ADD	130268	0.8608	-6.462	1.03E-10
6	rs66924955	32507855	G	ADD	130268	0.8608	-6.462	1.03E-10
6	rs147668426	32507931	G	ADD	140961	0.8579	-6.72	1.81E-11
6	rs571430728	32507938	T	ADD	140956	0.8579	-6.719	1.83E-11
6	rs539226504	32507944	A	ADD	140898	0.8584	-6.695	2.15E-11
6	rs140538649	32507946	T	ADD	140898	0.8584	-6.695	2.15E-11
6	rs555706008	32507953	G	ADD	141322	0.8583	-6.715	1.88E-11
6	rs573629697	32507971	G	ADD	141633	0.8589	-6.7	2.08E-11
6	rs180942055	32507976	T	ADD	141633	0.8589	-6.7	2.08E-11
6	rs186170828	32507977	G	ADD	141633	0.8589	-6.7	2.08E-11
6	rs142393802	32508021	T	ADD	142898	0.8583	-6.747	1.51E-11
6	rs549780202	32508055	T	ADD	142254	0.8587	-6.702	2.06E-11

6	rs564974789	32508061	A	ADD	142254	0.8587	-6.702	2.06E-11
6	rs532618689	32508064	T	ADD	142254	0.8587	-6.702	2.06E-11
6	rs192164737	32508091	G	ADD	142249	0.8587	-6.701	2.08E-11
6	rs66479855	32508187	T	ADD	147418	1.17	5.77	7.92E-09
6	rs556173675	32508202	G	ADD	141564	0.8592	-6.675	2.47E-11
6	rs67342127	32508204	T	ADD	147418	1.17	5.77	7.92E-09
6	rs538718414	32508207	T	ADD	141608	0.8593	-6.67	2.56E-11
6	rs72853923	32508222	G	ADD	141379	0.8585	-6.704	2.02E-11
6	rs72853924	32508239	G	ADD	140666	0.858	-6.724	1.77E-11
6	rs115081905	32508276	T	ADD	141413	0.8523	-7.033	2.02E-12
6	rs74715621	32508285	G	ADD	141451	0.8518	-7.061	1.66E-12
6	rs67860869	32508349	C	ADD	131790	0.8553	-6.742	1.56E-11
6	rs72508473	32508399	G	ADD	130957	0.8536	-6.817	9.27E-12
6	rs202231764	32508412	T	ADD	129213	0.8566	-6.619	3.61E-11
6	rs72853926	32508451	G	ADD	128140	0.8635	-6.248	4.16E-10
6	rs78017592	32508455	C	ADD	119547	1.187	6.589	4.42E-11
6	rs72853927	32508474	G	ADD	128892	0.861	-6.358	2.04E-10
6	rs72853928	32508486	T	ADD	129285	0.8609	-6.376	1.82E-10
6	rs75820413	32508505	G	ADD	131241	0.8627	-6.333	2.40E-10
6	rs62405590	32508559	G	ADD	146240	1.17	5.779	7.50E-09
6	rs373664556	32508561	C	ADD	140910	0.8561	-6.796	1.08E-11
6	rs76376937	32508568	G	ADD	140902	0.8559	-6.805	1.01E-11
6	rs116224274	32508583	C	ADD	140906	0.8564	-6.775	1.25E-11
6	rs77173653	32508587	G	ADD	140902	0.8565	-6.772	1.27E-11
6	rs78278896	32508647	G	ADD	114726	0.8616	-6.023	1.71E-09
6	rs72508474	32508738	C	ADD	131071	0.8583	-6.614	3.73E-11
6	rs62405592	32508757	A	ADD	147653	1.17	5.772	7.85E-09
6	rs115664796	32508758	C	ADD	130756	0.8578	-6.631	3.33E-11
6	rs113648142	32508762	T	ADD	131043	0.8586	-6.597	4.21E-11
6	rs67774869	32508794	C	ADD	139587	0.8606	-6.65	2.94E-11
6	rs67916930	32508823	G	ADD	140049	0.8614	-6.613	3.77E-11
6	rs113209512	32508875	T	ADD	136249	0.8564	-6.735	1.64E-11
6	rs73727954	32508902	T	ADD	136496	0.8567	-6.711	1.94E-11
6	rs72853940	32508945	T	ADD	139594	0.8596	-6.684	2.33E-11
6	rs68122208	32508981	A	ADD	147512	1.169	5.765	8.18E-09
6	rs73727960	32508989	G	ADD	139892	0.8624	-6.561	5.36E-11
6	rs200263528	32509009	C	ADD	139861	0.8607	-6.646	3.02E-11
6	rs111559749	32509012	T	ADD	133961	0.8665	-6.233	4.59E-10

6	rs73727961	32509022	A	ADD	139799	0.8599	-6.689	2.25E-11
6	rs72492303	32509061	T	ADD	139891	0.8589	-6.739	1.60E-11
6	rs67041740	32509273	C	ADD	138211	0.8596	-6.665	2.65E-11
6	rs79207037	32509294	A	ADD	136931	1.142	5.944	2.78E-09
6	rs72492304	32509311	C	ADD	138244	0.8589	-6.699	2.09E-11
6	rs66678933	32509320	T	ADD	138244	0.8589	-6.699	2.09E-11
6	rs75635252	32509332	G	ADD	135584	1.141	5.859	4.66E-09
6	rs79957603	32509348	A	ADD	135598	1.142	5.876	4.20E-09
6	rs28772724	32509357	T	ADD	135185	1.153	6.277	3.46E-10
6	rs66502326	32509411	T	ADD	136737	0.8563	-6.808	9.88E-12
6	rs72853949	32509435	T	ADD	132256	0.8624	-6.387	1.69E-10
6	rs115704383	32509460	T	ADD	138978	0.8574	-6.779	1.21E-11
6	rs114246255	32509461	G	ADD	138978	0.8574	-6.779	1.21E-11
6	rs116413420	32509466	T	ADD	132044	0.8616	-6.413	1.43E-10
6	rs72853950	32509472	T	ADD	138928	0.8579	-6.755	1.43E-11
6	rs72492306	32509510	A	ADD	138803	0.8556	-6.865	6.65E-12
6	rs72492307	32509519	C	ADD	139003	0.8572	-6.791	1.11E-11
6	rs113686014	32509551	A	ADD	136398	1.14	5.831	5.52E-09
6	rs28819746	32509579	C	ADD	139620	0.8538	-6.869	6.46E-12
6	rs68126668	32509603	A	ADD	139089	0.8568	-6.813	9.59E-12
6	rs73727972	32509683	G	ADD	113402	0.8443	-6.953	3.58E-12
6	rs73727973	32509691	G	ADD	139880	0.8568	-6.82	9.13E-12
6	rs113871699	32509693	T	ADD	114127	0.8448	-6.941	3.89E-12
6	rs76965357	32509778	C	ADD	137855	1.15	6.232	4.61E-10
6	rs66521709	32509829	A	ADD	139629	0.8539	-6.86	6.89E-12
6	rs79215033	32509858	G	ADD	140412	1.142	6.064	1.33E-09
6	rs72492310	32509867	G	ADD	143102	0.8577	-6.832	8.39E-12
6	rs74768610	32509868	C	ADD	140409	1.141	6.058	1.38E-09
6	rs72853968	32509883	C	ADD	143113	0.8582	-6.81	9.76E-12
6	rs28844904	32509895	G	ADD	147638	1.169	5.763	8.24E-09
6	rs78942674	32509897	T	ADD	141209	0.8567	-6.853	7.24E-12
6	rs72853970	32509919	A	ADD	141599	0.8567	-6.86	6.88E-12
6	rs59350153	32509935	T	ADD	138519	1.138	5.813	6.13E-09
6	rs72853971	32509938	C	ADD	141609	0.8571	-6.839	7.95E-12
6	rs72492311	32509979	T	ADD	139645	0.8546	-6.832	8.40E-12
6	rs57588792	32510012	A	ADD	138370	1.137	5.767	8.09E-09
6	rs116190325	32510039	A	ADD	134525	0.8572	-6.71	1.95E-11
6	rs115137792	32510114	C	ADD	133507	0.8487	-6.91	4.86E-12

6	rs199616440	32510115	T	ADD	133611	0.849	-6.894	5.42E-12
6	rs537977627	32510140	A	ADD	141363	0.856	-6.888	5.68E-12
6	rs71534520	32510146	G	ADD	140959	0.8529	-7.051	1.78E-12
6	rs58537387	32510176	T	ADD	138353	1.139	5.857	4.70E-09
6	rs111590055	32510177	C	ADD	141219	0.8555	-6.909	4.90E-12
6	rs113085907	32510224	G	ADD	141170	0.8536	-7.003	2.51E-12
6	rs147266303	32510268	C	ADD	140669	0.8532	-7.016	2.29E-12
6	rs28769628	32510281	A	ADD	147653	1.17	5.789	7.09E-09
6	rs112780153	32510301	C	ADD	141791	0.8537	-6.988	2.80E-12
6	rs112111526	32510317	A	ADD	142274	0.8508	-7.148	8.79E-13
6	rs144411837	32510337	C	ADD	142223	0.8539	-6.99	2.75E-12
6	rs140120971	32510343	C	ADD	142136	0.8539	-6.992	2.71E-12
6	rs143822887	32510344	A	ADD	142136	0.8539	-6.992	2.71E-12
6	rs28404276	32510355	G	ADD	138177	1.237	9.837	7.80E-23
6	rs113220298	32510369	T	ADD	140799	0.8536	-6.992	2.71E-12
6	rs112465099	32510430	G	ADD	141084	0.8564	-6.844	7.68E-12
6	rs113623173	32510438	T	ADD	138329	1.137	5.766	8.12E-09
6	rs80083020	32510439	C	ADD	141084	0.8564	-6.844	7.68E-12
6	rs112944992	32510457	C	ADD	141115	0.8567	-6.834	8.26E-12
6	rs115124923	32510482	A	ADD	125818	1.21	7.616	2.62E-14
6	rs116209683	32510508	C	ADD	141593	0.8566	-6.859	6.91E-12
6	rs74535936	32510547	G	ADD	141574	0.8562	-6.878	6.07E-12
6	rs79005639	32510582	C	ADD	138374	1.138	5.835	5.37E-09
6	rs76723065	32510591	G	ADD	141578	0.8559	-6.897	5.32E-12
6	rs114472333	32510598	T	ADD	141434	0.8563	-6.866	6.59E-12
6	rs114015220	32510599	T	ADD	141434	0.8563	-6.866	6.59E-12
6	rs200951960	32510600	G	ADD	124242	1.195	7.185	6.72E-13
6	rs112895453	32510602	G	ADD	138200	1.138	5.811	6.20E-09
6	rs368398419	32510709	C	ADD	143562	0.858	-6.805	1.01E-11
6	rs371989616	32510718	T	ADD	143562	0.858	-6.805	1.01E-11
6	rs138222522	32510733	T	ADD	142848	1.133	5.821	5.84E-09
6	rs112628749	32510769	T	ADD	142743	1.133	5.83	5.56E-09
6	rs113766057	32510772	A	ADD	142744	1.133	5.828	5.60E-09
6	rs112894757	32510777	T	ADD	142733	1.133	5.82	5.87E-09
6	rs536463096	32510951	G	ADD	144380	0.8558	-6.907	4.96E-12
6	rs187395433	32510968	G	ADD	144376	0.8555	-6.927	4.31E-12
6	rs71534526	32510974	C	ADD	144076	0.8153	-9.644	5.20E-22
6	rs541499994	32510983	C	ADD	144019	0.8553	-6.917	4.61E-12

6	rs183134414	32511069	T	ADD	144527	0.8544	-6.988	2.78E-12
6	rs111847217	32511131	G	ADD	141040	0.8562	-6.811	9.72E-12
6	rs111632178	32511149	C	ADD	141036	0.8563	-6.807	9.94E-12
6	rs112427949	32511164	T	ADD	141098	0.8557	-6.838	8.05E-12
6	rs190411898	32511180	T	ADD	139940	1.137	5.82	5.87E-09
6	rs111896611	32511183	T	ADD	142990	0.8569	-6.852	7.30E-12
6	rs71534530	32511211	A	ADD	140897	0.8137	-9.623	6.37E-22
6	6:32511475_G_C	32511475	C	ADD	118340	1.263	6.316	2.68E-10
6	rs74750549	32511488	T	ADD	100784	1.207	6.824	8.86E-12
6	rs72853979	32511572	T	ADD	145054	0.8611	-6.717	1.86E-11
6	rs62405593	32511602	A	ADD	148247	1.171	5.824	5.75E-09
6	rs111797109	32511650	T	ADD	140230	1.148	6.249	4.13E-10
6	rs73727980	32511774	C	ADD	148337	0.859	-6.855	7.13E-12
6	rs73727981	32511791	C	ADD	148783	0.8595	-6.83	8.49E-12
6	rs72853983	32511854	A	ADD	147948	0.8596	-6.818	9.25E-12
6	rs113192265	32511884	C	ADD	129816	1.186	7.11	1.16E-12
6	rs75141783	32511944	A	ADD	147884	0.8599	-6.804	1.02E-11
6	rs72492319	32511987	T	ADD	147836	0.8599	-6.803	1.03E-11
6	rs79202843	32512007	T	ADD	148293	1.171	5.825	5.72E-09
6	rs77789453	32512013	C	ADD	133697	1.148	5.973	2.33E-09
6	rs28584364	32512048	C	ADD	141630	1.229	9.637	5.60E-22
6	rs79508322	32512049	A	ADD	141175	1.135	5.78	7.47E-09
6	rs116675816	32512131	A	ADD	147491	0.8581	-6.881	5.93E-12
6	rs529580232	32512136	C	ADD	147489	0.8581	-6.881	5.95E-12
6	rs551034683	32512138	A	ADD	147489	0.8581	-6.881	5.95E-12
6	rs562852671	32512142	T	ADD	143866	0.8543	-6.952	3.59E-12
6	rs112458679	32512167	G	ADD	147415	0.8594	-6.814	9.47E-12
6	rs28467173	32512180	A	ADD	148102	1.17	5.813	6.12E-09
6	rs534064478	32512189	T	ADD	146910	0.8553	-6.989	2.77E-12
6	rs546382275	32512190	G	ADD	146910	0.8553	-6.989	2.77E-12
6	rs535694878	32512216	A	ADD	130215	0.8143	-9.114	7.92E-20
6	rs556986229	32512217	G	ADD	130215	0.8143	-9.114	7.92E-20
6	rs111725169	32512223	C	ADD	145796	0.8523	-7.122	1.07E-12
6	rs115303801	32512229	G	ADD	146853	0.8554	-6.979	2.98E-12
6	rs111289870	32512269	C	ADD	147398	0.8587	-6.852	7.28E-12
6	rs72853989	32512321	T	ADD	146035	0.8578	-6.875	6.20E-12
6	rs67081540	32512359	C	ADD	143751	0.8549	-6.996	2.63E-12
6	rs66497315	32512407	C	ADD	144512	0.8557	-6.958	3.45E-12

6	rs72492314	32512421	A	ADD	144513	0.8557	-6.958	3.44E-12
6	rs72492315	32512466	C	ADD	144189	0.8549	-6.982	2.92E-12
6	rs72492316	32512481	A	ADD	147735	1.169	5.747	9.09E-09
6	rs72492317	32512501	A	ADD	138021	0.8662	-6.303	2.92E-10
6	rs66857824	32512539	C	ADD	148003	1.177	5.937	2.90E-09
6	rs113522240	32512591	G	ADD	140683	1.145	6.123	9.21E-10
6	rs66534143	32512601	C	ADD	142427	0.8542	-7.018	2.26E-12
6	rs113046257	32512753	T	ADD	140420	1.135	5.734	9.79E-09
6	rs71534539	32512754	A	ADD	90251	0.7874	-8.491	2.05E-17
6	rs113085203	32512789	G	ADD	140430	1.135	5.748	9.02E-09
6	rs116585338	32512812	G	ADD	126053	1.186	6.93	4.20E-12
6	rs76235954	32512815	A	ADD	142450	0.8548	-6.944	3.82E-12
6	rs545256869	32512858	T	ADD	142311	0.8561	-6.88	6.00E-12
6	rs572081630	32512882	T	ADD	140545	1.136	5.752	8.83E-09
6	rs562624040	32513024	C	ADD	144308	0.8564	-6.893	5.45E-12
6	rs74394760	32513078	C	ADD	145351	0.8579	-6.856	7.08E-12
6	rs113568276	32513127	A	ADD	128894	1.176	6.92	4.51E-12
6	rs67499532	32513184	G	ADD	132172	0.8233	-8.727	2.62E-18
6	rs139201366	32513186	A	ADD	132840	0.8705	-5.779	7.52E-09
6	rs72844103	32513249	A	ADD	148394	1.17	5.812	6.16E-09
6	rs72844104	32513250	C	ADD	148394	1.17	5.812	6.16E-09
6	rs74950807	32513375	T	ADD	141670	0.866	-6.341	2.28E-10
6	rs76818741	32513378	A	ADD	141658	0.8659	-6.347	2.20E-10
6	rs112515515	32513422	T	ADD	140581	0.863	-6.448	1.14E-10
6	rs113011827	32513453	T	ADD	127497	1.193	7.24	4.48E-13
6	rs34442507	32513531	T	ADD	148313	1.178	5.995	2.03E-09
6	rs116817148	32513534	G	ADD	144341	0.8548	-6.998	2.59E-12
6	rs114353707	32513535	C	ADD	144341	0.8548	-6.998	2.59E-12
6	rs35997662	32513575	G	ADD	148313	1.178	5.995	2.03E-09
6	rs35301884	32513638	C	ADD	144340	0.8546	-7.006	2.45E-12
6	rs35350417	32513687	C	ADD	148449	1.17	5.814	6.11E-09
6	rs62405598	32513708	C	ADD	143213	1.138	5.876	4.21E-09
6	rs35687625	32513840	A	ADD	144134	0.8547	-7.003	2.50E-12
6	rs34615209	32513862	T	ADD	144134	0.8547	-7.003	2.50E-12
6	rs33998225	32513883	T	ADD	148316	1.178	5.996	2.02E-09
6	rs62405629	32513885	T	ADD	142434	1.138	5.86	4.62E-09
6	rs66706092	32513915	T	ADD	144070	0.8539	-7.044	1.87E-12
6	rs72844111	32513938	C	ADD	144065	0.8542	-7.029	2.08E-12

6	rs62405630	32513942	T	ADD	142414	1.138	5.863	4.56E-09
6	rs34072909	32513966	A	ADD	148451	1.17	5.813	6.13E-09
6	rs73728002	32513993	A	ADD	143184	0.8553	-6.954	3.56E-12
6	rs112587701	32514041	A	ADD	140042	1.149	6.283	3.32E-10
6	rs112158360	32514050	G	ADD	138304	1.14	5.778	7.54E-09
6	rs34369284	32514144	T	ADD	139707	0.8539	-6.866	6.61E-12
6	rs113484779	32514162	T	ADD	142640	1.135	5.76	8.39E-09
6	rs113961302	32514219	G	ADD	142068	1.136	5.758	8.52E-09
6	rs67255973	32514248	A	ADD	148461	1.17	5.812	6.16E-09
6	rs66863443	32514284	A	ADD	148313	1.17	5.787	7.18E-09
6	rs73729106	32514288	T	ADD	126864	0.842	-7.455	8.95E-14
6	rs67571763	32514302	A	ADD	148313	1.17	5.787	7.18E-09
6	rs77783340	32514313	T	ADD	143747	0.8558	-6.936	4.04E-12
6	rs68124732	32514384	T	ADD	148457	1.17	5.812	6.17E-09
6	rs115806221	32514401	G	ADD	143862	0.8558	-6.937	4.00E-12
6	rs62405631	32514500	C	ADD	142820	1.136	5.787	7.18E-09
6	rs72844118	32514539	T	ADD	143742	0.857	-6.877	6.13E-12
6	rs62405633	32514566	T	ADD	147594	1.173	5.92	3.22E-09
6	rs201795916	32514567	C	ADD	143681	0.8573	-6.862	6.81E-12
6	rs62405634	32514623	A	ADD	143371	1.171	5.847	5.01E-09
6	rs73395560	32514671	C	ADD	143257	1.177	5.975	2.29E-09
6	rs114171624	32514706	G	ADD	143673	0.857	-6.872	6.31E-12
6	rs537206245	32514754	C	ADD	137632	1.137	5.764	8.23E-09
6	rs539579029	32514764	T	ADD	137632	1.137	5.764	8.23E-09
6	rs116748457	32514768	G	ADD	126328	0.8426	-7.439	1.01E-13
6	rs185234644	32514792	C	ADD	121105	1.198	7.201	6.00E-13
6	rs574347579	32514817	A	ADD	137745	1.138	5.771	7.89E-09
6	rs116085924	32514884	G	ADD	143043	0.8564	-6.892	5.52E-12
6	rs62405635	32516494	G	ADD	148514	1.17	5.787	7.18E-09
6	rs62405636	32516526	G	ADD	148514	1.17	5.787	7.18E-09
6	rs76033762	32516702	C	ADD	140864	0.8542	-6.977	3.02E-12
6	rs201639066	32516705	T	ADD	135574	1.142	5.917	3.28E-09
6	rs62405637	32516706	C	ADD	148514	1.169	5.763	8.24E-09
6	rs62405638	32516715	T	ADD	135950	1.142	5.907	3.47E-09
6	rs62405639	32516724	C	ADD	135950	1.142	5.907	3.47E-09
6	rs62405640	32516729	T	ADD	140864	0.8542	-6.977	3.02E-12
6	rs113850533	32516839	T	ADD	133134	0.8531	-6.901	5.16E-12
6	rs111901878	32516893	T	ADD	124442	0.8623	-6.281	3.36E-10

6	rs554943670	32516971	G	ADD	114207	1.165	5.82	5.87E-09
6	rs71534598	32516972	T	ADD	148323	1.178	5.991	2.08E-09
6	rs537501315	32516973	A	ADD	107679	1.202	6.42	1.37E-10
6	rs113727677	32517010	C	ADD	121346	0.863	-6.162	7.19E-10
6	rs185282713	32517018	C	ADD	116837	1.158	5.735	9.74E-09
6	rs138087636	32517031	T	ADD	116895	1.158	5.746	9.15E-09
6	rs111945767	32517045	C	ADD	128874	0.8612	-6.407	1.49E-10
6	rs370791576	32517082	A	ADD	134147	1.14	5.798	6.72E-09
6	rs61362782	32517084	C	ADD	148170	1.174	5.841	5.20E-09
6	rs71536503	32517119	G	ADD	148517	1.169	5.765	8.18E-09
6	rs111340421	32517174	G	ADD	137151	0.8574	-6.744	1.54E-11
6	rs192002545	32517227	A	ADD	121107	1.162	6.085	1.17E-09
6	rs183870834	32517228	C	ADD	121105	1.162	6.083	1.18E-09
6	rs74821931	32517233	A	ADD	125131	0.8484	-6.982	2.92E-12
6	rs112300533	32517237	T	ADD	125126	0.8483	-6.984	2.87E-12
6	rs530246116	32517244	C	ADD	121046	1.163	6.11	9.96E-10
6	rs71536505	32517251	G	ADD	148463	1.169	5.775	7.70E-09
6	rs67816706	32517266	T	ADD	118679	0.8376	-7.39	1.46E-13
6	rs67965605	32517293	T	ADD	148473	1.169	5.782	7.38E-09
6	rs111403760	32517303	C	ADD	121318	1.162	6.085	1.16E-09
6	rs116333937	32517381	T	ADD	115845	1.169	5.835	5.37E-09
6	rs114233058	32517386	T	ADD	128396	0.852	-6.885	5.79E-12
6	rs115916707	32517387	G	ADD	128396	0.852	-6.885	5.79E-12
6	rs78055135	32517412	A	ADD	123799	1.162	6.25	4.10E-10
6	rs115712530	32517414	A	ADD	116423	1.175	6.085	1.16E-09
6	rs68175425	32517446	G	ADD	127514	0.8464	-7.122	1.07E-12
6	rs114520912	32517504	T	ADD	135239	0.8488	-7.151	8.61E-13
6	rs116645664	32517505	C	ADD	135239	0.8488	-7.151	8.61E-13
6	rs78733108	32517511	G	ADD	124895	1.149	5.744	9.22E-09
6	rs73729111	32517525	T	ADD	129049	0.8577	-6.574	4.91E-11
6	rs67721312	32517556	T	ADD	135226	0.8486	-7.16	8.05E-13
6	rs74194578	32517590	T	ADD	135364	0.8491	-7.138	9.47E-13
6	rs111365964	32517646	G	ADD	126846	1.174	6.634	3.27E-11
6	rs79890132	32517668	A	ADD	125811	1.149	5.797	6.75E-09
6	rs111838566	32517727	G	ADD	118679	1.159	5.873	4.29E-09
6	rs199882626	32517752	A	ADD	121024	1.153	5.756	8.60E-09
6	rs201326238	32517754	T	ADD	112196	1.176	6.157	7.42E-10
6	rs62405642	32517759	G	ADD	125290	0.8556	-6.624	3.49E-11

6	rs116135263	32517913	A	ADD	104613	1.196	5.809	6.29E-09
6	rs62405644	32517933	T	ADD	148260	1.171	5.824	5.76E-09
6	rs113460787	32518089	T	ADD	128087	0.8561	-6.628	3.41E-11
6	rs75861594	32518103	G	ADD	128244	0.8554	-6.668	2.60E-11
6	rs112004746	32518155	T	ADD	121162	0.8537	-6.644	3.06E-11
6	rs66931410	32518235	C	ADD	136549	0.8076	-9.709	2.77E-22
6	rs113182435	32518236	A	ADD	113900	1.188	6.443	1.17E-10
6	rs111693298	32518251	T	ADD	114531	1.185	6.429	1.29E-10
6	rs113232784	32518256	A	ADD	118382	0.8472	-6.915	4.69E-12
6	rs551468481	32518258	G	ADD	114525	1.188	6.493	8.43E-11
6	rs71536510	32518262	T	ADD	148531	1.17	5.807	6.36E-09
6	rs71536511	32518265	A	ADD	148531	1.17	5.805	6.42E-09
6	rs111649944	32518268	T	ADD	125690	0.853	-6.742	1.56E-11
6	rs147023195	32518295	T	ADD	126418	0.8544	-6.681	2.37E-11
6	rs66468365	32518310	A	ADD	148529	1.18	6.033	1.61E-09
6	rs66482188	32518312	A	ADD	148503	1.171	5.835	5.37E-09
6	rs66884109	32518369	G	ADD	148590	1.179	6.015	1.80E-09
6	rs138825368	32518375	A	ADD	124239	1.15	5.745	9.20E-09
6	rs78622201	32518405	A	ADD	128500	0.8539	-6.749	1.49E-11
6	rs112180912	32518436	A	ADD	128495	0.8539	-6.748	1.50E-11
6	rs115754308	32518466	T	ADD	125342	1.149	5.754	8.70E-09
6	rs28613510	32518507	G	ADD	125455	1.249	9.76	1.67E-22
6	rs28524681	32518584	A	ADD	148413	1.17	5.82	5.89E-09
6	rs114776163	32518625	G	ADD	113102	0.8549	-5.924	3.14E-09
6	rs113787446	32518630	C	ADD	113106	0.8549	-5.926	3.11E-09
6	rs67461697	32518767	C	ADD	99112	0.8109	-6.391	1.65E-10
6	rs74290599	32518785	T	ADD	99108	0.811	-6.388	1.69E-10
6	rs74997284	32518934	G	ADD	116215	0.8469	-6.137	8.40E-10
6	rs78326230	32518935	A	ADD	116215	0.8469	-6.137	8.40E-10
6	rs536708130	32518949	G	ADD	116166	0.8475	-6.112	9.84E-10
6	rs28417075	32519013	T	ADD	137954	1.271	6.215	5.12E-10
6	rs28635588	32519041	G	ADD	148497	1.174	5.812	6.19E-09
6	rs72844144	32519125	A	ADD	110066	0.8408	-6.219	5.00E-10
6	rs66532036	32519319	A	ADD	129272	0.854	-6.74	1.58E-11
6	rs67000090	32519392	G	ADD	148622	1.169	5.763	8.25E-09
6	rs67600647	32519504	G	ADD	148628	1.169	5.766	8.14E-09
6	rs72492322	32519628	G	ADD	100348	0.8321	-5.974	2.31E-09
6	rs72844152	32519652	A	ADD	127865	0.8533	-6.705	2.02E-11

6	rs369883062	32519734	G	ADD	127860	0.8552	-6.647	2.99E-11
6	rs71536515	32519743	A	ADD	148614	1.169	5.785	7.27E-09
6	rs536146370	32519759	A	ADD	126970	0.8507	-6.819	9.15E-12
6	rs73729121	32519768	G	ADD	148582	1.17	5.79	7.04E-09
6	rs73729122	32519777	G	ADD	126979	0.8513	-6.796	1.07E-11
6	rs71536516	32519823	T	ADD	144204	1.174	5.895	3.74E-09
6	rs530370261	32519826	T	ADD	124748	0.8515	-6.674	2.49E-11
6	rs71536517	32519828	T	ADD	148449	1.169	5.778	7.58E-09
6	rs73397403	32519849	G	ADD	125708	0.8546	-6.574	4.90E-11
6	rs115123240	32519919	G	ADD	127735	0.8529	-6.765	1.33E-11
6	rs72492324	32520018	T	ADD	148496	1.168	5.755	8.65E-09
6	rs114031016	32520035	T	ADD	126918	1.19	7.329	2.32E-13
6	rs67717173	32520137	T	ADD	126302	0.8547	-6.642	3.10E-11
6	rs72492325	32520151	C	ADD	127839	0.8537	-6.73	1.69E-11
6	rs116173033	32520295	G	ADD	120637	0.8448	-6.984	2.87E-12
6	rs115217308	32520296	A	ADD	120637	0.8448	-6.984	2.87E-12
6	rs73729131	32520313	T	ADD	120637	0.8448	-6.984	2.87E-12
6	rs67077728	32520338	A	ADD	148690	1.179	6.029	1.65E-09
6	rs71536520	32520362	A	ADD	148460	1.169	5.785	7.24E-09
6	rs72492327	32520465	G	ADD	127039	0.8529	-6.755	1.43E-11
6	rs71536521	32520558	A	ADD	148531	1.168	5.756	8.61E-09
6	rs71536522	32520583	A	ADD	148638	1.168	5.748	9.02E-09
6	rs3828852	32520595	A	ADD	126520	0.8542	-6.672	2.53E-11
6	rs3828848	32520665	C	ADD	124736	0.8608	-6.286	3.26E-10
6	rs66935282	32520683	G	ADD	148750	1.169	5.792	6.97E-09
6	rs66723041	32520707	G	ADD	148783	1.17	5.849	4.95E-09
6	rs376327303	32520719	A	ADD	122056	0.8474	-6.743	1.55E-11
6	rs66758173	32520729	G	ADD	148754	1.178	6.018	1.76E-09
6	rs72844171	32520749	C	ADD	128321	0.854	-6.726	1.74E-11
6	rs61615404	32520785	T	ADD	148723	1.176	5.954	2.61E-09
6	rs58495148	32520816	G	ADD	148783	1.179	6.063	1.33E-09
6	rs73729138	32520854	G	ADD	128043	0.8567	-6.583	4.61E-11
6	rs111343881	32521022	C	ADD	141332	1.138	5.886	3.95E-09
6	rs3828832	32521083	C	ADD	134250	0.86	-6.319	2.63E-10
6	rs3828831	32521117	C	ADD	138807	0.8108	-9.611	7.20E-22
6	rs35957455	32521195	T	ADD	126915	0.8556	-6.568	5.10E-11
6	rs114029309	32521341	G	ADD	88273	1.248	7.458	8.81E-14
6	rs113225456	32521406	T	ADD	122248	0.856	-6.497	8.22E-11

6	rs72844187	32521419	G	ADD	122253	0.8558		-6.505	7.78E-11
6	rs72844188	32521427	C	ADD	122253	0.8558		-6.505	7.78E-11
6	rs66796862	32521664	C	ADD	122262	0.856		-6.496	8.24E-11
6	rs538116343	32521742	T	ADD	109582	0.8602		-5.804	6.48E-09
6	rs73397416	32521951	C	ADD	96505	0.8416		-5.849	4.93E-09
6	rs35697584	32522151	A	ADD	103036	0.8469		-5.894	3.77E-09
6	rs117137018	32522795	A	ADD	114127	1.215		6.476	9.39E-11
6	rs35222480	32522813	C	ADD	62511	0.7936		-5.797	6.77E-09
6	rs35222480	32522813	A	ADD	122408	1.182		5.82	5.87E-09
6	rs73726814	32523235	C	ADD	117073	0.8595		-6.128	8.92E-10
6	rs34405098	32523269	T	ADD	117092	0.8603		-6.091	1.13E-09
6	rs35179057	32523349	C	ADD	107034	0.8584		-5.947	2.73E-09
6	rs187785448	32523396	T	ADD	111634	1.232		6.515	7.28E-11
6	rs79071881	32525686	T	ADD	104199	1.247		6.965	3.28E-12
6	rs188138917	32525964	T	ADD	93879	1.27		6.134	8.56E-10
6	rs28646006	32527793	T	ADD	66630	0.8226		-6.038	1.56E-09
6	rs28819133	32527988	C	ADD	77836	1.195		5.768	8.03E-09
6	rs28793944	32528004	A	ADD	92327	1.189		5.984	2.17E-09
6	rs66777350	32528293	A	ADD	140121	0.8041		-6.995	2.65E-12
6	rs67792106	32528323	G	ADD	140341	0.8048		-6.967	3.25E-12
6	rs66491634	32528457	G	ADD	90726	1.192		6.063	1.33E-09
6	rs68110554	32528475	G	ADD	91486	1.194		6.072	1.26E-09
6	rs66502252	32528521	G	ADD	91239	1.192		5.997	2.01E-09
6	rs28796313	32528538	T	ADD	96896	1.231		7.598	3.01E-14
6	rs28835301	32528570	G	ADD	140895	0.8092		-6.908	4.91E-12
6	rs28848198	32529106	G	ADD	98659	0.789		-7.009	2.40E-12
6	rs113474692	32529177	G	ADD	89638	1.21		6.873	6.28E-12
6	rs28809558	32529190	G	ADD	89725	1.191		6.002	1.95E-09
6	rs78953426	32529417	C	ADD	91877	1.195		6.15	7.75E-10
6	rs68115965	32529669	C	ADD	139540	0.8024		-7.036	1.98E-12
6	rs78506734	32529695	T	ADD	139807	0.8055		-6.93	4.22E-12
6	rs75094512	32529708	T	ADD	92026	1.193		6.061	1.36E-09
6	rs75184770	32529709	G	ADD	91536	1.194		6.104	1.04E-09
6	rs34067190	32529806	T	ADD	141116	0.8056		-6.795	1.08E-11
6	rs35175534	32530029	C	ADD	119737	1.268		6.85	7.38E-12
6	rs34812354	32530046	T	ADD	91948	1.192		6.022	1.72E-09
6	rs35772438	32530407	C	ADD	91287	1.188		5.883	4.04E-09
6	rs67240795	32530458	C	ADD	91373	1.194		6.036	1.58E-09

6	rs67979569	32530540	T	ADD	91298	1.191	5.904	3.55E-09
6	rs76564265	32530582	G	ADD	91232	1.191	5.901	3.62E-09
6	rs35021449	32530583	T	ADD	130170	1.298	6.921	4.47E-12
6	rs66874970	32530748	C	ADD	91534	1.195	6.13	8.78E-10
6	rs67454457	32530767	G	ADD	91510	1.196	6.153	7.59E-10
6	rs67455807	32530881	A	ADD	91229	1.197	6.173	6.68E-10
6	rs67387464	32530892	C	ADD	91182	1.196	6.159	7.31E-10
6	rs66869156	32531295	T	ADD	91013	1.192	5.936	2.92E-09
6	rs67829983	32531335	C	ADD	90991	1.192	5.931	3.01E-09
6	rs71536570	32531422	C	ADD	90959	1.193	5.96	2.53E-09
6	rs67927015	32531442	T	ADD	90956	1.193	5.965	2.44E-09
6	rs116100204	32531556	C	ADD	90531	1.199	6.271	3.59E-10
6	rs143553568	32531583	T	ADD	135983	0.8271	-5.755	8.65E-09
6	rs35603463	32531745	T	ADD	70798	1.234	6.771	1.28E-11
6	rs34105415	32532251	T	ADD	90411	1.189	5.948	2.71E-09
6	rs36054655	32532260	C	ADD	90411	1.189	5.948	2.71E-09
6	rs71536576	32532346	G	ADD	90890	1.189	5.955	2.60E-09
6	rs71536577	32532356	T	ADD	90999	1.19	5.967	2.41E-09
6	rs35115714	32532460	C	ADD	90610	1.192	5.965	2.44E-09
6	rs35855915	32532691	G	ADD	90319	1.193	5.983	2.19E-09
6	rs71536582	32533078	G	ADD	90492	1.196	6.095	1.10E-09
6	rs67336789	32533094	T	ADD	90273	1.193	5.956	2.59E-09
6	rs71536584	32533116	C	ADD	90123	1.191	5.925	3.12E-09
6	rs71536585	32533131	G	ADD	90117	1.191	5.925	3.12E-09
6	rs2284185	32533271	G	ADD	134436	0.8205	-5.8	6.62E-09
6	rs67410451	32533352	A	ADD	90051	1.19	5.923	3.17E-09
6	rs141022812	32533567	G	ADD	88981	1.191	6.03	1.65E-09
6	rs71536590	32533587	A	ADD	90322	1.191	5.943	2.80E-09
6	rs79498062	32533785	A	ADD	90358	1.197	6.202	5.58E-10
6	rs75729173	32533813	C	ADD	90323	1.197	6.193	5.90E-10
6	rs77052117	32533886	C	ADD	90349	1.199	6.236	4.48E-10
6	rs79749596	32534021	T	ADD	78495	1.195	5.87	4.35E-09
6	rs71538515	32535300	A	ADD	89322	1.198	6.193	5.89E-10
6	rs11752277	32537771	G	ADD	100701	1.24	6.827	8.67E-12
6	6:32537804_G_T	32537804	T	ADD	138661	1.291	6.527	6.73E-11
6	rs9269326	32539996	A	ADD	86207	1.194	6.033	1.61E-09
6	rs369515426	32542282	G	ADD	127297	1.195	7.211	5.57E-13
6	rs575662827	32543874	C	ADD	128801	1.273	6.453	1.09E-10

6	rs9269542	32544042	G	ADD	80030	1.222	6.688	2.26E-11
6	rs9269543	32544061	T	ADD	105812	1.195	5.872	4.30E-09
6	rs796139742	32544107	G	ADD	136884	1.292	6.669	2.57E-11
6	rs9269547	32544173	T	ADD	100505	1.188	6.016	1.79E-09
6	rs35511257	32545392	C	ADD	133860	1.273	6.456	1.07E-10
6	rs1141883	32546592	A	ADD	100642	1.204	6.765	1.33E-11
6	rs1064695	32546833	A	ADD	117257	1.196	6.629	3.38E-11
6	rs35445101	32546879	G	ADD	148783	1.134	5.809	6.28E-09
6	rs9269702	32547143	G	ADD	110744	1.198	6.077	1.23E-09
6	rs9256938	32547194	C	ADD	100941	1.179	5.841	5.17E-09
6	rs141267330	32548054	G	ADD	136262	1.303	6.862	6.78E-12
6	rs9269749	32548136	G	ADD	146090	0.8626	-6.571	4.99E-11
6	rs28732284	32548667	C	ADD	143500	1.137	5.776	7.66E-09
6	rs28732291	32548697	G	ADD	143610	1.136	5.735	9.75E-09
6	rs9269771	32548734	T	ADD	107099	1.252	7.938	2.06E-15
6	rs28732302	32548855	C	ADD	146162	1.136	5.853	4.83E-09
6	rs9269792	32549128	C	ADD	99128	1.251	8.107	5.20E-16
6	rs9269798	32549296	A	ADD	134363	1.179	5.829	5.58E-09
6	rs9269799	32549313	G	ADD	143409	1.232	9.683	3.56E-22
6	rs701831	32549401	T	ADD	117050	1.183	5.734	9.80E-09
6	rs112796209	32549531	C	ADD	116489	1.251	6.602	4.06E-11
6	rs1059351	32549540	T	ADD	123316	1.224	7.728	1.10E-14
6	rs35095850	32549935	G	ADD	145485	1.143	6.116	9.58E-10
6	rs34835435	32549966	G	ADD	135867	1.152	6.133	8.60E-10
6	rs28723989	32550116	T	ADD	139693	1.14	5.784	7.31E-09
6	rs35789108	32550126	G	ADD	109217	1.173	6.266	3.71E-10
6	rs33927408	32550420	T	ADD	123992	1.188	6.803	1.02E-11
6	rs28724008	32550550	G	ADD	148783	1.132	5.735	9.78E-09
6	rs9269873	32550796	A	ADD	125535	0.86	-6.351	2.14E-10
6	rs28724031	32550819	A	ADD	145463	1.139	5.946	2.75E-09
6	rs9269915	32551453	G	ADD	125849	1.252	8.961	3.23E-19
6	rs1064664	32552075	G	ADD	95962	0.8373	-6.202	5.59E-10
6	rs9269968	32552251	G	ADD	98150	1.21	7.023	2.17E-12
6	rs34951355	32552416	A	ADD	130856	1.167	6.472	9.67E-11
6	rs113600317	32553125	T	ADD	146378	1.137	5.891	3.85E-09
6	rs9270051	32553751	T	ADD	143998	1.178	5.822	5.81E-09
6	rs9270052	32553807	G	ADD	104912	1.18	5.935	2.94E-09
6	rs200771848	32553816	T	ADD	145371	1.142	6.042	1.52E-09

6	rs113322920	32553849	C	ADD	135181	1.195	7.034	2.01E-12
6	rs9270081	32554243	T	ADD	98989	1.198	6.292	3.14E-10
6	rs113608847	32554284	G	ADD	126426	1.274	8.718	2.84E-18
6	rs9270104	32554726	A	ADD	133057	1.242	8.673	4.22E-18
6	rs76533559	32554897	G	ADD	131866	0.8534	-6.486	8.80E-11
6	rs9270123	32555032	G	ADD	94712	1.21	6.645	3.04E-11
6	rs9270126	32555052	T	ADD	132466	0.86	-6.354	2.10E-10
6	rs13207809	32555239	C	ADD	128145	1.271	7.073	1.52E-12
6	6:32555367_C_A	32555367	A	ADD	133826	0.528	-6.301	2.96E-10
6	rs9270152	32555376	T	ADD	69851	0.7651	-8.243	1.68E-16
6	rs9270154	32555389	G	ADD	92749	0.8133	-5.906	3.50E-09
6	rs34977123	32555736	G	ADD	124163	1.248	6.754	1.44E-11
6	rs34578704	32555779	A	ADD	132028	1.287	8.286	1.18E-16
6	rs5000802	32555832	C	ADD	89122	1.192	6.163	7.13E-10
6	rs9270182	32555833	A	ADD	137773	1.182	5.873	4.29E-09
6	6:32555855_CTTT_C	32555855	C	ADD	134179	0.8707	-5.988	2.13E-09
6	rs145244672	32556461	G	ADD	121469	1.25	6.134	8.54E-10
6	rs35331833	32556635	A	ADD	128948	1.156	6.111	9.90E-10
6	rs113650892	32557163	A	ADD	121082	1.201	7.078	1.46E-12
6	rs9270270	32557208	T	ADD	134888	1.183	5.731	9.99E-09
6	rs566639334	32557269	GGT	ADD	135585	0.8592	-6.447	1.14E-10
6	rs201931294	32557626	A	ADD	120455	1.209	5.812	6.18E-09
6	rs9270363	32558137	T	ADD	137941	0.8714	-6.003	1.94E-09
6	rs9270387	32558317	C	ADD	116449	0.8578	-6.301	2.95E-10
6	rs367562112	32558766	AACAG	ADD	140521	1.248	8.173	3.00E-16
6	rs9270454	32558862	G	ADD	142753	1.18	5.879	4.12E-09
6	rs77122291	32559019	A	ADD	147682	1.134	5.784	7.29E-09
6	rs115879259	32559213	T	ADD	131056	1.27	7.592	3.15E-14
6	6:32559256_TG_T	32559256	T	ADD	144053	0.8681	-6.274	3.53E-10
6	rs34958241	32559575	G	ADD	137517	1.231	7.485	7.18E-14
6	rs34784936	32559648	T	ADD	137442	1.233	7.557	4.13E-14
6	rs9270529	32559658	T	ADD	137435	0.8745	-5.809	6.28E-09
6	rs9270530	32559686	A	ADD	137701	0.8733	-5.874	4.24E-09
6	rs34066151	32559702	G	ADD	137607	1.236	7.668	1.74E-14
6	rs9270533	32559709	T	ADD	137922	0.873	-5.893	3.79E-09

6	rs35299998	32559710	T	ADD	137607	1.236	7.668	1.74E-14
6	rs9270535	32559714	A	ADD	137921	0.873	-5.893	3.79E-09
6	rs9270538	32559758	A	ADD	144870	0.8633	-6.521	6.98E-11
6	rs28366267	32559781	C	ADD	148096	1.134	5.771	7.89E-09
6	rs34855541	32559825	G	ADD	148107	1.227	8.053	8.06E-16
6	rs9270540	32559848	C	ADD	144555	0.8624	-6.56	5.40E-11
6	rs9270541	32559864	T	ADD	147345	0.8646	-6.511	7.46E-11
6	rs28366268	32559874	G	ADD	145398	1.135	5.79	7.02E-09
6	rs9270543	32559881	A	ADD	144623	0.8638	-6.491	8.54E-11
6	rs9270544	32559882	T	ADD	144623	0.8638	-6.491	8.54E-11
6	rs35743245	32559904	T	ADD	146039	1.224	7.864	3.72E-15
6	rs9270547	32559951	G	ADD	145273	0.8629	-6.553	5.63E-11
6	rs9270548	32559960	T	ADD	145265	0.8631	-6.544	6.01E-11
6	rs73729339	32560017	A	ADD	144700	0.8627	-6.545	5.95E-11
6	rs36096565	32560025	G	ADD	135277	1.218	7.61	2.75E-14
6	rs35972518	32560026	G	ADD	146302	1.219	7.735	1.04E-14
6	rs35917796	32560028	T	ADD	146340	1.219	7.743	9.71E-15
6	rs35380574	32560051	T	ADD	146382	1.219	7.751	9.13E-15
6	rs74207277	32560091	T	ADD	144830	0.8628	-6.543	6.01E-11
6	rs73729340	32560155	A	ADD	145123	0.863	-6.54	6.13E-11
6	6:32560182_AGTCACTATT_A	32560182	A	ADD	147524	1.225	7.956	1.78E-15
6	rs35395738	32560209	C	ADD	139391	1.208	7.343	2.08E-13
6	rs71542413	32560240	T	ADD	145257	0.8623	-6.575	4.87E-11
6	rs71542414	32560247	G	ADD	145277	0.8618	-6.599	4.13E-11
6	6:32560270_CAGG_C	32560270	C	ADD	145405	0.8612	-6.636	3.23E-11
6	rs71542415	32560298	C	ADD	145377	0.8618	-6.603	4.03E-11
6	rs74921648	32560307	A	ADD	145415	0.8614	-6.623	3.52E-11
6	rs36207014	32560332	A	ADD	145445	0.8611	-6.637	3.20E-11
6	rs34539464	32560339	G	ADD	145404	0.8608	-6.65	2.94E-11
6	rs79966773	32560341	C	ADD	137491	0.8506	-6.156	7.48E-10
6	rs35472547	32560370	T	ADD	147238	1.224	7.962	1.69E-15
6	rs34291045	32560385	T	ADD	129294	1.288	7.783	7.06E-15
6	rs34939562	32560388	A	ADD	147255	1.224	7.94	2.03E-15
6	rs9270550	32560441	G	ADD	142617	0.8662	-6.328	2.49E-10
6	rs34924558	32560446	T	ADD	142839	1.225	7.581	3.44E-14
6	rs34496598	32560455	T	ADD	142914	1.224	7.556	4.15E-14
6	rs34415150	32560477	G	ADD	145161	1.244	8.408	4.15E-17

6	rs9270556	32560606	A	ADD	147785	0.8636	-6.563	5.28E-11
6	rs35118762	32560631	T	ADD	148311	1.224	7.965	1.66E-15
6	rs9270558	32560667	A	ADD	142078	0.8674	-6.254	4.00E-10
6	rs34928543	32560695	C	ADD	148178	1.225	7.988	1.38E-15
6	rs9270559	32560698	G	ADD	141739	0.8662	-6.328	2.48E-10
6	rs28366296	32560727	A	ADD	141007	1.147	6.1	1.06E-09
6	rs6910879	32560739	G	ADD	137841	1.289	6.935	4.06E-12
6	rs2760975	32560771	A	ADD	120828	1.239	6.757	1.41E-11
6	rs28366302	32560934	C	ADD	148783	1.133	5.749	8.98E-09
6	rs28366303	32560963	G	ADD	148460	1.133	5.757	8.55E-09
6	rs9270570	32560978	G	ADD	146935	0.8646	-6.495	8.29E-11
6	6:32560993_TA_T	32560993	T	ADD	148515	1.134	5.791	7.01E-09
6	rs28366307	32560995	C	ADD	148515	1.134	5.791	7.01E-09
6	rs9270576	32561068	T	ADD	124737	0.856	-5.982	2.21E-09
6	rs9270580	32561123	C	ADD	143130	0.8639	-6.474	9.55E-11
6	rs35074855	32561140	G	ADD	130577	1.297	7.442	9.93E-14
6	rs28366312	32561175	C	ADD	137983	1.149	6.021	1.73E-09
6	rs28366313	32561186	A	ADD	137248	1.163	6.525	6.78E-11
6	rs28366314	32561201	G	ADD	134097	1.177	6.827	8.69E-12
6	rs28366315	32561207	T	ADD	145926	1.139	5.938	2.89E-09
6	rs9270583	32561229	G	ADD	137843	0.8589	-6.641	3.11E-11
6	rs28366316	32561246	C	ADD	140173	1.152	6.266	3.69E-10
6	rs35525122	32561283	A	ADD	148563	1.225	8.006	1.19E-15
6	rs9270585	32561300	C	ADD	148196	1.163	7.134	9.78E-13
6	rs9270588	32561327	C	ADD	147827	1.159	6.982	2.90E-12
6	rs35265698	32561334	G	ADD	148218	1.224	7.971	1.57E-15
6	rs9270589	32561390	T	ADD	147961	0.8666	-6.425	1.32E-10
6	rs9270590	32561392	A	ADD	146459	0.8631	-6.54	6.14E-11
6	rs9270591	32561424	C	ADD	146046	1.162	7.061	1.65E-12
6	rs9270592	32561429	T	ADD	145697	0.8627	-6.543	6.05E-11
6	rs9270593	32561445	G	ADD	145602	0.8629	-6.532	6.51E-11
6	rs34350244	32561465	T	ADD	144885	1.234	8.238	1.75E-16
6	rs35294087	32561466	G	ADD	148211	1.224	7.97	1.59E-15
6	6:32561468_TA_T	32561468	T	ADD	148472	1.226	8.013	1.12E-15
6	rs28366319	32561495	A	ADD	148194	1.133	5.753	8.75E-09
6	rs34535888	32561527	C	ADD	148405	1.227	8.05	8.28E-16
6	rs34553045	32561565	C	ADD	140053	1.264	8.55	1.23E-17
6	rs76288224	32561566	G	ADD	147182	0.7661	-5.78	7.48E-09

6	rs35371668	32561638	T	ADD	142445	1.246	8.211	2.19E-16
6	rs28366327	32561678	A	ADD	148526	1.134	5.78	7.48E-09
6	rs34647096	32561681	A	ADD	148391	1.224	7.974	1.53E-15
6	rs28366328	32561766	G	ADD	148305	1.133	5.76	8.43E-09
6	rs188575117	32561935	C	ADD	135943	1.249	7.701	1.35E-14
6	rs28366358	32565164	G	ADD	133042	1.161	6.345	2.22E-10
6	rs2760980	32565201	A	ADD	141865	1.221	7.359	1.86E-13
6	rs2760981	32565465	A	ADD	125419	0.8527	-6.911	4.82E-12
6	rs2760983	32565601	G	ADD	146977	1.232	8.12	4.67E-16
6	rs113134061	32566041	TCCTC	ADD	148312	1.227	8.056	7.91E-16
6	rs62405651	32566296	C	ADD	147570	0.6113	-7.949	1.88E-15
6	rs2760984	32566329	C	ADD	142250	1.246	8.164	3.23E-16
6	rs2760985	32566398	A	ADD	148314	1.227	8.049	8.33E-16
6	rs2454139	32566635	C	ADD	143484	1.224	7.565	3.89E-14
6	rs687308	32567256	T	ADD	148348	1.226	8.046	8.54E-16
6	rs142264362	32567283	A	ADD	148557	0.6186	-8.094	5.75E-16
6	rs9689000	32567308	G	ADD	148433	0.6875	-7.125	1.04E-12
6	rs112481371	32567551	G	ADD	148045	0.6896	-6.985	2.85E-12
6	rs62405652	32567784	C	ADD	148032	0.6886	-7.005	2.48E-12
6	rs35117964	32568146	G	ADD	139722	1.242	8.026	1.00E-15
6	rs9270747	32568292	A	ADD	142247	1.262	10.43	1.78E-25
6	rs34117221	32568324	G	ADD	142327	1.227	7.573	3.65E-14
6	rs34331363	32568439	T	ADD	138744	1.217	6.959	3.43E-12
6	rs35226637	32568448	A	ADD	137307	1.221	7.045	1.85E-12
6	rs35653258	32568783	A	ADD	138688	1.218	6.983	2.89E-12
6	rs537365538	32569261	A	ADD	148464	0.6886	-7.111	1.15E-12
6	rs2647059	32569362	C	ADD	144494	1.218	7.438	1.02E-13
6	rs576777795	32569435	CA	ADD	140244	0.7637	-7.464	8.41E-14
6	rs9270806	32569563	T	ADD	146818	0.861	-6.693	2.18E-11
6	rs9270807	32569572	A	ADD	146821	0.861	-6.694	2.18E-11
6	rs11757382	32569637	C	ADD	147269	0.7999	-6.662	2.71E-11
6	rs477515	32569691	A	ADD	148353	1.14	6.025	1.69E-09
6	rs6938929	32569712	A	ADD	146019	0.7991	-6.589	4.42E-11
6	rs476775	32569722	T	ADD	147092	0.8613	-6.687	2.28E-11
6	rs476602	32569792	C	ADD	146651	0.8614	-6.676	2.46E-11
6	rs622137	32569852	A	ADD	143967	1.14	5.883	4.02E-09

6	rs116067873	32569953	T	ADD	148586	0.62	-8.083	6.33E-16
6	rs535476317	32570019	A	ADD	147409	1.142	6.067	1.30E-09
6	rs2516052	32570147	G	ADD	146940	1.142	6.024	1.70E-09
6	rs2516051	32570184	T	ADD	146354	1.142	6.046	1.49E-09
6	6:32570261_AG_A	32570261	A	ADD	146736	0.8611	-6.688	2.26E-11
6	rs34039593	32570311	G	ADD	148353	1.225	8.009	1.16E-15
6	rs2516050	32570352	A	ADD	148015	0.862	-6.677	2.44E-11
6	rs2647062	32570417	C	ADD	137250	1.228	7.144	9.06E-13
6	rs558721	32570573	T	ADD	143800	1.222	7.534	4.91E-14
6	rs9281920	32570947	TG	ADD	146794	0.8594	-6.771	1.28E-11
6	rs679242	32570958	T	ADD	143864	1.223	7.576	3.56E-14
6	rs679243	32570959	T	ADD	146669	0.8602	-6.724	1.77E-11
6	rs9270862	32570981	C	ADD	145336	0.8595	-6.743	1.55E-11
6	rs9270863	32570982	A	ADD	145336	0.8595	-6.743	1.55E-11
6	rs680061	32571098	G	ADD	143126	0.8642	-6.426	1.31E-10
6	6:32571102_CT_C	32571102	C	ADD	145055	0.8581	-6.775	1.24E-11
6	rs2760990	32571110	A	ADD	143860	1.223	7.581	3.42E-14
6	rs28383179	32571116	A	ADD	145056	1.142	6.04	1.54E-09
6	rs28383180	32571118	C	ADD	145053	1.142	6.037	1.57E-09
6	rs2647066	32571122	T	ADD	148393	1.226	8.021	1.05E-15
6	rs9270869	32571155	C	ADD	146705	0.8608	-6.702	2.05E-11
6	rs9270871	32571160	G	ADD	146706	0.8608	-6.701	2.07E-11
6	rs9270876	32571314	A	ADD	144711	0.8614	-6.604	4.00E-11
6	rs9270877	32571318	G	ADD	144708	0.8614	-6.603	4.04E-11
6	rs9270878	32571320	A	ADD	144708	0.8614	-6.603	4.04E-11
6	rs28383181	32571669	T	ADD	147461	1.142	6.057	1.39E-09
6	rs9270884	32571734	G	ADD	123881	0.8651	-6.082	1.19E-09
6	rs28383182	32571811	A	ADD	125552	1.175	6.324	2.54E-10
6	rs12211942	32571897	G	ADD	131814	1.161	6.417	1.39E-10
6	rs17425622	32571961	C	ADD	146408	1.228	8.056	7.86E-16
6	rs146910480	32572023	GC	ADD	144152	0.8015	-6.267	3.68E-10
6	rs585305	32572048	T	ADD	147164	1.142	6.068	1.30E-09
6	rs34102154	32572106	A	ADD	147905	0.8051	-6.536	6.32E-11
6	rs586610	32572311	C	ADD	136319	1.165	6.469	9.86E-11
6	rs601148	32573265	T	ADD	144607	1.224	7.79	6.71E-15
6	rs601945	32573415	G	ADD	148203	1.227	8.042	8.83E-16
6	6:32573456_TAATC_T	32573456	T	ADD	148679	0.6883	-7.146	8.90E-13

6	rs602457	32573562	C	ADD	137228	1.249	7.784	7.03E-15
6	rs34242956	32574132	A	ADD	148674	0.6883	-7.129	1.02E-12
6	rs2647074	32574360	T	ADD	148324	0.8368	-7.666	1.78E-14
6	rs113315602	32574575	C	ADD	136754	1.24	5.877	4.18E-09
6	rs515185	32574742	A	ADD	148615	1.221	7.877	3.35E-15
6	rs628754	32574787	A	ADD	148000	0.8616	-6.684	2.33E-11
6	rs7760841	32574868	T	ADD	137248	1.24	7.548	4.43E-14
6	rs7770010	32574896	A	ADD	148591	1.223	7.932	2.16E-15
6	rs7761182	32575099	T	ADD	148643	1.221	7.883	3.20E-15
6	rs113742050	32575432	T	ADD	141293	1.275	6.729	1.71E-11
6	rs4713555	32575524	T	ADD	148420	0.8603	-6.093	1.11E-09
6	rs116454087	32575602	G	ADD	148733	0.6252	-7.978	1.49E-15
6	rs3021304	32575658	C	ADD	148783	0.879	-6.042	1.52E-09
6	rs188053776	32577014	T	ADD	147177	0.6404	-7.662	1.83E-14
6	6:32577088_TC_T	32577088	T	ADD	131152	1.288	7.308	2.72E-13
6	6:32577107_TAAA_T	32577107	T	ADD	125058	0.8664	-6.199	5.70E-10
6	rs560530	32577222	A	ADD	147921	1.232	8.482	2.21E-17
6	rs660895	32577380	G	ADD	148783	1.227	8.362	6.16E-17
6	6:32577561_ATG_A	32577561	A	ADD	148474	1.228	8.412	4.02E-17
6	6:32577914_GGA_G	32577914	G	ADD	148485	1.228	8.402	4.37E-17
6	rs532965	32577973	G	ADD	148783	1.222	7.908	2.61E-15
6	rs3997868	32578590	G	ADD	148299	1.23	8.439	3.21E-17
6	rs504594	32578772	A	ADD	148467	1.223	7.915	2.47E-15
6	rs6917729	32579643	A	ADD	148344	1.218	7.766	8.08E-15
6	rs3997872	32580617	A	ADD	148479	1.223	7.921	2.36E-15
6	rs62406297	32581650	A	ADD	148747	0.6257	-7.976	1.51E-15
6	rs149839148	32581740	A	ADD	147649	1.222	7.874	3.44E-15
6	rs522308	32581922	T	ADD	148474	1.141	6.047	1.47E-09
6	rs521539	32581973	A	ADD	148783	1.227	8.378	5.41E-17
6	rs3129751	32582189	C	ADD	148475	1.224	7.937	2.08E-15
6	rs28507026	32582485	G	ADD	148612	0.8057	-6.954	3.55E-12
6	rs62406298	32582541	G	ADD	148778	0.6257	-7.977	1.50E-15
6	rs62406299	32582558	C	ADD	148778	0.6257	-7.977	1.50E-15
6	rs3104413	32582650	G	ADD	148783	1.22	7.854	4.02E-15
6	rs62406300	32582772	A	ADD	148779	0.6257	-7.977	1.50E-15
6	rs62406301	32582807	C	ADD	148779	0.6257	-7.977	1.50E-15
6	rs3129753	32583027	C	ADD	146869	1.226	7.926	2.26E-15

6	rs6605556	32583099	G	ADD	146352	1.229	7.987	1.38E-15
6	rs6941393	32583200	A	ADD	146604	1.231	8.408	4.19E-17
6	rs6941395	32583206	A	ADD	146864	1.226	7.927	2.24E-15
6	rs6931277	32583357	T	ADD	148692	1.22	7.847	4.25E-15
6	rs6941972	32583529	A	ADD	133349	1.257	7.793	6.54E-15
6	rs115625939	32583584	G	ADD	148382	0.6698	-9.462	3.02E-21
6	rs113676242	32583719	G	ADD	148782	0.6256	-7.98	1.47E-15
6	rs113468765	32583749	T	ADD	148781	0.6256	-7.979	1.48E-15
6	rs1281935	32583820	T	ADD	137923	1.295	7.229	4.88E-13
6	rs9271349	32583830	A	ADD	142329	1.167	5.82	5.88E-09
6	rs112648389	32583900	G	ADD	144874	0.6242	-6.731	1.68E-11
6	rs1281934	32584382	G	ADD	142509	1.295	6.893	5.46E-12
6	6:32584419_CTT_C	32584419	C	ADD	147060	1.141	6.028	1.66E-09
6	rs73730372	32584581	T	ADD	148748	0.6729	-9.43	4.11E-21
6	rs62406302	32584662	G	ADD	148780	0.6257	-7.978	1.49E-15
6	rs510205	32584693	G	ADD	148365	1.226	8.289	1.14E-16
6	rs28752520	32584739	C	ADD	147605	0.8368	-6.962	3.35E-12
6	rs113177970	32584749	T	ADD	148778	0.6256	-7.978	1.48E-15
6	rs111290278	32584863	A	ADD	148175	0.6225	-7.89	3.03E-15
6	rs114701564	32584875	A	ADD	147786	0.6347	-7.469	8.08E-14
6	rs73730377	32584880	G	ADD	148235	0.6693	-9.417	4.64E-21
6	rs113232664	32584901	G	ADD	146427	0.6178	-7.007	2.44E-12
6	rs28752526	32584918	G	ADD	145206	1.152	6.446	1.15E-10
6	rs145969392	32584936	GT	ADD	146877	1.153	6.553	5.64E-11
6	rs685810	32584952	G	ADD	148755	1.149	6.418	1.38E-10
6	rs113016235	32584977	G	ADD	148774	0.6748	-9.383	6.44E-21
6	rs28752534	32585068	C	ADD	131272	0.7678	-10.05	8.77E-24
6	rs113243185	32585071	C	ADD	148494	0.6708	-9.474	2.68E-21
6	rs13213821	32585199	G	ADD	138428	0.7709	-9.739	2.05E-22
6	6:32585268_TC_T	32585268	T	ADD	147407	0.6668	-9.369	7.33E-21
6	rs62406303	32585294	G	ADD	147268	0.7447	-10.5	8.71E-26
6	rs62406304	32585298	T	ADD	147886	0.6273	-7.933	2.15E-15
6	rs62406305	32585371	A	ADD	147306	0.6709	-9.45	3.40E-21
6	rs13217434	32585388	C	ADD	138495	0.7711	-9.734	2.16E-22
6	rs62406306	32585520	A	ADD	148780	0.6257	-7.978	1.49E-15
6	rs34692256	32585805	G	ADD	148780	0.6257	-7.978	1.49E-15
6	rs28383263	32585843	G	ADD	148718	0.625	-7.997	1.28E-15
6	rs11751024	32586236	A	ADD	148431	0.8162	-9.196	3.73E-20

6	rs11755689	32586376	G	ADD	130288	0.7796	-9.825	8.81E-23
6	rs62404063	32586419	A	ADD	148747	0.626	-7.97	1.59E-15
6	rs1281933	32586507	T	ADD	147725	0.6332	-7.892	2.98E-15
6	rs28383298	32586573	T	ADD	147547	0.6096	-7.959	1.73E-15
6	rs9271363	32586732	G	ADD	148779	1.18	6.217	5.08E-10
6	rs13191810	32586831	T	ADD	148717	0.8671	-6.114	9.74E-10
6	rs607929	32586998	G	ADD	147497	0.8792	-5.974	2.31E-09
6	rs28688825	32587157	G	ADD	148766	0.7479	-7.959	1.74E-15
6	rs28533694	32587235	T	ADD	148708	0.8104	-8.45	2.91E-17
6	rs9271378	32587300	G	ADD	148696	0.8836	-5.845	5.06E-09
6	rs1281932	32587350	A	ADD	142526	1.233	8.176	2.94E-16
6	rs111294540	32587359	A	ADD	148774	0.6259	-7.972	1.56E-15
6	rs190231509	32587425	T	ADD	148706	0.6225	-8.026	1.01E-15
6	rs544537666	32587494	G	ADD	148134	0.6214	-7.868	3.60E-15
6	rs36232627	32587499	C	ADD	148134	0.6214	-7.868	3.60E-15
6	rs36232626	32587500	T	ADD	148134	0.6214	-7.868	3.60E-15
6	rs36232625	32587507	T	ADD	148145	0.6214	-7.868	3.62E-15
6	rs34116693	32587603	T	ADD	148775	0.6259	-7.972	1.56E-15
6	rs36232342	32587618	C	ADD	148773	0.6261	-7.967	1.63E-15
6	rs9271409	32587628	C	ADD	147766	1.135	5.797	6.76E-09
6	rs35168703	32587652	T	ADD	148228	0.6189	-7.95	1.87E-15
6	rs35390996	32587663	A	ADD	148770	0.6262	-7.962	1.69E-15
6	rs116300229	32587808	A	ADD	144323	0.6041	-6.724	1.77E-11
6	rs1281931	32587966	C	ADD	137869	1.292	7.183	6.80E-13
6	rs80257164	32587967	A	ADD	148775	0.6261	-7.967	1.63E-15
6	rs9271429	32587985	A	ADD	148671	1.171	5.976	2.29E-09
6	rs200991817	32588104	T	ADD	144196	0.6188	-6.624	3.50E-11
6	6:32588135_GT_G	32588135	G	ADD	146401	0.6195	-7.38	1.58E-13
6	rs115076650	32588215	G	ADD	148780	0.6258	-7.973	1.55E-15
6	rs115632345	32588480	T	ADD	148683	0.6261	-7.965	1.65E-15
6	rs9271464	32588544	T	ADD	140389	1.142	5.862	4.56E-09
6	rs9271465	32588554	T	ADD	140418	1.142	5.856	4.73E-09
6	rs9271469	32588630	C	ADD	146219	1.135	5.749	8.99E-09
6	rs76815088	32588713	C	ADD	148781	0.6259	-7.972	1.56E-15
6	rs144591706	32588819	T	ADD	148725	0.6282	-7.909	2.59E-15
6	rs561763188	32588828	C	ADD	147627	0.6287	-7.607	2.80E-14
6	rs9271480	32588831	A	ADD	148735	1.133	5.737	9.62E-09
6	rs114014117	32588842	G	ADD	142280	0.6017	-5.947	2.74E-09

6	rs9271485	32588953	G<br>A<br>A<br>A	ADD	148475	1.133	5.743	9.30E-09
6	rs145443211	32588970	C	ADD	146567	1.135	5.769	7.98E-09
6	rs9271486	32588980	T	ADD	146569	1.135	5.768	8.00E-09
6	rs9271489	32589051	A	ADD	148783	0.8288	-7.969	1.60E-15
6	rs9271492	32589122	C	ADD	148756	0.8432	-7.45	9.35E-14
6	rs34645399	32589169	G	ADD	148783	0.625	-8.007	1.18E-15
6	6:32589233_GC_G	32589233	G	ADD	148779	0.6257	-7.978	1.49E-15
6	rs9271494	32589240	C	ADD	142930	1.222	7.462	8.55E-14
6	rs144081603	32589374	C	ADD	143438	0.628	-6.14	8.24E-10
6	rs9271508	32589410	T	ADD	148753	0.8279	-8.01	1.15E-15
6	rs9271509	32589415	A	ADD	145768	1.2	7.12	1.08E-12
6	rs112401673	32589416	T	ADD	148211	0.6208	-7.898	2.83E-15
6	rs114785125	32589520	G	ADD	148237	0.6192	-7.942	2.00E-15
6	rs9271513	32589523	C	ADD	148771	1.172	6.014	1.81E-09
6	rs112447098	32589544	A	ADD	148772	0.626	-7.969	1.60E-15
6	rs9271516	32589668	A	ADD	148301	1.138	5.944	2.79E-09
6	6:32589721_TAATTCTC_T	32589721	T	ADD	141928	1.151	6.307	2.84E-10
6	rs9271517	32589740	G	ADD	141929	1.151	6.307	2.85E-10
6	rs9271519	32589751	C	ADD	142440	1.145	6.063	1.34E-09
6	rs9271521	32589783	T	ADD	142624	1.146	6.08	1.21E-09
6	rs9271522	32589791	C	ADD	148759	0.8287	-7.975	1.52E-15
6	rs34135912	32589793	A	ADD	148766	0.6258	-7.973	1.55E-15
6	rs9271523	32589806	G	ADD	142803	1.145	6.082	1.19E-09
6	rs9271527	32589867	T	ADD	148624	0.8279	-8.011	1.14E-15
6	rs9271529	32589937	G	ADD	147103	1.171	5.951	2.67E-09
6	rs9271532	32589945	C	ADD	143572	1.137	5.764	8.23E-09
6	rs9271533	32589952	T	ADD	145144	0.8301	-7.845	4.32E-15
6	rs9271536	32589978	A	ADD	148744	1.172	6.008	1.88E-09
6	rs9271537	32589982	T	ADD	147154	0.8641	-6.7	2.09E-11
6	rs9271539	32590028	A	ADD	148765	0.8286	-7.979	1.48E-15
6	6:32590033_AAT_A	32590033	A	ADD	147944	0.6295	-7.886	3.13E-15
6	6:32590087_GT_G	32590087	G	ADD	148733	1.134	5.794	6.86E-09
6	rs35025478	32590162	G	ADD	148765	0.6258	-7.973	1.55E-15
6	rs35786457	32590165	A	ADD	148771	0.6257	-7.978	1.49E-15
6	rs35947801	32590187	A	ADD	148765	0.6258	-7.973	1.55E-15
6	rs9271558	32590348	G	ADD	147514	0.8311	-7.822	5.19E-15

6	rs113705304	32590453	G	ADD	148672	0.6616	-9.469	2.82E-21
6	rs9271568	32590463	A	ADD	134712	1.146	5.767	8.06E-09
6	rs2105899	32590473	T	ADD	148351	1.17	5.923	3.16E-09
6	rs9271571	32590483	T	ADD	146555	1.142	6.041	1.54E-09
6	rs2105898	32590498	T	ADD	148375	1.17	5.92	3.22E-09
6	6:32590600_AT_A	32590600	A	ADD	148754	1.133	5.763	8.27E-09
6	rs570194309	32590602	C	ADD	148754	1.133	5.763	8.27E-09
6	rs9271576	32590612	C	ADD	147303	1.135	5.775	7.71E-09
6	rs34043628	32590619	C	ADD	147581	0.6289	-7.902	2.74E-15
6	rs2395225	32590624	C	ADD	146061	0.8306	-7.824	5.11E-15
6	rs9271579	32590637	A	ADD	147250	1.212	7.622	2.49E-14
6	rs35700251	32590639	T	ADD	148709	0.624	-8.01	1.15E-15
6	rs9271580	32590641	G	ADD	148489	1.213	7.753	8.99E-15
6	rs35062987	32590735	T	ADD	148757	0.8499	-5.822	5.82E-09
6	rs34831921	32590768	A	ADD	148715	0.6606	-9.515	1.81E-21
6	rs35236701	32590778	T	ADD	148697	0.6243	-8.002	1.23E-15
6	rs2097431	32590833	A	ADD	146056	1.172	5.995	2.04E-09
6	rs2097431	32590833	T	ADD	80871	0.828	-6.382	1.75E-10
6	rs9271584	32590870	C	ADD	148756	1.133	5.733	9.89E-09
6	rs9271585	32590879	A	ADD	148756	1.133	5.733	9.89E-09
6	6:32591008_TA_T	32591008	T	ADD	148702	0.6243	-8.004	1.21E-15
6	rs9271589	32591128	A	ADD	148742	1.133	5.733	9.84E-09
6	rs9271594	32591213	G	ADD	148692	1.223	7.926	2.27E-15
6	rs35407265	32591226	G	ADD	148602	0.661	-9.489	2.34E-21
6	rs35316732	32591232	T	ADD	148602	0.661	-9.489	2.34E-21
6	rs34376179	32591238	G	ADD	144674	0.6707	-8.346	7.05E-17
6	rs3129764	32591300	G	ADD	147729	1.173	6.034	1.60E-09
6	rs9271602	32591383	G	ADD	146589	1.135	5.938	2.89E-09
6	rs34915133	32591422	G	ADD	148377	0.6603	-9.515	1.81E-21
6	rs3129765	32591428	A	ADD	148297	1.171	5.967	2.42E-09
6	rs9271604	32591430	G	ADD	148697	1.134	5.776	7.64E-09
6	rs9271605	32591448	A	ADD	148745	1.133	5.735	9.75E-09
6	rs112953812	32591500	A	ADD	148722	0.6247	-7.99	1.35E-15
6	rs9271607	32591566	G	ADD	148740	1.133	5.738	9.57E-09
6	rs73728618	32591567	A	ADD	148701	0.6607	-9.511	1.90E-21
6	rs9271608	32591588	G	ADD	148681	1.221	7.883	3.19E-15
6	rs9271609	32591597	C	ADD	148749	1.133	5.762	8.34E-09
6	rs35001273	32591664	A	ADD	148115	0.6622	-9.376	6.86E-21

6	rs9271615	32591686	G	ADD	148074	0.8054	-10.08	6.60E-24
6	rs201769738	32591687	A	ADD	148096	0.6623	-9.375	6.91E-21
6	6:32591689_GCA_G	32591689	G	ADD	148073	0.8055	-10.08	6.89E-24
6	rs556499677	32591695	G	ADD	148439	0.628	-7.796	6.40E-15
6	rs114076271	32591717	A	ADD	148725	0.6247	-7.992	1.33E-15
6	rs9271618	32591752	A	ADD	147988	0.8278	-7.984	1.42E-15
6	rs34412149	32591892	G	ADD	148707	0.6232	-8.019	1.06E-15
6	rs9271623	32591914	C	ADD	141301	0.8271	-7.903	2.72E-15
6	rs9271629	32591976	C	ADD	148363	1.171	5.962	2.49E-09
6	rs9271630	32591989	G	ADD	140996	0.8262	-7.945	1.94E-15
6	rs9271631	32591996	A	ADD	148364	1.171	5.979	2.24E-09
6	rs1281947	32592009	G	ADD	147958	1.216	7.645	2.08E-14
6	6:32592027_AATCT_A	32592027	A	ADD	148678	1.134	5.771	7.90E-09
6	rs35867827	32592068	C	ADD	148424	0.6608	-9.505	1.99E-21
6	rs9271637	32592124	G	ADD	140489	1.144	5.973	2.33E-09
6	rs532216409	32592152	G	ADD	148204	0.663	-9.319	1.17E-20
6	rs9271640	32592200	T	ADD	148526	1.17	5.955	2.60E-09
6	rs9271641	32592209	A	ADD	148469	1.171	5.982	2.21E-09
6	rs35224566	32592313	C	ADD	148413	0.6602	-9.517	1.79E-21
6	rs9271644	32592337	A	ADD	148063	1.134	5.781	7.42E-09
6	rs9271645	32592345	C	ADD	148486	1.172	6.027	1.67E-09
6	rs9271646	32592351	G	ADD	147925	0.8284	-7.955	1.79E-15
6	rs9271647	32592354	G	ADD	147931	0.8286	-7.946	1.92E-15
6	rs9271648	32592358	T	ADD	147904	0.8054	-10.08	6.49E-24
6	rs9271649	32592359	G	ADD	147931	0.8286	-7.946	1.92E-15
6	rs9271650	32592383	T	ADD	148750	1.171	5.998	2.00E-09
6	rs9271653	32592556	C	ADD	148278	0.8046	-10.14	3.82E-24
6	rs9271654	32592565	A	ADD	148032	0.8296	-7.882	3.23E-15
6	rs9271655	32592567	G	ADD	148660	1.171	6.006	1.91E-09
6	rs9271658	32592588	T	ADD	148660	1.171	6.006	1.91E-09
6	rs9271660	32592602	G	ADD	148090	1.133	5.736	9.72E-09
6	rs9271661	32592605	A	ADD	142467	1.142	5.97	2.37E-09
6	rs9271662	32592628	A	ADD	142532	0.817	-8.234	1.82E-16
6	rs9271663	32592636	A	ADD	147986	0.8046	-10.12	4.29E-24
6	rs9271664	32592656	T	ADD	148689	1.171	6.002	1.95E-09
6	rs9271667	32592710	C	ADD	147877	0.8275	-7.992	1.32E-15
6	rs9271673	32592833	C	ADD	148704	1.17	5.947	2.74E-09
6	rs9271674	32592845	C	ADD	147212	1.134	5.781	7.41E-09

6	rs9271675	32592862	C	ADD	146130	1.139	5.937	2.90E-09
6	rs9271678	32592908	C	ADD	143162	1.171	5.927	3.09E-09
6	rs9271679	32592909	A	ADD	143162	1.171	5.927	3.09E-09
6	rs9271680	32592915	G	ADD	143187	1.171	5.933	2.98E-09
6	rs9271681	32592918	T	ADD	147473	0.8332	-7.785	6.99E-15
6	rs9271682	32592923	G	ADD	144924	1.137	5.819	5.92E-09
6	rs9271683	32592950	C	ADD	148783	1.172	6.05	1.45E-09
6	rs9271684	32592953	T	ADD	112531	0.823	-7.328	2.33E-13
6	rs9271685	32592970	C	ADD	146034	1.139	5.926	3.10E-09
6	rs9271686	32592973	T	ADD	147796	1.17	5.982	2.20E-09
6	rs9271688	32592992	G	ADD	148542	0.8291	-7.931	2.17E-15
6	rs9271689	32592999	C	ADD	147798	1.17	5.982	2.21E-09
6	rs9271691	32593006	G	ADD	147521	0.8053	-10.06	8.08E-24
6	rs9271692	32593010	A	ADD	132774	0.8225	-8.585	9.10E-18
6	rs9271693	32593033	T	ADD	148475	1.134	5.765	8.19E-09
6	rs78390183	32593040	T	ADD	148445	0.6161	-8.095	5.71E-16
6	rs9271694	32593051	C	ADD	148731	0.8289	-7.944	1.96E-15
6	rs9271695	32593080	A	ADD	148687	1.171	5.994	2.05E-09
6	rs9271697	32593095	G	ADD	148753	1.17	5.956	2.59E-09
6	rs35508382	32593144	G	ADD	148629	0.6597	-9.537	1.47E-21
6	rs9271699	32593179	G	ADD	148233	0.8056	-10.07	7.40E-24
6	rs9271700	32593198	G	ADD	147738	1.171	6.014	1.81E-09
6	rs9271701	32593199	T	ADD	148599	1.171	5.994	2.05E-09
6	rs9271702	32593220	T	ADD	147761	1.171	6	1.98E-09
6	rs34720986	32593270	C	ADD	148407	0.6585	-9.516	1.80E-21
6	rs9271703	32593280	T	ADD	148647	0.829	-7.939	2.04E-15
6	rs9271704	32593317	G	ADD	144544	0.845	-7.002	2.53E-12
6	rs9271705	32593321	T	ADD	145530	0.8426	-7.148	8.83E-13
6	rs9271707	32593356	T	ADD	148316	0.804	-10.19	2.20E-24
6	rs9271708	32593386	A	ADD	119497	0.8076	-7.143	9.14E-13
6	rs9271709	32593392	A	ADD	148783	0.8285	-7.966	1.64E-15
6	rs9271711	32593430	T	ADD	141901	0.8008	-10.03	1.18E-23
6	rs9271712	32593432	A	ADD	141108	0.8106	-9.568	1.09E-21
6	rs9271714	32593434	G	ADD	141901	0.8008	-10.03	1.18E-23
6	rs9271715	32593441	T	ADD	141915	0.8008	-10.03	1.15E-23
6	rs9271716	32593442	G	ADD	141915	0.8008	-10.03	1.15E-23
6	rs9271717	32593453	G	ADD	141948	0.801	-10.02	1.23E-23
6	rs9271718	32593465	T	ADD	142415	1.135	5.76	8.41E-09

6	rs9271719	32593479	C	ADD	148671	1.17	5.959	2.53E-09
6	rs9271720	32593507	A	ADD	148783	0.8051	-10.13	4.22E-24
6	rs9271721	32593519	G	ADD	148675	1.17	5.964	2.47E-09
6	rs9271722	32593522	G	ADD	148635	1.135	5.843	5.13E-09
6	rs9271724	32593552	C	ADD	148215	0.8305	-7.844	4.36E-15
6	rs9271726	32593566	G	ADD	148604	1.21	7.689	1.48E-14
6	rs9271728	32593592	C	ADD	147250	0.8336	-7.647	2.06E-14
6	rs9271729	32593600	A	ADD	147241	0.8337	-7.643	2.13E-14
6	rs562122363	32593617	C	ADD	144320	0.6306	-6.445	1.16E-10
6	rs9271733	32593633	A	ADD	144383	0.8469	-6.893	5.46E-12
6	rs9271738	32593691	G	ADD	144480	0.8453	-6.98	2.96E-12
6	rs9271739	32593692	T	ADD	138756	0.8152	-9.153	5.55E-20
6	rs28362368	32594040	G	ADD	148335	0.8294	-7.91	2.58E-15
6	rs72848204	32594073	T	ADD	148209	0.8286	-7.951	1.86E-15
6	rs34518860	32594103	A	ADD	148387	0.657	-9.552	1.27E-21
6	6:32594132_AG_A	32594132	A	ADD	147308	0.6197	-7.734	1.04E-14
6	rs9271764	32594159	A	ADD	148526	1.133	5.736	9.72E-09
6	rs9271765	32594166	G	ADD	148526	1.133	5.736	9.72E-09
6	rs9271766	32594169	A	ADD	148730	1.17	5.952	2.66E-09
6	rs9271767	32594176	T	ADD	148526	1.133	5.736	9.72E-09
6	rs35130701	32594199	G	ADD	148635	0.8295	-7.915	2.48E-15
6	rs9271769	32594217	T	ADD	147014	1.135	5.784	7.31E-09
6	rs9271771	32594274	T	ADD	148783	1.171	5.986	2.15E-09
6	rs9271772	32594275	G	ADD	148778	1.17	5.976	2.29E-09
6	rs75237145	32594295	T	ADD	144748	0.802	-10.15	3.46E-24
6	rs114969562	32594299	A	ADD	133075	0.8606	-5.738	9.61E-09
6	rs9271774	32594309	A	ADD	148746	1.171	5.989	2.11E-09
6	rs568805507	32594388	T	ADD	142030	0.6807	-7.286	3.21E-13
6	rs535840294	32594394	G	ADD	142006	0.6818	-7.255	4.01E-13
6	rs116093550	32594395	G	ADD	145322	0.817	-9.341	9.56E-21
6	rs116361489	32594396	T	ADD	145322	0.817	-9.341	9.56E-21
6	rs111634228	32594452	G	ADD	146359	0.8096	-9.778	1.39E-22
6	rs114309058	32594470	A	ADD	83482	0.7789	-6.23	4.66E-10
6	rs9271780	32594535	C	ADD	137727	0.8145	-9.181	4.29E-20
6	rs9271781	32594547	A	ADD	136676	0.8152	-9.106	8.55E-20
6	rs9271782	32594562	A	ADD	131111	0.8097	-9.166	4.91E-20
6	rs9271783	32594579	A	ADD	117366	0.8331	-7.371	1.69E-13
6	6:32594650_CA_C	32594650	C	ADD	127419	0.813	-8.787	1.54E-18

6	rs9271784	32594704	A	ADD	111549	0.8448	-6.608	3.89E-11
6	rs9271786	32594709	T	ADD	130441	0.8127	-8.949	3.59E-19
6	rs9271787	32594718	C	ADD	137814	0.8092	-9.456	3.21E-21
6	rs9271788	32594734	G	ADD	145346	0.7992	-10.31	6.05E-25
6	rs9271789	32594768	A	ADD	145006	0.8005	-10.23	1.51E-24
6	rs9271791	32594810	A	ADD	144997	0.8006	-10.22	1.59E-24
6	rs147599819	32594816	GGAT	ADD	110595	0.8454	-6.6	4.12E-11
6	rs9281963	32594818	AT	ADD	137810	1.221	7.784	7.02E-15
6	rs200191577	32594819	GAT	ADD	137810	1.221	7.784	7.02E-15
6	rs9271794	32594855	A	ADD	147602	0.8049	-10.09	6.39E-24
6	rs72848212	32594898	T	ADD	148381	0.8037	-10.18	2.40E-24
6	rs9271823	32594915	G	ADD	148462	0.8037	-10.18	2.36E-24
6	rs9271824	32594916	G	ADD	148783	0.805	-10.12	4.57E-24
6	rs9271842	32594953	A	ADD	106282	0.8205	-7.426	1.12E-13
6	rs9271843	32594957	T	ADD	143357	0.8015	-10.06	8.04E-24
6	rs3129767	32594975	G	ADD	147143	1.173	5.808	6.31E-09
6	rs9271846	32594980	A	ADD	138573	0.8064	-9.614	6.96E-22
6	6:32594986_TA_T	32594986	T	ADD	138570	0.8064	-9.616	6.86E-22
6	rs9271847	32594993	G	ADD	138635	0.8062	-9.63	5.98E-22
6	rs116518618	32594998	T	ADD	137070	0.6715	-6.026	1.68E-09
6	rs9271848	32595027	G	ADD	146286	0.8045	-10.06	8.41E-24
6	rs3104390	32595031	A	ADD	148756	1.17	5.964	2.47E-09
6	rs9271850	32595060	G	ADD	146139	0.8244	-8.052	8.18E-16
6	rs3129768	32595083	G	ADD	148783	1.165	5.803	6.50E-09
6	rs9271854	32595118	A	ADD	148515	0.8049	-10.12	4.55E-24
6	rs9271855	32595119	C	ADD	148515	0.8049	-10.12	4.55E-24
6	rs9271856	32595120	A	ADD	148515	0.8049	-10.12	4.55E-24
6	rs9271857	32595147	T	ADD	137390	0.8203	-8.898	5.67E-19
6	rs116603449	32595194	T	ADD	83719	0.7686	-7.074	1.50E-12
6	rs9271858	32595223	G	ADD	148552	0.8045	-10.15	3.46E-24
6	rs9271859	32595231	G	ADD	148171	0.8065	-10.02	1.24E-23
6	rs9271861	32595287	T	ADD	139059	0.8019	-9.903	4.06E-23
6	rs9271862	32595333	C	ADD	125918	0.8242	-8.223	1.99E-16
6	rs9271863	32595334	A	ADD	129104	0.8115	-8.984	2.62E-19
6	rs6921651	32595345	A	ADD	131046	0.8256	-8.441	3.15E-17
6	rs9271865	32595360	T	ADD	130703	0.8132	-8.942	3.84E-19

6	rs9271866	32595367	T	ADD	130698	0.8132	-8.939	3.91E-19
6	rs9271867	32595368	G	ADD	130698	0.8132	-8.939	3.91E-19
6	rs9271868	32595375	C	ADD	130227	0.8078	-9.039	1.58E-19
6	rs9271869	32595376	A	ADD	130225	0.8078	-9.039	1.59E-19
6	rs9271870	32595377	T	ADD	130225	0.8078	-9.039	1.59E-19
6	rs9271871	32595383	C	ADD	144631	0.8342	-7.491	6.85E-14
6	rs9271872	32595418	G	ADD	147815	0.8318	-7.773	7.69E-15
6	rs9271873	32595421	A	ADD	147816	0.8318	-7.773	7.68E-15
6	rs6926251	32595429	T	ADD	147510	1.17	5.979	2.25E-09
6	rs9271876	32595448	G	ADD	141615	0.8088	-9.656	4.65E-22
6	rs9271877	32595469	C	ADD	141965	0.8088	-9.673	3.92E-22
6	rs9271878	32595474	C	ADD	141993	0.8086	-9.681	3.63E-22
6	rs9271879	32595489	T	ADD	142179	0.8085	-9.695	3.17E-22
6	rs9271880	32595596	C	ADD	147096	0.8348	-7.57	3.74E-14
6	rs9271881	32595604	A	ADD	138013	0.8106	-9.39	6.01E-21
6	rs9271882	32595605	T	ADD	138006	0.8105	-9.399	5.51E-21
6	rs9271883	32595610	A	ADD	138040	0.8111	-9.365	7.57E-21
6	rs9271884	32595628	C	ADD	137693	0.8133	-9.246	2.32E-20
6	rs9271885	32595629	T	ADD	137693	0.8133	-9.246	2.32E-20
6	rs9271886	32595633	T	ADD	137693	0.8133	-9.246	2.32E-20
6	rs9271887	32595679	A	ADD	148638	0.8291	-7.935	2.10E-15
6	rs9271888	32595732	C	ADD	148558	0.8058	-10.07	7.38E-24
6	rs9271889	32595747	A	ADD	148714	0.8055	-10.1	5.67E-24
6	rs9271890	32595779	A	ADD	148611	0.8057	-10.08	6.78E-24
6	rs9271891	32595783	T	ADD	148700	0.8055	-10.1	5.60E-24
6	rs9271892	32595813	C	ADD	148679	0.8058	-10.08	6.93E-24
6	rs35879651	32595857	T	ADD	148464	0.6225	-7.973	1.55E-15
6	rs9271893	32595859	T	ADD	148650	0.8046	-10.15	3.41E-24
6	rs9271894	32595874	T	ADD	148783	0.8295	-7.913	2.51E-15
6	rs9271896	32595907	A	ADD	148634	0.8046	-10.14	3.58E-24
6	rs9271897	32595954	A	ADD	148598	0.8048	-10.13	4.06E-24
6	rs9271898	32595972	A	ADD	148646	0.8053	-10.1	5.42E-24
6	rs9271899	32596007	G	ADD	148395	0.8066	-10.02	1.20E-23
6	rs9271900	32596013	G	ADD	148528	0.8303	-7.872	3.50E-15
6	rs9271901	32596027	T	ADD	148398	0.8066	-10.02	1.19E-23
6	rs9271906	32596048	A	ADD	148399	0.8065	-10.02	1.19E-23
6	6:32596074_CAG_C	32596074	C	ADD	148215	0.8051	-10.12	4.40E-24
6	rs9271907	32596123	T	ADD	147017	0.8108	-9.734	2.16E-22

6	rs9271908	32596131	T A C T T A C A	ADD	147017	0.8108	-9.734	2.16E-22
6	rs148565413	32596159	G	ADD	144804	0.8156	-9.395	5.73E-21
6	rs9271909	32596177	T	ADD	144804	0.8156	-9.395	5.73E-21
6	rs9271910	32596183	C	ADD	144819	0.8154	-9.406	5.16E-21
6	rs2133036	32596203	T	ADD	146935	1.206	7.345	2.06E-13
6	rs9271912	32596207	T	ADD	145881	0.8128	-9.583	9.43E-22
6	rs9271913	32596222	A	ADD	145880	0.8128	-9.583	9.43E-22
6	6:32596232_GA_G	32596232	G	ADD	148747	0.8054	-10.1	5.43E-24
6	rs9271914	32596238	C	ADD	145882	0.8128	-9.584	9.34E-22
6	rs9271915	32596294	C	ADD	147305	0.8083	-9.888	4.69E-23
6	rs2036202	32596309	C	ADD	148519	1.168	5.876	4.19E-09
6	rs9271917	32596336	G	ADD	148062	0.8092	-9.87	5.63E-23
6	rs9271918	32596352	T	ADD	148464	0.8071	-9.998	1.55E-23
6	rs9271919	32596423	G	ADD	148608	0.8046	-10.14	3.66E-24
6	rs202092176	32596429	T	ADD	139981	0.6776	-6.704	2.03E-11
6	rs9271920	32596430	G	ADD	126066	0.7993	-9.453	3.28E-21
6	rs9271921	32596431	G	ADD	126066	0.7993	-9.453	3.28E-21
6	rs9271922	32596444	A	ADD	141289	0.8043	-9.842	7.44E-23
6	rs116489113	32596496	G	ADD	148656	0.6236	-8.006	1.18E-15
6	rs9271924	32596498	A	ADD	91943	0.7636	-8.249	1.59E-16
6	rs9271925	32596540	G	ADD	89952	0.7774	-7.445	9.73E-14
6	rs9271926	32596566	T	ADD	89950	0.7774	-7.445	9.71E-14
6	rs9271927	32596647	C	ADD	146399	0.8022	-10.18	2.49E-24
6	rs9271928	32596648	A	ADD	142721	0.8064	-9.807	1.05E-22
6	rs9271929	32596650	C	ADD	142717	0.8026	-9.98	1.86E-23
6	6:32596661_TG_T	32596661	T	ADD	141594	0.8042	-9.897	4.27E-23
6	rs9271930	32596677	G	ADD	145618	0.8046	-10.05	9.63E-24
6	rs9271932	32596714	C	ADD	147188	0.8049	-10.09	5.83E-24
6	rs9271933	32596717	A	ADD	147188	0.8049	-10.09	5.83E-24
6	rs9271934	32596773	T	ADD	148749	0.805	-10.12	4.34E-24
6	6:32596798_AG_A	32596798	A	ADD	148665	0.66	-9.526	1.63E-21
6	6:32596817_TTC_T	32596817	T	ADD	146896	0.8039	-10.14	3.70E-24
6	rs9271935	32596831	C	ADD	146896	0.8039	-10.14	3.77E-24

6	6:32596838_ATTG_A	32596838	A	ADD	148677	1.133	5.757	8.55E-09
6	rs73728889	32596856	G	ADD	148651	0.6605	-9.51	1.90E-21
6	rs9271937	32596870	G	ADD	145595	0.8082	-9.845	7.17E-23
6	rs541843223	32596877	T	ADD	148167	0.6217	-7.919	2.39E-15
6	rs116098252	32596890	C	ADD	146653	0.6249	-7.272	3.55E-13
6	rs9271939	32596921	C	ADD	146092	0.8084	-9.85	6.87E-23
6	rs6928873	32596946	A	ADD	147378	0.8282	-7.96	1.72E-15
6	rs115826298	32596985	A	ADD	148622	0.6233	-8	1.25E-15
6	rs6929057	32597005	C	ADD	146019	0.7814	-9.391	5.94E-21
6	rs3129769	32597022	A	ADD	147088	1.229	8.049	8.37E-16
6	rs9271945	32597034	A	ADD	148685	0.8043	-10.16	2.88E-24
6	6:32597087_CA_C	32597087	C	ADD	148684	1.149	6.428	1.30E-10
6	rs9271947	32597095	T	ADD	117771	0.784	-9.567	1.10E-21
6	6:32597096_AAC_A	32597096	A	ADD	148736	0.8057	-10.08	6.52E-24
6	rs9271948	32597105	A	ADD	145458	0.8172	-9.328	1.08E-20
6	rs9271951	32597172	A	ADD	145745	0.812	-9.637	5.58E-22
6	rs9271954	32597216	C	ADD	147531	0.806	-10.03	1.12E-23
6	rs9271955	32597281	A	ADD	148582	0.8063	-10.05	9.12E-24
6	rs9271957	32597335	C	ADD	148582	0.8063	-10.05	9.33E-24
6	rs9271959	32597377	C	ADD	146737	0.8105	-9.744	1.95E-22
6	rs1281944	32597379	C	ADD	134496	1.245	6.721	1.80E-11
6	rs9271960	32597385	T	ADD	146735	0.8105	-9.744	1.96E-22
6	rs9271961	32597412	A	ADD	139640	0.8086	-9.572	1.05E-21
6	rs9271962	32597434	C	ADD	140411	0.8024	-9.951	2.50E-23
6	rs9271963	32597453	A	ADD	138272	0.8021	-9.827	8.63E-23
6	rs9271964	32597464	C	ADD	134978	0.8049	-9.547	1.34E-21
6	rs9271966	32597507	T	ADD	134172	0.8048	-9.55	1.30E-21
6	rs9271968	32597529	G	ADD	133214	0.8104	-9.237	2.53E-20
6	rs189302941	32597537	A	ADD	88736	0.7156	-6.657	2.79E-11
6	rs9271970	32597550	A	ADD	133636	0.8145	-9.04	1.57E-19
6	rs9271972	32597567	T	ADD	133179	0.8488	-7.098	1.27E-12
6	rs543085682	32597598	T	ADD	146474	0.6396	-6.995	2.66E-12
6	rs9271976	32597617	C	ADD	133984	0.8071	-9.454	3.27E-21
6	rs9271977	32597623	A	ADD	134048	0.8073	-9.448	3.45E-21
6	rs9271978	32597632	C	ADD	128656	0.8097	-9.059	1.31E-19
6	rs9271979	32597635	C	ADD	128225	0.8454	-7.065	1.60E-12
6	rs9271981	32597639	C	ADD	128527	0.8106	-9.011	2.04E-19
6	rs9271983	32597647	T	ADD	129371	0.8133	-8.921	4.64E-19

6	rs9271984	32597669	G	ADD	128008	0.8173	-8.655	4.95E-18
6	rs9271985	32597679	T	ADD	128022	0.8172	-8.66	4.74E-18
6	rs9271986	32597700	A	ADD	128215	0.8169	-8.668	4.41E-18
6	rs9271987	32597716	G	ADD	129216	0.8169	-8.664	4.57E-18
6	rs9271988	32597725	A	ADD	129244	0.8172	-8.648	5.22E-18
6	rs9271989	32597729	T	ADD	129256	0.8171	-8.654	4.99E-18
6	rs9271990	32597735	T	ADD	129473	0.8159	-8.723	2.71E-18
6	rs9271991	32597738	G	ADD	129473	0.8159	-8.723	2.70E-18
6	rs9271995	32597777	A	ADD	131134	0.8146	-8.88	6.70E-19
6	rs9271997	32597801	A	ADD	146901	0.836	-7.614	2.66E-14
6	rs2395518	32597812	C	ADD	147262	1.215	7.604	2.87E-14
6	6:32597821_TG_T	32597821	T	ADD	145735	0.8088	-9.818	9.40E-23
6	rs9271999	32597832	G	ADD	145910	0.8089	-9.811	1.01E-22
6	rs9272000	32597834	T	ADD	145910	0.8089	-9.811	1.01E-22
6	rs9272001	32597901	G	ADD	145361	0.807	-9.891	4.57E-23
6	rs9272002	32597924	G	ADD	144790	0.8411	-7.873	3.46E-15
6	rs9272003	32597940	G	ADD	144788	0.8411	-7.874	3.43E-15
6	rs9272004	32597964	A	ADD	140410	0.8042	-9.842	7.44E-23
6	rs9272005	32597992	G	ADD	140345	0.8035	-9.874	5.42E-23
6	rs9272006	32597993	T	ADD	140345	0.8035	-9.874	5.42E-23
6	rs9272007	32598038	T	ADD	147570	0.8047	-10.1	5.52E-24
6	rs9272008	32598042	A	ADD	147516	0.8049	-10.09	6.07E-24
6	rs9272009	32598063	T	ADD	147899	0.8051	-10.09	5.97E-24
6	rs2395519	32598067	C	ADD	147994	1.223	7.921	2.35E-15
6	rs1281943	32598090	C	ADD	141402	1.286	6.865	6.67E-12
6	rs9272012	32598156	A	ADD	134372	0.8202	-8.734	2.46E-18
6	rs376422579	32598161	GTATC	ADD	134055	0.8203	-8.718	2.83E-18
6	rs139089699	32598180	A	ADD	147241	0.6208	-7.649	2.02E-14
6	rs9272013	32598224	T	ADD	148521	0.8066	-10.03	1.17E-23
6	rs11970000	32598291	G	ADD	148147	0.7698	-9.796	1.18E-22
6	rs9272015	32598299	A	ADD	147795	0.806	-10.04	1.02E-23
6	6:32598314_ATTTG_A	32598314	A	ADD	147758	0.8062	-10.03	1.17E-23
6	rs9272016	32598346	T	ADD	147961	0.8059	-10.05	9.37E-24
6	rs115527034	32598356	C	ADD	148614	0.6234	-7.997	1.27E-15
6	rs112313206	32598405	T	ADD	144194	0.8155	-9.383	6.43E-21
6	rs7771138	32598419	A	ADD	140205	0.7673	-9.282	1.66E-20

6	rs7770879	32598423	A	ADD	140205	0.7673	-9.282	1.66E-20
6	6:32598432_TGCAGAA_T	32598432	T	ADD	139920	0.8127	-9.369	7.30E-21
6	rs148437409	32598479	C	ADD	139914	0.8125	-9.38	6.59E-21
6	rs558095162	32598504	T	ADD	143259	0.8084	-9.746	1.93E-22
6	rs9272021	32598515	G	ADD	145433	1.211	7.679	1.61E-14
6	rs9272022	32598529	G	ADD	144603	0.8066	-9.891	4.56E-23
6	rs9272023	32598558	G	ADD	145141	0.8199	-9.226	2.80E-20
6	rs9272025	32598581	A	ADD	148362	1.135	5.812	6.17E-09
6	rs9272026	32598618	G	ADD	148293	0.8048	-10.12	4.35E-24
6	rs9272027	32598622	G	ADD	148292	0.8048	-10.12	4.37E-24
6	rs151002495	32598680	A	ADD	148636	0.6225	-8.021	1.05E-15
6	rs9272028	32598706	T	ADD	148194	0.8052	-10.09	5.86E-24
6	rs9272029	32598707	G	ADD	148194	0.8052	-10.09	5.86E-24
6	6:32598754_CTGG_C	32598754	C	ADD	140395	0.8	-8.66	4.70E-18
6	rs114354029	32598764	G	ADD	146181	0.8065	-9.965	2.17E-23
6	rs9272031	32598779	C	ADD	146203	0.8065	-9.965	2.18E-23
6	rs9272032	32598782	G	ADD	146145	0.806	-9.992	1.65E-23
6	rs7758269	32598812	C	ADD	147082	0.848	-7.196	6.21E-13
6	rs9272035	32598814	T	ADD	146179	0.8081	-9.875	5.35E-23
6	rs9272036	32598841	A	ADD	147532	0.8212	-9.232	2.66E-20
6	rs9272037	32598844	C	ADD	147452	0.8077	-9.929	3.10E-23
6	rs3104381	32598854	A	ADD	148670	1.22	7.835	4.70E-15
6	rs9272040	32598903	G	ADD	148748	0.8189	-9.389	6.06E-21
6	rs9272041	32598906	A	ADD	148748	0.8189	-9.389	6.06E-21
6	rs9272042	32598917	C	ADD	148748	0.8189	-9.389	6.06E-21
6	rs9272043	32598946	G	ADD	148753	0.8189	-9.389	6.05E-21
6	rs9272044	32598971	A	ADD	148756	0.8054	-10.1	5.40E-24
6	rs9272045	32599006	T	ADD	148652	0.8193	-9.365	7.60E-21
6	rs9272049	32599045	G	ADD	148645	0.8059	-10.07	7.45E-24
6	rs9272050	32599071	G	ADD	148680	1.265	11.04	2.51E-28
6	rs3104378	32599163	T	ADD	148158	1.209	7.497	6.54E-14
6	rs9272054	32599164	A	ADD	147281	0.809	-9.834	8.05E-23
6	rs9272055	32599176	A	ADD	146590	0.8073	-9.908	3.84E-23
6	rs9272056	32599180	A	ADD	146617	0.8208	-9.21	3.27E-20
6	rs9272057	32599190	A	ADD	146622	0.8208	-9.212	3.19E-20
6	rs9272059	32599223	C	ADD	148652	0.804	-10.17	2.73E-24
6	rs9272061	32599250	A	ADD	148622	0.8059	-10.07	7.44E-24
6	rs9272062	32599265	A	ADD	148647	0.8196	-9.346	9.10E-21

6	rs35779452	32599272	G	ADD	148608	0.7699	-9.834	8.08E-23
6	rs34061722	32599337	T	ADD	148609	0.7699	-9.831	8.25E-23
6	rs9272064	32599338	T	ADD	148751	0.8054	-10.1	5.29E-24
6	rs9272067	32599401	A	ADD	148687	0.8044	-10.15	3.20E-24
6	rs35360625	32599435	C	ADD	147743	0.7707	-9.781	1.35E-22
6	6:32599462_AT_A	32599462	A	ADD	148140	0.6222	-7.904	2.71E-15
6	rs9272070	32599485	G	ADD	148738	0.8188	-9.392	5.88E-21
6	rs9272071	32599487	C	ADD	148002	0.8394	-8.068	7.12E-16
6	rs540002313	32599555	A	ADD	140473	0.7778	-8.85	8.77E-19
6	rs9272072	32599563	G	ADD	139338	0.8126	-9.369	7.34E-21
6	rs9272073	32599570	T	ADD	139319	0.8126	-9.369	7.30E-21
6	rs9272074	32599571	G	ADD	139603	0.8244	-8.799	1.38E-18
6	rs9272075	32599593	G	ADD	136753	0.8174	-9.015	1.97E-19
6	rs147857322	32599601	A	ADD	138025	0.8123	-7.596	3.06E-14
6	rs9272076	32599624	T	ADD	135117	0.8218	-8.711	3.02E-18
6	rs9272077	32599625	G	ADD	135117	0.8218	-8.711	3.02E-18
6	rs9272079	32599632	T	ADD	133820	0.821	-8.46	2.66E-17
6	rs9272080	32599633	G	ADD	133820	0.821	-8.46	2.66E-17
6	rs9272081	32599655	C	ADD	133558	0.8236	-8.627	6.31E-18
6	rs9272084	32599680	T	ADD	134688	0.8039	-9.624	6.31E-22
6	rs9272085	32599685	C	ADD	135409	0.8149	-9.145	5.98E-20
6	rs9272086	32599688	T	ADD	134694	0.8038	-9.628	6.06E-22
6	rs550053442	32599693	A	ADD	137180	0.7515	-9.656	4.65E-22
6	rs9272090	32599736	C	ADD	139020	0.8179	-9.131	6.76E-20
6	rs9272091	32599745	C	ADD	139018	0.818	-9.13	6.85E-20
6	rs9272092	32599756	C	ADD	133153	0.8081	-9.334	1.02E-20
6	rs9272093	32599777	T	ADD	130750	0.8104	-9.068	1.22E-19
6	rs9272094	32599781	C	ADD	129273	0.8154	-8.882	6.56E-19
6	rs9272095	32599782	A	ADD	128675	0.8093	-9.038	1.59E-19
6	rs9272096	32599784	C	ADD	129272	0.8154	-8.882	6.56E-19
6	rs9272097	32599786	T	ADD	128714	0.809	-9.056	1.35E-19
6	rs9272098	32599787	G	ADD	128714	0.809	-9.056	1.35E-19
6	rs9272099	32599813	G	ADD	140835	0.8025	-9.966	2.14E-23
6	rs9272102	32599830	C	ADD	141118	0.8019	-10.01	1.40E-23
6	rs11751908	32599862	G	ADD	144149	0.7693	-9.502	2.06E-21
6	rs9272103	32599896	T	ADD	148777	0.8053	-10.11	5.07E-24
6	rs9272104	32599918	C	ADD	144292	0.8178	-9.309	1.29E-20
6	rs142794334	32599945	TA	ADD	145696	0.7996	-10.31	6.31E-25

			A<br>C<br>A					
6	rs9272105	32599999	A	ADD	148783	0.8187	-9.408	5.04E-21
6	rs35585151	32600046	A	ADD	148625	0.8019	-8.684	3.81E-18
6	rs35242582	32600057	G	ADD	148783	0.771	-9.792	1.21E-22
6	rs34180045	32600066	G	ADD	147334	0.7753	-9.499	2.12E-21
6	rs34107231	32600074	G	ADD	147150	0.7753	-9.482	2.49E-21
6	rs35635298	32600079	C	ADD	147148	0.7753	-9.48	2.54E-21
6	rs35744886	32600084	T	ADD	147149	0.7753	-9.48	2.53E-21
6	rs35828545	32600099	C	ADD	147183	0.7759	-9.459	3.10E-21
6	rs3104375	32600101	C	ADD	148736	1.22	7.828	4.96E-15
6	rs35426875	32600135	C	ADD	147318	0.7765	-9.448	3.46E-21
6	rs35656734	32600153	T	ADD	148626	0.7997	-8.785	1.56E-18
6	6:32600200_CTG_C	32600200	C	ADD	145061	0.7991	-7.629	2.37E-14
6	rs34927613	32600211	A	ADD	146149	0.7723	-9.52	1.74E-21
6	rs9272108	32600228	C	ADD	148740	0.8055	-10.1	5.76E-24
6	rs13211123	32600249	C	ADD	147363	0.7695	-9.731	2.21E-22
6	rs9272109	32600253	T	ADD	146648	0.8107	-9.731	2.23E-22
6	rs9272110	32600287	C	ADD	147429	0.805	-10.08	7.01E-24
6	rs9272113	32600291	C	ADD	147429	0.805	-10.08	7.01E-24
6	rs9272114	32600317	C	ADD	148723	0.8191	-9.379	6.66E-21
6	rs9272116	32600404	C	ADD	148783	0.8196	-9.346	9.12E-21
6	rs9272117	32600411	T	ADD	148737	0.8057	-10.08	6.51E-24
6	rs9272118	32600448	T	ADD	148734	0.8192	-9.369	7.33E-21
6	rs9272119	32600470	C	ADD	148079	0.8087	-9.891	4.58E-23
6	rs115883274	32600473	C	ADD	148217	0.6199	-7.967	1.63E-15
6	6:32600482_GT_G	32600482	G	ADD	148079	0.8087	-9.89	4.60E-23
6	rs9272120	32600501	G	ADD	148729	0.8055	-10.1	5.72E-24
6	rs13211628	32600548	G	ADD	146040	0.803	-7.606	2.84E-14
6	rs9272121	32600556	C	ADD	146161	0.8107	-9.714	2.62E-22
6	rs9272122	32600562	C	ADD	146840	1.262	10.9	1.22E-27
6	rs9272124	32600581	A	ADD	144503	0.8148	-9.421	4.48E-21
6	rs9272125	32600584	G	ADD	144508	0.8278	-8.761	1.94E-18
6	rs9272126	32600589	C	ADD	144510	0.8278	-8.761	1.94E-18
6	rs9272127	32600595	C	ADD	144475	0.8274	-8.78	1.64E-18
6	rs9272128	32600601	A	ADD	145702	0.8129	-9.568	1.09E-21
6	rs9272129	32600602	T	ADD	143243	0.8103	-9.624	6.35E-22
6	rs148418496	32600607	A	ADD	146480	0.6252	-7.207	5.72E-13

6	rs9272130	32600638	G	ADD	148737	0.8056	-10.09	6.12E-24
6	rs9272132	32600677	G	ADD	148700	0.8196	-9.352	8.60E-21
6	rs9272134	32600705	G	ADD	147568	0.8052	-10.07	7.37E-24
6	rs9272135	32600723	A	ADD	144475	0.8054	-9.947	2.59E-23
6	rs9272136	32600730	T	ADD	144475	0.8054	-9.947	2.59E-23
6	rs9272137	32600738	C	ADD	144474	0.8054	-9.947	2.59E-23
6	rs9272138	32600757	A	ADD	144674	0.8055	-9.946	2.63E-23
6	rs9272141	32600797	A	ADD	140554	0.8063	-9.753	1.79E-22
6	rs9272142	32600800	C	ADD	141796	0.8179	-9.226	2.81E-20
6	rs9272143	32600803	C	ADD	147574	0.8046	-10.12	4.70E-24
6	rs9272146	32600914	C	ADD	148700	0.8052	-10.11	4.97E-24
6	rs9272147	32600976	G	ADD	143422	0.8326	-8.472	2.41E-17
6	rs9272148	32600980	C	ADD	143414	0.8202	-9.096	9.33E-20
6	rs3104372	32600983	T	ADD	145016	1.189	6.95	3.66E-12
6	rs9272150	32601000	A	ADD	131560	0.8136	-8.977	2.78E-19
6	rs9272151	32601005	G	ADD	131559	0.8137	-8.973	2.89E-19
6	rs9272152	32601007	A	ADD	131562	0.8137	-8.973	2.88E-19
6	rs9272153	32601018	C	ADD	135438	0.8137	-9.15	5.72E-20
6	rs9272154	32601019	A	ADD	130633	0.8149	-8.408	4.15E-17
6	rs9272155	32601043	C	ADD	140428	0.8274	-8.695	3.46E-18
6	rs9272156	32601081	C	ADD	142264	0.8162	-9.266	1.92E-20
6	rs1826962	32601083	C	ADD	143659	1.196	7.135	9.71E-13
6	rs9272158	32601122	A	ADD	137366	0.816	-9.11	8.22E-20
6	rs9272160	32601130	A	ADD	134812	0.826	-8.595	8.36E-18
6	rs9272161	32601139	G	ADD	131856	0.8207	-8.644	5.41E-18
6	rs9272162	32601140	A	ADD	131856	0.8207	-8.644	5.41E-18
6	rs9272163	32601146	T	ADD	131856	0.8206	-8.645	5.36E-18
6	rs13215699	32601153	A	ADD	126240	0.7934	-6.776	1.24E-11
6	rs9272164	32601154	C	ADD	131853	0.8261	-8.471	2.43E-17
6	rs9272165	32601170	C	ADD	133533	0.8165	-8.917	4.77E-19
6	rs9272166	32601209	C	ADD	143935	0.8169	-9.286	1.61E-20
6	rs9272167	32601212	A	ADD	142052	0.8295	-8.631	6.07E-18
6	rs9272170	32601243	G	ADD	141807	0.8298	-8.609	7.39E-18
6	rs9272171	32601249	G	ADD	143690	0.8176	-9.238	2.52E-20
6	rs9272172	32601260	C	ADD	141807	0.8298	-8.608	7.42E-18
6	rs114651315	32601278	T	ADD	140023	0.825	-6.517	7.19E-11
6	rs72848251	32601288	A	ADD	137930	0.8154	-6.889	5.64E-12
6	rs9272174	32601307	T	ADD	143913	0.8176	-9.251	2.23E-20

6	rs2002777	32601332	G	ADD	142494	1.235	9.723	2.40E-22
6	rs72848254	32601375	G	ADD	140376	0.8265	-6.478	9.30E-11
6	rs73728598	32601447	G	ADD	141414	0.8277	-6.513	7.36E-11
6	rs9272179	32601451	C	ADD	143025	0.8303	-8.623	6.50E-18
6	rs9272180	32601461	T	ADD	143031	0.8302	-8.626	6.34E-18
6	rs72848256	32601531	G	ADD	140647	0.8079	-7.401	1.36E-13
6	rs9272182	32601564	A	ADD	143121	0.8298	-8.661	4.66E-18
6	rs9272183	32601604	T	ADD	142556	0.8322	-8.514	1.69E-17
6	rs9272185	32601620	A	ADD	142556	0.8319	-8.53	1.46E-17
6	rs9272186	32601635	T	ADD	142548	0.832	-8.527	1.51E-17
6	rs114803358	32601708	A	ADD	144437	0.7846	-8.779	1.65E-18
6	rs115566240	32601718	T	ADD	141161	0.8064	-7.97	1.59E-15
6	rs114567049	32601719	A	ADD	143328	0.7836	-8.711	3.02E-18
6	rs9272193	32601785	G	ADD	143232	0.8161	-9.375	6.94E-21
6	rs72848261	32601790	C	ADD	141041	0.7653	-9.269	1.88E-20
6	rs114020256	32601797	A	ADD	138200	0.7724	-9.081	1.07E-19
6	rs9272194	32601825	G	ADD	137805	0.8176	-9.098	9.23E-20
6	rs9272195	32601830	G	ADD	139173	0.8116	-9.38	6.57E-21
6	rs112216801	32601838	A	ADD	136216	0.7807	-8.227	1.92E-16
6	rs151005270	32601874	T	ADD	136934	0.7895	-7.906	2.66E-15
6	rs9272201	32601875	G	ADD	137284	0.8223	-8.838	9.76E-19
6	rs113861133	32601911	C	ADD	134040	0.7824	-7.892	2.98E-15
6	rs116630455	32601946	C	ADD	137013	0.7686	-8.747	2.19E-18
6	rs9272207	32601966	C	ADD	136099	0.8214	-8.873	7.11E-19
6	rs371225543	32601968	T	ADD	136982	0.7633	-8.947	3.65E-19
6	rs139238973	32602002	A	ADD	140577	1.241	9.905	3.95E-23
6	rs79428469	32602034	C	ADD	143957	0.7521	-10.08	6.67E-24
6	rs72848263	32602065	C	ADD	145554	0.7603	-9.88	5.06E-23
6	rs9272214	32602096	A	ADD	143403	0.8122	-9.595	8.43E-22
6	rs34145408	32602124	T	ADD	147928	0.7683	-9.729	2.27E-22
6	rs3104371	32602137	G	ADD	148404	1.208	7.584	3.35E-14
6	rs17211454	32602179	A	ADD	147885	0.77	-9.637	5.59E-22
6	rs17205114	32602196	G	ADD	147889	0.77	-9.636	5.63E-22
6	rs17205128	32602217	G	ADD	145772	0.7633	-9.745	1.94E-22
6	rs17205135	32602222	G	ADD	145773	0.7633	-9.746	1.92E-22
6	rs9272218	32602238	T	ADD	148780	0.8189	-9.392	5.87E-21
6	rs9272221	32602334	A	ADD	145382	0.8113	-9.715	2.60E-22
6	rs9272226	32602396	C	ADD	148783	1.251	10.71	9.56E-27

6	rs17211510	32602430	A	ADD	148783	0.7679	-9.858	6.31E-23
6	rs17205170	32602483	T	ADD	148762	0.8367	-6.173	6.70E-10
6	6:32602495_ACT_A	32602495	A	ADD	148766	0.6214	-8.083	6.31E-16
6	rs17205177	32602518	G	ADD	148781	0.7674	-9.879	5.14E-23
6	rs17205184	32602551	A	ADD	148674	0.794	-8.059	7.67E-16
6	rs17205191	32602556	A	ADD	147577	0.7844	-9.364	7.68E-21
6	rs9272229	32602568	T	ADD	147585	0.8138	-9.725	2.35E-22
6	rs17211545	32602608	C	ADD	148739	0.7672	-9.881	5.03E-23
6	rs9272231	32602617	T	ADD	147718	0.8185	-9.359	8.06E-21
6	rs9272232	32602630	T	ADD	147797	0.8136	-9.628	6.10E-22
6	rs139299944	32602665	CT	ADD	148384	0.8242	-8.38	5.28E-17
6	rs17211580	32602754	A	ADD	148746	0.7678	-9.858	6.30E-23
6	rs9272239	32602778	G	ADD	148250	0.8184	-9.402	5.35E-21
6	rs9272242	32602790	C	ADD	148252	0.8183	-9.405	5.21E-21
6	rs17211601	32602817	G	ADD	148722	0.7677	-9.861	6.17E-23
6	rs28533091	32602864	A	ADD	148722	0.7677	-9.861	6.17E-23
6	rs9272245	32602872	C	ADD	147763	1.259	10.66	1.59E-26
6	rs28694749	32603029	C	ADD	148737	0.7683	-9.835	7.96E-23
6	rs9272254	32603098	T	ADD	148390	0.8194	-9.348	8.89E-21
6	6:32603185_TTGAC_T	32603185	T	ADD	148667	0.7713	-5.962	2.49E-09
6	rs9272263	32603295	C	ADD	147644	0.8352	-6.425	1.32E-10
6	rs62404084	32603321	T	ADD	148562	0.7946	-8.009	1.15E-15
6	rs28407322	32603355	C	ADD	148628	0.7686	-9.8	1.13E-22
6	rs115878164	32603405	T	ADD	148679	0.6249	-7.984	1.42E-15
6	rs9272265	32603412	T	ADD	148694	0.8191	-9.377	6.78E-21
6	rs9272266	32603416	A	ADD	148368	0.7689	-9.801	1.12E-22
6	rs28675927	32603488	G	ADD	148472	0.7681	-9.818	9.45E-23
6	rs576432697	32603715	CT	ADD	147470	0.626	-7.566	3.84E-14
6	rs1391371	32603798	T	ADD	148155	1.204	7.443	9.82E-14
6	rs386699532	32604008	ATTCTT	ADD	148111	1.137	5.902	3.59E-09
6	rs9272318	32604059	G	ADD	146155	1.154	6.608	3.90E-11
6	rs9272323	32604151	T	ADD	146763	1.141	6.039	1.55E-09
6	rs9272335	32604259	T	ADD	144166	1.15	6.408	1.47E-10
6	rs62404085	32604384	A	ADD	148783	0.6255	-7.989	1.37E-15
6	rs9272347	32604394	C	ADD	145208	1.152	6.53	6.56E-11

6	rs9272353	32604456	C	ADD	135679	1.255	8.05	8.28E-16
6	rs9272358	32604538	A	ADD	135111	1.158	6.28	3.39E-10
6	rs9272362	32604564	T	ADD	135062	1.158	6.3	2.98E-10
6	rs9272363	32604585	T	ADD	135333	1.257	8.152	3.57E-16
6	rs9272365	32604621	C	ADD	144640	1.151	6.42	1.36E-10
6	rs9272366	32604622	A	ADD	144711	1.146	6.249	4.13E-10
6	rs9272368	32604642	G	ADD	144685	1.146	6.244	4.27E-10
6	rs9272370	32604644	A	ADD	144685	1.146	6.244	4.27E-10
6	rs62404086	32604660	T	ADD	148241	0.6211	-7.947	1.92E-15
6	rs9272378	32604718	G	ADD	143988	1.153	6.527	6.73E-11
6	rs9272380	32604732	A	ADD	143964	1.153	6.504	7.83E-11
6	rs9272381	32604742	C	ADD	145146	1.229	8.402	4.38E-17
6	rs6938008	32604743	C	ADD	143321	1.141	6.122	9.25E-10
6	rs9272383	32604749	T	ADD	143983	0.8005	-10.3	6.73E-25
6	rs9272385	32604763	C	ADD	145047	1.23	8.427	3.54E-17
6	rs9272390	32604795	T	ADD	144536	1.233	8.517	1.64E-17
6	rs111590907	32604865	T	ADD	148708	0.6217	-8.072	6.92E-16
6	rs9272407	32604931	A	ADD	144159	1.15	6.382	1.75E-10
6	rs9272417	32605078	G	ADD	145869	1.227	8.137	4.05E-16
6	rs9272420	32605101	G	ADD	145840	1.151	6.475	9.51E-11
6	rs9272422	32605132	C	ADD	147417	1.22	8.256	1.51E-16
6	rs9272425	32605178	C	ADD	145863	1.229	8.413	4.00E-17
6	rs1129735	32605295	T	ADD	147833	1.141	6.131	8.74E-10
6	rs9272435	32605336	A	ADD	145791	1.153	6.534	6.41E-11
6	rs9272443	32605418	A	ADD	144777	1.154	6.561	5.35E-11
6	rs9272444	32605419	C	ADD	144777	1.154	6.561	5.35E-11
6	rs9272456	32605555	A	ADD	145770	1.228	8.333	7.85E-17
6	rs9272461	32605609	A	ADD	147315	1.204	7.361	1.83E-13
6	rs9272463	32605646	G	ADD	144839	1.151	6.4	1.55E-10
6	rs9272466	32605696	T	ADD	144823	1.224	8.062	7.52E-16
6	rs9272472	32605724	G	ADD	144506	1.153	6.487	8.74E-11
6	rs34481484	32605861	G	ADD	148654	0.6244	-7.991	1.34E-15
6	rs115444659	32605950	T	ADD	148700	0.6219	-8.066	7.24E-16
6	rs9272485	32605972	G	ADD	144573	1.149	6.309	2.81E-10
6	6:32606018_TTTC_T	32606018	T	ADD	145424	1.224	8.067	7.20E-16
6	rs9272492	32606052	G	ADD	147044	1.145	6.289	3.20E-10
6	rs9272498	32606217	A	ADD	143341	1.154	6.495	8.31E-11
6	rs9272503	32606286	C	ADD	143483	1.156	6.581	4.66E-11

6	rs9272510	32606367	G	ADD	142429	1.153	6.449	1.12E-10
6	rs9272514	32606385	T	ADD	142414	1.153	6.463	1.03E-10
6	rs527732063	32606476	A	ADD	123041	0.8267	-6.465	1.01E-10
6	rs567796452	32606482	C	ADD	147418	0.6277	-7.522	5.39E-14
6	rs531816759	32606483	C	ADD	147418	0.6277	-7.522	5.39E-14
6	rs113409319	32606500	GGA	ADD	148376	0.6256	-7.927	2.25E-15
6	rs373801706	32606560	G	ADD	146762	0.6245	-7.993	1.32E-15
6	rs41268938	32606616	T	ADD	143903	1.23	8.293	1.10E-16
6	rs28383356	32606646	C	ADD	141569	1.155	6.511	7.48E-11
6	rs28375776	32606658	G	ADD	147738	0.7498	-7.802	6.11E-15
6	rs34701372	32606676	C	ADD	148554	0.6252	-7.97	1.59E-15
6	rs28636516	32606701	G	ADD	148180	0.7738	-7.377	1.62E-13
6	rs75983419	32606721	T	ADD	148621	0.6224	-8.052	8.16E-16
6	rs28383357	32606732	A	ADD	142263	1.15	6.334	2.40E-10
6	rs74379225	32606742	G	ADD	145032	0.6342	-7.719	1.17E-14
6	rs28383361	32606846	A	ADD	143488	1.15	6.354	2.10E-10
6	rs28383362	32606864	C	ADD	143617	1.15	6.358	2.04E-10
6	rs28383363	32606868	C	ADD	143617	1.15	6.358	2.04E-10
6	rs9272544	32606878	G	ADD	142900	1.231	9.629	6.03E-22
6	rs28383365	32606942	A	ADD	144802	1.149	6.329	2.47E-10
6	rs78702533	32606960	T	ADD	148529	0.6249	-7.977	1.50E-15
6	rs41268940	32606987	G	ADD	145857	1.224	8.084	6.27E-16
6	rs79772777	32607017	A	ADD	147306	0.6284	-7.575	3.60E-14
6	rs28383367	32607031	T	ADD	148068	1.134	5.801	6.60E-09
6	rs41268942	32607126	G	ADD	148351	1.207	7.568	3.80E-14
6	rs28383370	32607152	C	ADD	147797	1.134	5.761	8.35E-09
6	rs28383371	32607154	T	ADD	147797	1.134	5.761	8.35E-09
6	rs28383372	32607174	T	ADD	146370	1.148	6.365	1.95E-10
6	rs28383373	32607193	C	ADD	146257	1.148	6.339	2.32E-10
6	rs79298326	32607221	G	ADD	148035	0.6185	-8.135	4.11E-16
6	rs80217639	32607224	T	ADD	148035	0.6185	-8.135	4.11E-16
6	rs76867035	32607270	G	ADD	148470	0.6197	-8.089	5.99E-16
6	rs35248896	32607370	T	ADD	144433	1.209	7.353	1.93E-13
6	rs63749607	32607406	G	ADD	148456	0.6197	-8.103	5.38E-16
6	rs28383386	32607567	A	ADD	145182	1.142	6.061	1.35E-09
6	rs28383387	32607575	T	ADD	143996	1.154	6.52	7.04E-11
6	rs41269944	32607627	T	ADD	146059	1.207	7.412	1.24E-13

6	rs41269945	32607853	T	ADD	140422	1.233	7.772	7.74E-15
6	rs36221399	32607861	T	ADD	148501	0.6185	-8.121	4.61E-16
6	6:32607898_AG_A	32607898	A	ADD	147950	1.141	6.064	1.33E-09
6	rs17426593	32608077	C	ADD	140882	1.242	8.167	3.15E-16
6	rs28383405	32608108	T	ADD	148554	1.133	5.744	9.23E-09
6	rs28383406	32608179	C	ADD	148711	1.134	5.794	6.86E-09
6	rs41269955	32608269	A	ADD	133418	1.221	6.662	2.70E-11
6	rs370803972	32608334	G	ADD	108797	0.805	-8.823	1.11E-18
6	rs36220784	32608350	A	ADD	148489	0.6188	-8.113	4.94E-16
6	rs34430979	32608425	A	ADD	146909	0.628	-7.481	7.36E-14
6	rs34141382	32608478	C	ADD	136297	1.235	7.416	1.20E-13
6	rs4286817	32608821	A	ADD	130579	1.249	8.804	1.32E-18
6	rs75046941	32608855	G	ADD	146841	0.6297	-7.451	9.28E-14
6	rs75503615	32608934	T	ADD	147776	0.6749	-7.169	7.53E-13
6	rs78824971	32608946	A	ADD	147737	0.6784	-7.095	1.29E-12
6	rs78496075	32608967	G	ADD	146770	0.6644	-7.397	1.39E-13
6	rs75174795	32608977	G	ADD	146988	0.6801	-7.057	1.71E-12
6	rs34763586	32608998	C	ADD	138735	1.221	7.272	3.55E-13
6	rs28383439	32609042	A	ADD	148095	1.137	5.91	3.43E-09
6	rs36219699	32609195	A	ADD	147719	0.6745	-7.181	6.94E-13
6	rs12722072	32609239	A	ADD	138316	1.219	7.213	5.49E-13
6	rs4467816	32609371	C	ADD	146152	0.7808	-9.277	1.75E-20
6	rs28383455	32609380	T	ADD	146672	1.141	6.031	1.63E-09
6	rs28383457	32609456	T	ADD	148382	1.137	5.883	4.03E-09
6	rs35874654	32609479	A	ADD	148260	1.214	7.781	7.22E-15
6	rs34965214	32609545	T	ADD	143417	1.223	7.727	1.10E-14
6	rs28383460	32609586	G	ADD	148480	1.136	5.862	4.59E-09
6	rs41268284	32609621	G	ADD	147415	1.204	7.383	1.54E-13
6	rs9272730	32609634	G	ADD	147512	1.141	6.05	1.45E-09
6	rs9272731	32609645	C	ADD	147518	1.141	6.039	1.56E-09
6	rs9272736	32609671	A	ADD	143449	1.151	6.383	1.74E-10
6	rs374819369	32609696	AGAAG	ADD	134225	1.221	7.9	2.79E-15
6	rs9272742	32609722	T	ADD	147778	1.22	7.997	1.27E-15
6	rs1048173	32609855	C	ADD	146232	1.136	5.819	5.91E-09
6	rs41547417	32609874	G	ADD	147878	0.6733	-7.204	5.84E-13
6	rs7990	32609965	A	ADD	137500	1.241	6.971	3.14E-12

6	rs2308890	32610008	T	ADD	147817	1.142	6.105	1.03E-09
6	rs35975014	32610075	G	ADD	147255	0.6645	-7.376	1.63E-13
6	6:32610099_TC_T	32610099	T	ADD	148350	1.135	5.8	6.62E-09
6	rs75076975	32610134	A	ADD	148340	1.135	5.806	6.40E-09
6	rs748519761	32610142	T	ADD	125621	1.167	6.537	6.29E-11
6	rs62404092	32610151	A	ADD	147411	0.674	-7.185	6.70E-13
6	rs397843773	32610171	T	ADD	148341	1.134	5.788	7.12E-09
6	rs79351547	32610196	A	ADD	148348	1.135	5.806	6.41E-09
6	rs116022266	32610201	A	ADD	148346	1.135	5.806	6.40E-09
6	rs9272774	32610255	A	ADD	148386	1.135	5.808	6.34E-09
6	rs9272775	32610257	C	ADD	148277	1.133	5.76	8.43E-09
6	rs78712211	32610300	T	ADD	146968	0.6629	-7.397	1.39E-13
6	rs77179964	32610313	G	ADD	147995	0.6734	-7.2	6.04E-13
6	rs9272785	32610401	A	ADD	140249	1.226	7.59	3.20E-14
6	rs1048414	32610478	G	ADD	147722	1.137	5.878	4.14E-09
6	rs1048419	32610481	C	ADD	147722	1.137	5.878	4.14E-09
6	rs9272803	32610591	T	ADD	147490	1.135	5.78	7.49E-09
6	rs36027005	32610643	T	ADD	147635	0.6748	-7.165	7.77E-13
6	rs35444025	32610688	C	ADD	147632	0.6747	-7.166	7.74E-13
6	rs62404094	32610696	A	ADD	147624	0.6747	-7.166	7.73E-13
6	rs7708	32610749	A	ADD	143449	0.7147	-6.445	1.15E-10
6	rs9272896	32610806	C	ADD	127750	1.157	5.924	3.14E-09
6	rs9272899	32610809	T	ADD	125796	1.161	6.013	1.83E-09
6	rs28371169	32610819	G	ADD	129960	1.162	6.23	4.65E-10
6	rs9272905	32610836	T	ADD	129476	1.161	6.166	7.01E-10
6	rs9272913	32610860	T	ADD	129364	1.16	6.1	1.06E-09
6	rs9272914	32610863	G	ADD	129364	1.16	6.1	1.06E-09
6	rs36216730	32611094	T	ADD	147860	0.675	-7.166	7.71E-13
6	rs62404112	32611122	C	ADD	147863	0.675	-7.166	7.73E-13
6	rs36216571	32611215	T	ADD	147889	0.6733	-7.205	5.82E-13
6	rs45512899	32611299	T	ADD	147991	0.6754	-7.156	8.29E-13
6	rs9273014	32611650	A	ADD	145952	1.136	5.833	5.45E-09
6	rs36214506	32611709	C	ADD	148048	0.6732	-7.206	5.75E-13
6	rs36214159	32611759	G	ADD	148026	0.6731	-7.208	5.67E-13
6	rs78846394	32612096	G	ADD	148194	0.6716	-7.249	4.19E-13
6	rs9273063	32612166	T	ADD	117985	1.176	5.914	3.35E-09
6	rs9273070	32612241	T	ADD	118489	1.185	6.224	4.83E-10
6	rs9273073	32612279	C	ADD	116284	1.164	5.783	7.34E-09

6	rs62404117	32612417	A	ADD	148573	0.6175	-8.133	4.18E-16
6	rs62404118	32612522	C	ADD	148179	0.6746	-7.178	7.05E-13
6	rs9273107	32612620	G	ADD	123494	1.285	6.64	3.13E-11
6	rs62404119	32612905	A	ADD	148454	0.6188	-8.115	4.84E-16
6	rs62404120	32612962	C	ADD	148244	0.6729	-7.215	5.38E-13
6	rs9273148	32612982	A	ADD	128934	1.18	6.752	1.46E-11
6	rs9273165	32613124	G	ADD	148424	0.6179	-8.141	3.91E-16
6	rs62404121	32613349	A	ADD	148324	0.6726	-7.222	5.12E-13
6	rs9282052	32613360	CA	ADD	143447	1.209	7.294	3.01E-13
6	rs184755809	32613600	A	ADD	105437	1.204	5.831	5.52E-09
6	rs193292687	32614432	A	ADD	126413	1.25	6.134	8.57E-10
6	rs78568738	32614573	C	ADD	148352	0.6735	-7.207	5.72E-13
6	rs79139281	32614768	C	ADD	148359	0.6753	-7.168	7.62E-13
6	rs114336758	32614829	C	ADD	129421	1.281	6.443	1.17E-10
6	rs117601730	32614833	G	ADD	133803	1.296	6.741	1.57E-11
6	rs62404122	32614857	C	ADD	148357	0.6753	-7.17	7.49E-13
6	rs78337030	32615310	G	ADD	148385	0.6755	-7.163	7.90E-13
6	rs561575493	32615452	A	ADD	123493	1.23	5.779	7.53E-09
6	rs28479464	32615457	C	ADD	148250	0.8074	-7.553	4.26E-14
6	rs772081778	32615636	G	ADD	131214	1.276	7.005	2.46E-12
6	rs62404123	32615963	G	ADD	148377	0.6753	-7.169	7.58E-13
6	rs62404124	32616074	A	ADD	138053	0.7018	-6.585	4.54E-11
6	rs58069912	32616339	C	ADD	135115	0.6884	-7.001	2.54E-12
6	rs79088780	32617512	T	ADD	100615	0.852	-5.893	3.79E-09
6	rs28798705	32617809	G	ADD	131028	0.8065	-8.333	7.88E-17
6	rs62404127	32618229	A	ADD	141429	0.6871	-6.856	7.07E-12
6	rs575147125	32619144	G	ADD	105531	1.288	7.159	8.10E-13
6	rs28651573	32619218	G	ADD	93757	1.208	6.741	1.58E-11
6	rs2858877	32620549	A	ADD	117075	1.231	6.185	6.20E-10
6	rs77062257	32620764	T	ADD	109798	1.197	5.996	2.02E-09
6	6:32620828_G_A	32620828	A	ADD	140704	1.284	6.346	2.22E-10
6	rs75150448	32620831	A	ADD	148299	0.6728	-7.227	4.94E-13
6	6:32621290_C_T	32621290	T	ADD	91224	1.226	6.289	3.19E-10
6	rs28590669	32621443	G	ADD	148265	0.6567	-9.6	7.98E-22
6	rs28704178	32621483	A	ADD	148366	0.6563	-9.614	6.98E-22
6	6:32621590_T_C	32621590	C	ADD	105261	1.186	6.36	2.02E-10
6	rs28886951	32622035	T	ADD	148359	0.6561	-9.62	6.57E-22

6	rs35932914	32622150	A	ADD	148386	0.6561	-9.619	6.66E-22
6	rs28374650	32623367	T	ADD	148010	0.7813	-9.394	5.76E-21
6	rs9273348	32623487	G	ADD	87209	1.204	6.421	1.36E-10
6	rs73732214	32623528	A	ADD	140085	0.7426	-8.256	1.51E-16
6	rs55986091	32623713	A	ADD	148231	0.656	-9.623	6.42E-22
6	rs4637681	32623928	T	ADD	111139	1.273	9.803	1.09E-22
6	rs55866704	32624001	T	ADD	143760	0.689	-9.028	1.74E-19
6	rs185774274	32624515	C	ADD	102269	1.221	5.976	2.29E-09
6	rs9501646	32624843	A	ADD	103018	1.295	7.091	1.33E-12
6	6:32624923_C_A	32624923	A	ADD	97109	1.187	5.801	6.59E-09
6	rs4548048	32625022	T	ADD	141187	0.6776	-9.145	5.95E-20
6	rs4639377	32625283	T	ADD	103891	1.195	6.632	3.30E-11
6	6:32625319_A_G	32625319	G	ADD	98712	1.174	5.808	6.33E-09
6	rs34046799	32625442	A	ADD	145124	0.7094	-8.623	6.51E-18
6	rs28442287	32626007	T	ADD	148709	0.6616	-9.506	1.97E-21
6	rs28584179	32626119	T	ADD	148783	0.6607	-9.554	1.25E-21
6	rs6928482	32626249	C	ADD	148783	1.136	6.047	1.48E-09
6	rs28407950	32626348	T	ADD	148554	0.7818	-9.392	5.89E-21
6	rs112796194	32626392	C	ADD	148552	0.7828	-9.35	8.73E-21
6	rs34983241	32626397	T	ADD	148552	0.7828	-9.35	8.73E-21
6	rs34004019	32626403	G	ADD	122094	0.747	-8.371	5.72E-17
6	rs28515515	32626432	T	ADD	148552	0.7828	-9.35	8.73E-21
6	rs28414666	32626451	A	ADD	148449	0.7418	-10.57	4.28E-26
6	rs28672722	32626537	T	ADD	148447	0.7563	-10.17	2.69E-24
6	rs34386495	32626730	T	ADD	148538	0.8157	-7.238	4.55E-13
6	rs28718232	32626750	G	ADD	148450	0.7417	-10.57	4.16E-26
6	rs28731358	32626883	C	ADD	144082	0.7812	-9.324	1.13E-20
6	rs28414125	32626975	C	ADD	148535	0.7383	-8.083	6.30E-16
6	rs4713574	32627038	G	ADD	128725	0.7797	-10.64	1.89E-26
6	rs28579922	32627044	A	ADD	148547	0.6581	-9.578	9.87E-22
6	rs9273395	32627094	T	ADD	134007	1.158	6.478	9.28E-11
6	6:32627122_T_C	32627122	C	ADD	123342	1.247	6.013	1.82E-09
6	rs4993986	32627652	G	ADD	148735	1.154	6.613	3.78E-11
6	rs6689	32627700	G	ADD	148502	0.8398	-6.132	8.70E-10
6	rs41270875	32628272	G	ADD	139530	1.169	5.798	6.69E-09
6	rs9273501	32628361	A	ADD	148756	1.161	6.881	5.92E-12
6	rs9273505	32628420	T	ADD	148757	1.154	6.61	3.83E-11
6	rs41270883	32628597	T	ADD	145511	1.168	5.827	5.65E-09

6	rs9273608	32629110	T	ADD	134925	1.17	6.734	1.65E-11
6	rs28724240	32629328	A	ADD	112442	1.294	7.196	6.18E-13
6	rs28724243	32629347	C	ADD	126101	1.184	6.615	3.71E-11
6	rs28724245	32629400	G	ADD	147838	1.152	6.481	9.14E-11
6	rs28724249	32629509	C	ADD	148584	1.154	6.604	4.01E-11
6	rs28724251	32629535	A	ADD	146367	1.158	6.673	2.52E-11
6	rs1130398	32629764	T	ADD	148762	1.155	6.633	3.30E-11
6	rs701564	32629809	T	ADD	148783	1.168	5.94	2.85E-09
6	rs1063323	32629891	T	ADD	148683	1.155	6.641	3.12E-11
6	rs1063321	32629955	T	ADD	148750	1.155	6.622	3.54E-11
6	rs9274094	32630155	A	ADD	142470	1.156	6.576	4.83E-11
6	rs9274129	32630230	C	ADD	129740	1.147	5.958	2.55E-09
6	rs28724260	32630240	G	ADD	129741	1.147	5.958	2.55E-09
6	rs9274150	32630276	G	ADD	129596	1.145	5.862	4.57E-09
6	rs9274167	32630317	G	ADD	124590	1.154	6.086	1.16E-09
6	6:32630610_C_A	32630610	A	ADD	129751	1.171	6.913	4.73E-12
6	rs9274193	32630684	T	ADD	135583	1.162	6.589	4.42E-11
6	rs9274194	32630689	C	ADD	110927	1.195	5.97	2.37E-09
6	rs41270925	32630692	A	ADD	125842	1.183	6.014	1.81E-09
6	rs9274195	32630701	T	ADD	126447	1.178	7.059	1.68E-12
6	rs9274198	32630720	T	ADD	126210	1.18	7.154	8.42E-13
6	6:32630721_A_G	32630721	G	ADD	126300	1.176	6.995	2.65E-12
6	rs779124117	32630758	A	ADD	126938	1.177	7.053	1.75E-12
6	rs9274227	32631142	G	ADD	116876	1.155	5.802	6.55E-09
6	rs9274229	32631152	A	ADD	122722	1.149	5.855	4.78E-09
6	6:32631178_G_A	32631178	A	ADD	122871	1.151	5.943	2.81E-09
6	rs9274235	32631207	T	ADD	135820	1.174	7.049	1.80E-12
6	6:32631218_T_G	32631218	G	ADD	135796	1.174	7.054	1.74E-12
6	6:32631273_A_T	32631273	T	ADD	135534	1.172	6.969	3.19E-12
6	6:32631300_A_G	32631300	G	ADD	143723	1.178	6.031	1.63E-09
6	6:32631428_T_C	32631428	C	ADD	136905	1.174	7.16	8.07E-13
6	rs9274278	32631711	T	ADD	111693	1.171	6.476	9.45E-11
6	rs9274286	32631802	A	ADD	115298	1.166	6.402	1.54E-10
6	rs28724262	32631889	C	ADD	114654	1.191	7.002	2.52E-12
6	rs28724264	32631937	G	ADD	112770	1.183	6.708	1.97E-11
6	rs9274306	32632006	T	ADD	136957	0.8789	-5.77	7.93E-09
6	rs9274346	32632367	T	ADD	130457	1.173	7.036	1.98E-12

6	rs9274349	32632408	T	ADD	138018	1.162	6.695	2.16E-11
6	rs41263825	32632543	A	ADD	101020	1.271	6.681	2.38E-11
6	rs1130397	32632587	G	ADD	145792	0.836	-6.089	1.14E-09
6	rs17412833	32632598	T	ADD	145785	0.8361	-6.085	1.16E-09
6	rs1130392	32632628	C	ADD	127662	0.868	-5.784	7.28E-09
6	rs1049082	32632744	T	ADD	111235	1.163	6.013	1.82E-09
6	rs281862065	32632770	G	ADD	121094	0.7438	-8.094	5.79E-16
6	rs1049068	32632777	T	ADD	147328	0.6682	-9.317	1.20E-20
6	rs1130375	32632820	G	ADD	148783	1.167	5.899	3.66E-09
6	rs9274407	32632832	A	ADD	146902	1.17	5.745	9.22E-09
6	rs76141774	32633341	A	ADD	146832	0.6428	-7.268	3.66E-13
6	rs62404143	32633467	G	ADD	148430	0.6387	-7.767	8.01E-15
6	rs28746843	32634137	C	ADD	96217	1.205	5.744	9.26E-09
6	rs62404144	32634990	T	ADD	148523	0.6386	-7.772	7.73E-15
6	rs6913173	32635364	C	ADD	139124	1.139	5.94	2.85E-09
6	rs9274679	32636653	C	ADD	148524	1.145	6.399	1.56E-10
6	rs9274683	32636780	A	ADD	148373	1.143	6.327	2.49E-10
6	rs9274684	32636785	T	ADD	148766	1.143	6.357	2.06E-10
6	rs9274685	32636844	C	ADD	148728	1.143	6.32	2.61E-10
6	rs9274687	32636882	T	ADD	148754	1.143	6.357	2.06E-10
6	rs9274689	32636930	C	ADD	129406	1.15	6.064	1.33E-09
6	rs9274690	32636940	C	ADD	144308	1.147	6.381	1.76E-10
6	rs9274691	32636957	C	ADD	134794	1.158	6.6	4.12E-11
6	rs9274692	32636958	A	ADD	134794	1.158	6.6	4.12E-11
6	rs17211818	32636963	A	ADD	138493	1.21	6.536	6.34E-11
6	rs9274693	32636972	T	ADD	138397	1.155	6.606	3.94E-11
6	rs1626993	32636991	T	ADD	148177	1.158	6.769	1.30E-11
6	rs9274695	32636997	T	ADD	148158	1.151	6.395	1.61E-10
6	rs9274696	32637007	C	ADD	148571	1.145	6.398	1.57E-10
6	rs9274697	32637016	G	ADD	148783	1.141	6.386	1.71E-10
6	rs9274698	32637095	C	ADD	148711	1.143	6.355	2.08E-10
6	rs17211868	32637135	G	ADD	145798	1.17	5.853	4.82E-09
6	rs17205521	32637138	A	ADD	147149	1.177	6.161	7.24E-10
6	rs9274702	32637184	A	ADD	144993	1.14	6.146	7.95E-10
6	rs9274705	32637245	A	ADD	144649	1.144	6.309	2.80E-10
6	rs9274707	32637266	G	ADD	144646	1.144	6.314	2.72E-10
6	rs9274708	32637267	A	ADD	144646	1.144	6.314	2.72E-10
6	rs9274710	32637287	G	ADD	144932	1.145	6.348	2.19E-10

6	rs17211951	32637315	A	ADD	142278	1.176	5.779	7.53E-09
6	rs17211958	32637316	G	ADD	142132	1.175	5.8	6.65E-09
6	rs17205605	32637327	T	ADD	143469	1.183	6.127	8.98E-10
6	rs17205612	32637333	T	ADD	143372	1.179	6.095	1.09E-09
6	rs9274713	32637341	T	ADD	144396	1.148	6.444	1.16E-10
6	rs17211965	32637355	G	ADD	143377	1.179	6.089	1.13E-09
6	rs9274715	32637391	A	ADD	134083	1.171	7.129	1.01E-12
6	rs17211993	32637394	G	ADD	143744	1.18	6.144	8.03E-10
6	rs9274717	32637454	A	ADD	131338	1.158	6.479	9.26E-11
6	rs9274718	32637469	C	ADD	121168	1.161	6.194	5.87E-10
6	rs9274719	32637470	C	ADD	142507	1.137	5.933	2.98E-09
6	rs9274720	32637479	A	ADD	142536	1.137	5.924	3.15E-09
6	rs3997832	32637497	C	ADD	142035	1.15	6.376	1.81E-10
6	rs28746878	32637515	C	ADD	134513	1.259	5.885	3.97E-09
6	rs9274725	32637573	T	ADD	148617	1.137	6.085	1.17E-09
6	rs9274726	32637593	G	ADD	148695	1.144	6.365	1.95E-10
6	rs34400217	32637750	T	ADD	145649	1.169	5.902	3.59E-09
6	rs35744187	32637756	A	ADD	146832	1.166	5.931	3.01E-09
6	rs9274730	32637817	A	ADD	131213	1.168	6.887	5.70E-12
6	rs35135261	32637822	T	ADD	140798	1.176	5.803	6.52E-09
6	rs9274731	32637823	G	ADD	131059	1.167	6.852	7.28E-12
6	rs9274733	32637874	T	ADD	148039	1.145	6.417	1.39E-10
6	rs9274736	32637905	C	ADD	139555	1.149	6.35	2.15E-10
6	rs9274738	32637909	C	ADD	139555	1.149	6.35	2.15E-10
6	rs35402595	32637912	C	ADD	147597	1.173	6.069	1.29E-09
6	rs9274740	32637968	T	ADD	147248	1.148	6.514	7.31E-11
6	rs9274741	32637994	T	ADD	148760	0.8696	-6.615	3.70E-11
6	rs9274743	32638042	G	ADD	147231	1.148	6.501	7.99E-11
6	rs9274744	32638054	A	ADD	146622	1.145	6.379	1.78E-10
6	rs9274745	32638058	C	ADD	146693	0.8677	-6.661	2.72E-11
6	rs9274746	32638060	A	ADD	146622	1.145	6.379	1.78E-10
6	rs9274748	32638086	A	ADD	138473	1.149	6.342	2.27E-10
6	rs9274749	32638094	A	ADD	138469	1.149	6.34	2.30E-10
6	rs9274751	32638138	C	ADD	138540	1.155	6.606	3.95E-11
6	rs9274752	32638163	A	ADD	138578	1.155	6.59	4.41E-11
6	rs9274753	32638176	T	ADD	138581	1.155	6.589	4.43E-11
6	rs9274754	32638261	C	ADD	139214	1.152	6.476	9.43E-11
6	rs9274755	32638262	A	ADD	139214	1.152	6.476	9.43E-11

6	rs9274756	32638269	T	ADD	139214	1.152	6.476	9.43E-11
6	rs9274758	32638290	G	ADD	139320	1.153	6.509	7.58E-11
6	rs9274759	32638315	A	ADD	148722	1.142	6.283	3.32E-10
6	rs9274760	32638348	G	ADD	139310	1.152	6.502	7.93E-11
6	rs9274763	32638427	T	ADD	139317	1.152	6.501	7.99E-11
6	rs9274764	32638490	G	ADD	148766	1.142	6.294	3.10E-10
6	rs9274769	32638547	C	ADD	139255	1.153	6.526	6.74E-11
6	rs9274771	32638581	C	ADD	138370	1.151	6.434	1.24E-10
6	rs9274772	32638583	C	ADD	138370	1.151	6.434	1.24E-10
6	rs9274773	32638620	G	ADD	138372	1.152	6.444	1.16E-10
6	rs9274774	32638630	G	ADD	138372	1.152	6.444	1.16E-10
6	rs9274778	32638694	A	ADD	136950	1.152	6.431	1.27E-10
6	rs9274790	32639155	G	ADD	138020	1.154	6.528	6.66E-11
6	rs9274792	32639224	C	ADD	138411	1.152	6.458	1.06E-10
6	rs9274794	32639264	G	ADD	137667	1.148	6.274	3.53E-10
6	rs112453089	32639270	G	ADD	137668	1.148	6.272	3.56E-10
6	rs6914573	32639368	T	ADD	139136	1.152	6.482	9.05E-11
6	rs111983899	32639389	T	ADD	128959	1.147	5.961	2.51E-09
6	rs113374318	32639415	A	ADD	129365	1.146	5.939	2.86E-09
6	rs9274805	32639429	T	ADD	130828	1.149	6.125	9.05E-10
6	rs9274806	32639441	G	ADD	130835	1.149	6.121	9.28E-10
6	rs199884023	32639449	A	ADD	130849	1.149	6.128	8.92E-10
6	rs1794493	32639578	G	ADD	136648	1.138	5.853	4.81E-09
6	rs9274824	32639702	T	ADD	123772	1.173	6.827	8.64E-12
6	rs9274828	32639737	G	ADD	133597	1.146	6.073	1.26E-09
6	rs9274834	32639895	G	ADD	128488	1.169	6.868	6.50E-12
6	rs41316211	32639909	C	ADD	136159	1.202	6.125	9.08E-10
6	rs9274836	32639956	A	ADD	128497	1.169	6.867	6.55E-12
6	rs9274839	32640015	G	ADD	138423	1.15	6.395	1.61E-10
6	rs9274843	32640096	G	ADD	136966	1.148	6.245	4.25E-10
6	rs9274853	32640420	A	ADD	138424	1.15	6.398	1.58E-10
6	rs9274855	32640494	A	ADD	127122	1.172	6.95	3.64E-12
6	rs9274857	32640542	G	ADD	124055	1.176	6.999	2.57E-12
6	rs9274861	32640592	G	ADD	134475	1.152	6.376	1.82E-10
6	rs9274864	32640646	G	ADD	136496	1.152	6.423	1.34E-10
6	rs9274865	32640671	T	ADD	136493	1.152	6.425	1.32E-10
6	rs9274867	32640745	A	ADD	127858	1.15	6.059	1.37E-09
6	rs9274869	32640759	T	ADD	127629	1.149	6.018	1.76E-09

6	rs9274871	32640792	G	ADD	119184	1.185	7.099	1.26E-12
6	rs9274872	32640793	G	ADD	119184	1.185	7.099	1.26E-12
6	rs62404145	32641178	T	ADD	148463	0.6345	-7.858	3.90E-15
6	rs9274943	32641784	T	ADD	101880	1.172	5.817	5.99E-09
6	rs9274944	32641786	A	ADD	101880	1.172	5.817	5.99E-09
6	rs9274954	32641878	T	ADD	120380	1.159	6.136	8.49E-10
6	rs9274955	32641890	G	ADD	120733	1.164	6.339	2.32E-10
6	rs9274957	32641914	C	ADD	121232	1.165	6.393	1.62E-10
6	rs9274962	32641936	A	ADD	124067	1.161	6.339	2.31E-10
6	rs9274967	32642030	C	ADD	126292	1.166	6.698	2.12E-11
6	rs9274969	32642073	T	ADD	117298	1.165	6.313	2.74E-10
6	rs9274973	32642147	G	ADD	131750	1.158	6.546	5.90E-11
6	rs9274974	32642152	T	ADD	131756	1.158	6.55	5.76E-11
6	rs9274977	32642247	G	ADD	126330	1.162	6.531	6.55E-11
6	rs9274978	32642277	C	ADD	125883	1.157	6.321	2.60E-10
6	rs9274980	32642290	T	ADD	125932	1.158	6.36	2.02E-10
6	rs9274986	32642386	A	ADD	130459	1.164	6.715	1.89E-11
6	rs9274987	32642405	T	ADD	129793	1.161	6.587	4.49E-11
6	rs9274988	32642406	T	ADD	129793	1.161	6.587	4.49E-11
6	rs9280073	32642469	T	ADD	114975	1.16	5.982	2.21E-09
6	rs9274995	32642615	T	ADD	143684	1.132	5.795	6.85E-09
6	rs9274996	32642641	T	ADD	144085	1.134	5.844	5.08E-09
6	rs9274997	32642673	T	ADD	142945	1.141	6.124	9.14E-10
6	rs62404146	32642679	C	ADD	148406	0.6384	-7.752	9.05E-15
6	rs9274999	32642709	A	ADD	132746	1.162	6.722	1.80E-11
6	rs9275001	32642738	T	ADD	130876	1.161	6.635	3.24E-11
6	rs9275007	32642921	A	ADD	141404	1.141	6.093	1.11E-09
6	rs9275008	32642944	C	ADD	141127	1.141	6.062	1.35E-09
6	rs9275010	32642966	T	ADD	131140	1.156	6.444	1.16E-10
6	rs9275011	32642969	T	ADD	131130	1.157	6.452	1.11E-10
6	rs9275013	32643011	C	ADD	133698	1.158	6.577	4.79E-11
6	rs9275014	32643023	A	ADD	133697	1.158	6.577	4.79E-11
6	rs28755304	32646193	T	ADD	114182	1.18	6.352	2.12E-10
6	rs114708995	32646969	T	ADD	104440	1.228	6.153	7.62E-10
6	rs28371245	32647982	C	ADD	118241	1.164	6.273	3.54E-10
6	rs28371246	32647995	C	ADD	116861	1.164	6.192	5.94E-10
6	rs9275043	32648061	C	ADD	121295	1.182	7.015	2.30E-12
6	rs9275046	32648080	C	ADD	121338	1.181	6.974	3.09E-12

6	rs9275047	32648084	G	ADD	121338	1.181	6.974	3.09E-12
6	rs9275054	32648208	G	ADD	130197	1.159	6.5	8.04E-11
6	rs9275055	32648220	C	ADD	130196	1.159	6.502	7.95E-11
6	rs9275057	32648253	G	ADD	133820	1.156	6.492	8.49E-11
6	rs9275059	32648298	A	ADD	134133	1.159	6.635	3.24E-11
6	rs9275062	32648337	C	ADD	136220	1.155	6.527	6.70E-11
6	rs9275069	32648437	G	ADD	144720	1.14	6.125	9.09E-10
6	rs9275070	32648449	T	ADD	144718	1.14	6.125	9.08E-10
6	rs9275071	32648490	A	ADD	134788	1.142	5.914	3.34E-09
6	rs9275073	32648513	G	ADD	145630	1.138	6.04	1.54E-09
6	rs9275078	32648592	C	ADD	147480	1.137	6.051	1.44E-09
6	rs9275081	32648669	T	ADD	147301	1.139	6.113	9.76E-10
6	rs9275082	32648684	C	ADD	147522	1.142	6.245	4.23E-10
6	rs114394994	32648685	T	ADD	139610	1.259	5.878	4.14E-09
6	rs9275084	32648722	G	ADD	147121	1.138	6.092	1.12E-09
6	rs9275085	32648735	A	ADD	147121	1.138	6.092	1.12E-09
6	rs9275086	32648809	G	ADD	148761	1.143	6.314	2.71E-10
6	rs9275087	32648893	C	ADD	148761	1.143	6.314	2.71E-10
6	rs9275088	32648931	A	ADD	148320	1.145	6.39	1.66E-10
6	rs9275089	32648947	T	ADD	147171	1.137	6.038	1.56E-09
6	rs545072519	32648948	C	ADD	143519	1.264	5.942	2.81E-09
6	rs9275091	32648972	T	ADD	147178	1.137	6.039	1.55E-09
6	rs9275101	32649355	A	ADD	148745	1.141	6.265	3.73E-10
6	rs9275102	32649361	A	ADD	148745	1.141	6.265	3.73E-10
6	rs9275103	32649386	C	ADD	148742	1.141	6.267	3.69E-10
6	rs9275105	32649416	T	ADD	148742	1.141	6.267	3.69E-10
6	rs9275106	32649475	C	ADD	147426	1.143	6.301	2.96E-10
6	rs9275112	32649773	A	ADD	148762	1.142	6.314	2.73E-10
6	rs9275114	32649897	A	ADD	148749	1.141	6.246	4.22E-10
6	rs9275116	32649952	C	ADD	148748	1.141	6.245	4.24E-10
6	rs9275118	32649996	G	ADD	147579	1.14	6.179	6.45E-10
6	rs9275119	32650031	A	ADD	148712	1.141	6.249	4.12E-10
6	rs9275123	32650256	T	ADD	148755	1.142	6.278	3.44E-10
6	rs9275125	32650293	T	ADD	148755	1.142	6.278	3.44E-10
6	rs9275126	32650307	T	ADD	146948	1.146	6.439	1.21E-10
6	rs9275130	32650409	C	ADD	148755	1.141	6.265	3.74E-10
6	rs9275131	32650467	T	ADD	148757	1.142	6.296	3.05E-10
6	rs9275133	32650588	G	ADD	148766	1.141	6.257	3.92E-10

6	rs9275135	32650736	C	ADD	148615	1.142	6.281	3.35E-10
6	rs9275136	32650757	T	ADD	147817	1.144	6.357	2.06E-10
6	rs9275138	32650958	G	ADD	148589	1.142	6.302	2.94E-10
6	rs9275139	32651005	A	ADD	147502	1.145	6.41	1.45E-10
6	rs9275141	32651117	G	ADD	135768	1.152	6.384	1.73E-10
6	rs9275143	32651192	T	ADD	145629	1.143	6.259	3.88E-10
6	rs9275145	32651363	T	ADD	148763	1.141	6.258	3.90E-10
6	rs9275146	32651495	T	ADD	148275	1.14	6.187	6.12E-10
6	rs9275148	32651585	C	ADD	147408	1.145	6.397	1.59E-10
6	rs2856695	32651894	G	ADD	148027	1.138	6.096	1.09E-09
6	rs3021058	32652359	C	ADD	139579	1.147	6.283	3.33E-10
6	rs3021057	32652363	T	ADD	145543	1.144	6.306	2.87E-10
6	rs9275154	32652435	T	ADD	148182	1.142	6.274	3.51E-10
6	rs9275160	32652620	A	ADD	148331	1.141	6.024	1.71E-09
6	rs9275162	32652687	C	ADD	148720	1.143	6.33	2.45E-10
6	rs2856693	32653283	T	ADD	148776	1.143	6.317	2.67E-10
6	rs9275168	32653676	T	ADD	148090	0.8687	-6.645	3.04E-11
6	rs9275169	32653721	T	ADD	148685	0.8693	-6.632	3.32E-11
6	rs9275170	32653726	G	ADD	148592	0.869	-6.645	3.03E-11
6	rs9275172	32653997	A	ADD	148764	0.8693	-6.63	3.36E-11
6	rs9275173	32654037	T	ADD	148755	0.8693	-6.633	3.29E-11
6	rs9275177	32654174	T	ADD	146960	1.153	6.685	2.30E-11
6	rs9275178	32654181	A	ADD	146952	1.153	6.685	2.30E-11
6	rs113457465	32654241	C	ADD	141551	1.274	6.139	8.33E-10
6	rs9275180	32654278	T	ADD	147552	1.147	6.474	9.53E-11
6	rs2856690	32654536	A	ADD	145415	1.14	6.12	9.34E-10
6	rs2856689	32654551	G	ADD	145460	1.14	6.122	9.22E-10
6	rs2856686	32654869	C	ADD	148075	1.144	6.35	2.16E-10
6	rs2856685	32655072	T	ADD	147842	1.142	6.285	3.29E-10
6	rs2856684	32655095	C	ADD	147842	1.142	6.285	3.29E-10
6	rs2856679	32655615	A	ADD	145235	1.137	5.993	2.07E-09
6	rs9275187	32656038	C	ADD	122844	1.164	6.456	1.07E-10
6	rs9275188	32656136	T	ADD	137207	1.148	6.272	3.57E-10
6	rs9275189	32656137	G	ADD	137207	1.148	6.272	3.57E-10
6	rs9275199	32656680	T	ADD	145950	1.146	6.407	1.48E-10
6	rs2856676	32656833	A	ADD	148625	1.141	6.234	4.56E-10
6	rs9275202	32656869	G	ADD	148107	1.145	6.386	1.70E-10
6	rs2647000	32656890	C	ADD	148110	1.144	6.383	1.73E-10

6	rs4642516	32657543	T	ADD	148783	1.142	6.286	3.26E-10
6	rs7754464	32657818	G	ADD	148730	1.142	6.275	3.50E-10
6	rs7754885	32657858	A	ADD	148646	1.14	6.209	5.34E-10
6	rs2858330	32658715	C	ADD	148532	1.141	6.247	4.18E-10
6	rs9275216	32658748	T	ADD	148321	1.137	5.846	5.04E-09
6	rs2858329	32658801	G	ADD	148172	1.14	6.193	5.89E-10
6	rs9275218	32658933	G	ADD	148043	1.14	5.939	2.87E-09
6	rs62404159	32661150	G	ADD	145652	0.6263	-7.287	3.18E-13
6	rs9275272	32662546	C	ADD	148039	1.151	6.479	9.25E-11
6	rs9275273	32662559	A	ADD	148368	1.15	6.446	1.15E-10
6	rs9275274	32662566	C	ADD	148196	1.149	6.405	1.50E-10
6	rs9275289	32663208	C	ADD	138900	1.156	6.294	3.10E-10
6	rs9275291	32663260	A	ADD	138466	1.153	6.146	7.94E-10
6	rs9275297	32663446	A	ADD	148433	1.152	6.502	7.94E-11
6	rs5000634	32663564	G	ADD	148759	1.15	6.437	1.22E-10
6	rs6457619	32663986	G	ADD	148773	1.15	6.438	1.21E-10
6	rs6457622	32664163	C	ADD	148777	1.15	6.436	1.23E-10
6	rs7745040	32664332	C	ADD	148783	1.15	6.432	1.26E-10
6	rs2894380	32664675	G	ADD	148757	1.149	6.417	1.39E-10
6	rs2856667	32665079	C	ADD	148667	1.142	6.274	3.52E-10
6	rs62406889	32672214	T	ADD	147694	0.6342	-7.737	1.02E-14
6	rs17219281	32675645	A	ADD	148683	1.208	5.757	8.55E-09
6	rs7764819	32680576	G	ADD	148783	1.213	6.345	2.22E-10
6	rs7765379	32680928	G	ADD	148783	1.212	6.327	2.50E-10
6	rs17212937	32681339	T	ADD	148783	1.211	6.297	3.03E-10
6	rs3873444	32682724	T	ADD	148783	0.7491	-7.076	1.49E-12
6	rs9275652	32685822	C	ADD	148783	0.851	-6.074	1.25E-09
6	rs9275655	32685918	G	ADD	148691	0.8493	-6.141	8.21E-10
6	rs9275657	32685967	A	ADD	148706	0.849	-6.152	7.65E-10
6	rs9275659	32686103	A	ADD	148783	0.8488	-6.162	7.19E-10
6	rs9275660	32686196	A	ADD	148783	0.8506	-6.089	1.14E-09
6	rs9275661	32686248	C	ADD	148783	0.8488	-6.162	7.19E-10
6	rs9275663	32686380	G	ADD	148783	0.8488	-6.162	7.19E-10
6	rs9275666	32686627	T	ADD	148629	0.8473	-6.22	4.96E-10
6	rs9275669	32686676	A	ADD	148628	0.8473	-6.22	4.96E-10
6	rs9275674	32686806	T	ADD	148775	0.8485	-6.175	6.62E-10
6	rs9275680	32687218	T	ADD	148491	0.8412	-6.571	5.00E-11
6	rs9275682	32687297	G	ADD	148775	0.8485	-6.174	6.65E-10

6	rs9275686	32687570	A	ADD	148783	0.8501	-6.087	1.15E-09
6	rs150845531	32687609	AT	ADD	148779	0.8483	-6.182	6.33E-10
6	rs9275690	32687720	A	ADD	148783	0.8483	-6.182	6.31E-10
6	rs9275696	32687904	G	ADD	148782	0.8483	-6.183	6.30E-10
6	rs9275699	32688021	G	ADD	148772	0.848	-6.197	5.77E-10
6	rs9275702	32688100	T	ADD	148702	0.8472	-6.227	4.75E-10
6	rs9275711	32688228	C	ADD	148046	0.8492	-6.144	8.05E-10
6	rs9275723	32688483	A	ADD	148782	0.8483	-6.183	6.30E-10
6	rs9275728	32688638	C	ADD	148781	0.8483	-6.183	6.29E-10
6	rs9275731	32688752	T	ADD	148590	0.8464	-6.252	4.06E-10
6	rs9275735	32688787	A	ADD	148779	0.8483	-6.183	6.29E-10
6	rs9275740	32688859	C	ADD	148563	0.8489	-6.15	7.76E-10
6	rs9275741	32688872	A	ADD	148326	0.8487	-6.142	8.17E-10
6	rs9275764	32689289	T	ADD	148781	0.8483	-6.183	6.29E-10
6	rs9275769	32689431	C	ADD	148090	0.8466	-6.215	5.14E-10
6	rs9275770	32689432	A	ADD	148700	0.8482	-6.185	6.20E-10
6	rs201794780	32689730	T	ADD	119522	1.152	5.905	3.52E-09
6	rs9275791	32689938	A	ADD	147440	0.8489	-6.085	1.16E-09
6	rs9275792	32689946	A	ADD	148724	0.8481	-6.187	6.11E-10
6	rs9275796	32690078	C	ADD	148737	0.8485	-6.175	6.63E-10
6	rs9275806	32690320	C	ADD	148075	0.8452	-6.28	3.39E-10
6	rs9275807	32690332	T	ADD	147990	0.8454	-6.27	3.62E-10
6	rs9275829	32690784	C	ADD	147339	0.8546	-5.856	4.75E-09
6	rs9275848	32691173	T	ADD	148159	0.8502	-6.093	1.11E-09
6	rs9275858	32691345	G	ADD	148336	0.8488	-6.158	7.35E-10
6	rs763026	32691745	T	ADD	148770	0.8481	-6.19	6.01E-10
6	rs763025	32691937	G	ADD	148770	0.8481	-6.19	6.01E-10
6	rs9275899	32692094	A	ADD	148702	0.848	-6.192	5.94E-10
6	rs9275911	32692391	G	ADD	148704	0.848	-6.194	5.88E-10
6	rs9275936	32692933	A	ADD	148783	0.8519	-6.031	1.63E-09
6	rs9275945	32693085	G	ADD	148469	0.8471	-6.214	5.15E-10
6	rs9275957	32693294	A	ADD	147846	0.8531	-5.93	3.03E-09
6	rs9275965	32693482	A	ADD	148777	0.8484	-6.178	6.49E-10
6	rs9275970	32693615	C	ADD	147949	0.852	-5.97	2.37E-09
6	rs9275986	32694068	G	ADD	148778	0.8485	-6.176	6.58E-10
6	rs9275988	32694169	A	ADD	147641	0.8564	-5.795	6.81E-09
6	rs9275989	32694211	A	ADD	148072	0.8458	-6.252	4.04E-10
6	rs9275996	32694401	C	ADD	148617	0.8459	-6.28	3.39E-10

6	6:32694936_CT AAT_C	32694936	C	ADD	148306	0.8493	-6.129	8.82E-10
6	rs9276021	32695212	C	ADD	148776	0.8485	-6.177	6.54E-10
6	rs9276039	32695505	T	ADD	148776	0.8484	-6.178	6.49E-10
6	rs9276054	32695798	C	ADD	148215	0.845	-6.291	3.16E-10
6	rs9276055	32695818	T	ADD	148287	0.8458	-6.264	3.75E-10
6	rs9276074	32696443	A	ADD	148770	0.8484	-6.178	6.48E-10
6	rs9276075	32696543	A	ADD	144729	0.848	-5.949	2.69E-09
6	rs9276098	32696894	G	ADD	148771	0.8484	-6.178	6.50E-10
6	6:32696963_CT _C	32696963	C	ADD	148611	0.8479	-6.197	5.77E-10
6	rs9276106	32697037	T	ADD	148768	0.8484	-6.179	6.44E-10
6	rs9276111	32697160	C	ADD	148656	0.8485	-6.172	6.74E-10
6	rs9276127	32697606	C	ADD	148766	0.8484	-6.179	6.45E-10
6	rs9276132	32697755	G	ADD	148395	0.8515	-6.03	1.64E-09
6	rs9276138	32697893	T	ADD	148768	0.8472	-6.228	4.73E-10
6	rs9276149	32698156	T	ADD	148769	0.8472	-6.228	4.71E-10
6	6:32698360_CT CTCAGTGT_C	32698360	C	ADD	148586	0.8483	-6.179	6.47E-10
6	rs9276164	32698468	T	ADD	148771	0.8472	-6.227	4.75E-10
6	rs9276168	32698790	G	ADD	148773	0.8472	-6.228	4.72E-10
6	rs9276171	32698918	G	ADD	148741	0.8443	-6.386	1.71E-10
6	rs9276172	32698947	C	ADD	148768	0.847	-6.239	4.39E-10
6	rs112350825	32699144	C	ADD	108617	0.8469	-6.173	6.69E-10
6	rs9276188	32699262	C	ADD	148782	0.8471	-6.232	4.61E-10
6	rs150842907	32699274	A T	ADD	148390	0.8439	-6.347	2.19E-10
6	rs9276190	32699350	T	ADD	148782	0.8471	-6.232	4.61E-10
6	rs9276191	32699437	A	ADD	148782	0.8471	-6.232	4.61E-10
6	rs9276198	32699633	A	ADD	148764	0.8475	-6.216	5.11E-10
6	rs9276201	32699662	G	ADD	148780	0.8472	-6.23	4.65E-10
6	rs2858884	32700083	C	ADD	148783	0.8587	-5.788	7.12E-09
6	rs2858883	32700761	C	ADD	148759	0.8756	-6.004	1.92E-09
6	rs9276242	32701189	C	ADD	148779	0.8471	-6.232	4.61E-10
6	rs2859088	32701295	T	ADD	148770	0.8759	-5.986	2.15E-09
6	rs2859087	32701338	G	ADD	148782	0.8586	-5.793	6.90E-09
6	rs9276249	32701372	A	ADD	148778	0.8468	-6.246	4.20E-10
6	rs9276251	32701444	A	ADD	148780	0.8471	-6.232	4.60E-10
6	rs2859081	32702108	G	ADD	148779	0.8586	-5.792	6.96E-09
6	rs2859079	32702381	G	ADD	148782	0.8586	-5.794	6.88E-09
6	rs2859078	32702449	G	ADD	148783	0.8578	-5.832	5.46E-09

6	rs2859076	32702586	A	ADD	148782	0.8586	-5.794	6.88E-09
6	rs9276286	32702722	T	ADD	148742	0.8583	-5.803	6.51E-09
6	rs2859075	32702798	G	ADD	148782	0.8586	-5.794	6.88E-09
6	6:32703231_GT_G	32703231	G	ADD	147879	0.8424	-6.388	1.68E-10
6	rs9276306	32703589	A	ADD	148772	0.847	-6.236	4.48E-10
6	rs6457642	32704362	T	ADD	148073	0.8452	-6.405	1.51E-10
6	rs9276313	32704706	A	ADD	148769	0.8471	-6.233	4.56E-10
6	rs9276314	32704786	T	ADD	148776	0.8468	-6.244	4.25E-10
6	rs9276325	32705210	T	ADD	148779	0.8469	-6.241	4.35E-10
6	rs9276327	32705401	T	ADD	148781	0.8469	-6.241	4.34E-10
6	rs9276339	32705700	A	ADD	148779	0.847	-6.238	4.43E-10
6	6:32705862_AG_A	32705862	A	ADD	148739	0.8439	-6.401	1.54E-10
6	rs9276348	32706209	G	ADD	148777	0.8441	-6.394	1.62E-10
6	rs9276351	32706416	A	ADD	148783	0.844	-6.399	1.56E-10
6	rs9276357	32706559	G	ADD	148783	0.8475	-6.218	5.05E-10
6	rs9276358	32706725	G	ADD	148782	0.8469	-6.241	4.34E-10
6	rs9276361	32706936	A	ADD	148763	0.8437	-6.411	1.45E-10
6	rs9276363	32707023	C	ADD	148416	0.8474	-6.209	5.32E-10
6	rs9276365	32707040	A	ADD	148423	0.8445	-6.365	1.95E-10
6	rs71536152	32707352	AT	ADD	148697	0.8464	-6.259	3.88E-10
6	rs9276375	32707585	A	ADD	148766	0.8469	-6.242	4.33E-10
6	rs5021448	32707706	T	ADD	148767	0.8441	-6.396	1.60E-10
6	rs5021452	32708074	T	ADD	146518	0.8432	-6.339	2.32E-10
6	rs5021453	32708124	G	ADD	146547	0.8666	-6.397	1.59E-10
6	6:32708511_CTG_C	32708511	C	ADD	148689	0.8422	-6.472	9.67E-11
6	rs9276401	32709204	C	ADD	148731	0.8473	-6.224	4.85E-10
6	rs4394270	32709964	C	ADD	148783	0.8775	-5.857	4.71E-09
6	rs4496840	32709990	T	ADD	148769	0.8777	-5.847	5.00E-09
6	rs4403295	32710010	T	ADD	148751	0.8777	-5.845	5.08E-09
6	rs9276425	32711050	T	ADD	148434	0.8487	-6.058	1.38E-09
6	rs9276456	32715943	G	ADD	148247	0.8464	-6.142	8.14E-10
6	rs9276550	32722243	T	ADD	148780	0.8479	-6.092	1.11E-09
6	rs9276555	32722754	A	ADD	148783	0.8489	-6.051	1.44E-09
6	rs9276556	32723233	C	ADD	148783	0.8474	-6.115	9.63E-10
6	rs9276576	32725832	G	ADD	148724	0.8474	-6.108	1.01E-09
6	rs9276584	32730835	T	ADD	148710	0.8488	-6.047	1.47E-09
6	rs9276585	32731313	A	ADD	148711	0.8487	-6.05	1.45E-09

6	rs9276607	32737257	T	ADD	148783	0.8486	-6.055	1.41E-09
6	rs1367728	33034815	A	ADD	148783	0.8069	-5.832	5.47E-09
6	rs2301225	33035860	A	ADD	148672	0.8047	-5.895	3.75E-09
6	rs28986425	33039535	T	ADD	148716	0.8055	-5.865	4.50E-09
6	6:33041188_AATTGATGTGAACCACCCC_A	33041188	A	ADD	148016	0.8067	-5.812	6.16E-09
6	rs6934289	33044956	C	ADD	148550	0.8053	-5.89	3.87E-09
6	rs14362	33052981	A	ADD	148316	0.8088	-5.752	8.81E-09
6	rs2179918	33062016	C	ADD	148734	0.806	-5.864	4.53E-09
6	rs3117245	33069567	A	ADD	148678	0.8053	-5.881	4.09E-09
6	rs35701899	33079450	C	ADD	148502	0.8092	-5.757	8.57E-09

Supplement Table 8. Summary of LD clumping of discovery cohort.

CHR	RSID	BP	P	TOTAL	NSIG	S05	S01	S001	S0001
2	2:113992800_TCC_T	113992800	8.76E-10	225	90	44	18	5	68
5	rs27069	1347128	2.51E-09	136	21	8	19	10	78
6	rs9272050	32599071	2.51E-28	6681	643	601	469	388	4580
6	rs2523496	31377719	1.74E-20	2651	818	329	315	220	969
6	rs55986091	32623713	6.42E-22	1927	118	190	133	306	1180
6	rs9274715	32637391	1.01E-12	5691	2072	573	673	446	1927
6	rs2523545	31333499	1.82E-12	2086	235	340	157	424	930
6	rs144847276	31335416	6.33E-11	306	151	27	31	28	69
6	rs2070600	32151443	3.98E-10	895	211	306	125	139	114
6	rs113547322	32206619	1.20E-09	1064	19	23	23	96	903
6	6:31530636_CAA_C	31530636	2.96E-09	620	309	45	150	39	77
6	rs2301225	33035860	3.75E-09	317	99	13	54	10	141
6	rs116071718	30990081	9.06E-09	452	73	67	201	99	12

TOTAL   Total number of other SNPs in clump (pass >1000kb and >0.9
NSIG   Number of clumped SNPs that are not significant ($p > 0.05$)
S05   Number of clumped SNPs $0.01 < p < 0.05$
S01   Number of clumped SNPs $0.001 < p < 0.01$
S001   Number of clumped SNPs $0.0001 < p < 0.001$
S0001   Number of clumped SNPs $p < 0.0001$

	A	B	C	D	E	F	G	H
1	**Supplement Table 9. GWAS catalog search of all previously reported loci for cervical cancer phenotype**							
2								
3	**DATE ADDED**	**PUBMEDID**	**FIRST AUTH**	**DATE**	**JOURNAL**	**LINK**	**STUDY**	**DISEASE/TRAIT**
4	01/07/2013	23482656	Chen D	12/03/2013	J Natl Cancer Inst	www.ncbi.nlm.nih.gov/pubmed/23482656	Genome-wide association study of susceptibility loci for cervical cancer.	Cervical cancer
5	01/07/2013	23482656	Chen D	12/03/2013	J Natl Cancer Inst	www.ncbi.nlm.nih.gov/pubmed/23482656	Genome-wide association study of susceptibility loci for cervical cancer.	Cervical cancer
6	01/07/2013	23482656	Chen D	12/03/2013	J Natl Cancer Inst	www.ncbi.nlm.nih.gov/pubmed/23482656	Genome-wide association study of susceptibility loci for cervical cancer.	Cervical cancer
7	01/07/2013	23482656	Chen D	12/03/2013	J Natl Cancer Inst	www.ncbi.nlm.nih.gov/pubmed/23482656	Genome-wide association study of susceptibility loci for cervical cancer.	Cervical cancer
8	01/07/2013	23482656	Chen D	12/03/2013	J Natl Cancer Inst	www.ncbi.nlm.nih.gov/pubmed/23482656	Genome-wide association study of susceptibility loci for cervical cancer.	Cervical cancer
9	01/07/2013	23482656	Chen D	12/03/2013	J Natl Cancer Inst	www.ncbi.nlm.nih.gov/pubmed/23482656	Genome-wide association study of susceptibility loci for cervical cancer.	Cervical cancer
10	08/11/2014	24700089	Miura K	04/04/2014	J Med Virol	www.ncbi.nlm.nih.gov/pubmed/24700089	Genome-wide association study of HPV-associated cervical cancer in Japanese women.	Cervical cancer
11	08/11/2014	24700089	Miura K	04/04/2014	J Med Virol	www.ncbi.nlm.nih.gov/pubmed/24700089	Genome-wide association study of HPV-associated cervical cancer in Japanese women.	Cervical cancer
12	08/11/2014	24700089	Miura K	04/04/2014	J Med Virol	www.ncbi.nlm.nih.gov/pubmed/24700089	Genome-wide association study of HPV-associated cervical cancer in Japanese women.	Cervical cancer
13	08/11/2014	24700089	Miura K	04/04/2014	J Med Virol	www.ncbi.nlm.nih.gov/pubmed/24700089	Genome-wide association study of HPV-associated cervical cancer in Japanese women.	Cervical cancer
14	08/11/2014	24700089	Miura K	04/04/2014	J Med Virol	www.ncbi.nlm.nih.gov/pubmed/24700089	Genome-wide association study of HPV-associated cervical cancer in Japanese women.	Cervical cancer
15	08/11/2014	24700089	Miura K	04/04/2014	J Med Virol	www.ncbi.nlm.nih.gov/pubmed/24700089	Genome-wide association study of HPV-associated cervical cancer in Japanese women.	Cervical cancer
16	08/11/2014	24700089	Miura K	04/04/2014	J Med Virol	www.ncbi.nlm.nih.gov/pubmed/24700089	Genome-wide association study of HPV-associated cervical cancer in Japanese women.	Cervical cancer
17	08/11/2014	24700089	Miura K	04/04/2014	J Med Virol	www.ncbi.nlm.nih.gov/pubmed/24700089	Genome-wide association study of HPV-associated cervical cancer in Japanese women.	Cervical cancer
18	08/11/2014	24700089	Miura K	04/04/2014	J Med Virol	www.ncbi.nlm.nih.gov/pubmed/24700089	Genome-wide association study of HPV-associated cervical cancer in Japanese women.	Cervical cancer
19	16/04/2020	31488892	Masuda T	05/09/2019	Eur J Hum Genet	www.ncbi.nlm.nih.gov/pubmed/31488892	GWAS of five gynecologic diseases and cross-trait analysis in Japanese.	Cervical cancer (multi-trait analysis)
20	16/04/2020	31488892	Masuda T	05/09/2019	Eur J Hum Genet	www.ncbi.nlm.nih.gov/pubmed/31488892	GWAS of five gynecologic diseases and cross-trait analysis in Japanese.	Cervical cancer (multi-trait analysis)
21	16/04/2020	31488892	Masuda T	05/09/2019	Eur J Hum Genet	www.ncbi.nlm.nih.gov/pubmed/31488892	GWAS of five gynecologic diseases and cross-trait analysis in Japanese.	Cervical cancer (multi-trait analysis)
22	16/04/2020	31488892	Masuda T	05/09/2019	Eur J Hum Genet	www.ncbi.nlm.nih.gov/pubmed/31488892	GWAS of five gynecologic diseases and cross-trait analysis in Japanese.	Cervical cancer (multi-trait analysis)
23	16/04/2020	31488892	Masuda T	05/09/2019	Eur J Hum Genet	www.ncbi.nlm.nih.gov/pubmed/31488892	GWAS of five gynecologic diseases and cross-trait analysis in Japanese.	Cervical cancer (multi-trait analysis)
24	16/04/2020	31488892	Masuda T	05/09/2019	Eur J Hum Genet	www.ncbi.nlm.nih.gov/pubmed/31488892	GWAS of five gynecologic diseases and cross-trait analysis in Japanese.	Cervical cancer (multi-trait analysis)
25	16/04/2020	31488892	Masuda T	05/09/2019	Eur J Hum Genet	www.ncbi.nlm.nih.gov/pubmed/31488892	GWAS of five gynecologic diseases and cross-trait analysis in Japanese.	Cervical cancer (multi-trait analysis)
26	16/04/2020	31488892	Masuda T	05/09/2019	Eur J Hum Genet	www.ncbi.nlm.nih.gov/pubmed/31488892	GWAS of five gynecologic diseases and cross-trait analysis in Japanese.	Cervical cancer (multi-trait analysis)
27	16/04/2020	31488892	Masuda T	05/09/2019	Eur J Hum Genet	www.ncbi.nlm.nih.gov/pubmed/31488892	GWAS of five gynecologic diseases and cross-trait analysis in Japanese.	Cervical cancer (multi-trait analysis)
28	16/04/2020	31488892	Masuda T	05/09/2019	Eur J Hum Genet	www.ncbi.nlm.nih.gov/pubmed/31488892	GWAS of five gynecologic diseases and cross-trait analysis in Japanese.	Cervical cancer (multi-trait analysis)
29	16/04/2020	31488892	Masuda T	05/09/2019	Eur J Hum Genet	www.ncbi.nlm.nih.gov/pubmed/31488892	GWAS of five gynecologic diseases and cross-trait analysis in Japanese.	Cervical cancer (multi-trait analysis)
30	16/04/2020	31488892	Masuda T	05/09/2019	Eur J Hum Genet	www.ncbi.nlm.nih.gov/pubmed/31488892	GWAS of five gynecologic diseases and cross-trait analysis in Japanese.	Cervical cancer (multi-trait analysis)
31	16/04/2020	31488892	Masuda T	05/09/2019	Eur J Hum Genet	www.ncbi.nlm.nih.gov/pubmed/31488892	GWAS of five gynecologic diseases and cross-trait analysis in Japanese.	Cervical cancer (multi-trait analysis)
32	16/04/2020	31488892	Masuda T	05/09/2019	Eur J Hum Genet	www.ncbi.nlm.nih.gov/pubmed/31488892	GWAS of five gynecologic diseases and cross-trait analysis in Japanese.	Cervical cancer (multi-trait analysis)
33	16/04/2020	31488892	Masuda T	05/09/2019	Eur J Hum Genet	www.ncbi.nlm.nih.gov/pubmed/31488892	GWAS of five gynecologic diseases and cross-trait analysis in Japanese.	Cervical cancer (multi-trait analysis)
34	16/04/2020	31488892	Masuda T	05/09/2019	Eur J Hum Genet	www.ncbi.nlm.nih.gov/pubmed/31488892	GWAS of five gynecologic diseases and cross-trait analysis in Japanese.	Cervical cancer (multi-trait analysis)
35	16/04/2020	31488892	Masuda T	05/09/2019	Eur J Hum Genet	www.ncbi.nlm.nih.gov/pubmed/31488892	GWAS of five gynecologic diseases and cross-trait analysis in Japanese.	Cervical cancer (multi-trait analysis)
36	16/04/2020	31488892	Masuda T	05/09/2019	Eur J Hum Genet	www.ncbi.nlm.nih.gov/pubmed/31488892	GWAS of five gynecologic diseases and cross-trait analysis in Japanese.	Cervical cancer (multi-trait analysis)
37	16/04/2020	31488892	Masuda T	05/09/2019	Eur J Hum Genet	www.ncbi.nlm.nih.gov/pubmed/31488892	GWAS of five gynecologic diseases and cross-trait analysis in Japanese.	Cervical cancer (multi-trait analysis)
38	16/04/2020	31488892	Masuda T	05/09/2019	Eur J Hum Genet	www.ncbi.nlm.nih.gov/pubmed/31488892	GWAS of five gynecologic diseases and cross-trait analysis in Japanese.	Cervical cancer (multi-trait analysis)
39	16/04/2020	31488892	Masuda T	05/09/2019	Eur J Hum Genet	www.ncbi.nlm.nih.gov/pubmed/31488892	GWAS of five gynecologic diseases and cross-trait analysis in Japanese.	Cervical cancer (multi-trait analysis)
40	16/04/2020	31488892	Masuda T	05/09/2019	Eur J Hum Genet	www.ncbi.nlm.nih.gov/pubmed/31488892	GWAS of five gynecologic diseases and cross-trait analysis in Japanese.	Cervical cancer (multi-trait analysis)
41	16/04/2020	31488892	Masuda T	05/09/2019	Eur J Hum Genet	www.ncbi.nlm.nih.gov/pubmed/31488892	GWAS of five gynecologic diseases and cross-trait analysis in Japanese.	Cervical cancer (multi-trait analysis)
42	16/04/2020	31488892	Masuda T	05/09/2019	Eur J Hum Genet	www.ncbi.nlm.nih.gov/pubmed/31488892	GWAS of five gynecologic diseases and cross-trait analysis in Japanese.	Cervical cancer (multi-trait analysis)
43	16/04/2020	31488892	Masuda T	05/09/2019	Eur J Hum Genet	www.ncbi.nlm.nih.gov/pubmed/31488892	GWAS of five gynecologic diseases and cross-trait analysis in Japanese.	Cervical cancer (multi-trait analysis)
44	16/04/2020	31488892	Masuda T	05/09/2019	Eur J Hum Genet	www.ncbi.nlm.nih.gov/pubmed/31488892	GWAS of five gynecologic diseases and cross-trait analysis in Japanese.	Cervical cancer (multi-trait analysis)
45	16/04/2020	31488892	Masuda T	05/09/2019	Eur J Hum Genet	www.ncbi.nlm.nih.gov/pubmed/31488892	GWAS of five gynecologic diseases and cross-trait analysis in Japanese.	Cervical cancer (multi-trait analysis)
46	16/04/2020	31488892	Masuda T	05/09/2019	Eur J Hum Genet	www.ncbi.nlm.nih.gov/pubmed/31488892	GWAS of five gynecologic diseases and cross-trait analysis in Japanese.	Cervical cancer (multi-trait analysis)
47	16/04/2020	31488892	Masuda T	05/09/2019	Eur J Hum Genet	www.ncbi.nlm.nih.gov/pubmed/31488892	GWAS of five gynecologic diseases and cross-trait analysis in Japanese.	Cervical cancer (multi-trait analysis)
48	16/04/2020	31488892	Masuda T	05/09/2019	Eur J Hum Genet	www.ncbi.nlm.nih.gov/pubmed/31488892	GWAS of five gynecologic diseases and cross-trait analysis in Japanese.	Cervical cancer (multi-trait analysis)
49	16/04/2020	31488892	Masuda T	05/09/2019	Eur J Hum Genet	www.ncbi.nlm.nih.gov/pubmed/31488892	GWAS of five gynecologic diseases and cross-trait analysis in Japanese.	Cervical cancer (multi-trait analysis)
50	16/04/2020	31488892	Masuda T	05/09/2019	Eur J Hum Genet	www.ncbi.nlm.nih.gov/pubmed/31488892	GWAS of five gynecologic diseases and cross-trait analysis in Japanese.	Cervical cancer (multi-trait analysis)
51	16/04/2020	31488892	Masuda T	05/09/2019	Eur J Hum Genet	www.ncbi.nlm.nih.gov/pubmed/31488892	GWAS of five gynecologic diseases and cross-trait analysis in Japanese.	Cervical cancer (multi-trait analysis)
52	16/04/2020	31488892	Masuda T	05/09/2019	Eur J Hum Genet	www.ncbi.nlm.nih.gov/pubmed/31488892	GWAS of five gynecologic diseases and cross-trait analysis in Japanese.	Cervical cancer (multi-trait analysis)
53	16/04/2020	31488892	Masuda T	05/09/2019	Eur J Hum Genet	www.ncbi.nlm.nih.gov/pubmed/31488892	GWAS of five gynecologic diseases and cross-trait analysis in Japanese.	Cervical cancer (multi-trait analysis)
54	16/04/2020	31488892	Masuda T	05/09/2019	Eur J Hum Genet	www.ncbi.nlm.nih.gov/pubmed/31488892	GWAS of five gynecologic diseases and cross-trait analysis in Japanese.	Cervical cancer (multi-trait analysis)
55	16/04/2020	31488892	Masuda T	05/09/2019	Eur J Hum Genet	www.ncbi.nlm.nih.gov/pubmed/31488892	GWAS of five gynecologic diseases and cross-trait analysis in Japanese.	Cervical cancer (multi-trait analysis)
56	16/04/2020	31488892	Masuda T	05/09/2019	Eur J Hum Genet	www.ncbi.nlm.nih.gov/pubmed/31488892	GWAS of five gynecologic diseases and cross-trait analysis in Japanese.	Cervical cancer (multi-trait analysis)
57	16/04/2020	31488892	Masuda T	05/09/2019	Eur J Hum Genet	www.ncbi.nlm.nih.gov/pubmed/31488892	GWAS of five gynecologic diseases and cross-trait analysis in Japanese.	Cervical cancer
58	16/04/2020	31488892	Masuda T	05/09/2019	Eur J Hum Genet	www.ncbi.nlm.nih.gov/pubmed/31488892	GWAS of five gynecologic diseases and cross-trait analysis in Japanese.	Cervical cancer
59	16/04/2020	31488892	Masuda T	05/09/2019	Eur J Hum Genet	www.ncbi.nlm.nih.gov/pubmed/31488892	GWAS of five gynecologic diseases and cross-trait analysis in Japanese.	Cervical cancer
60	16/04/2020	31488892	Masuda T	05/09/2019	Eur J Hum Genet	www.ncbi.nlm.nih.gov/pubmed/31488892	GWAS of five gynecologic diseases and cross-trait analysis in Japanese.	Cervical cancer
61	16/04/2020	31488892	Masuda T	05/09/2019	Eur J Hum Genet	www.ncbi.nlm.nih.gov/pubmed/31488892	GWAS of five gynecologic diseases and cross-trait analysis in Japanese.	Cervical cancer
62	16/04/2020	31488892	Masuda T	05/09/2019	Eur J Hum Genet	www.ncbi.nlm.nih.gov/pubmed/31488892	GWAS of five gynecologic diseases and cross-trait analysis in Japanese.	Cervical cancer
63	16/04/2020	31488892	Masuda T	05/09/2019	Eur J Hum Genet	www.ncbi.nlm.nih.gov/pubmed/31488892	GWAS of five gynecologic diseases and cross-trait analysis in Japanese.	Cervical cancer

	I	J	K	L	M	N
1						
2						
3	**INITIAL SAMPLE SIZE**	**REPLICATION SAMPLE SIZE**	**REGION**	**CHR_ID**	**CHR_POS**	**REPORTED GENE(S)**
4	1,034 European ancestry cases, 3,948 European ancestry controls	1,140 European ancestry cases, 1,058 European ancestry controls				HLA
5	1,034 European ancestry cases, 3,948 European ancestry controls	1,140 European ancestry cases, 1,058 European ancestry controls				HLA
6	1,034 European ancestry cases, 3,948 European ancestry controls	1,140 European ancestry cases, 1,058 European ancestry controls	6p21.33	6	31422633	MICA
7	1,034 European ancestry cases, 3,948 European ancestry controls	1,140 European ancestry cases, 1,058 European ancestry controls	6p21.32	6	32633026	HLA-DRB1, HLA-DQA1
8	1,034 European ancestry cases, 3,948 European ancestry controls	1,140 European ancestry cases, 1,058 European ancestry controls	6p21.32	6	33121846	HLA-DPB2
9	1,034 European ancestry cases, 3,948 European ancestry controls	1,140 European ancestry cases, 1,058 European ancestry controls				HLA-B
10	226 Japanese ancestry cases, 186 Japanese ancestry controls	NA	16p13.2	16	9232128	intergenic
11	226 Japanese ancestry cases, 186 Japanese ancestry controls	NA	8p21.3	8	20734998	intergenic
12	226 Japanese ancestry cases, 186 Japanese ancestry controls	NA	8p23.3	8	2021565	intergenic
13	226 Japanese ancestry cases, 186 Japanese ancestry controls	NA	7q21.13	7	88970471	ZNF804B
14	226 Japanese ancestry cases, 186 Japanese ancestry controls	NA	2q36.3	2	227644742	intergenic
15	226 Japanese ancestry cases, 186 Japanese ancestry controls	NA	7q11.23	7	77433395	intergenic
16	226 Japanese ancestry cases, 186 Japanese ancestry controls	NA	2q14.2	2	118131653	intergenic
17	226 Japanese ancestry cases, 186 Japanese ancestry controls	NA	18q22.3	18	72259522	intergenic
18	226 Japanese ancestry cases, 186 Japanese ancestry controls	NA	1q23.3	1	160773959	intergenic
19	538 Japanese ancestry cases, 39,556 Japanese ancestry controls	NA	12q24.23	12	119604245	Intergenic
20	538 Japanese ancestry cases, 39,556 Japanese ancestry controls	NA	13q12.11	13	22100187	Intergenic
21	538 Japanese ancestry cases, 39,556 Japanese ancestry controls	NA	13q33.3	13	106422664	Intergenic
22	538 Japanese ancestry cases, 39,556 Japanese ancestry controls	NA	14q11.2	14	20259308	Intergenic
23	538 Japanese ancestry cases, 39,556 Japanese ancestry controls	NA	14q11.2	14	20368118	Intergenic
24	538 Japanese ancestry cases, 39,556 Japanese ancestry controls	NA	14q21.1	14	41755191	Intergenic
25	538 Japanese ancestry cases, 39,556 Japanese ancestry controls	NA	14q32.31	14	101059844	Intergenic
26	538 Japanese ancestry cases, 39,556 Japanese ancestry controls	NA	16p13.3	16	6687493	Intergenic
27	538 Japanese ancestry cases, 39,556 Japanese ancestry controls	NA	17q24.3	17	72101710	Intergenic
28	538 Japanese ancestry cases, 39,556 Japanese ancestry controls	NA	19q13.32	19	45666590	Intergenic
29	538 Japanese ancestry cases, 39,556 Japanese ancestry controls	NA	19q13.41	19	51734303	Intergenic
30	538 Japanese ancestry cases, 39,556 Japanese ancestry controls	NA	20p13	20	4817030	Intergenic
31	538 Japanese ancestry cases, 39,556 Japanese ancestry controls	NA	20q13.33	20	64106159	Intergenic
32	538 Japanese ancestry cases, 39,556 Japanese ancestry controls	NA	6p22.1	6	29829266	Intergenic
33	538 Japanese ancestry cases, 39,556 Japanese ancestry controls	NA	6p22.1	6	30075223	Intergenic
34	538 Japanese ancestry cases, 39,556 Japanese ancestry controls	NA	6p21.33	6	30830500	Intergenic
35	538 Japanese ancestry cases, 39,556 Japanese ancestry controls	NA	6p21.33	6	30972610	Intergenic
36	538 Japanese ancestry cases, 39,556 Japanese ancestry controls	NA	6p21.33	6	31245753	Intergenic
37	538 Japanese ancestry cases, 39,556 Japanese ancestry controls	NA	6p21.33	6	31330644	Intergenic
38	538 Japanese ancestry cases, 39,556 Japanese ancestry controls	NA	6p21.33	6	31762974	Intergenic
39	538 Japanese ancestry cases, 39,556 Japanese ancestry controls	NA	6p21.33	6	31972441	Intergenic
40	538 Japanese ancestry cases, 39,556 Japanese ancestry controls	NA	6p21.33	6	32093562	Intergenic
41	538 Japanese ancestry cases, 39,556 Japanese ancestry controls	NA	6p21.32	6	32241674	Intergenic
42	538 Japanese ancestry cases, 39,556 Japanese ancestry controls	NA	6p21.32	6	32609610	Intergenic
43	538 Japanese ancestry cases, 39,556 Japanese ancestry controls	NA	6p21.32	6	32714813	Intergenic
44	538 Japanese ancestry cases, 39,556 Japanese ancestry controls	NA	6p21.32	6	32920459	Intergenic
45	538 Japanese ancestry cases, 39,556 Japanese ancestry controls	NA	6p21.32	6	33107666	Intergenic
46	538 Japanese ancestry cases, 39,556 Japanese ancestry controls	NA	6q22.31	6	122773922	Intergenic
47	538 Japanese ancestry cases, 39,556 Japanese ancestry controls	NA	7q22.3	7	105691372	Intergenic
48	538 Japanese ancestry cases, 39,556 Japanese ancestry controls	NA	7q33	7	133504366	Intergenic
49	538 Japanese ancestry cases, 39,556 Japanese ancestry controls	NA	7q33	7	133732785	Intergenic
50	538 Japanese ancestry cases, 39,556 Japanese ancestry controls	NA	8p23.1	8	9854826	Intergenic
51	538 Japanese ancestry cases, 39,556 Japanese ancestry controls	NA	8q12.1	8	56298047	Intergenic
52	538 Japanese ancestry cases, 39,556 Japanese ancestry controls	NA	9q21.2	9	76775287	Intergenic
53	538 Japanese ancestry cases, 39,556 Japanese ancestry controls	NA	9q32	9	112272205	Intergenic
54	538 Japanese ancestry cases, 39,556 Japanese ancestry controls	NA	10p13	10	17199011	Intergenic
55	538 Japanese ancestry cases, 39,556 Japanese ancestry controls	NA	11p15.5	11	2158112	Intergenic
56	538 Japanese ancestry cases, 39,556 Japanese ancestry controls	NA	12p11.22	12	29599423	Intergenic
57	538 Japanese ancestry cases, 39,556 Japanese ancestry controls	NA	6p22.1	6	27811880	Intergenic
58	538 Japanese ancestry cases, 39,556 Japanese ancestry controls	NA	6p22.1	6	28137256	Intergenic
59	538 Japanese ancestry cases, 39,556 Japanese ancestry controls	NA	6p22.1	6	28356161	Intergenic
60	538 Japanese ancestry cases, 39,556 Japanese ancestry controls	NA	6p22.1	6	28467717	Intergenic
61	538 Japanese ancestry cases, 39,556 Japanese ancestry controls	NA	6p22.1	6	28639807	Intergenic
62	538 Japanese ancestry cases, 39,556 Japanese ancestry controls	NA	6p22.1	6	28800350	Intergenic
63	538 Japanese ancestry cases, 39,556 Japanese ancestry controls	NA	6p22.1	6	29016978	Intergenic

	O	P	Q
1			
2			
3	**MAPPED_GENE**	**UPSTREAM_GENE_II**	**DOWNSTREAM_GE**
4			
5			
6	AL645933.5		
7	HLA-DQA1, HLA-DQA1, HLA-DQA1, HLA-DQA1, HLA-DQA1		
8	HLA-DPB2, HLA-DPB2, HLA-DPB2, HLA-DPB2, HLA-DPB2, HLA-DPB2, HLA-DPB2		
9			
10	RPL21P119 - LINC02177	ENSG00000220793	ENSG00000261617
11	AC018541.1 - TMEM97P2	ENSG00000253300	ENSG00000253866
12	KBTBD11 - AC245164.1	ENSG00000273645	ENSG00000253805
13	ZNF804B		
14	C2orf83		
15	AC004921.1 - GCNT1P5	ENSG00000273341	ENSG00000237620
16	INSIG2 - THORLNC	ENSG00000125629	ENSG00000226856
17	AC069114.1 - CBLN2	ENSG00000265995	ENSG00000141668
18	SLAMF7 - AL121985.3	ENSG00000026751	ENSG00000275801
19	TMEM233, AC002070.1		
20	AL136962.1		
21	LINC00460 - RPL35P9	ENSG00000233532	ENSG00000174418
22	TTC5		
23	TEP1		
24	LRFN5		
25	MIR154 - MIR496	ENSG00000207978	ENSG00000207961
26	RBFOX1		
27	LINC02097, SOX9-AS1		
28	RN7SL836P - GIPR	ENSG00000241226	ENSG00000010310
29	HAS1 - FPR1	ENSG00000105509	ENSG00000171051
30	RASSF2		
31	NPBWR2, MYT1		
32	HLA-G, HLA-G, HLA-G, HLA-G, HLA-G, HLA-G, HLA-G, HLA-G		
33	RNF39, RNF39, RNF39, RNF39, RNF39, RNF39, RNF39, RNF39		
34	LINC00243		
35	NAPGP2 - MUC21	ENSG00000275906	ENSG00000231350
36	AL662844.2 - HLA-C	ENSG00000255899	ENSG00000228299
37	HLA-B, HLA-B, HLA-B, HLA-B, HLA-B, HLA-B		
38	MSH5-SAPCD1, MSH5-SAPCD1, MSH5-SAPCD1, MSH5-SAPCD1, MSH5-SAPCD1, MSH5-SAPCD1		
39	STK19, STK19, STK19, STK19, STK19, STK19		
40	TNXB, TNXB, TNXB, TNXB, TNXB, TNXB		
41	NOTCH4 - TSBP1-AS1	ENSG00000206312	ENSG00000235135
42	HLA-DRB1 - HLA-DQA1	ENSG00000206240	ENSG00000196735
43	MTCO3P1 - AL662789.1	ENSG00000237145	ENSG00000232080
44	HLA-Z - HLA-DMB	ENSG00000237871	ENSG00000226264
45	COL11A2P1 - HLA-DPB2	ENSG00000230816	ENSG00000225132
46	RN7SL564P - FABP7	ENSG00000240606	ENSG00000164434
47	ATXN7L1		
48	EXOC4		
49	RPS3AP27, EXOC4		
50	TNKS - LINC00599	ENSG00000173273	ENSG00000253230
51	AC107952.1 - SDR16C5	ENSG00000254216	ENSG00000170786
52	PCA3, PRUNE2		
53	PTBP3		
54	TRDMT1		
55	INS-IGF2, AC132217.2		
56	TMTC1		
57	H3C10 - H2AC14	ENSG00000278828	ENSG00000276368
58	AL358933.1, ZSCAN16-AS1		
59	ZKSCAN3, ZSCAN31		
60	OR2E1P - Z98745.2	ENSG00000219262	ENSG00000287804
61	AL121932.2		
62	NOP56P1 - AL662890.1	ENSG00000273323	ENSG00000225173
63	ZNF311 - OR2AD1P	ENSG00000197935	ENSG00000233650

	R	S	T	U	V
1					
2					
3	**SNP_GENE_IDS**	**UPSTREAM_(**	**DOWNSTREAM_**	**STRONGEST SNP-RISK ALLELE**	**SNPS**
4				DRB1*1301-DQA1*0103-DQB1*0603-?	DRB1*1301-D
5				DRB1*1501-DQB1*0602-?	DRB1*1501-D
6	ENSG00000288587			rs2516448-?	rs2516448
7	ENSG00000232062, ENSG00000206305, ENSG00000236418, ENSG00000225890, ENSG00000196735			rs9272143-?	rs9272143
8	ENSG00000168379, ENSG00000226071, ENSG00000234834, ENSG00000224557, ENSG00000225429, ENSG00000223372, ENSG00000225132			rs3117027-?	rs3117027
9				HLA-B*0702-?	HLA-B*0702
10		75247	123460	rs4782151-C	rs4782151
11		34846	199437	rs11985951-T	rs11985951
12		14629	11943	rs6558578-G	rs6558578
13	ENSG00000182348			rs3899697-A	rs3899697
14	ENSG00000042304			rs997363-C	rs997363
15		7951	28063	rs7780883-A	rs7780883
16		20656	475	rs6726538-A	rs6726538
17		314945	277158	rs8088832-C	rs8088832
18		19138	1995	rs12068654-T	rs12068654
19	ENSG00000224982, ENSG00000248636			rs142761815-?	rs142761815
20	ENSG00000276476			rs146343995-?	rs146343995
21		38349	3109	rs141629209-?	rs141629209
22	ENSG00000136319			rs145233920-?	rs145233920
23	ENSG00000129566			rs149049108-?	rs149049108
24	ENSG00000165379			rs138523818-?	rs138523818
25		6	729	rs41286572-?	rs41286572
26	ENSG00000078328			rs72764914-?	rs72764914
27	ENSG00000226101, ENSG00000234899			rs140991990-?	rs140991990
28		14785	1631	rs56182403-?	rs56182403
29		10312	10869	rs75724373-?	rs75724373
30	ENSG00000101265			rs201524967-?	rs201524967
31	ENSG00000125522, ENSG00000196132			rs75874947-?	rs75874947
32	ENSG00000206506, ENSG00000233095, ENSG00000237216, ENSG00000276051, ENSG00000204632, ENSG00000230413, ENSG00000235346, ENSG00000235680			rs145023077-?	rs145023077
33	ENSG00000235022, ENSG00000237733, ENSG00000230467, ENSG00000227171, ENSG00000206500, ENSG00000236967, ENSG00000230332, ENSG00000204618			rs142979264-?	rs142979264
34	ENSG00000214894			rs78868683-?	rs78868683
35		10214	11108	rs2530710-?	rs2530710
36		20695	22996	rs117759870-?	rs117759870
37	ENSG00000228964, ENSG00000223532, ENSG00000234745, ENSG00000232126, ENSG00000224608, ENSG00000206450			rs28752857-?	rs28752857
38	ENSG00000254405, ENSG00000255419, ENSG00000255152, ENSG00000264619, ENSG00000266442, ENSG00000266027			rs28399987-?	rs28399987
39	ENSG00000234947, ENSG00000226257, ENSG00000204344, ENSG00000206342, ENSG00000236250, ENSG00000226033			rs75393409-?	rs75393409
40	ENSG00000168477, ENSG00000236236, ENSG00000206258, ENSG00000229353, ENSG00000233323, ENSG00000231608			rs57079996-?	rs57079996
41		17607	12966	rs380911-?	rs380911
42		19762	18569	rs36022506-?	rs36022506
43		7858	3192	rs113721192-?	rs113721192
44		23969	14170	rs117231810-?	rs117231810
45		336	4785	rs910320-?	rs910320
46		28393	5794	rs9375182-?	rs9375182
47	ENSG00000146776			rs10272208-?	rs10272208
48	ENSG00000131558			rs147175877-?	rs147175877
49	ENSG00000226205, ENSG00000131558			rs149996974-?	rs149996974
50		72480	45045	rs2090064-?	rs2090064
51		44675	1963	rs4077818-?	rs4077818
52	ENSG00000225937, ENSG00000106772			rs17784899-?	rs17784899
53	ENSG00000119314			rs140330106-?	rs140330106
54	ENSG00000107614			rs147380957-?	rs147380957
55	ENSG00000129965, ENSG00000284779			rs150806792-?	rs150806792
56	ENSG00000133687			rs377042484-?	rs377042484
57		580	2422	rs144335201-?	rs144335201
58	ENSG00000261839, ENSG00000269293			rs79476506-?	rs79476506
59	ENSG00000189298, ENSG00000235109			rs213237-?	rs213237
60		11576	9279	rs77011267-?	rs77011267
61	ENSG00000287279			rs138803712-?	rs138803712
62		16346	37519	rs141456208-?	rs141456208
63		11662	9702	rs3129795-?	rs3129795

	W	X	Y	Z	AA	AB	AC	AD	AE	AF	AG	AH	AI	AJ
1														
2														
3	**MERGED**	**SNP_ID_CURR**	**CONTEXT**	**INTERGENIC**	**RISK ALLEL**	**P-VALUE**	**PVALUE_MLOG**	**OR or BETA**	**95% CI (TEX**	**PLATFORM [SNPS PASSING QC**	**CNV**	**MAPPED_TRAIT**	**STUDY ACCESSION**	
4	0			1	NR	9.00E-10	9.045757491	2.13	[1.67-2.70]	Illumina [632668]	N	cervical carcinoma	GCST001900	
5	0			1	0.15	4.00E-07	6.397940009	1.39	[1.23-1.58]	Illumina [632668]	N	cervical carcinoma	GCST001900	
6	0	2516448	intron_variant	0	NR	4.00E-18	17.39794001	1.42	[1.31-1.54]	Illumina [632668]	N	cervical carcinoma	GCST001900	
7	0	9272143	intron_variant	0	NR	3.00E-22	21.52287875	1.49	[1.37-1.61]	Illumina [632668]	N	cervical carcinoma	GCST001900	
8	0	3117027	intron_variant	0	NR	1.00E-07	7	1.25	[1.15-1.36]	Illumina [632668]	N	cervical carcinoma	GCST001900	
9	0			1	0.14	8.00E-08	7.096910013	1.42	[1.25-1.61]	Illumina [632668]	N	cervical carcinoma	GCST001900	
10	0	4782151	regulatory_region_variant	1	0.4677	5.00E-06	5.301029996	1.91	[1.44-2.52]	Affymetrix [556045]	N	cervical carcinoma	GCST002405	
11	0	11985951	intergenic_variant	1	0.871	6.00E-06	5.22184875	3.36	[1.94-5.83]	Affymetrix [556045]	N	cervical carcinoma	GCST002405	
12	0	6558578	intergenic_variant	1	0.7608	7.00E-06	5.15490196	2.31	[1.59-3.34]	Affymetrix [556045]	N	cervical carcinoma	GCST002405	
13	0	3899697	intron_variant	0	0.2787	9.00E-06	5.045757491	1.949	[1.449-2.621]	Affymetrix [556045]	N	cervical carcinoma	GCST002405	
14	0	997363	intron_variant	0	0.4032	1.00E-06	6	1.986	[1.50-2.62]	Affymetrix [556045]	N	cervical carcinoma	GCST002405	
15	0	7780883	regulatory_region_variant	1	0.05376	2.00E-06	5.698970004	3.28	[1.956-5.50]	Affymetrix [556045]	N	cervical carcinoma	GCST002405	
16	0	6726538	intergenic_variant	1	0.3871	3.00E-06	5.522878745	1.946	[1.471-2.573]	Affymetrix [556045]	N	cervical carcinoma	GCST002405	
17	0	8088832	intergenic_variant	1	0.508	4.00E-06	5.397940009	1.94	[1.46-2.57]	Affymetrix [556045]	N	cervical carcinoma	GCST002405	
18	0	12068654	intergenic_variant	1	0.885	5.00E-06	5.301029996	3.23	[2.06-6.93]	Affymetrix [556045]	N	cervical carcinoma	GCST002405	
19	0	142761815	intron_variant	0		4.00E-06	5.397940009	1.1	[1.06-1.14]	Illumina [7645193] (imputed)	N	cervical carcinoma	GCST009825	
20	0	146343995	intron_variant	0		5.00E-07	6.301029996	1.16	[1.09-1.23]	Illumina [7645193] (imputed)	N	cervical carcinoma	GCST009825	
21	0	141629209	regulatory_region_variant	1		4.00E-07	6.397940009	1.17	[1.10-1.25]	Illumina [7645193] (imputed)	N	cervical carcinoma	GCST009825	
22	0	145233920	intron_variant	0		2.00E-06	5.698970004	1.08	[1.05-1.11]	Illumina [7645193] (imputed)	N	cervical carcinoma	GCST009825	
23	0	149049108	3_prime_UTR_variant	0		4.00E-06	5.397940009	1.09	[1.05-1.13]	Illumina [7645193] (imputed)	N	cervical carcinoma	GCST009825	
24	0	138523818	intron_variant	0		2.00E-06	5.698970004	1.14	[1.08-1.20]	Illumina [7645193] (imputed)	N	cervical carcinoma	GCST009825	
25	0	41286572	intergenic_variant	1		4.00E-06	5.397940009	1.09	[1.05-1.13]	Illumina [7645193] (imputed)	N	cervical carcinoma	GCST009825	
26	0	72764914	intron_variant	0		3.00E-07	6.522878745	1.07	[1.04-1.10]	Illumina [7645193] (imputed)	N	cervical carcinoma	GCST009825	
27	0	140991990	intron_variant	0		7.00E-08	7.15490196	1.16	[1.10-1.23]	Illumina [7645193] (imputed)	N	cervical carcinoma	GCST009825	
28	0	56182403	regulatory_region_variant	1		9.00E-07	6.045757491	1.11	[1.06-1.15]	Illumina [7645193] (imputed)	N	cervical carcinoma	GCST009825	
29	0	75724373	regulatory_region_variant	1		8.00E-06	5.096910013	1.04	[1.02-1.06]	Illumina [7645193] (imputed)	N	cervical carcinoma	GCST009825	
30	0	201524967	intron_variant	0		3.00E-06	5.522878745	1.14	[1.08-1.20]	Illumina [7645193] (imputed)	N	cervical carcinoma	GCST009825	
31	0	75874947	missense_variant	0		2.00E-07	6.698970004	1.12	[1.07-1.17]	Illumina [7645193] (imputed)	N	cervical carcinoma	GCST009825	
32	0	145023077	intron_variant	0		7.00E-08	7.15490196	1.06	[1.04-1.09]	Illumina [7645193] (imputed)	N	cervical carcinoma	GCST009825	
33	0	142979264	splice_region_variant	0		6.00E-08	7.22184875	1.06	[1.04-1.09]	Illumina [7645193] (imputed)	N	cervical carcinoma	GCST009825	
34	0	78868683	intron_variant	0		3.00E-07	6.522878745	1.05	[1.03-1.08]	Illumina [7645193] (imputed)	N	cervical carcinoma	GCST009825	
35	0	2530710	intergenic_variant	1		4.00E-07	6.397940009	1.0416667	[1.03-1.06]	Illumina [7645193] (imputed)	N	cervical carcinoma	GCST009825	
36	0	117759870	regulatory_region_variant	1		6.00E-08	7.22184875	1.06	[1.04-1.09]	Illumina [7645193] (imputed)	N	cervical carcinoma	GCST009825	
37	0	28752857	intron_variant	0		5.00E-08	7.301029996	1.06	[1.04-1.08]	Illumina [7645193] (imputed)	N	cervical carcinoma	GCST009825	
38	0	28399987	5_prime_UTR_variant	0		7.00E-08	7.15490196	1.06	[1.04-1.09]	Illumina [7645193] (imputed)	N	cervical carcinoma	GCST009825	
39	0	75393409	synonymous_variant	0		2.00E-07	6.698970004	1.06	[1.04-1.09]	Illumina [7645193] (imputed)	N	cervical carcinoma	GCST009825	
40	0	57079996	intron_variant	0		1.00E-07	7	1.06	[1.04-1.09]	Illumina [7645193] (imputed)	N	cervical carcinoma	GCST009825	
41	0	380911	intergenic_variant	1		9.00E-06	5.045757491	1.04	[1.02-1.06]	Illumina [7645193] (imputed)	N	cervical carcinoma	GCST009825	
42	0	36022506	intron_variant	1		2.00E-06	5.698970004	1.06	[1.03-1.08]	Illumina [7645193] (imputed)	N	cervical carcinoma	GCST009825	
43	0	113721192	intergenic_variant	1		1.00E-06	6	1.06	[1.03-1.08]	Illumina [7645193] (imputed)	N	cervical carcinoma	GCST009825	
44	0	117231810	intergenic_variant	1		4.00E-06	5.397940009	1.06	[1.03-1.08]	Illumina [7645193] (imputed)	N	cervical carcinoma	GCST009825	
45	0	910320	regulatory_region_variant	1		8.00E-08	7.096910013	1.05	[1.03-1.07]	Illumina [7645193] (imputed)	N	cervical carcinoma	GCST009825	
46	0	9375182	intergenic_variant	1		3.00E-06	5.522878745	1.0309278	[1.02-1.05]	Illumina [7645193] (imputed)	N	cervical carcinoma	GCST009825	
47	0	10272208	intron_variant	0		5.00E-07	6.301029996	1.0309278	[1.02-1.05]	Illumina [7645193] (imputed)	N	cervical carcinoma	GCST009825	
48	0	147175877	intron_variant	0		6.00E-06	5.22184875	1.1	[1.05-1.14]	Illumina [7645193] (imputed)	N	cervical carcinoma	GCST009825	
49	0	149996974	non_coding_transcript_exon_variant	0		3.00E-06	5.522878745	1.14	[1.08-1.21]	Illumina [7645193] (imputed)	N	cervical carcinoma	GCST009825	
50	0	2090064	intergenic_variant	1		3.00E-07	6.522878745	1.0416667	[1.02-1.05]	Illumina [7645193] (imputed)	N	cervical carcinoma	GCST009825	
51	0	4077818	intergenic_variant	1		2.00E-06	5.698970004	1.04	[1.02-1.06]	Illumina [7645193] (imputed)	N	cervical carcinoma	GCST009825	
52	0	17784899	intron_variant	0		5.00E-07	6.301029996	1.05	[1.03-1.07]	Illumina [7645193] (imputed)	N	cervical carcinoma	GCST009825	
53	0	140330106	intron_variant	0		8.00E-06	5.096910013	1.1	[1.06-1.15]	Illumina [7645193] (imputed)	N	cervical carcinoma	GCST009825	
54	0	147380957	intron_variant	0		7.00E-06	5.15490196	1.07	[1.04-1.10]	Illumina [7645193] (imputed)	N	cervical carcinoma	GCST009825	
55	0	150806792	intron_variant	0		8.00E-08	7.096910013	1.19	[1.12-1.26]	Illumina [7645193] (imputed)	N	cervical carcinoma	GCST009825	
56	0	377042484	intron_variant	0		3.00E-06	5.522878745	1.12	[1.07-1.17]	Illumina [7645193] (imputed)	N	cervical carcinoma	GCST009825	
57	0	144335201	regulatory_region_variant	1		3.00E-06	5.522878745	1.78	[1.40-2.27]	Illumina [7645193] (imputed)	N	cervical carcinoma	GCST009826	
58	0	79476506	non_coding_transcript_exon_variant	0		1.00E-06	6	1.79	[1.41-2.26]	Illumina [7645193] (imputed)	N	cervical carcinoma	GCST009826	
59	0	213237	5_prime_UTR_variant	0		7.00E-07	6.15490196	1.36	[1.21-1.54]	Illumina [7645193] (imputed)	N	cervical carcinoma	GCST009826	
60	0	77011267	intergenic_variant	1		1.00E-06	6	1.79	[1.41-2.26]	Illumina [7645193] (imputed)	N	cervical carcinoma	GCST009826	
61	0	138803712	intron_variant	0		2.00E-06	5.698970004	1.82	[1.43-2.33]	Illumina [7645193] (imputed)	N	cervical carcinoma	GCST009826	
62	0	141456208	intergenic_variant	1		4.00E-06	5.397940009	1.75	[1.38-2.23]	Illumina [7645193] (imputed)	N	cervical carcinoma	GCST009826	
63	0	3129795	TF_binding_site_variant	1		2.00E-06	5.698970004	1.6129031	[1.32-1.96]	Illumina [7645193] (imputed)	N	cervical carcinoma	GCST009826	

	A	B	C	D	E	F	G	H
64	16/04/2020	31488892	Masuda T	05/09/2019	Eur J Hum Genet	www.ncbi.nlm.nih.gov/pubmed/31488892	GWAS of five gynecologic diseases and cross-trait analysis in Japanese.	Cervical cancer
65	16/04/2020	31488892	Masuda T	05/09/2019	Eur J Hum Genet	www.ncbi.nlm.nih.gov/pubmed/31488892	GWAS of five gynecologic diseases and cross-trait analysis in Japanese.	Cervical cancer
66	16/04/2020	31488892	Masuda T	05/09/2019	Eur J Hum Genet	www.ncbi.nlm.nih.gov/pubmed/31488892	GWAS of five gynecologic diseases and cross-trait analysis in Japanese.	Cervical cancer
67	16/04/2020	31488892	Masuda T	05/09/2019	Eur J Hum Genet	www.ncbi.nlm.nih.gov/pubmed/31488892	GWAS of five gynecologic diseases and cross-trait analysis in Japanese.	Cervical cancer
68	16/04/2020	31488892	Masuda T	05/09/2019	Eur J Hum Genet	www.ncbi.nlm.nih.gov/pubmed/31488892	GWAS of five gynecologic diseases and cross-trait analysis in Japanese.	Cervical cancer
69	16/04/2020	31488892	Masuda T	05/09/2019	Eur J Hum Genet	www.ncbi.nlm.nih.gov/pubmed/31488892	GWAS of five gynecologic diseases and cross-trait analysis in Japanese.	Cervical cancer
70	16/04/2020	31488892	Masuda T	05/09/2019	Eur J Hum Genet	www.ncbi.nlm.nih.gov/pubmed/31488892	GWAS of five gynecologic diseases and cross-trait analysis in Japanese.	Cervical cancer
71	16/04/2020	31488892	Masuda T	05/09/2019	Eur J Hum Genet	www.ncbi.nlm.nih.gov/pubmed/31488892	GWAS of five gynecologic diseases and cross-trait analysis in Japanese.	Cervical cancer
72	16/04/2020	31488892	Masuda T	05/09/2019	Eur J Hum Genet	www.ncbi.nlm.nih.gov/pubmed/31488892	GWAS of five gynecologic diseases and cross-trait analysis in Japanese.	Cervical cancer
73	16/04/2020	31488892	Masuda T	05/09/2019	Eur J Hum Genet	www.ncbi.nlm.nih.gov/pubmed/31488892	GWAS of five gynecologic diseases and cross-trait analysis in Japanese.	Cervical cancer
74	16/04/2020	31488892	Masuda T	05/09/2019	Eur J Hum Genet	www.ncbi.nlm.nih.gov/pubmed/31488892	GWAS of five gynecologic diseases and cross-trait analysis in Japanese.	Cervical cancer
75	16/04/2020	31488892	Masuda T	05/09/2019	Eur J Hum Genet	www.ncbi.nlm.nih.gov/pubmed/31488892	GWAS of five gynecologic diseases and cross-trait analysis in Japanese.	Cervical cancer
76	16/04/2020	31488892	Masuda T	05/09/2019	Eur J Hum Genet	www.ncbi.nlm.nih.gov/pubmed/31488892	GWAS of five gynecologic diseases and cross-trait analysis in Japanese.	Cervical cancer
77	16/04/2020	31488892	Masuda T	05/09/2019	Eur J Hum Genet	www.ncbi.nlm.nih.gov/pubmed/31488892	GWAS of five gynecologic diseases and cross-trait analysis in Japanese.	Cervical cancer
78	16/04/2020	31488892	Masuda T	05/09/2019	Eur J Hum Genet	www.ncbi.nlm.nih.gov/pubmed/31488892	GWAS of five gynecologic diseases and cross-trait analysis in Japanese.	Cervical cancer
79	16/04/2020	31488892	Masuda T	05/09/2019	Eur J Hum Genet	www.ncbi.nlm.nih.gov/pubmed/31488892	GWAS of five gynecologic diseases and cross-trait analysis in Japanese.	Cervical cancer
80	16/04/2020	31488892	Masuda T	05/09/2019	Eur J Hum Genet	www.ncbi.nlm.nih.gov/pubmed/31488892	GWAS of five gynecologic diseases and cross-trait analysis in Japanese.	Cervical cancer
81	16/04/2020	31488892	Masuda T	05/09/2019	Eur J Hum Genet	www.ncbi.nlm.nih.gov/pubmed/31488892	GWAS of five gynecologic diseases and cross-trait analysis in Japanese.	Cervical cancer
82	16/04/2020	31488892	Masuda T	05/09/2019	Eur J Hum Genet	www.ncbi.nlm.nih.gov/pubmed/31488892	GWAS of five gynecologic diseases and cross-trait analysis in Japanese.	Cervical cancer
83	16/04/2020	31488892	Masuda T	05/09/2019	Eur J Hum Genet	www.ncbi.nlm.nih.gov/pubmed/31488892	GWAS of five gynecologic diseases and cross-trait analysis in Japanese.	Cervical cancer
84	16/04/2020	31488892	Masuda T	05/09/2019	Eur J Hum Genet	www.ncbi.nlm.nih.gov/pubmed/31488892	GWAS of five gynecologic diseases and cross-trait analysis in Japanese.	Cervical cancer
85	16/04/2020	31488892	Masuda T	05/09/2019	Eur J Hum Genet	www.ncbi.nlm.nih.gov/pubmed/31488892	GWAS of five gynecologic diseases and cross-trait analysis in Japanese.	Cervical cancer
86	16/04/2020	31488892	Masuda T	05/09/2019	Eur J Hum Genet	www.ncbi.nlm.nih.gov/pubmed/31488892	GWAS of five gynecologic diseases and cross-trait analysis in Japanese.	Cervical cancer
87	16/04/2020	31488892	Masuda T	05/09/2019	Eur J Hum Genet	www.ncbi.nlm.nih.gov/pubmed/31488892	GWAS of five gynecologic diseases and cross-trait analysis in Japanese.	Cervical cancer
88	16/04/2020	31488892	Masuda T	05/09/2019	Eur J Hum Genet	www.ncbi.nlm.nih.gov/pubmed/31488892	GWAS of five gynecologic diseases and cross-trait analysis in Japanese.	Cervical cancer
89	16/04/2020	31488892	Masuda T	05/09/2019	Eur J Hum Genet	www.ncbi.nlm.nih.gov/pubmed/31488892	GWAS of five gynecologic diseases and cross-trait analysis in Japanese.	Cervical cancer
90	16/04/2020	31488892	Masuda T	05/09/2019	Eur J Hum Genet	www.ncbi.nlm.nih.gov/pubmed/31488892	GWAS of five gynecologic diseases and cross-trait analysis in Japanese.	Cervical cancer
91	16/04/2020	31488892	Masuda T	05/09/2019	Eur J Hum Genet	www.ncbi.nlm.nih.gov/pubmed/31488892	GWAS of five gynecologic diseases and cross-trait analysis in Japanese.	Cervical cancer
92	16/04/2020	31488892	Masuda T	05/09/2019	Eur J Hum Genet	www.ncbi.nlm.nih.gov/pubmed/31488892	GWAS of five gynecologic diseases and cross-trait analysis in Japanese.	Cervical cancer
93	16/04/2020	31488892	Masuda T	05/09/2019	Eur J Hum Genet	www.ncbi.nlm.nih.gov/pubmed/31488892	GWAS of five gynecologic diseases and cross-trait analysis in Japanese.	Cervical cancer
94	16/04/2020	31488892	Masuda T	05/09/2019	Eur J Hum Genet	www.ncbi.nlm.nih.gov/pubmed/31488892	GWAS of five gynecologic diseases and cross-trait analysis in Japanese.	Cervical cancer
95	16/04/2020	31488892	Masuda T	05/09/2019	Eur J Hum Genet	www.ncbi.nlm.nih.gov/pubmed/31488892	GWAS of five gynecologic diseases and cross-trait analysis in Japanese.	Cervical cancer
96	16/04/2020	31488892	Masuda T	05/09/2019	Eur J Hum Genet	www.ncbi.nlm.nih.gov/pubmed/31488892	GWAS of five gynecologic diseases and cross-trait analysis in Japanese.	Cervical cancer
97	16/04/2020	31488892	Masuda T	05/09/2019	Eur J Hum Genet	www.ncbi.nlm.nih.gov/pubmed/31488892	GWAS of five gynecologic diseases and cross-trait analysis in Japanese.	Cervical cancer
98	16/04/2020	31488892	Masuda T	05/09/2019	Eur J Hum Genet	www.ncbi.nlm.nih.gov/pubmed/31488892	GWAS of five gynecologic diseases and cross-trait analysis in Japanese.	Cervical cancer
99	16/04/2020	31488892	Masuda T	05/09/2019	Eur J Hum Genet	www.ncbi.nlm.nih.gov/pubmed/31488892	GWAS of five gynecologic diseases and cross-trait analysis in Japanese.	Cervical cancer
100	16/04/2020	31488892	Masuda T	05/09/2019	Eur J Hum Genet	www.ncbi.nlm.nih.gov/pubmed/31488892	GWAS of five gynecologic diseases and cross-trait analysis in Japanese.	Cervical cancer
101	16/04/2020	31488892	Masuda T	05/09/2019	Eur J Hum Genet	www.ncbi.nlm.nih.gov/pubmed/31488892	GWAS of five gynecologic diseases and cross-trait analysis in Japanese.	Cervical cancer
102	16/04/2020	31488892	Masuda T	05/09/2019	Eur J Hum Genet	www.ncbi.nlm.nih.gov/pubmed/31488892	GWAS of five gynecologic diseases and cross-trait analysis in Japanese.	Cervical cancer
103	16/04/2020	31488892	Masuda T	05/09/2019	Eur J Hum Genet	www.ncbi.nlm.nih.gov/pubmed/31488892	GWAS of five gynecologic diseases and cross-trait analysis in Japanese.	Cervical cancer
104	16/04/2020	31488892	Masuda T	05/09/2019	Eur J Hum Genet	www.ncbi.nlm.nih.gov/pubmed/31488892	GWAS of five gynecologic diseases and cross-trait analysis in Japanese.	Cervical cancer (multi-trait analysis)
105	16/04/2020	31488892	Masuda T	05/09/2019	Eur J Hum Genet	www.ncbi.nlm.nih.gov/pubmed/31488892	GWAS of five gynecologic diseases and cross-trait analysis in Japanese.	Cervical cancer (multi-trait analysis)
106	16/04/2020	31488892	Masuda T	05/09/2019	Eur J Hum Genet	www.ncbi.nlm.nih.gov/pubmed/31488892	GWAS of five gynecologic diseases and cross-trait analysis in Japanese.	Cervical cancer (multi-trait analysis)
107	16/04/2020	31488892	Masuda T	05/09/2019	Eur J Hum Genet	www.ncbi.nlm.nih.gov/pubmed/31488892	GWAS of five gynecologic diseases and cross-trait analysis in Japanese.	Cervical cancer (multi-trait analysis)
108	16/04/2020	31488892	Masuda T	05/09/2019	Eur J Hum Genet	www.ncbi.nlm.nih.gov/pubmed/31488892	GWAS of five gynecologic diseases and cross-trait analysis in Japanese.	Cervical cancer (multi-trait analysis)
109	16/04/2020	31488892	Masuda T	05/09/2019	Eur J Hum Genet	www.ncbi.nlm.nih.gov/pubmed/31488892	GWAS of five gynecologic diseases and cross-trait analysis in Japanese.	Cervical cancer (multi-trait analysis)
110	16/04/2020	31488892	Masuda T	05/09/2019	Eur J Hum Genet	www.ncbi.nlm.nih.gov/pubmed/31488892	GWAS of five gynecologic diseases and cross-trait analysis in Japanese.	Cervical cancer (multi-trait analysis)
111	16/04/2020	31488892	Masuda T	05/09/2019	Eur J Hum Genet	www.ncbi.nlm.nih.gov/pubmed/31488892	GWAS of five gynecologic diseases and cross-trait analysis in Japanese.	Cervical cancer (multi-trait analysis)
112	16/04/2020	31488892	Masuda T	05/09/2019	Eur J Hum Genet	www.ncbi.nlm.nih.gov/pubmed/31488892	GWAS of five gynecologic diseases and cross-trait analysis in Japanese.	Cervical cancer (multi-trait analysis)
113	16/04/2020	31488892	Masuda T	05/09/2019	Eur J Hum Genet	www.ncbi.nlm.nih.gov/pubmed/31488892	GWAS of five gynecologic diseases and cross-trait analysis in Japanese.	Cervical cancer (multi-trait analysis)
114	16/04/2020	31488892	Masuda T	05/09/2019	Eur J Hum Genet	www.ncbi.nlm.nih.gov/pubmed/31488892	GWAS of five gynecologic diseases and cross-trait analysis in Japanese.	Cervical cancer (multi-trait analysis)
115	16/04/2020	31488892	Masuda T	05/09/2019	Eur J Hum Genet	www.ncbi.nlm.nih.gov/pubmed/31488892	GWAS of five gynecologic diseases and cross-trait analysis in Japanese.	Cervical cancer (multi-trait analysis)
116	16/04/2020	31488892	Masuda T	05/09/2019	Eur J Hum Genet	www.ncbi.nlm.nih.gov/pubmed/31488892	GWAS of five gynecologic diseases and cross-trait analysis in Japanese.	Cervical cancer (multi-trait analysis)
117	16/04/2020	31488892	Masuda T	05/09/2019	Eur J Hum Genet	www.ncbi.nlm.nih.gov/pubmed/31488892	GWAS of five gynecologic diseases and cross-trait analysis in Japanese.	Cervical cancer (multi-trait analysis)
118	16/04/2020	31488892	Masuda T	05/09/2019	Eur J Hum Genet	www.ncbi.nlm.nih.gov/pubmed/31488892	GWAS of five gynecologic diseases and cross-trait analysis in Japanese.	Cervical cancer (multi-trait analysis)
119	16/04/2020	31488892	Masuda T	05/09/2019	Eur J Hum Genet	www.ncbi.nlm.nih.gov/pubmed/31488892	GWAS of five gynecologic diseases and cross-trait analysis in Japanese.	Cervical cancer (multi-trait analysis)
120	16/04/2020	31488892	Masuda T	05/09/2019	Eur J Hum Genet	www.ncbi.nlm.nih.gov/pubmed/31488892	GWAS of five gynecologic diseases and cross-trait analysis in Japanese.	Cervical cancer (multi-trait analysis)
121	16/04/2020	31488892	Masuda T	05/09/2019	Eur J Hum Genet	www.ncbi.nlm.nih.gov/pubmed/31488892	GWAS of five gynecologic diseases and cross-trait analysis in Japanese.	Cervical cancer (multi-trait analysis)
122	16/04/2020	31488892	Masuda T	05/09/2019	Eur J Hum Genet	www.ncbi.nlm.nih.gov/pubmed/31488892	GWAS of five gynecologic diseases and cross-trait analysis in Japanese.	Cervical cancer (multi-trait analysis)
123	16/04/2020	31488892	Masuda T	05/09/2019	Eur J Hum Genet	www.ncbi.nlm.nih.gov/pubmed/31488892	GWAS of five gynecologic diseases and cross-trait analysis in Japanese.	Cervical cancer (multi-trait analysis)
124	16/04/2020	31488892	Masuda T	05/09/2019	Eur J Hum Genet	www.ncbi.nlm.nih.gov/pubmed/31488892	GWAS of five gynecologic diseases and cross-trait analysis in Japanese.	Cervical cancer (multi-trait analysis)
125	16/04/2020	31488892	Masuda T	05/09/2019	Eur J Hum Genet	www.ncbi.nlm.nih.gov/pubmed/31488892	GWAS of five gynecologic diseases and cross-trait analysis in Japanese.	Cervical cancer (multi-trait analysis)
126	16/04/2020	31488892	Masuda T	05/09/2019	Eur J Hum Genet	www.ncbi.nlm.nih.gov/pubmed/31488892	GWAS of five gynecologic diseases and cross-trait analysis in Japanese.	Cervical cancer (multi-trait analysis)

	I	J	K	L	M	N
64	538 Japanese ancestry cases, 39,556 Japanese ancestry controls	NA	6p22.1	6	29256134	Intergenic
65	538 Japanese ancestry cases, 39,556 Japanese ancestry controls	NA	6p22.1	6	29641918	Intergenic
66	538 Japanese ancestry cases, 39,556 Japanese ancestry controls	NA	6p22.1	6	29829266	Intergenic
67	538 Japanese ancestry cases, 39,556 Japanese ancestry controls	NA	6p22.1	6	30075223	Intergenic
68	538 Japanese ancestry cases, 39,556 Japanese ancestry controls	NA	6p21.33	6	30830500	Intergenic
69	538 Japanese ancestry cases, 39,556 Japanese ancestry controls	NA	6p21.33	6	30972610	Intergenic
70	538 Japanese ancestry cases, 39,556 Japanese ancestry controls	NA	6p21.33	6	31343518	Intergenic
71	538 Japanese ancestry cases, 39,556 Japanese ancestry controls	NA	6p21.33	6	31562896	Intergenic
72	538 Japanese ancestry cases, 39,556 Japanese ancestry controls	NA	6p21.33	6	31771721	Intergenic
73	538 Japanese ancestry cases, 39,556 Japanese ancestry controls	NA	6p21.33	6	31972441	Intergenic
74	538 Japanese ancestry cases, 39,556 Japanese ancestry controls	NA	6p21.33	6	32093562	Intergenic
75	538 Japanese ancestry cases, 39,556 Japanese ancestry controls	NA	6p21.32	6	32241674	Intergenic
76	538 Japanese ancestry cases, 39,556 Japanese ancestry controls	NA	6p21.32	6	32482186	Intergenic
77	538 Japanese ancestry cases, 39,556 Japanese ancestry controls	NA	6p21.32	6	32714813	Intergenic
78	538 Japanese ancestry cases, 39,556 Japanese ancestry controls	NA	6p21.32	6	32920459	Intergenic
79	538 Japanese ancestry cases, 39,556 Japanese ancestry controls	NA	6p21.32	6	33108313	Intergenic
80	538 Japanese ancestry cases, 39,556 Japanese ancestry controls	NA	6q22.31	6	122773922	Intergenic
81	538 Japanese ancestry cases, 39,556 Japanese ancestry controls	NA	7q22.3	7	105691372	Intergenic
82	538 Japanese ancestry cases, 39,556 Japanese ancestry controls	NA	6p21.33	6	30512137	Intergenic
83	538 Japanese ancestry cases, 39,556 Japanese ancestry controls	NA	6p21.33	6	30719695	Intergenic
84	538 Japanese ancestry cases, 39,556 Japanese ancestry controls	NA	11p15.5	11	2158112	INS-IGF2
85	538 Japanese ancestry cases, 39,556 Japanese ancestry controls	NA	17q24.3	17	72101710	SOX9
86	538 Japanese ancestry cases, 39,556 Japanese ancestry controls	NA	1q23.3	1	162498796	Intergenic
87	538 Japanese ancestry cases, 39,556 Japanese ancestry controls	NA	1q23.3	1	162660256	Intergenic
88	538 Japanese ancestry cases, 39,556 Japanese ancestry controls	NA	2p25.2	2	4581682	Intergenic
89	538 Japanese ancestry cases, 39,556 Japanese ancestry controls	NA	2p16.3	2	50256810	Intergenic
90	538 Japanese ancestry cases, 39,556 Japanese ancestry controls	NA	2p13.1	2	73416499	Intergenic
91	538 Japanese ancestry cases, 39,556 Japanese ancestry controls	NA	2q12.1	2	105183335	Intergenic
92	538 Japanese ancestry cases, 39,556 Japanese ancestry controls	NA	2q24.3	2	164262092	Intergenic
93	538 Japanese ancestry cases, 39,556 Japanese ancestry controls	NA	2q32.1	2	185844965	Intergenic
94	538 Japanese ancestry cases, 39,556 Japanese ancestry controls	NA	2q36.3	2	226586765	Intergenic
95	538 Japanese ancestry cases, 39,556 Japanese ancestry controls	NA				Intergenic
96	538 Japanese ancestry cases, 39,556 Japanese ancestry controls	NA				Intergenic
97	538 Japanese ancestry cases, 39,556 Japanese ancestry controls	NA	3p12.2	3	81782913	Intergenic
98	538 Japanese ancestry cases, 39,556 Japanese ancestry controls	NA	3q28	3	190245452	Intergenic
99	538 Japanese ancestry cases, 39,556 Japanese ancestry controls	NA	4p16.3	4	2279800	Intergenic
100	538 Japanese ancestry cases, 39,556 Japanese ancestry controls	NA	4q26	4	113726137	Intergenic
101	538 Japanese ancestry cases, 39,556 Japanese ancestry controls	NA	4q31.22	4	146884350	Intergenic
102	538 Japanese ancestry cases, 39,556 Japanese ancestry controls	NA	6p22.1	6	27685380	Intergenic
103	538 Japanese ancestry cases, 39,556 Japanese ancestry controls	NA	20q13.33	20	64106159	Intergenic
104	538 Japanese ancestry cases, 39,556 Japanese ancestry controls	NA	6p21.33	6	30512137	Intergenic
105	538 Japanese ancestry cases, 39,556 Japanese ancestry controls	NA	6p21.33	6	30719695	Intergenic
106	538 Japanese ancestry cases, 39,556 Japanese ancestry controls	NA	11p15.5	11	2158112	INS-IGF2
107	538 Japanese ancestry cases, 39,556 Japanese ancestry controls	NA	17q24.3	17	72101710	SOX9
108	538 Japanese ancestry cases, 39,556 Japanese ancestry controls	NA	1q23.1	1	157596180	Intergenic
109	538 Japanese ancestry cases, 39,556 Japanese ancestry controls	NA	1q23.3	1	162660256	Intergenic
110	538 Japanese ancestry cases, 39,556 Japanese ancestry controls	NA	2p25.2	2	4581682	Intergenic
111	538 Japanese ancestry cases, 39,556 Japanese ancestry controls	NA	2p16.3	2	50256810	Intergenic
112	538 Japanese ancestry cases, 39,556 Japanese ancestry controls	NA	2p13.1	2	73416499	Intergenic
113	538 Japanese ancestry cases, 39,556 Japanese ancestry controls	NA	2q24.3	2	164262092	Intergenic
114	538 Japanese ancestry cases, 39,556 Japanese ancestry controls	NA	2q32.1	2	185844965	Intergenic
115	538 Japanese ancestry cases, 39,556 Japanese ancestry controls	NA	2q36.3	2	226586765	Intergenic
116	538 Japanese ancestry cases, 39,556 Japanese ancestry controls	NA	3p25.1	3	13530464	Intergenic
117	538 Japanese ancestry cases, 39,556 Japanese ancestry controls	NA				Intergenic
118	538 Japanese ancestry cases, 39,556 Japanese ancestry controls	NA	3q28	3	190245452	Intergenic
119	538 Japanese ancestry cases, 39,556 Japanese ancestry controls	NA	4p16.3	4	2279800	Intergenic
120	538 Japanese ancestry cases, 39,556 Japanese ancestry controls	NA	4q26	4	113726137	Intergenic
121	538 Japanese ancestry cases, 39,556 Japanese ancestry controls	NA	4q31.22	4	146884350	Intergenic
122	538 Japanese ancestry cases, 39,556 Japanese ancestry controls	NA	6p22.1	6	28031037	Intergenic
123	538 Japanese ancestry cases, 39,556 Japanese ancestry controls	NA	6p22.1	6	28137256	Intergenic
124	538 Japanese ancestry cases, 39,556 Japanese ancestry controls	NA	6p22.1	6	28356161	Intergenic
125	538 Japanese ancestry cases, 39,556 Japanese ancestry controls	NA	6p22.1	6	28499293	Intergenic
126	538 Japanese ancestry cases, 39,556 Japanese ancestry controls	NA	6p22.1	6	28639807	Intergenic

	O	P	Q
64	AL672167.1		
65	SUMO2P1 - MOG	ENSG00000228130	ENSG00000236561
66	HLA-G, HLA-G, HLA-G, HLA-G, HLA-G, HLA-G, HLA-G, HLA-G		
67	RNF39, RNF39, RNF39, RNF39, RNF39, RNF39, RNF39, RNF39		
68	LINC00243		
69	NAPGP2 - MUC21	ENSG00000275906	ENSG00000231350
70	HLA-B, HLA-B, HLA-B, HLA-B, HLA-B, HLA-B		
71	NFKBIL1 - LTA	ENSG00000206440	ENSG00000226275
72	VWA7, VWA7, VWA7, VWA7, VWA7, VWA7		
73	STK19, STK19, STK19, STK19, STK19, STK19		
74	TNXB, TNXB, TNXB, TNXB, TNXB, TNXB		
75	NOTCH4 - TSBP1-AS1	ENSG00000206312	ENSG00000235135
76	HLA-DRB9 - HLA-DRB5	ENSG00000226035	ENSG00000198502
77	MTCO3P1 - AL662789.1	ENSG00000237145	ENSG00000232080
78	HLA-Z - HLA-DMB	ENSG00000237871	ENSG00000226264
79	COL11A2P1 - HLA-DPB2	ENSG00000230816	ENSG00000225132
80	RN7SL564P - FABP7	ENSG00000240606	ENSG00000164434
81	ATXN7L1		
82	HLA-E - LINC02569	ENSG00000206493	ENSG00000235781
83	MDC1 - TUBB	ENSG00000206481	ENSG00000229684
84	INS-IGF2, AC132217.2		
85	LINC02097, SOX9-AS1		
86	UHMK1		
87	DDR2		
88	NPM1P48 - LINC01249	ENSG00000231821	ENSG00000231532
89	NRXN1		
90	ALMS1		
91	LINC01918 - GPR45	ENSG00000226508	ENSG00000135973
92	AC016766.1		
93	FSIP2 - AC097500.1	ENSG00000188738	ENSG00000287129
94	AC062015.1 - MIR5702	ENSG00000235070	ENSG00000263363
95			
96			
97	AC017015.2 - AC129807.1	ENSG00000242190	ENSG00000242009
98	NMNAT1P3 - CLDN1	ENSG00000238077	ENSG00000163347
99	ZFYVE28		
100	CAMK2D		
101	TTC29		
102	AL009179.3 - AL009179.1	ENSG00000287674	ENSG00000281706
103	NPBWR2, MYT1		
104	HLA-E - LINC02569	ENSG00000206493	ENSG00000235781
105	MDC1 - TUBB	ENSG00000206481	ENSG00000229684
106	INS-IGF2, AC132217.2		
107	LINC02097, SOX9-AS1		
108	FCRL4		
109	DDR2		
110	NPM1P48 - LINC01249	ENSG00000231821	ENSG00000231532
111	NRXN1		
112	ALMS1		
113	AC016766.1		
114	FSIP2 - AC097500.1	ENSG00000188738	ENSG00000287129
115	AC062015.1 - MIR5702	ENSG00000235070	ENSG00000263363
116	HDAC11 - FBLN2	ENSG00000163517	ENSG00000163520
117			
118	NMNAT1P3 - CLDN1	ENSG00000238077	ENSG00000163347
119	ZFYVE28		
120	CAMK2D		
121	TTC29		
122	ZSCAN16-AS1		
123	AL358933.1, ZSCAN16-AS1		
124	ZKSCAN3, ZSCAN31		
125	Z98745.1 - GPX6	ENSG00000286819	ENSG00000198704
126	AL121932.2		

	R	S	T	U	V
64	ENSG00000197171			rs149016863-?	rs149016863
65		5575	15084	rs9257927-?	rs9257927
66	ENSG00000206506, ENSG00000233095, ENSG00000237216, ENSG00000276051, ENSG00000204632, ENSG00000230413, ENSG00000235346, ENSG00000235680			rs145023077-?	rs145023077
67	ENSG00000235022, ENSG00000237733, ENSG00000230467, ENSG00000227171, ENSG00000206500, ENSG00000236967, ENSG00000230332, ENSG00000204618			rs142979264-?	rs142979264
68	ENSG00000214894			rs78868683-?	rs78868683
69		10214	11108	rs2530710-?	rs2530710
70	ENSG00000228964, ENSG00000223532, ENSG00000234745, ENSG00000232126, ENSG00000224608, ENSG00000206450			rs28380833-?	rs28380833
71		4067	9158	rs138885312-?	rs138885312
72	ENSG00000238203, ENSG00000230048, ENSG00000204396, ENSG00000223757, ENSG00000234433, ENSG00000227144			rs143029752-?	rs143029752
73	ENSG00000234947, ENSG00000226257, ENSG00000204344, ENSG00000206342, ENSG00000236250, ENSG00000226033			rs75393409-?	rs75393409
74	ENSG00000168477, ENSG00000236236, ENSG00000206258, ENSG00000229353, ENSG00000233323, ENSG00000231608			rs57079996-?	rs57079996
75		17607	12966	rs380911-?	rs380911
76		8686	35167	rs367607265-?	rs367607265
77		7858	3192	rs113721192-?	rs113721192
78		23969	14170	rs117231810-?	rs117231810
79		983	4138	rs872956-?	rs872956
80		28393	5794	rs9375182-?	rs9375182
81	ENSG00000146776			rs10272208-?	rs10272208
82		17943	4129	rs140668832-?	rs140668832
83		2248	657	rs117670375-?	rs117670375
84	ENSG00000129965, ENSG00000284779			rs150806792-?	rs150806792
85	ENSG00000226101, ENSG00000234899			rs140991990-?	rs140991990
86	ENSG00000152332			rs57561731-?	rs57561731
87	ENSG00000162733			rs75068199-?	rs75068199
88		66626	46534	rs76883373-?	rs76883373
89	ENSG00000179915			rs62133116-?	rs62133116
90	ENSG00000116127			rs11684858-?	rs11684858
91		37911	58408	rs2679850-?	rs2679850
92	ENSG00000237844			rs190074712-?	rs190074712
93		11675	105504	rs183319101-?	rs183319101
94		401394	71945	rs559021322-?	rs559021322
95				chr3:59760625-?	chr3:59760625
96				chr3:59866982-?	chr3:59866982
97		18157	57601	rs76537691-?	rs76537691
98		77801	60255	rs117143670-?	rs117143670
99	ENSG00000159733			rs556607390-?	rs556607390
100	ENSG00000145349			rs146513397-?	rs146513397
101	ENSG00000137473			rs185348880-?	rs185348880
102		4825	8655	rs77050118-?	rs77050118
103	ENSG00000125522, ENSG00000196132			rs75874947-?	rs75874947
104		17943	4129	rs140668832-?	rs140668832
105		2248	657	rs117670375-?	rs117670375
106	ENSG00000129965, ENSG00000284779			rs150806792-?	rs150806792
107	ENSG00000226101, ENSG00000234899			rs140991990-?	rs140991990
108	ENSG00000163518			rs1885560-?	rs1885560
109	ENSG00000162733			rs75068199-?	rs75068199
110		66626	46534	rs76883373-?	rs76883373
111	ENSG00000179915			rs62133116-?	rs62133116
112	ENSG00000116127			rs11684858-?	rs11684858
113	ENSG00000237844			rs190074712-?	rs190074712
114		11675	105504	rs183319101-?	rs183319101
115		401394	71945	rs559021322-?	rs559021322
116		24040	18661	rs59692692-?	rs59692692
117				chr3:59866982-?	chr3:59866982
118		77801	60255	rs117143670-?	rs117143670
119	ENSG00000159733			rs556607390-?	rs556607390
120	ENSG00000145349			rs146513397-?	rs146513397
121	ENSG00000137473			rs185348880-?	rs185348880
122	ENSG00000269293			rs74994185-?	rs74994185
123	ENSG00000261839, ENSG00000269293			rs79476506-?	rs79476506
124	ENSG00000189298, ENSG00000235109			rs213237-?	rs213237
125		7632	4003	rs146362527-?	rs146362527
126	ENSG00000287279			rs138803712-?	rs138803712

	W	X	Y	Z	AA	AB	AC	AD	AE	AF	AG	AH	AI	AJ
64	0	149016863	intron_variant	0		3.00E-06	5.522878745	1.74	[1.38-2.20]	Illumina [7645193] (imputed)	N	cervical carcinoma	GCST009826	
65	0	9257927	regulatory_region_variant	1		1.00E-08	8	1.66	[1.39-1.97]	Illumina [7645193] (imputed)	N	cervical carcinoma	GCST009826	
66	0	145023077	intron_variant	0		8.00E-09	8.096910013	1.83	[1.49-2.25]	Illumina [7645193] (imputed)	N	cervical carcinoma	GCST009826	
67	0	142979264	splice_region_variant	0		7.00E-09	8.15490196	1.84	[1.50-2.26]	Illumina [7645193] (imputed)	N	cervical carcinoma	GCST009826	
68	0	78868683	intron_variant	0		1.00E-07	7	1.68	[1.39-2.04]	Illumina [7645193] (imputed)	N	cervical carcinoma	GCST009826	
69	0	2530710	intergenic_variant	1		1.00E-07	7	1.5151515	[1.3-1.75]	Illumina [7645193] (imputed)	N	cervical carcinoma	GCST009826	
70	0	28380833	intron_variant	0		8.00E-09	8.096910013	1.8	[1.47-2.20]	Illumina [7645193] (imputed)	N	cervical carcinoma	GCST009826	
71	0	138885312	intergenic_variant	1		2.00E-08	7.698970004	1.8	[1.47-2.20]	Illumina [7645193] (imputed)	N	cervical carcinoma	GCST009826	
72	0	143029752	non_coding_transcript_exon_variant	0		3.00E-08	7.522878745	1.78	[1.45-2.19]	Illumina [7645193] (imputed)	N	cervical carcinoma	GCST009826	
73	0	75393409	synonymous_variant	0		4.00E-08	7.397940009	1.78	[1.45-2.18]	Illumina [7645193] (imputed)	N	cervical carcinoma	GCST009826	
74	0	57079996	intron_variant	0		4.00E-08	7.397940009	1.77	[1.45-2.18]	Illumina [7645193] (imputed)	N	cervical carcinoma	GCST009826	
75	0	380911	intergenic_variant	1		3.00E-06	5.522878745	1.51	[1.27-1.80]	Illumina [7645193] (imputed)	N	cervical carcinoma	GCST009826	
76	0	367607265	regulatory_region_variant	1		2.00E-07	6.698970004	1.74	[1.41-2.14]	Illumina [7645193] (imputed)	N	cervical carcinoma	GCST009826	
77	0	113721192	intergenic_variant	1		3.00E-07	6.522878745	1.76	[1.42-2.18]	Illumina [7645193] (imputed)	N	cervical carcinoma	GCST009826	
78	0	117231810	intergenic_variant	1		1.00E-06	6	1.69	[1.37-2.09]	Illumina [7645193] (imputed)	N	cervical carcinoma	GCST009826	
79	0	872956	regulatory_region_variant	1		8.00E-08	7.096910013	1.58	[1.34-1.86]	Illumina [7645193] (imputed)	N	cervical carcinoma	GCST009826	
80	0	9375182	intergenic_variant	1		3.00E-06	5.522878745	1.3513513	[1.19-1.54]	Illumina [7645193] (imputed)	N	cervical carcinoma	GCST009826	
81	0	10272208	intron_variant	0		2.00E-06	5.698970004	1.3333333	[1.19-1.52]	Illumina [7645193] (imputed)	N	cervical carcinoma	GCST009826	
82	0	140668832	intergenic_variant	1		3.00E-10	9.522878745	1.92	[1.57-2.35]	Illumina [7645193] (imputed)	N	cervical carcinoma	GCST009826	
83	0	117670375	regulatory_region_variant	1		3.00E-10	9.522878745	1.92	[1.57-2.35]	Illumina [7645193] (imputed)	N	cervical carcinoma	GCST009826	
84	0	150806792	intron_variant	0		5.00E-08	7.301029996	4.96	[2.79-8.83]	Illumina [7645193] (imputed)	N	cervical carcinoma	GCST009826	
85	0	140991990	intron_variant	0		3.00E-08	7.522878745	4.94	[2.80-8.71]	Illumina [7645193] (imputed)	N	cervical carcinoma	GCST009826	
86	0	57561731	intron_variant	0		9.00E-06	5.045757491	1.7	[1.34-2.14]	Illumina [7645193] (imputed)	N	cervical carcinoma	GCST009826	
87	0	75068199	intron_variant	0		4.00E-07	6.397940009	1.82	[1.44-2.30]	Illumina [7645193] (imputed)	N	cervical carcinoma	GCST009826	
88	0	76883373	intergenic_variant	1		8.00E-06	5.096910013	1.97	[1.46-2.65]	Illumina [7645193] (imputed)	N	cervical carcinoma	GCST009826	
89	0	62133116	intron_variant	0		2.00E-06	5.698970004	1.48	[1.26-1.74]	Illumina [7645193] (imputed)	N	cervical carcinoma	GCST009826	
90	0	11684858	intron_variant	0		7.00E-06	5.15490196	1.79	[1.39-2.30]	Illumina [7645193] (imputed)	N	cervical carcinoma	GCST009826	
91	0	2679850	intergenic_variant	1		4.00E-06	5.397940009	2.2222223	[1.59-3.12]	Illumina [7645193] (imputed)	N	cervical carcinoma	GCST009826	
92	0	190074712	intron_variant	0		3.00E-07	6.522878745	4.68	[2.60-8.43]	Illumina [7645193] (imputed)	N	cervical carcinoma	GCST009826	
93	0	183319101	intergenic_variant	1		8.00E-06	5.096910013	1.3333333	[1.18-1.52]	Illumina [7645193] (imputed)	N	cervical carcinoma	GCST009826	
94	0	559021322	intergenic_variant	1		2.00E-06	5.698970004	3.63	[2.14-6.17]	Illumina [7645193] (imputed)	N	cervical carcinoma	GCST009826	
95	0			1		3.00E-06	5.522878745	4.15	[2.28-7.58]	Illumina [7645193] (imputed)	N	cervical carcinoma	GCST009826	
96	0			1		5.00E-07	6.301029996	4.98	[2.67-9.30]	Illumina [7645193] (imputed)	N	cervical carcinoma	GCST009826	
97	0	76537691	regulatory_region_variant	1		8.00E-06	5.096910013	2.81	[1.78-4.43]	Illumina [7645193] (imputed)	N	cervical carcinoma	GCST009826	
98	0	117143670	intergenic_variant	1		5.00E-06	5.301029996	3.45	[2.03-5.86]	Illumina [7645193] (imputed)	N	cervical carcinoma	GCST009826	
99	0	556607390	intron_variant	0		6.00E-06	5.22184875	2.25	[1.58-3.21]	Illumina [7645193] (imputed)	N	cervical carcinoma	GCST009826	
100	0	146513397	intron_variant	0		7.00E-07	6.15490196	3.95	[2.30-6.79]	Illumina [7645193] (imputed)	N	cervical carcinoma	GCST009826	
101	0	185348880	intron_variant	0		8.00E-07	6.096910013	3.42	[2.10-5.57]	Illumina [7645193] (imputed)	N	cervical carcinoma	GCST009826	
102	0	77050118	intergenic_variant	1		3.00E-06	5.522878745	1.79	[1.40-2.28]	Illumina [7645193] (imputed)	N	cervical carcinoma	GCST009826	
103	0	75874947	missense_variant	0		1.00E-07	7	2.84	[1.93-4.19]	Illumina [7645193] (imputed)	N	cervical carcinoma	GCST009826	
104	0	140668832	intergenic_variant	1		2.00E-09	8.698970004	1.07	[1.05-1.09]	Illumina [7645193] (imputed)	N	cervical carcinoma	GCST009825	
105	0	117670375	regulatory_region_variant	1		2.00E-09	8.698970004	1.07	[1.05-1.09]	Illumina [7645193] (imputed)	N	cervical carcinoma	GCST009825	
106	0	150806792	intron_variant	0		8.00E-08	7.096910013	1.19	[1.12-1.26]	Illumina [7645193] (imputed)	N	cervical carcinoma	GCST009825	
107	0	140991990	intron_variant	0		7.00E-08	7.15490196	1.16	[1.10-1.23]	Illumina [7645193] (imputed)	N	cervical carcinoma	GCST009825	
108	0	1885560	intron_variant	0		6.00E-06	5.22184875	1.0526316	[1.03-1.09]	Illumina [7645193] (imputed)	N	cervical carcinoma	GCST009825	
109	0	75068199	intron_variant	0		4.00E-07	6.397940009	1.07	[1.04-1.10]	Illumina [7645193] (imputed)	N	cervical carcinoma	GCST009825	
110	0	76883373	intergenic_variant	1		5.00E-06	5.301029996	1.07	[1.04-1.10]	Illumina [7645193] (imputed)	N	cervical carcinoma	GCST009825	
111	0	62133116	intron_variant	0		2.00E-06	5.698970004	1.05	[1.03-1.07]	Illumina [7645193] (imputed)	N	cervical carcinoma	GCST009825	
112	0	11684858	intron_variant	0		4.00E-06	5.397940009	1.06	[1.03-1.09]	Illumina [7645193] (imputed)	N	cervical carcinoma	GCST009825	
113	0	190074712	intron_variant	0		2.00E-06	5.698970004	1.17	[1.10-1.25]	Illumina [7645193] (imputed)	N	cervical carcinoma	GCST009825	
114	0	183319101	intergenic_variant	1		6.00E-06	5.22184875	1.0309278	[1.02-1.05]	Illumina [7645193] (imputed)	N	cervical carcinoma	GCST009825	
115	0	559021322	intergenic_variant	1		4.00E-06	5.397940009	1.13	[1.07-1.19]	Illumina [7645193] (imputed)	N	cervical carcinoma	GCST009825	
116	0	59692692	intergenic_variant	1		6.00E-06	5.22184875	1.06	[1.03-1.09]	Illumina [7645193] (imputed)	N	cervical carcinoma	GCST009825	
117	0			1		3.00E-06	5.522878745	1.16	[1.09-1.24]	Illumina [7645193] (imputed)	N	cervical carcinoma	GCST009825	
118	0	117143670	intergenic_variant	1		7.00E-06	5.15490196	1.13	[1.07-1.20]	Illumina [7645193] (imputed)	N	cervical carcinoma	GCST009825	
119	0	556607390	intron_variant	0		3.00E-06	5.522878745	1.1	[1.05-1.14]	Illumina [7645193] (imputed)	N	cervical carcinoma	GCST009825	
120	0	146513397	intron_variant	0		1.00E-06	6	1.16	[1.09-1.23]	Illumina [7645193] (imputed)	N	cervical carcinoma	GCST009825	
121	0	185348880	intron_variant	0		6.00E-06	5.22184875	1.13	[1.07-1.19]	Illumina [7645193] (imputed)	N	cervical carcinoma	GCST009825	
122	0	74994185	intron_variant	0		6.00E-06	5.22184875	1.06	[1.04-1.09]	Illumina [7645193] (imputed)	N	cervical carcinoma	GCST009825	
123	0	79476506	non_coding_transcript_exon_variant	0		7.00E-06	5.15490196	1.06	[1.03-1.09]	Illumina [7645193] (imputed)	N	cervical carcinoma	GCST009825	
124	0	213237	5_prime_UTR_variant	0		2.00E-06	5.698970004	1.03	[1.02-1.05]	Illumina [7645193] (imputed)	N	cervical carcinoma	GCST009825	
125	0	146362527	intergenic_variant	1		7.00E-06	5.15490196	1.06	[1.03-1.09]	Illumina [7645193] (imputed)	N	cervical carcinoma	GCST009825	
126	0	138803712	intron_variant	0		8.00E-06	5.096910013	1.06	[1.03-1.09]	Illumina [7645193] (imputed)	N	cervical carcinoma	GCST009825	

	A	B	C	D	E	F	G	H
127	16/04/2020	31488892	Masuda T	05/09/2019	Eur J Hum Genet	www.ncbi.nlm.nih.gov/pubmed/31488892	GWAS of five gynecologic diseases and cross-trait analysis in Japanese.	Cervical cancer (multi-trait analysis)
128	16/04/2020	31488892	Masuda T	05/09/2019	Eur J Hum Genet	www.ncbi.nlm.nih.gov/pubmed/31488892	GWAS of five gynecologic diseases and cross-trait analysis in Japanese.	Cervical cancer
129	16/04/2020	31488892	Masuda T	05/09/2019	Eur J Hum Genet	www.ncbi.nlm.nih.gov/pubmed/31488892	GWAS of five gynecologic diseases and cross-trait analysis in Japanese.	Cervical cancer
130	16/04/2020	31488892	Masuda T	05/09/2019	Eur J Hum Genet	www.ncbi.nlm.nih.gov/pubmed/31488892	GWAS of five gynecologic diseases and cross-trait analysis in Japanese.	Cervical cancer
131	16/04/2020	31488892	Masuda T	05/09/2019	Eur J Hum Genet	www.ncbi.nlm.nih.gov/pubmed/31488892	GWAS of five gynecologic diseases and cross-trait analysis in Japanese.	Cervical cancer
132	16/04/2020	31488892	Masuda T	05/09/2019	Eur J Hum Genet	www.ncbi.nlm.nih.gov/pubmed/31488892	GWAS of five gynecologic diseases and cross-trait analysis in Japanese.	Cervical cancer
133	16/04/2020	31488892	Masuda T	05/09/2019	Eur J Hum Genet	www.ncbi.nlm.nih.gov/pubmed/31488892	GWAS of five gynecologic diseases and cross-trait analysis in Japanese.	Cervical cancer
134	16/04/2020	31488892	Masuda T	05/09/2019	Eur J Hum Genet	www.ncbi.nlm.nih.gov/pubmed/31488892	GWAS of five gynecologic diseases and cross-trait analysis in Japanese.	Cervical cancer
135	16/04/2020	31488892	Masuda T	05/09/2019	Eur J Hum Genet	www.ncbi.nlm.nih.gov/pubmed/31488892	GWAS of five gynecologic diseases and cross-trait analysis in Japanese.	Cervical cancer
136	16/04/2020	31488892	Masuda T	05/09/2019	Eur J Hum Genet	www.ncbi.nlm.nih.gov/pubmed/31488892	GWAS of five gynecologic diseases and cross-trait analysis in Japanese.	Cervical cancer
137	16/04/2020	31488892	Masuda T	05/09/2019	Eur J Hum Genet	www.ncbi.nlm.nih.gov/pubmed/31488892	GWAS of five gynecologic diseases and cross-trait analysis in Japanese.	Cervical cancer
138	16/04/2020	31488892	Masuda T	05/09/2019	Eur J Hum Genet	www.ncbi.nlm.nih.gov/pubmed/31488892	GWAS of five gynecologic diseases and cross-trait analysis in Japanese.	Cervical cancer
139	16/04/2020	31488892	Masuda T	05/09/2019	Eur J Hum Genet	www.ncbi.nlm.nih.gov/pubmed/31488892	GWAS of five gynecologic diseases and cross-trait analysis in Japanese.	Cervical cancer
140	16/04/2020	31488892	Masuda T	05/09/2019	Eur J Hum Genet	www.ncbi.nlm.nih.gov/pubmed/31488892	GWAS of five gynecologic diseases and cross-trait analysis in Japanese.	Cervical cancer
141	16/04/2020	31488892	Masuda T	05/09/2019	Eur J Hum Genet	www.ncbi.nlm.nih.gov/pubmed/31488892	GWAS of five gynecologic diseases and cross-trait analysis in Japanese.	Cervical cancer
142	16/04/2020	31488892	Masuda T	05/09/2019	Eur J Hum Genet	www.ncbi.nlm.nih.gov/pubmed/31488892	GWAS of five gynecologic diseases and cross-trait analysis in Japanese.	Cervical cancer
143	16/04/2020	31488892	Masuda T	05/09/2019	Eur J Hum Genet	www.ncbi.nlm.nih.gov/pubmed/31488892	GWAS of five gynecologic diseases and cross-trait analysis in Japanese.	Cervical cancer
144	16/04/2020	31488892	Masuda T	05/09/2019	Eur J Hum Genet	www.ncbi.nlm.nih.gov/pubmed/31488892	GWAS of five gynecologic diseases and cross-trait analysis in Japanese.	Cervical cancer
145	16/04/2020	31488892	Masuda T	05/09/2019	Eur J Hum Genet	www.ncbi.nlm.nih.gov/pubmed/31488892	GWAS of five gynecologic diseases and cross-trait analysis in Japanese.	Cervical cancer
146	16/04/2020	31488892	Masuda T	05/09/2019	Eur J Hum Genet	www.ncbi.nlm.nih.gov/pubmed/31488892	GWAS of five gynecologic diseases and cross-trait analysis in Japanese.	Cervical cancer
147	16/04/2020	31488892	Masuda T	05/09/2019	Eur J Hum Genet	www.ncbi.nlm.nih.gov/pubmed/31488892	GWAS of five gynecologic diseases and cross-trait analysis in Japanese.	Cervical cancer
148	16/04/2020	31488892	Masuda T	05/09/2019	Eur J Hum Genet	www.ncbi.nlm.nih.gov/pubmed/31488892	GWAS of five gynecologic diseases and cross-trait analysis in Japanese.	Cervical cancer
149	16/04/2020	31488892	Masuda T	05/09/2019	Eur J Hum Genet	www.ncbi.nlm.nih.gov/pubmed/31488892	GWAS of five gynecologic diseases and cross-trait analysis in Japanese.	Cervical cancer
150	16/04/2020	31488892	Masuda T	05/09/2019	Eur J Hum Genet	www.ncbi.nlm.nih.gov/pubmed/31488892	GWAS of five gynecologic diseases and cross-trait analysis in Japanese.	Cervical cancer
151	16/04/2020	31488892	Masuda T	05/09/2019	Eur J Hum Genet	www.ncbi.nlm.nih.gov/pubmed/31488892	GWAS of five gynecologic diseases and cross-trait analysis in Japanese.	Cervical cancer
152	16/04/2020	31488892	Masuda T	05/09/2019	Eur J Hum Genet	www.ncbi.nlm.nih.gov/pubmed/31488892	GWAS of five gynecologic diseases and cross-trait analysis in Japanese.	Cervical cancer
153	12/02/2019	30412241	Takeuchi F	09/11/2018	Hum Mol Genet	www.ncbi.nlm.nih.gov/pubmed/30412241	Genome-wide association study of cervical cancer suggests a role for ARRDC3 gene in hu	Cervical cancer
154	12/02/2019	30412241	Takeuchi F	09/11/2018	Hum Mol Genet	www.ncbi.nlm.nih.gov/pubmed/30412241	Genome-wide association study of cervical cancer suggests a role for ARRDC3 gene in hu	Cervical cancer
155	12/02/2019	30412241	Takeuchi F	09/11/2018	Hum Mol Genet	www.ncbi.nlm.nih.gov/pubmed/30412241	Genome-wide association study of cervical cancer suggests a role for ARRDC3 gene in hu	Cervical cancer
156	12/02/2019	30412241	Takeuchi F	09/11/2018	Hum Mol Genet	www.ncbi.nlm.nih.gov/pubmed/30412241	Genome-wide association study of cervical cancer suggests a role for ARRDC3 gene in hu	Cervical cancer
157	12/02/2019	30412241	Takeuchi F	09/11/2018	Hum Mol Genet	www.ncbi.nlm.nih.gov/pubmed/30412241	Genome-wide association study of cervical cancer suggests a role for ARRDC3 gene in hu	Cervical cancer
158	27/01/2017	27285765	Chen D	07/06/2016	Oncotarget	www.ncbi.nlm.nih.gov/pubmed/27285765	Pooled analysis of genome-wide association studies of cervical intraepithelial neoplasia 3	Cervical cancer
159	03/10/2017	28806749	Leo PJ	14/08/2017	PLoS Genet	www.ncbi.nlm.nih.gov/pubmed/28806749	Defining the genetic susceptibility to cervical neoplasia-A genome-wide association study.	Cervical cancer
160	03/10/2017	28806749	Leo PJ	14/08/2017	PLoS Genet	www.ncbi.nlm.nih.gov/pubmed/28806749	Defining the genetic susceptibility to cervical neoplasia-A genome-wide association study.	Cervical cancer
161	03/10/2017	28806749	Leo PJ	14/08/2017	PLoS Genet	www.ncbi.nlm.nih.gov/pubmed/28806749	Defining the genetic susceptibility to cervical neoplasia-A genome-wide association study.	Cervical cancer
162	03/10/2017	28806749	Leo PJ	14/08/2017	PLoS Genet	www.ncbi.nlm.nih.gov/pubmed/28806749	Defining the genetic susceptibility to cervical neoplasia-A genome-wide association study.	Cervical cancer
163	03/10/2017	28806749	Leo PJ	14/08/2017	PLoS Genet	www.ncbi.nlm.nih.gov/pubmed/28806749	Defining the genetic susceptibility to cervical neoplasia-A genome-wide association study.	Cervical cancer
164	03/10/2017	28806749	Leo PJ	14/08/2017	PLoS Genet	www.ncbi.nlm.nih.gov/pubmed/28806749	Defining the genetic susceptibility to cervical neoplasia-A genome-wide association study.	Cervical cancer
165	03/10/2017	28806749	Leo PJ	14/08/2017	PLoS Genet	www.ncbi.nlm.nih.gov/pubmed/28806749	Defining the genetic susceptibility to cervical neoplasia-A genome-wide association study.	Cervical cancer
166	03/10/2017	28806749	Leo PJ	14/08/2017	PLoS Genet	www.ncbi.nlm.nih.gov/pubmed/28806749	Defining the genetic susceptibility to cervical neoplasia-A genome-wide association study.	Cervical cancer
167	03/10/2017	28806749	Leo PJ	14/08/2017	PLoS Genet	www.ncbi.nlm.nih.gov/pubmed/28806749	Defining the genetic susceptibility to cervical neoplasia-A genome-wide association study.	Cervical cancer
168	03/10/2017	28806749	Leo PJ	14/08/2017	PLoS Genet	www.ncbi.nlm.nih.gov/pubmed/28806749	Defining the genetic susceptibility to cervical neoplasia-A genome-wide association study.	Cervical cancer
169	03/10/2017	28806749	Leo PJ	14/08/2017	PLoS Genet	www.ncbi.nlm.nih.gov/pubmed/28806749	Defining the genetic susceptibility to cervical neoplasia-A genome-wide association study.	Cervical cancer
170	03/10/2017	28806749	Leo PJ	14/08/2017	PLoS Genet	www.ncbi.nlm.nih.gov/pubmed/28806749	Defining the genetic susceptibility to cervical neoplasia-A genome-wide association study.	Cervical cancer
171	03/10/2017	28806749	Leo PJ	14/08/2017	PLoS Genet	www.ncbi.nlm.nih.gov/pubmed/28806749	Defining the genetic susceptibility to cervical neoplasia-A genome-wide association study.	Cervical cancer
172	03/10/2017	28806749	Leo PJ	14/08/2017	PLoS Genet	www.ncbi.nlm.nih.gov/pubmed/28806749	Defining the genetic susceptibility to cervical neoplasia-A genome-wide association study.	Cervical cancer
173	26/05/2017	28120872	Li X	25/01/2017	Sci Rep	www.ncbi.nlm.nih.gov/pubmed/28120872	Genome-wide association study identifies four SNPs associated with response to platinum	Response to platinum-based neoadjuvan
174	26/05/2017	28120872	Li X	25/01/2017	Sci Rep	www.ncbi.nlm.nih.gov/pubmed/28120872	Genome-wide association study identifies four SNPs associated with response to platinum	Response to platinum-based neoadjuvan
175	26/05/2017	28120872	Li X	25/01/2017	Sci Rep	www.ncbi.nlm.nih.gov/pubmed/28120872	Genome-wide association study identifies four SNPs associated with response to platinum	Response to platinum-based neoadjuvan
176	12/12/2013	23817570	Shi Y	30/06/2013	Nat Genet	www.ncbi.nlm.nih.gov/pubmed/23817570	A genome-wide association study identifies two new cervical cancer susceptibility loci at	Cervical cancer
177	12/12/2013	23817570	Shi Y	30/06/2013	Nat Genet	www.ncbi.nlm.nih.gov/pubmed/23817570	A genome-wide association study identifies two new cervical cancer susceptibility loci at	Cervical cancer
178	12/12/2013	23817570	Shi Y	30/06/2013	Nat Genet	www.ncbi.nlm.nih.gov/pubmed/23817570	A genome-wide association study identifies two new cervical cancer susceptibility loci at	Cervical cancer
179	12/12/2013	23817570	Shi Y	30/06/2013	Nat Genet	www.ncbi.nlm.nih.gov/pubmed/23817570	A genome-wide association study identifies two new cervical cancer susceptibility loci at	Cervical cancer

	I	J	K	L	M	N
127	538 Japanese ancestry cases, 39,556 Japanese ancestry controls	NA	6p22.1	6	29016978	Intergenic
128	538 Japanese ancestry cases, 39,556 Japanese ancestry controls	NA	7q33	7	133504366	Intergenic
129	538 Japanese ancestry cases, 39,556 Japanese ancestry controls	NA	7q33	7	133732785	Intergenic
130	538 Japanese ancestry cases, 39,556 Japanese ancestry controls	NA	7q33	7	133922241	Intergenic
131	538 Japanese ancestry cases, 39,556 Japanese ancestry controls	NA	8p23.1	8	9854826	Intergenic
132	538 Japanese ancestry cases, 39,556 Japanese ancestry controls	NA	8q12.1	8	56246637	Intergenic
133	538 Japanese ancestry cases, 39,556 Japanese ancestry controls	NA	8q12.1	8	59197008	Intergenic
134	538 Japanese ancestry cases, 39,556 Japanese ancestry controls	NA				Intergenic
135	538 Japanese ancestry cases, 39,556 Japanese ancestry controls	NA	9q21.2	9	76775287	Intergenic
136	538 Japanese ancestry cases, 39,556 Japanese ancestry controls	NA	10p13	10	17199011	Intergenic
137	538 Japanese ancestry cases, 39,556 Japanese ancestry controls	NA	11p15.5	11	2158112	Intergenic
138	538 Japanese ancestry cases, 39,556 Japanese ancestry controls	NA	11q12.1	11	58366737	Intergenic
139	538 Japanese ancestry cases, 39,556 Japanese ancestry controls	NA	12p11.22	12	29599423	Intergenic
140	538 Japanese ancestry cases, 39,556 Japanese ancestry controls	NA	12q24.23	12	119604245	Intergenic
141	538 Japanese ancestry cases, 39,556 Japanese ancestry controls	NA	13q12.11	13	22100187	Intergenic
142	538 Japanese ancestry cases, 39,556 Japanese ancestry controls	NA	13q33.3	13	106422664	Intergenic
143	538 Japanese ancestry cases, 39,556 Japanese ancestry controls	NA	14q11.2	14	20259308	Intergenic
144	538 Japanese ancestry cases, 39,556 Japanese ancestry controls	NA	14q11.2	14	20368118	Intergenic
145	538 Japanese ancestry cases, 39,556 Japanese ancestry controls	NA	14q21.1	14	41755191	Intergenic
146	538 Japanese ancestry cases, 39,556 Japanese ancestry controls	NA	14q32.31	14	101059844	Intergenic
147	538 Japanese ancestry cases, 39,556 Japanese ancestry controls	NA	16p13.3	16	6687493	Intergenic
148	538 Japanese ancestry cases, 39,556 Japanese ancestry controls	NA	17q24.3	17	72101710	Intergenic
149	538 Japanese ancestry cases, 39,556 Japanese ancestry controls	NA	19q13.32	19	45666590	Intergenic
150	538 Japanese ancestry cases, 39,556 Japanese ancestry controls	NA	19q13.41	19	51734303	Intergenic
151	538 Japanese ancestry cases, 39,556 Japanese ancestry controls	NA	20p13	20	4817030	Intergenic
152	538 Japanese ancestry cases, 39,556 Japanese ancestry controls	NA	20q13.32	20	58375898	Intergenic
153	2,609 East Asian ancestry cases, 4,712 East Asian ancestry controls	1,461 Han Chinese ancestry cases, 3,295 Han Chinese ancestry cont	2p11.2	2	85219363	NR
154	2,609 East Asian ancestry cases, 4,712 East Asian ancestry controls	1,461 Han Chinese ancestry cases, 3,295 Han Chinese ancestry cont	5q14.3	5	91087644	ADGRV1
155	2,609 East Asian ancestry cases, 4,712 East Asian ancestry controls	1,461 Han Chinese ancestry cases, 3,295 Han Chinese ancestry cont	7p11.2	7	54380269	HPVC1, LINC01445, VS
156	2,609 East Asian ancestry cases, 4,712 East Asian ancestry controls	1,461 Han Chinese ancestry cases, 3,295 Han Chinese ancestry cont	6p21.32	6	33143124	NR
157	2,609 East Asian ancestry cases, 4,712 East Asian ancestry controls	1,461 Han Chinese ancestry cases, 3,295 Han Chinese ancestry cont	17q21.1	17	39895095	
158	1,553 Swedish ancestry cervical intraepithelial neoplasia grade 3 cas	827 Swedish ancestry cervical intraepithelial neoplasia grade 3 cases	6p21.32	6	32616804	intergenic
159	2,866 European ancestry cases, 6,481 European ancestry controls	NA	6p21.33	6	31512891	MICB, MCCD1
160	2,866 European ancestry cases, 6,481 European ancestry controls	NA	4p12	4	47087167	GABRB1
161	2,866 European ancestry cases, 6,481 European ancestry controls	NA	7q34	7	139841136	TBXAS1
162	2,866 European ancestry cases, 6,481 European ancestry controls	NA	8p23.1	8	8523519	SGK223, CLDN23
163	2,866 European ancestry cases, 6,481 European ancestry controls	NA	8q13.3	8	69663895	SULF1, SLCO5A1
164	2,866 European ancestry cases, 6,481 European ancestry controls	NA	13q14.11	13	40927414	SUGT1P3, ELF1
165	2,866 European ancestry cases, 6,481 European ancestry controls	NA	15q26.3	15	99451803	LRRC28, MEF2A
166	2,866 European ancestry cases, 6,481 European ancestry controls	NA	18q22.3	18	73596115	LOC100505817, FBXO15
167	2,866 European ancestry cases, 6,481 European ancestry controls	NA	6p21.33	6	31572364	TNF
168	2,866 European ancestry cases, 6,481 European ancestry controls	NA	6p21.32	6	32633026	HLA-DRB1
169	2,866 European ancestry cases, 6,481 European ancestry controls	NA				HLA-DQB1, HLA-DRB1,
170	2,866 European ancestry cases, 6,481 European ancestry controls	NA				HLA-DQA1, HLA-DRB1
171	2,866 European ancestry cases, 6,481 European ancestry controls	NA				HLA-B, HLA-C
172	2,866 European ancestry cases, 6,481 European ancestry controls	NA				HLA-DRB1, HLA-DQB1,
173	66 Han Chinese ancestry non-responder cases, 160 Han Chinese anc	79 Han Chinese ancestry non-responder cases, 291 Han Chinese anc	4q34.3	4	179391655	intergenic
174	66 Han Chinese ancestry non-responder cases, 160 Han Chinese anc	79 Han Chinese ancestry non-responder cases, 291 Han Chinese anc	14q32.11	14	90698719	CALM1, TTC7B, NRDE2
175	66 Han Chinese ancestry non-responder cases, 160 Han Chinese anc	79 Han Chinese ancestry non-responder cases, 291 Han Chinese anc	16q23.3	16	83706502	MIR3182, HSBP1, MLY
176	Up to 1,364 Han Chinese ancestry cases, up to 3,028 Han Chinese a	4,167 Han Chinese ancestry cases, 7,196 Han Chinese ancestry cont	4q12	4	55885574	EXOC1
177	Up to 1,364 Han Chinese ancestry cases, up to 3,028 Han Chinese a	4,167 Han Chinese ancestry cases, 7,196 Han Chinese ancestry cont	6p21.32	6	33104395	HLA-DPB2
178	Up to 1,364 Han Chinese ancestry cases, up to 3,028 Han Chinese a	4,167 Han Chinese ancestry cases, 7,196 Han Chinese ancestry cont	17q21.1	17	39895095	GSDMB
179	Up to 1,364 Han Chinese ancestry cases, up to 3,028 Han Chinese a	4,167 Han Chinese ancestry cases, 7,196 Han Chinese ancestry cont	6p21.32	6	33236497	intergenic

	O	P	Q
127	ZNF311 - OR2AD1P	ENSG00000197935	ENSG00000233650
128	EXOC4		
129	RPS3AP27, EXOC4		
130	EXOC4		
131	TNKS - LINC00599	ENSG00000173273	ENSG00000253230
132	AC107952.2 - AC107952.1	ENSG00000272343	ENSG00000254216
133	AC087698.1 - RNA5SP267	ENSG00000206853	ENSG00000201763
134			
135	PCA3, PRUNE2		
136	TRDMT1		
137	INS-IGF2, AC132217.2		
138	OR5B1P		
139	TMTC1		
140	TMEM233, AC002070.1		
141	AL136962.1		
142	LINC00460 - RPL35P9	ENSG00000233532	ENSG00000174418
143	TTC5		
144	TEP1		
145	LRFN5		
146	MIR154 - MIR496	ENSG00000207978	ENSG00000207961
147	RBFOX1		
148	LINC02097, SOX9-AS1		
149	RN7SL836P - GIPR	ENSG00000241226	ENSG00000010310
150	HAS1 - FPR1	ENSG00000105509	ENSG00000171051
151	RASSF2		
152	RAB22A - VAPB	ENSG00000124209	ENSG00000124164
153	TCF7L1		
154	ADGRV1, LUCAT1		
155	LINC01445		
156	HLA-DPA3, HLA-DPA3, HLA-DPA3, HLA-DPA3, HLA-DPA3, HLA-DPA3		
157	ZPBP2 - GSDMB	ENSG00000186075	ENSG00000073605
158	HLA-DRB1 - HLA-DQA1	ENSG00000206240	ENSG00000196735
159	MICB - AL663061.2	ENSG00000231372	ENSG00000256851
160	GABRB1		
161	TBXAS1		
162	AC103957.2 - AC114550.2	ENSG00000254153	ENSG00000253343
163	SULF1 - SLCO5A1	ENSG00000137573	ENSG00000137571
164	SUGT1P3 - ELF1	ENSG00000239827	ENSG00000120690
165	AC015660.1 - AC015660.5	ENSG00000259341	ENSG00000286893
166	LINC02582 - AC079070.1	ENSG00000261780	ENSG00000265380
167	LTA, LTA, LTA, LTA, LTA, LTA, LTA		
168	HLA-DQA1, HLA-DQA1, HLA-DQA1, HLA-DQA1, HLA-DQA1		
169	HLA-DQA1		
170			
171			
172	HLA-DQA1		
173	AC020551.1		
174	AL139193.2, TTC7B		
175	CDH13, AC009063.3		
176	EXOC1		
177	COL11A2P1, COL11A2P1, COL11A2P1, COL11A2P1, COL11A2P1, COL11A2P1, COL11A2P1		
178	ZPBP2 - GSDMB	ENSG00000186075	ENSG00000073605
179	ZNF70P1 - HTATSF1P1	ENSG00000230949	ENSG00000224052

	R	S	T	U	V
127		11662	9702	rs3129795-?	rs3129795
128	ENSG00000131558			rs147175877-?	rs147175877
129	ENSG00000226205, ENSG00000131558			rs149996974-?	rs149996974
130	ENSG00000131558			rs141588684-?	rs141588684
131		72480	45045	rs2090064-?	rs2090064
132		23464	6323	rs75951998-?	rs75951998
133		59506	258877	rs58726170-?	rs58726170
134				chr9:22422311-?	chr9:22422311
135	ENSG00000225937, ENSG00000106772			rs17784899-?	rs17784899
136	ENSG00000107614			rs147380957-?	rs147380957
137	ENSG00000129965, ENSG00000284779			rs150806792-?	rs150806792
138	ENSG00000233499			rs184467454-?	rs184467454
139	ENSG00000133687			rs377042484-?	rs377042484
140	ENSG00000224982, ENSG00000248636			rs142761815-?	rs142761815
141	ENSG00000276476			rs146343995-?	rs146343995
142		38349	3109	rs141629209-?	rs141629209
143	ENSG00000136319			rs145233920-?	rs145233920
144	ENSG00000129566			rs149049108-?	rs149049108
145	ENSG00000165379			rs138523818-?	rs138523818
146		6	729	rs41286572-?	rs41286572
147	ENSG00000078328			rs72764914-?	rs72764914
148	ENSG00000226101, ENSG00000234899			rs140991990-?	rs140991990
149		14785	1631	rs56182403-?	rs56182403
150		10312	10869	rs75724373-?	rs75724373
151	ENSG00000101265			rs201524967-?	rs201524967
152		8391	13331	rs6513380-?	rs6513380
153	ENSG00000152284			rs6547598-A	rs6547598
154	ENSG00000164199, ENSG00000248323			rs59661306-G	rs59661306
155	ENSG00000231427			rs7457728-C	rs7457728
156	ENSG00000223696, ENSG00000223447, ENSG00000231895, ENSG00000227936, ENSG00000224929, ENSG00000237398			rs115137622-C	rs115137622
157		17199	9500	rs8067378-G	rs8067378
158		26956	11375	rs73730372-?	rs73730372
159		1767	3088	rs3132461-G	rs3132461
160	ENSG00000163288			rs4396968-T	rs4396968
161	ENSG00000059377			rs2267681-?	rs2267681
162		62182	32219	rs56804039-?	rs56804039
163		2980	3151	rs4738017-G	rs4738017
164		5640	4510	rs9532669-A	rs9532669
165		16643	21651	rs11637339-C	rs11637339
166		246236	115214	rs17087933-?	rs17087933
167	ENSG00000223919, ENSG00000231408, ENSG00000230279, ENSG00000226979, ENSG00000238130, ENSG00000173503, ENSG00000226275			rs2239704-A	rs2239704
168	ENSG00000232062, ENSG00000206305, ENSG00000236418, ENSG00000225890, ENSG00000196735			rs9272143-?	rs9272143
169				HLA-DQB1*602-?	HLA-DQB1*60
170				HLA-DQA1*0301-?	HLA-DQA1*03
171				HLA-B*15-?	HLA-B*15
172				HAL-DRB1*13-?	HAL-DRB1*13
173	ENSG00000250993			rs6812281-T	rs6812281
174	ENSG00000258716, ENSG00000165914			rs1742101-G	rs1742101
175	ENSG00000140945, ENSG00000261103			rs1364121-A	rs1364121
176	ENSG00000090989			rs13117307-T	rs13117307
177	ENSG00000228688, ENSG00000232802, ENSG00000227577, ENSG00000226513, ENSG00000234249, ENSG00000228164, ENSG00000230816			rs4282438-?	rs4282438
178		17199	9500	rs8067378-G	rs8067378
179		20169	1302	rs9277952-?	rs9277952

	W	X	Y	Z	AA	AB	AC	AD	AE	AF	AG	AH	AI	AJ
127	0	3129795	TF_binding_site_variant	1		8.00E-06	5.096910013	1.0526316	[1.03-1.08]	Illumina [7645193] (imputed)	N	cervical carcinoma	GCST009825	
128	0	147175877	intron_variant	0		3.00E-06	5.522878745	2.66	[1.77-4.02]	Illumina [7645193] (imputed)	N	cervical carcinoma	GCST009826	
129	0	149996974	non_coding_transcript_exon_variant	0		1.00E-06	6	3.79	[2.21-6.51]	Illumina [7645193] (imputed)	N	cervical carcinoma	GCST009826	
130	0	141588684	intron_variant	0		5.00E-06	5.301029996	3.6	[2.07-6.23]	Illumina [7645193] (imputed)	N	cervical carcinoma	GCST009826	
131	0	2090064	intergenic_variant	1		5.00E-07	6.301029996	1.4492754	[1.25-1.67]	Illumina [7645193] (imputed)	N	cervical carcinoma	GCST009826	
132	0	75951998	intergenic_variant	1		1.00E-06	6	1.86	[1.45-2.38]	Illumina [7645193] (imputed)	N	cervical carcinoma	GCST009826	
133	0	58726170	intergenic_variant	1		7.00E-06	5.15490196	2.01	[1.48-2.72]	Illumina [7645193] (imputed)	N	cervical carcinoma	GCST009826	
134	0			1		5.00E-06	5.301029996	4.82	[2.46-9.45]	Illumina [7645193] (imputed)	N	cervical carcinoma	GCST009826	
135	0	17784899	intron_variant	0		6.00E-07	6.22184875	1.5	[1.28-1.76]	Illumina [7645193] (imputed)	N	cervical carcinoma	GCST009826	
136	0	147380957	intron_variant	0		4.00E-06	5.397940009	1.86	[1.43-2.42]	Illumina [7645193] (imputed)	N	cervical carcinoma	GCST009826	
137	0	150806792	intron_variant	0		5.00E-08	7.301029996	4.96	[2.79-8.83]	Illumina [7645193] (imputed)	N	cervical carcinoma	GCST009826	
138	0	184467454	non_coding_transcript_exon_variant	0		9.00E-06	5.045757491	2.47	[1.66-3.67]	Illumina [7645193] (imputed)	N	cervical carcinoma	GCST009826	
139	0	377042484	intron_variant	0		4.00E-06	5.397940009	2.65	[1.75-4.01]	Illumina [7645193] (imputed)	N	cervical carcinoma	GCST009826	
140	0	142761815	intron_variant	0		2.00E-06	5.698970004	2.73	[1.81-4.12]	Illumina [7645193] (imputed)	N	cervical carcinoma	GCST009826	
141	0	146343995	intron_variant	0		2.00E-07	6.698970004	4.31	[2.49-7.46]	Illumina [7645193] (imputed)	N	cervical carcinoma	GCST009826	
142	0	141629209	regulatory_region_variant	1		9.00E-08	7.045757491	4.67	[2.65-8.22]	Illumina [7645193] (imputed)	N	cervical carcinoma	GCST009826	
143	0	145233920	intron_variant	0		1.00E-06	6	2.12	[1.57-2.87]	Illumina [7645193] (imputed)	N	cervical carcinoma	GCST009826	
144	0	149049108	3_prime_UTR_variant	0		4.00E-06	5.397940009	2.22	[1.58-3.11]	Illumina [7645193] (imputed)	N	cervical carcinoma	GCST009826	
145	0	138523818	intron_variant	0		3.00E-07	6.522878745	3.57	[2.19-5.83]	Illumina [7645193] (imputed)	N	cervical carcinoma	GCST009826	
146	0	41286572	intergenic_variant	1		4.00E-06	5.397940009	2.19	[1.57-3.06]	Illumina [7645193] (imputed)	N	cervical carcinoma	GCST009826	
147	0	72764914	intron_variant	0		2.00E-07	6.698970004	1.87	[1.47-2.37]	Illumina [7645193] (imputed)	N	cervical carcinoma	GCST009826	
148	0	140991990	intron_variant	0		3.00E-08	7.522878745	4.94	[2.80-8.71]	Illumina [7645193] (imputed)	N	cervical carcinoma	GCST009826	
149	0	56182403	regulatory_region_variant	1		8.00E-07	6.096910013	2.72	[1.83-4.04]	Illumina [7645193] (imputed)	N	cervical carcinoma	GCST009826	
150	0	75724373	regulatory_region_variant	1		5.00E-06	5.301029996	1.45	[1.24-1.70]	Illumina [7645193] (imputed)	N	cervical carcinoma	GCST009826	
151	0	201524967	intron_variant	0		2.00E-06	5.698970004	3.71	[2.17-6.36]	Illumina [7645193] (imputed)	N	cervical carcinoma	GCST009826	
152	0	6513380	intergenic_variant	1		7.00E-06	5.15490196	2.98	[1.85-4.79]	Illumina [7645193] (imputed)	N	cervical carcinoma	GCST009826	
153	0	6547598	intron_variant	0	0.93	1.00E-06	6	1.35	[NR]	Affymetrix, Illumina [4602429] (imp	N	cervical carcinoma	GCST007119	
154	0	59661306	intron_variant	0	0.15	2.00E-11	10.69897	1.2987013	[NR]	Affymetrix, Illumina [4602429] (imp	N	cervical carcinoma	GCST007119	
155	0	7457728	intron_variant	0	0.39	1.00E-08	8	1.19	[NR]	Affymetrix, Illumina [4602429] (imp	N	cervical carcinoma	GCST007119	
156	1	79249189	intron_variant	0	0.62	9.00E-09	8.045757491	1.32	[NR]	Affymetrix, Illumina [4602429] (imp	N	cervical carcinoma	GCST007119	
157	0	8067378	regulatory_region_variant	1	0.28	3.00E-06	5.522878745	1.22	[NR]	Affymetrix, Illumina [4602429] (imp	N	cervical carcinoma	GCST007119	
158	0	73730372	regulatory_region_variant	1		3.00E-19	18.52287875	1.67	[1.49-1.85]	Illumina [5471179]	N	cervical carcinoma	GCST003565	
159	0	3132461	intergenic_variant	1	NR	2.00E-13	12.69897	1.358	[NR]	Illumina [10863230] (imputed)	N	cervical carcinoma	GCST004833	
160	0	4396968	intron_variant	0	NR	1.00E-06	6	1.26	[NR]	Illumina [10863230] (imputed)	N	cervical carcinoma	GCST004833	
161	0	2267681	intron_variant	0	NR	2.00E-06	5.698970004	1.1806375	[NR]	Illumina [10863230] (imputed)	N	cervical carcinoma	GCST004833	
162	0	56804039	intergenic_variant	1	NR	4.00E-06	5.397940009	1.2091899	[NR]	Illumina [10863230] (imputed)	N	cervical carcinoma	GCST004833	
163	0	4738017	regulatory_region_variant	1	NR	7.00E-06	5.15490196	1.174	[NR]	Illumina [10863230] (imputed)	N	cervical carcinoma	GCST004833	
164	0	9532669	intergenic_variant	1	NR	2.00E-07	6.698970004	1.216	[NR]	Illumina [10863230] (imputed)	N	cervical carcinoma	GCST004833	
165	0	11637339	intergenic_variant	1	NR	6.00E-06	5.22184875	1.184	[NR]	Illumina [10863230] (imputed)	N	cervical carcinoma	GCST004833	
166	0	17087933	intergenic_variant	1	NR	2.00E-06	5.698970004	1.2787724	[NR]	Illumina [10863230] (imputed)	N	cervical carcinoma	GCST004833	
167	0	2239704	5_prime_UTR_variant	0	NR	6.00E-09	8.22184875	1.23	[NR]	Illumina [10863230] (imputed)	N	cervical carcinoma	GCST004833	
168	0	9272143	intron_variant	0	NR	5.00E-15	14.30103	1.3513513	[NR]	Illumina [10863230] (imputed)	N	cervical carcinoma	GCST004833	
169	0			1	0.141	4.00E-12	11.39794001	1.44	[NR]	Illumina [10863230] (imputed)	N	cervical carcinoma	GCST004833	
170	0			1	0.224	4.00E-08	7.397940009			Illumina [10863230] (imputed)	N	cervical carcinoma	GCST004833	
171	0			1	0.072	2.00E-09	8.698970004	0.64	[NR]	Illumina [10863230] (imputed)	N	cervical carcinoma	GCST004833	
172	0			1	0.106	1.00E-09	9	0.69	[NR]	Illumina [10863230] (imputed)	N	cervical carcinoma	GCST004833	
173	0	6812281	intron_variant	0	0.18	9.00E-09	8.045757491	2.37	[1.77-3.18]	Affymetrix [657178]	N	cervical carcinoma, respo	GCST004097	
174	0	1742101	non_coding_transcript_exon_variant	0	0.5	7.00E-06	5.15490196	1.92	[1.45-2.56]	Affymetrix [657178]	N	cervical carcinoma, respo	GCST004097	
175	0	1364121	non_coding_transcript_exon_variant	0	0.25	3.00E-06	5.522878745	1.98	[1.49-2.64]	Affymetrix [657178]	N	cervical carcinoma, respo	GCST004097	
176	0	13117307	intron_variant	0	0.1	3.00E-10	9.522878745	1.28	[1.19-1.38]	Affymetrix [563339]	N	cervical carcinoma	GCST002082	
177	0	4282438	intron_variant	0	0.56	5.00E-27	26.30103	1.35	[1.27-1.41]	Affymetrix [563339]	N	cervical carcinoma	GCST002082	
178	0	8067378	regulatory_region_variant	1	0.22	9.00E-10	9.045757491	1.19	[1.13-1.26]	Affymetrix [563339]	N	cervical carcinoma	GCST002082	
179	0	9277952	intergenic_variant	1	0.53	2.00E-09	8.698970004	1.18	[1.12-1.23]	Affymetrix [563339]	N	cervical carcinoma	GCST002082	

Supplement Table 10. Replication lookup of UKB SNPS derived from conditional logistic regression in the FInnGen r5 public data.

Phenotype (UKB equivalent)	CHR	pos	ref	alt	rsids	alternnate_rsid	nearest_gene	pval	beta	OR	sebeta	maf	maf_cases	maf_controls	n_hom_cases	n_het_cases	n_hom_contr	n_het_controls
Malignant neoplasm of cervix uteri (ICC)	2	113230915	G	A	rs10175462	NA	PAX8	0.01193	-0.0899	0.91402258	0.0358	0.4305	0.4077	0.4308	272	799.62	22601.06	59849.24
Malignant neoplasm of cervix uteri (ICC)	2	113235223	TCC	T	rs35724515	rs397778746	PAX8	0.01401	-0.088	0.91576088	0.0358	0.4304	0.4081	0.4307	273.6	797.87	22577.43	59870
Malignant neoplasm of cervix uteri (ICC)	5	1347013	C	T	rs27069	NA	CLPTM1L	2.54E-07	-0.1864	0.82994155	0.0362	0.4156	0.3725	0.4162	234.48	758.67	21007.22	59475.17
Malignant neoplasm of cervix uteri (ICC)	6	32631294	G	A	rs9272050	NA	HLA-DQA1	7.90E-08	-0.1951	0.82275238	0.0363	0.5592	0.5095	0.5598	422.45	834.38	38348.99	59824.16
Malignant neoplasm of cervix uteri (ICC)	12	109208011	A	G	rs117960705	NA	ACACB	0.8257	-0.0325	0.96802245	0.1475	0.01498	0.01472	0.01499	1.91	44.69	32.38	3590.26
Carcinoma in situ of cervix uteri (CIN3)	2	113230915	G	A	rs10175462	NA	PAX8	0.06941	-0.1508	0.86001969	0.083	0.4305	0.3927	0.4306	51.05	131.95	22822.02	60516.91
Carcinoma in situ of cervix uteri (CIN3)	2	113235223	TCC	T	rs35724515	rs397778746	PAX8	0.07467	-0.1483	0.86217242	0.0832	0.4304	0.3933	0.4305	51.23	131.98	22799.8	60535.89
Carcinoma in situ of cervix uteri (CIN3)	5	1347013	C	T	rs27069	NA	CLPTM1L	0.03929	-0.1728	0.84130586	0.0838	0.4156	0.3757	0.4157	42.53	138.84	21199.18	60095
Carcinoma in situ of cervix uteri (CIN3)	6	32631294	G	A	rs9272050	NA	HLA-DQA1	0.0004878	-0.2932	0.74587295	0.0841	0.5592	0.4827	0.5593	73.65	140.39	38697.79	60518.15
Carcinoma in situ of cervix uteri (CIN3)	12	109208011	A	G	rs117960705	NA	ACACB	0.3017	-0.3543	0.70166444	0.343	0.01498	0.0101	0.015	0	6.02	34.29	3628.94
Dysplasia of cervi uteri (Cervical dysplasia)*	2	113230915	G	A	rs10175462	NA	PAX8	0.0001735	-0.0865	0.91713555	0.023	0.4322	0.4112	0.4335	728.96	2034.03	12924.65	33944.03
Dysplasia of cervi uteri (Cervical dysplasia)*	2	113235223	TCC	T	rs35724515	rs397778746	PAX8	0.0002372	-0.0848	0.91869601	0.0231	0.432	0.4115	0.4333	731.31	2031.96	12904.49	33958.67
Dysplasia of cervi uteri (Cervical dysplasia)*	5	1347013	C	T	rs27069	NA	CLPTM1L	0.0004168	-0.0823	0.92099562	0.0233	0.4133	0.3948	0.4145	660.02	2033.02	11760.62	33652.54
Dysplasia of cervi uteri (Cervical dysplasia)*	6	32631294	G	A	rs9272050	NA	HLA-DQA1	5.33E-10	-0.1454	0.86467635	0.0234	0.559	0.5231	0.5612	1164.47	2113.34	21754.87	33902.9
Dysplasia of cervi uteri (Cervical dysplasia)*	12	109208011	A	G	rs117960705	NA	ACACB	0.1373	0.141	1.15142465	0.0949	0.01508	0.01701	0.01496	2.13	140.21	21.32	2021.42

Abbreviations: CHR: chromosome
*Cervical dysplasia phenotype consists mainly of CIN3 lesions however will contain a small number of CIN1 and CIN2 lesions as recorded in Finnish Cancer Registries and Hospital Discharge Registries

Supplement Table 11. eQTL results for Discovery SNPs (UKBB) P<10e-8 in Chr 2 novel hit identified from PanCanQTL database **for CESC (N=300)**

Analysis	Cancer Type	RSID	Genomic Location (hg19)	Allele (A/a)	Gene	Gene Position (hg19)	Beta	t-stat	P-value
**eQTL for rs4849179 in CESC**									
	CESC	rs4849179	chr2:113985170	C/T	LOC654433	chr2:113993107-114024579:+	-0.41	-6	6.06E-09
	CESC	rs4849179	chr2:113985170	C/T	PAX8	chr2:113973574-114036527:-	-0.28	-4.23	3.25E-05
**All the SNPs with eQTL with PAX8 in CESC**									
	CESC	rs10175462	chr2:113988492	G/A	PAX8	chr2:113973574-114036527:-	-0.28	-4.21	3.40E-05
	CESC	rs10206269	chr2:113990393	C/A	PAX8	chr2:113973574-114036527:-	-0.3	-4.29	2.46E-05
	CESC	rs11123170	chr2:113978940	C/G	PAX8	chr2:113973574-114036527:-	-0.3	-4.31	2.26E-05
	CESC	rs11123172	chr2:113984303	C/T	PAX8	chr2:113973574-114036527:-	0.28	4.16	4.31E-05
	CESC	rs2863243	chr2:113989267	A/G	PAX8	chr2:113973574-114036527:-	-0.28	-4.22	3.31E-05
	CESC	rs4849177	chr2:113982584	T/C	PAX8	chr2:113973574-114036527:-	-0.3	-4.49	1.05E-05
	CESC	rs4849179	chr2:113985170	C/T	PAX8	chr2:113973574-114036527:-	-0.28	-4.23	3.25E-05
	CESC	rs6707386	chr2:113981022	G/A	PAX8	chr2:113973574-114036527:-	-0.32	-4.69	4.40E-06
	CESC	rs6755040	chr2:113981137	T/C	PAX8	chr2:113973574-114036527:-	-0.31	-4.67	4.81E-06
	CESC	rs7421852	chr2:113990261	G/A	PAX8	chr2:113973574-114036527:-	-0.3	-4.3	2.33E-05
	CESC	rs7563094	chr2:113989327	T/C	PAX8	chr2:113973574-114036527:-	-0.28	-4.21	3.40E-05
	CESC	rs7578633	chr2:113978650	C/T	PAX8	chr2:113973574-114036527:-	-0.31	-4.43	1.34E-05
**All the SNPs with eQTL with LOC654433 in CESC**									
	CESC	rs10175462	chr2:113988492	G/A	LOC654433	chr2:113993107-114024579:+	-0.4	-5.9	1.09E-08
	CESC	rs10206269	chr2:113990393	C/A	LOC654433	chr2:113993107-114024579:+	-0.44	-6.19	2.22E-09
	CESC	rs10579377	chr2:113953974	GACA/G	LOC654433	chr2:113993107-114024579:+	0.32	4.58	7.04E-06
	CESC	rs11123170	chr2:113978940	C/G	LOC654433	chr2:113993107-114024579:+	-0.45	-6.32	1.02E-09
	CESC	rs11123172	chr2:113984303	C/T	LOC654433	chr2:113993107-114024579:+	0.41	5.9	1.06E-08
	CESC	rs12612729	chr2:113957048	G/A	LOC654433	chr2:113993107-114024579:+	0.32	4.58	7.04E-06
	CESC	rs12619508	chr2:113982040	C/A	LOC654433	chr2:113993107-114024579:+	0.35	5.03	8.76E-07
	CESC	rs12620738	chr2:113963739	C/T	LOC654433	chr2:113993107-114024579:+	0.32	4.57	7.52E-06
	CESC	rs2015769	chr2:113957637	A/G	LOC654433	chr2:113993107-114024579:+	0.32	4.58	7.04E-06
	CESC	rs2019137	chr2:113973632	G/A	LOC654433	chr2:113993107-114024579:+	0.33	4.7	4.04E-06
	CESC	rs2241978	chr2:113954006	A/G	LOC654433	chr2:113993107-114024579:+	0.32	4.58	7.04E-06
	CESC	rs2276561	chr2:113956371	G/C	LOC654433	chr2:113993107-114024579:+	0.32	4.58	7.04E-06
	CESC	rs2289897	chr2:113977454	G/A	LOC654433	chr2:113993107-114024579:+	-0.35	-4.42	1.44E-05
	CESC	rs2305133	chr2:113956821	C/G	LOC654433	chr2:113993107-114024579:+	0.32	4.58	7.04E-06
	CESC	rs2863240	chr2:113970116	C/T	LOC654433	chr2:113993107-114024579:+	0.29	3.98	8.77E-05

Abbreviations: CESC: Cervical squamous cell carcinoma and endocervical adenocarcinoma

Supplement Table 12. Two-sample inverse variance weighted (IVW) MR full results for all exposures with GWAS available for analysis

Exposure	OR	SE	Lower 95%CI	Upper 95%CI	P	FDR
Smoking (cigarettes/day)	2.456	0.509	1.636	3.687	1.48E-05	2.24e-05
Age at first pregnancy	0.800	0.068	0.676	0.945	8.79E-03	8.79E-03
Number of sexual partners	1.948	0.300	1.440	2.634	1.49E-05	2.24E-05

Abbreviations: *FDR: false discovery rate; OR: odds ratio; P: P-value; SE: standard error; 95%CI: 95%cconfidence interval.*

Supplement Table 13. Two-sample inverse variance weighted (IVW) MR sensitivity analysis results for all exposures

A) Weighted-Median

Exposure	OR	SE	CI-lower	CI-upper	P-value
Lifetime smoking index	1.992	0.557	1.152	3.445	1.36E-02
Number of sexual partners	1.945	0.399	1.301	2.906	1.17E-03
Age at first pregnancy	0.813	0.087	0.659	1.004	5.44E-02

B) MR-PRESSO

Exposure	Analysis	OR	SE	P-value
Lifetime smoking index	Raw	2.455734	0.50928208	3.02E-05
	Corrected	2.3140887	0.45967255	4.63E-05
Number of sexual partners	Raw	1.9476112	0.29985566	4.06E-05
	Corrected	NA	NA	NA
Age at first pregnancy	Raw	0.7995042	0.04995509	1.59E-02
	Corrected	NA	NA	NA

Supplement Table 14. Multivariable MR results for all exposures

A) Multivariable MR (age at first pregnancy adjusted for number of sexual partners*)

Exposure	OR	SE	CI-lower	CI-upper	P-value
Number of sexual partners	0.217	0.337	0.010	4.586	0.326
Age at first pregnancy	0.758	0.062	0.645	0.890	0.001
**(univariable results for age at first pregancy)**					
Age at first pregnancy	0.800	0.068	0.676	0.945	8.79E-03

B) Multivariable MR on risky behaviours (smoking adjusted for Lifetime smoking incidence) **

Exposure	OR	SE	CI-lower	CI-upper	P-value
Number of sexual partners	1.879	0.333	1.328	2.661	3.74E-04
Lifetime smoking index	1.737	0.425	1.075	2.806	2.42E-02
(univariable results)					
Number of sexual partners	1.948	0.300	1.440	2.634	1.49E-05
Lifetime smoking index	2.456	0.509	1.636	3.687	1.48E-05

*Genetic variants selected as instrumental variables are selected based on age at first pregnancy (n=10)

**Genetic variants selected as instrumental variables are selected based on smoking and number of sexual partner jointly (n=166)

4 SUPPLEMENTARY FIGURES

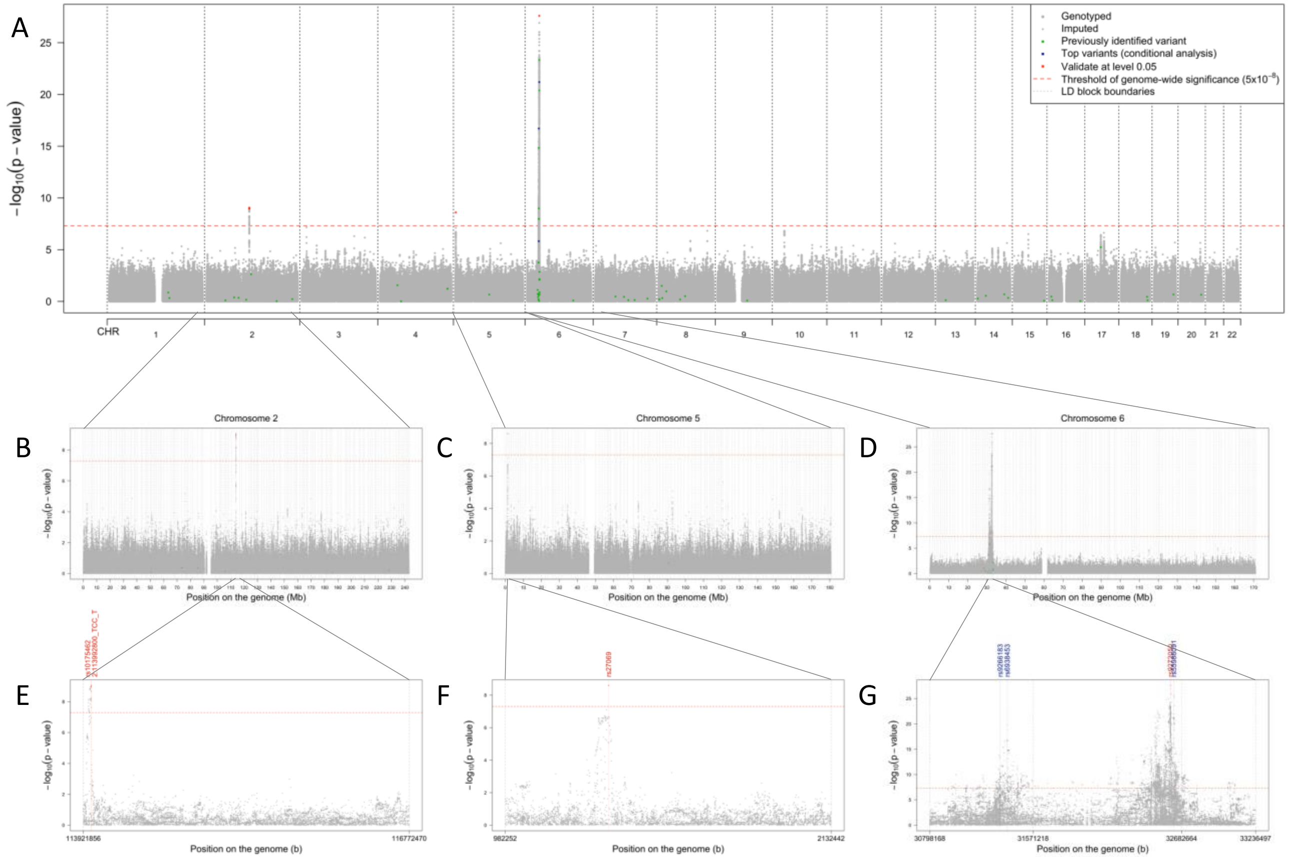

Supplementary Figure 1. Manhattan plots showing the associations between genetic variants and CIN3/ICC in the European unrelated population (N=4,797 cases vs N=146,178 controls). Genetic variants are ordered by their position on the genome (X-axis). The p-values, measuring the strength of association, are derived from additive logistic models and are represented on the -log10 scale (Y-axis). The genome-wide significance level is set to $5x10^{-8}$ (horizontal red line). Results are presented for all the (N=9,600,464) assayed and imputed variants (A), for chromosome 2 (N=798,812 variants, B), 5 (N=616,800 variants, C), and 6 (N=651,104 variants, D). Within each chromosome, the regions containing significant associations are represented in more detail (E, F, G, respectively). Lead SNPS identified in the conditional analysis (blue: rs10175462; rs27069; rs9272050; rs6938453; rs55986091; rs9266183) and derived from FinnGen r5 replication (red: rs10175462 or 113992800_TCC_T; rs27069; rs9272050) are represented.

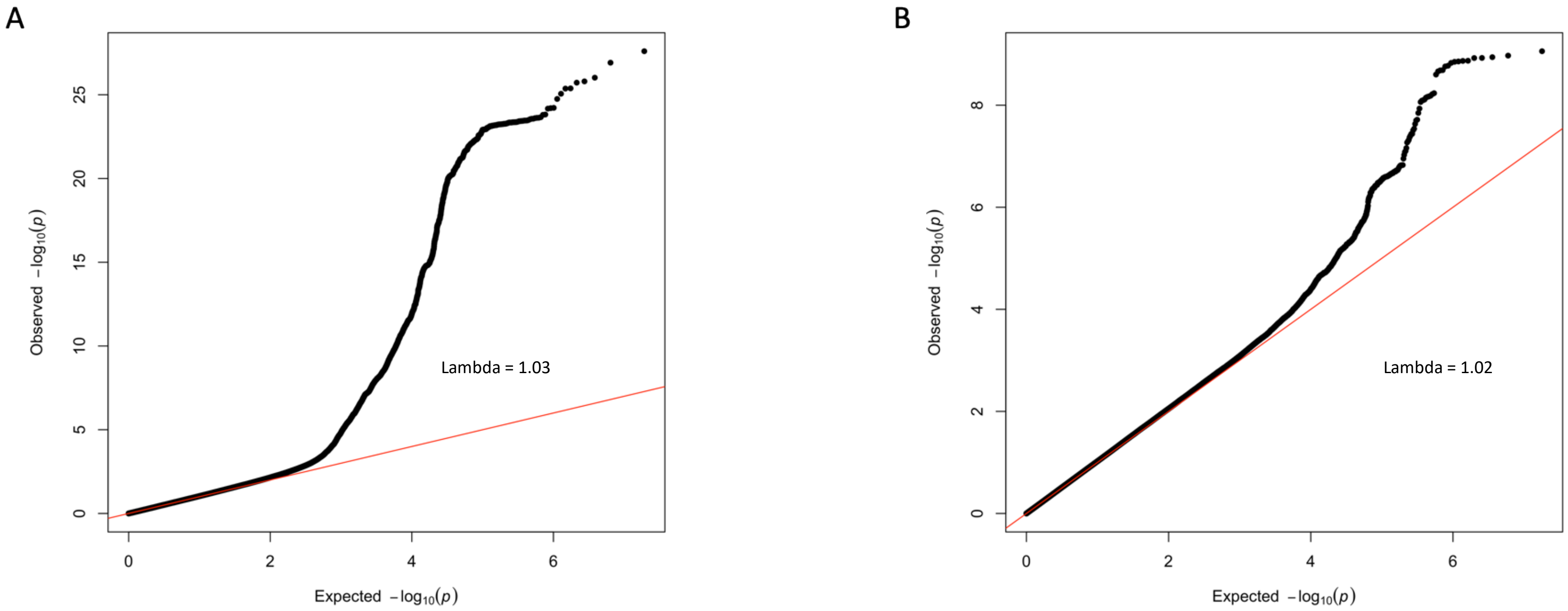

Supplementary Figure 2. Quantile-quantile (QQ) plot of p-values from the genome-wide association study of CIN3/ICC in unrelated participants of European ancestry. The observed p-value of association between each variant and the cervical cancer outcome (Y-axis) is represented as a function of the expected p-value under the assumption of their uniform distribution (X-axis). The red line represents perfect agreement between the observed and expected values. Findings are reported for the whole genome (A, N=9,600,464 variants) and excluding the extended HLA region (B, N=651,104 variants). The Lambda is annotated, which is the ratio of the median empirically observed distribution of the *P*-value to the expected.

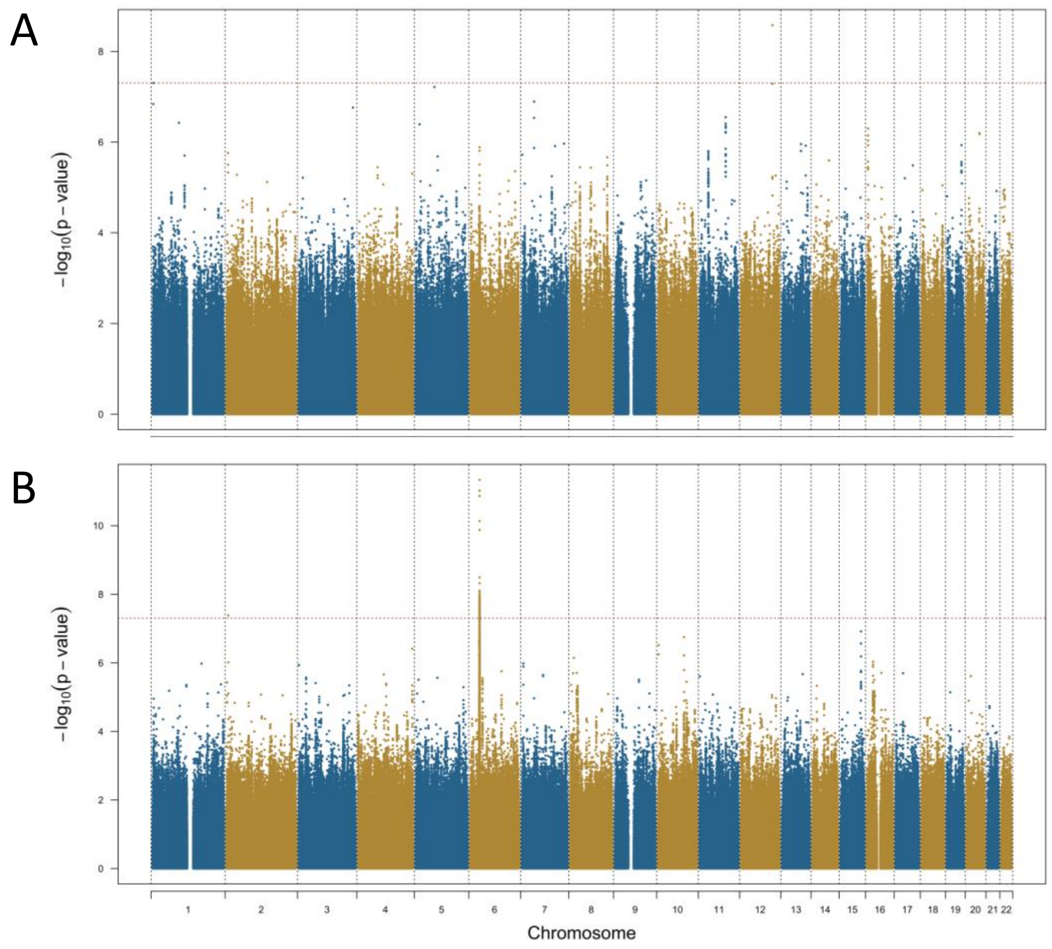

Supplementary Figure 3. Manhattan plots showing the associations between genetic variants and ICC (A, N=764 cases and N=146,178 controls) or CIN3 status (B, subset of N=764 cases and N=146,178 controls) in the European unrelated population. Genetic variants (N=9,600,464) are ordered by their position on the genome (X-axis). The p-values, measuring the strength of association, are derived from additive logistic models and are represented on the -log10 scale (Y-axis). The genome-wide significance level is set to $5x10^{-8}$ (horizontal red line).

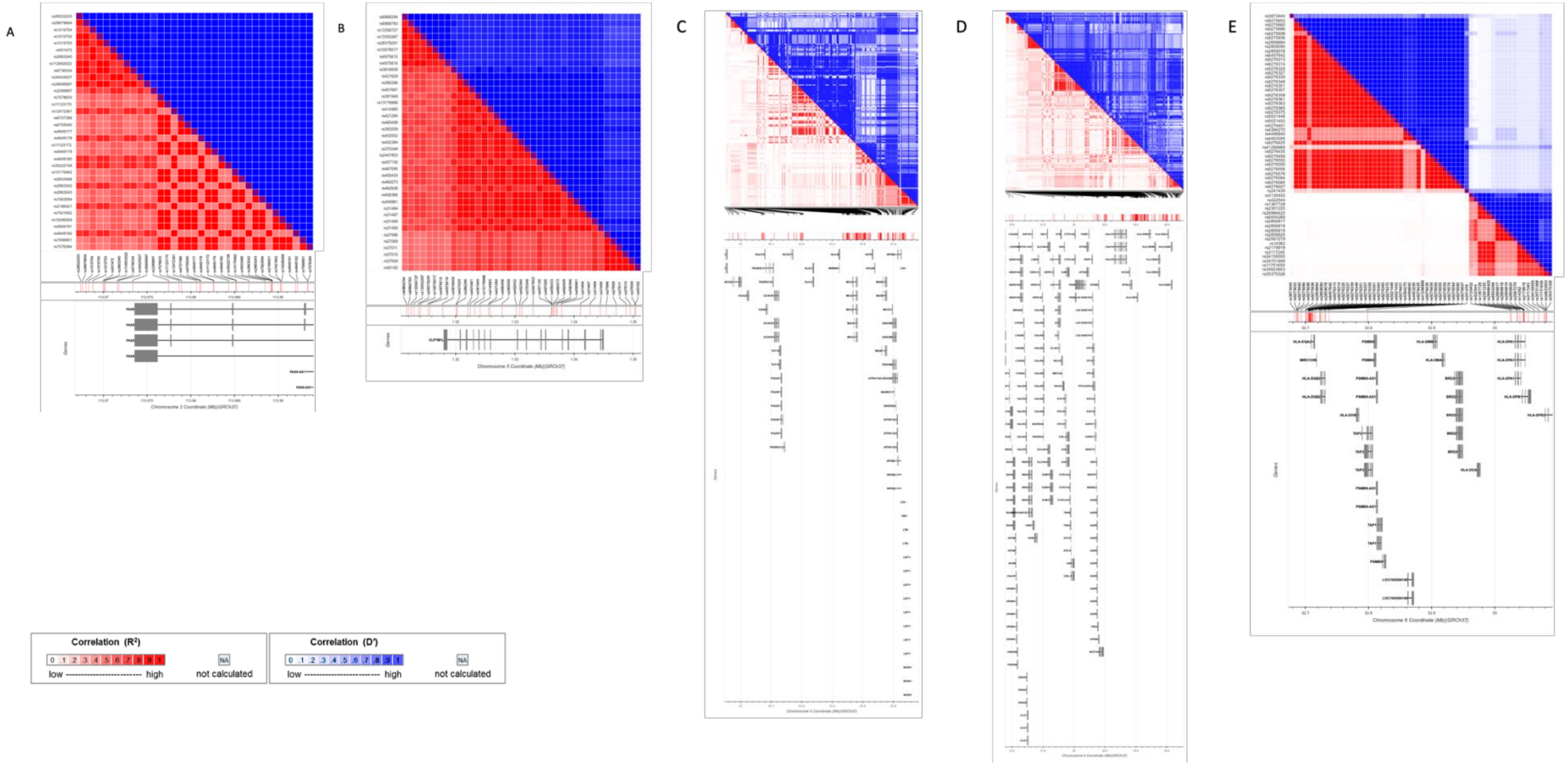

Supplementary Figure 4. Heatmap of pairwise linkage disequilibrium statistics between genome-wide significant variants ($p<5x10^{-8}$) with imputation scores above 0.95 in chromosome 2 (A), chromosome 5 (for $p<10^{-6}$, B), and chromosome 6 separated by LD block (C, D, E). The r-squared (red) and correlations (blue) have been generated using LDlink on reference British population from the 1K genome project. Exons of corresponding transcripts and gene name are also displayed.

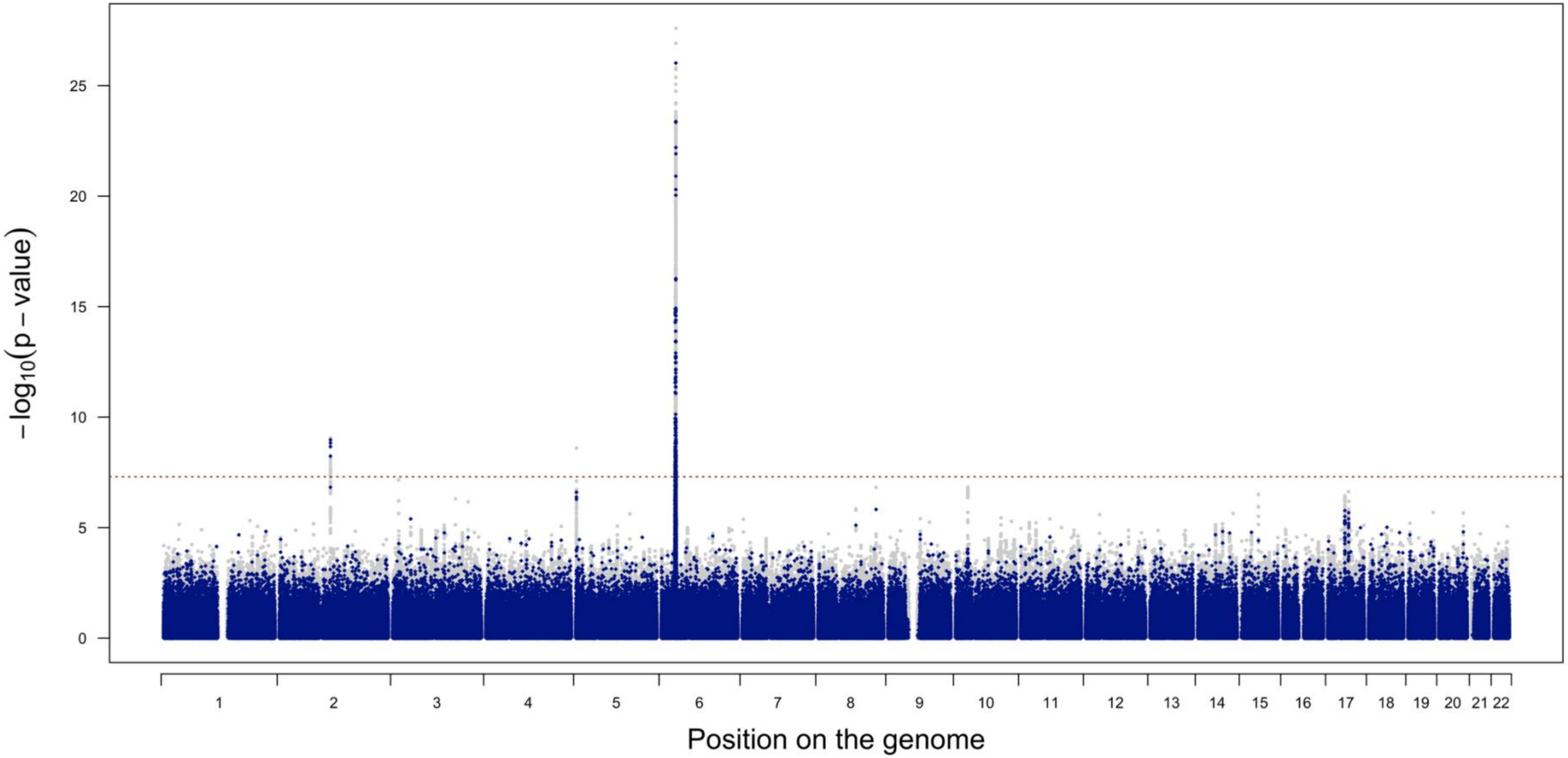

Supplementary Figure 5. Manhattan plots showing the associations between genetic variants and cervical cancer status in the European unrelated population (N=4,797 cases, of which 764 ICC, vs N=146,178 controls). Genetic variants are ordered by their position on the genome (X-axis). The p-values, measuring the strength of association, are derived from additive logistic models and are represented on the -log10 scale (Y-axis). The genome-wide significance level is set to $5x10^{-8}$ (horizontal red line). Results are presented for all the (N=9,600,464) genotyped and imputed variants (grey), and the (N=656,284) genotyped only variants (blue).

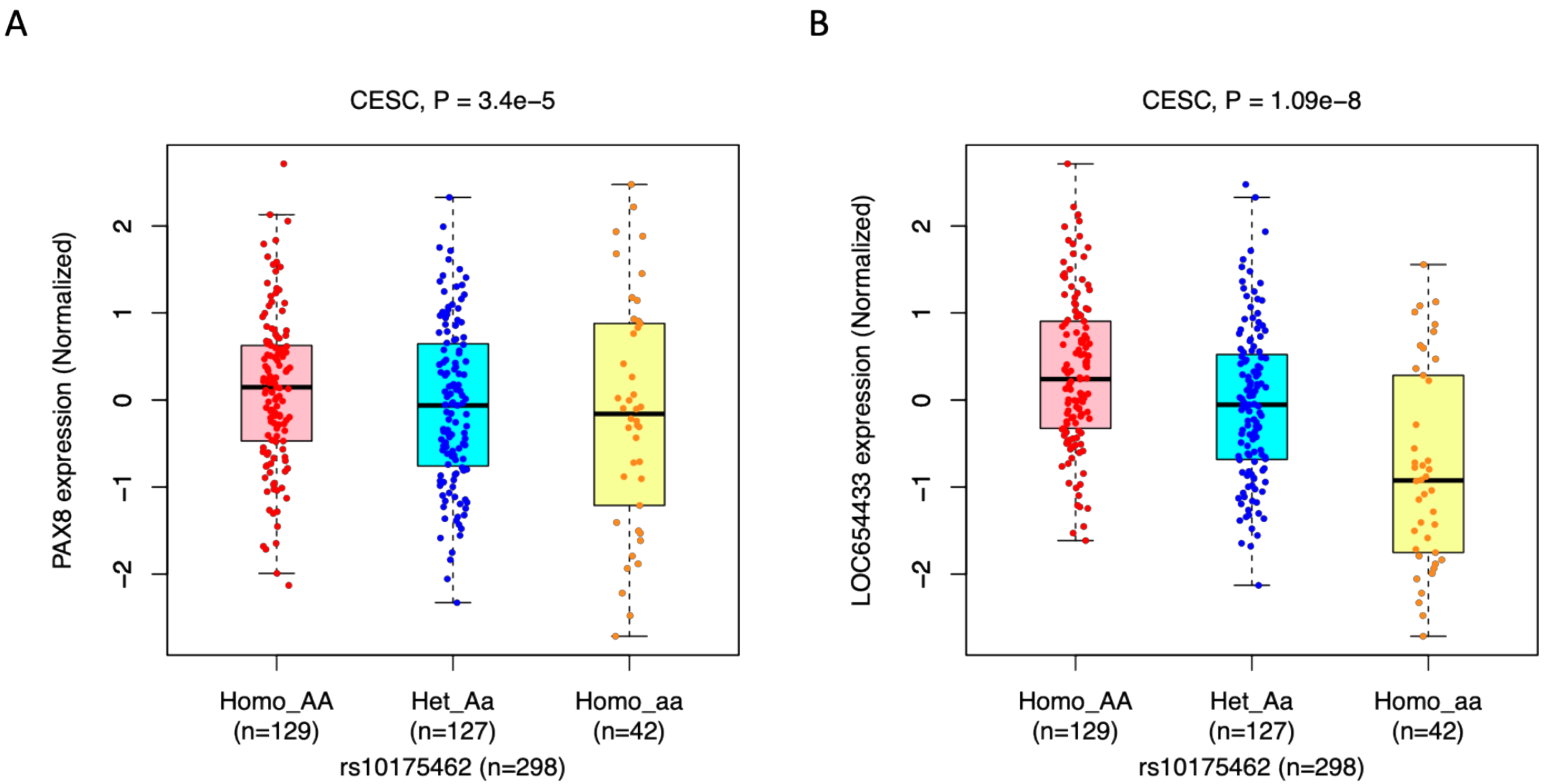

Supplementary Figure 6. Significant SNPs were annotated for their effect on eQTL ratios in ICC from the PanCanQTL database. Box plots show the gene expression levels in PAX8 (A) and LOC654433 (B) genes, for 298 cervical cancer tissue samples for the three genotypes at rs10175462. Overall p-value for differential expression, as computed from an additive linear model (performed using Matrix eQTL), is given above the plot. Homozygosity for the T allele was associated with decreased gene expression at PAX8 and LOC654433. The T allele in the discovery set conferred a decreased risk of cervical cancer trait for PAX8 (OR=0.873; SE = -6.05; P= 1.41e-9) and LOC654433 (OR=0.670; SE =-5.90; P=1.09e-8). Abbreviations: CESC: cervical squamous cell cancer tissue; Homo: homozygous; Het: heterozygous.

5 FINNGEN CONTRIBUTORS (RELEASE 5)

Steering Committee

Aarno Palotie Institute for Molecular Medicine Finland, HiLIFE, University of Helsinki, Finland

Mark Daly Institute for Molecular Medicine Finland, HiLIFE, University of Helsinki, Finland

Pharmaceutical companies

Howard Jacob Abbvie, Chicago, IL, United States

Athena Matakidou Astra Zeneca, Cambridge, United Kingdom

Heiko Runz Biogen, Cambridge, MA, United States

Sally John Biogen, Cambridge, MA, United States

Robert Plenge Celgene, Summit, NJ, United States

Mark McCarthy Genentech, San Francisco, CA, United States

Julie Hunkapiller Genentech, San Francisco, CA, United States

Meg Ehm GlaxoSmithKline, Brentford, United Kingdom

Dawn Waterworth GlaxoSmithKline, Brentford, United Kingdom

Caroline Fox Merck, Kenilworth, NJ, United States

Anders Malarstig Pfizer, New York, NY, United States

Kathy Klinger Sanofi, Paris, France

Kathy Call Sanofi, Paris, France

Tim Behrens Maze Therapeutics, San Francisco, CA, United States

Patrick Loerch Janssen Biotech, Beerse, Belgium

University of Helsinki & Biobanks

Tomi Mäkelä HiLIFE, University of Helsinki, Finland, Finland

Jaakko Kaprio Institute for Molecular Medicine Finland, HiLIFE, Helsinki, Finland, Finland

Petri Virolainen Auria Biobank / University of Turku / Hospital District of Southwest Finland, Turku, Finland

Kari Pulkki Auria Biobank / University of Turku / Hospital District of Southwest Finland, Turku, Finland

Terhi Kilpi THL Biobank / The National Institute of Health and Welfare Helsinki, Finland

Markus Perola THL Biobank / The National Institute of Health and Welfare Helsinki, Finland

Jukka Partanen Finnish Red Cross Blood Service / Finnish Hematology Registry and Clinical Biobank, Helsinki, Finland

Anne Pitkäranta Helsinki Biobank / Helsinki University and Hospital District of Helsinki and Uusimaa, Helsinki

Riitta Kaarteenaho Northern Finland Biobank Borealis / University of Oulu / Northern Ostrobothnia Hospital District, Oulu, Finland

Seppo Vainio Northern Finland Biobank Borealis / University of Oulu / Northern Ostrobothnia Hospital District, Oulu, Finland

Miia Turpeinen Northern Finland Biobank Borealis / University of Oulu / Northern Ostrobothnia Hospital

Raisa Serpi Northern Finland Biobank Borealis / University of Oulu / Northern Ostrobothnia Hospital

Tarja Laitinen Finnish Clinical Biobank Tampere / University of Tampere / Pirkanmaa Hospital District, Tampere, Finland

Johanna Mäkelä Finnish Clinical Biobank Tampere / University of Tampere / Pirkanmaa Hospital District, Tampere, Finland

Veli-Matti Kosma Biobank of Eastern Finland / University of Eastern Finland / Northern Savo Hospital District, Kuopio, Finland

Urho Kujala Central Finland Biobank / University of Jyväskylä / Central Finland Health Care District, Jyväskylä, Finland

Other Experts/ Non-Voting Members

Outi Tuovila Business Finland, Helsinki, Finland

Minna Hendolin Business Finland, Helsinki, Finland

Raimo Pakkanen Business Finland, Helsinki, Finland

Scientific Committee

Pharmaceutical companies

Jeff Waring	Abbvie, Chicago, IL, United States
Bridget Riley-Gillis	Abbvie, Chicago, IL, United States
Athena Matakidou	Astra Zeneca, Cambridge, United Kingdom
Heiko Runz	Biogen, Cambridge, MA, United States
Jimmy Liu	Biogen, Cambridge, MA, United States
Shameek Biswas	Celgene, Summit, NJ, United States
Julie Hunkapiller	Genentech, San Francisco, CA, United States
Dawn Waterworth	GlaxoSmithKline, Brentford, United Kingdom
Meg Ehm	GlaxoSmithKline, Brentford, United Kingdom
Dorothee Diogo	Merck, Kenilworth, NJ, United States
Caroline Fox	Merck, Kenilworth, NJ, United States
Anders Malarstig	Pfizer, New York, NY, United States
Catherine Marshall	Pfizer, New York, NY, United States
Xinli Hu	Pfizer, New York, NY, United States
Kathy Call	Sanofi, Paris, France
Kathy Klinger	Sanofi, Paris, France
Matthias Gossel	Sanofi, Paris, France
Robert Graham	Maze Therapeutics, San Francisco, CA, United States
Tim Behrens	Maze Therapeutics, San Francisco, CA, United States
Beryl Cummings	Maze Therapeutics, San Francisco, CA, United States
Wilco Fleuren	Janssen Biotech, Beerse, Belgium

University of Helsinki & Biobanks

Samuli Ripatti Institute for Molecular Medicine Finland, HiLIFE, Helsinki, Finland

Johanna Schleutker Auria Biobank / Univ. of Turku / Hospital District of Southwest Finland, Turku, Finland

Markus Perola THL Biobank / The National Institute of Health and Welfare Helsinki, Finland

Mikko Arvas Finnish Red Cross Blood Service / Finnish Hematology Registry and Clinical Biobank, Helsinki, Finland

Olli Carpén Helsinki Biobank / Helsinki University and Hospital District of Helsinki and Uusimaa, Helsinki

Reetta Hinttala Northern Finland Biobank Borealis / University of Oulu / Northern Ostrobothnia Hospital District, Oulu, Finland

Johannes Kettunen Northern Finland Biobank Borealis / University of Oulu / Northern Ostrobothnia Hospital District, Oulu, Finland

Johanna Mäkelä Finnish Clinical Biobank Tampere / University of Tampere / Pirkanmaa Hospital District, Tampere, Finland

Arto Mannermaa Biobank of Eastern Finland / University of Eastern Finland / Northern Savo Hospital District, Kuopio, Finland

Jari Laukkanen Central Finland Biobank / University of Jyväskylä / Central Finland Health Care District, Jyväskylä, Finland

Urho Kujala Central Finland Biobank / University of Jyväskylä / Central Finland Health Care District, Jyväskylä, Finland

Other Experts/ Non-Voting Members

Outi Tuovila Business Finland, Helsinki, Finland

Minna Hendolin Business Finland, Helsinki, Finland

Raimo Pakkanen Business Finland, Helsinki, Finland

Clinical Groups

Oncology Group

Heikki Joensuu Hospital District of Helsinki and Uusimaa, Helsinki, Finland

Olli Carpén Hospital District of Helsinki and Uusimaa, Helsinki, Finland

Tuomo Meretoja Hospital District of Helsinki and Uusimaa, Helsinki, Finland

Lauri Aaltonen	Hospital District of Helsinki and Uusimaa, Helsinki, Finland
Johanna Mattson	Hospital District of Helsinki and Uusimaa, Helsinki, Finland
Johanna Schleutker	University of Turku, Turku, Finland
Annika Auranen	Pirkanmaa Hospital District , Tampere, Finland
Peeter Karihtala	Northern Ostrobothnia Hospital District, Oulu, Finland
Saila Kauppila	Northern Ostrobothnia Hospital District, Oulu, Finland
Päivi Auvinen	Northern Savo Hospital District, Kuopio, Finland
Klaus Elenius	Hospital District of Southwest Finland, Turku, Finland
Relja Popovic	Abbvie, Chicago, IL, United States
Jeffrey Waring	Abbvie, Chicago, IL, United States
Bridget Riley-Gillis	Abbvie, Chicago, IL, United States
Anne Lehtonen	Abbvie, Chicago, IL, United States
Athena Matakidou	Astra Zeneca, Cambridge, United Kingdom
Jennifer Schutzman	Genentech, San Francisco, CA, United States
Julie Hunkapiller	Genentech, San Francisco, CA, United States
Natalie Bowers	Genentech, San Francisco, CA, United States
John Michon	Genentech, San Francisco, CA, United States
Vinay Mehta	Merck, Kenilworth, NJ, United States
Andrey Loboda	Merck, Kenilworth, NJ, United States
Aparna Chhibber	Merck, Kenilworth, NJ, United States
Heli Lehtonen	Pfizer, New York, NY, United States
Stefan McDonough	Pfizer, New York, NY, United States
Marika Crohns	Sanofi, Paris, France
Diptee Kulkarni	GlaxoSmithKline, Brentford, United Kingdom¨

FinnGen Analysis working group

Justin Wade Davis	Abbvie, Chicago, IL, United States
Bridget Riley-Gillis	Abbvie, Chicago, IL, United States

Danjuma Quarless	Abbvie, Chicago, IL, United States
Fedik Rahimov	Abbvie, Chicago, IL, United States
Sahar Esmaeeli	Abbvie, Chicago, IL, United States
Slavé Petrovski	Astra Zeneca, Cambridge, United Kingdom
Eleonor Wigmore	Astra Zeneca, Cambridge, United Kingdom
Jimmy Liu	Biogen, Cambridge, MA, United States
Chia-Yen Chen	Biogen, Cambridge, MA, United States
Paola Bronson	Biogen, Cambridge, MA, United States
Ellen Tsai	Biogen, Cambridge, MA, United States
Stephanie Loomis	Biogen, Cambridge, MA, United States
Yunfeng Huang	Biogen, Cambridge, MA, United States
Joseph Maranville	Celgene, Summit, NJ, United States
Shameek Biswas	Celgene, Summit, NJ, United States
Elmutaz Shaikho Elhaj Mohammed	Celgene, Summit, NJ, United States
Samir Wadhawan	Bristol-Meyers-Squibb
Erika Kvikstad	Bristol-Meyers-Squibb
Minal Caliskan	Bristol-Meyers-Squibb
Diana Chang	Genentech, San Francisco, CA, United States
Julie Hunkapiller	Genentech, San Francisco, CA, United States
Tushar Bhangale	Genentech, San Francisco, CA, United States
Natalie Bowers	Genentech, San Francisco, CA, United States
Sarah Pendergrass	Genentech, San Francisco, CA, United States
Dorothee Diogo	Merck, Kenilworth, NJ, United States
Emily Holzinger	Merck, Kenilworth, NJ, United States
Padhraig Gormley	Merck, Kenilworth, NJ, United States
Xing Chen	Pfizer, New York, NY, United States
Åsa Hedman	Pfizer, New York, NY, United States
Karen S King	GlaxoSmithKline, Brentford, United Kingdom
Clarence Wang	Sanofi, Paris, France

Ethan Xu Sanofi, Paris, France

Franck Auge Sanofi, Paris, France

Clement Chatelain Sanofi, Paris, France

Deepak Rajpal Sanofi, Paris, France

Dongyu Liu Sanofi, Paris, France

Katherine Call Sanofi, Paris, France

Tai-he Xia Sanofi, Paris, France

Beryl Cummings Maze Therapeutics, San Francisco, CA, United States

Matt Brauer Maze Therapeutics, San Francisco, CA, United States

Mitja Kurki Institute for Molecular Medicine Finland, HiLIFE, University of Helsinki, Finland / Broad Institute, Cambridge, MA, United States

Samuli Ripatti Institute for Molecular Medicine Finland, HiLIFE, University of Helsinki, Finland

Mark Daly Institute for Molecular Medicine Finland, HiLIFE, University of Helsinki, Finland

Juha Karjalainen Institute for Molecular Medicine Finland, HiLIFE, University of Helsinki, Finland / Broad Institute, Cambridge, MA, United States

Aki Havulinna Institute for Molecular Medicine Finland, HiLIFE, University of Helsinki, Finland

Anu Jalanko Institute for Molecular Medicine Finland, HiLIFE, University of Helsinki, Finland

Priit Palta Institute for Molecular Medicine Finland, HiLIFE, University of Helsinki, Finland

Pietro della Briotta Parolo Institute for Molecular Medicine Finland, HiLIFE, University of Helsinki, Finland

Wei Zhou Broad Institute, Cambridge, MA, United States

Susanna Lemmelä Institute for Molecular Medicine Finland, HiLIFE, University of Helsinki, Finland

Manuel Rivas University of Stanford, Stanford, CA, United States

Jarmo Harju Institute for Molecular Medicine Finland, HiLIFE, University of Helsinki, Finland

Aarno Palotie Institute for Molecular Medicine Finland, HiLIFE, University of Helsinki, Finland

Arto Lehisto Institute for Molecular Medicine Finland, HiLIFE, University of Helsinki, Finland

Andrea Ganna Institute for Molecular Medicine Finland, HiLIFE, University of Helsinki, Finland

Vincent Llorens Institute for Molecular Medicine Finland, HiLIFE, University of Helsinki, Finland

Hannele Laivuori Institute for Molecular Medicine Finland, HiLIFE, University of Helsinki, Finland

Sina Rüeger Institute for Molecular Medicine Finland, HiLIFE, University of Helsinki, Finland

Mari E Niemi Institute for Molecular Medicine Finland, HiLIFE, University of Helsinki, Finland

Taru Tukiainen Institute for Molecular Medicine Finland, HiLIFE, University of Helsinki, Finland

Mary Pat Reeve Institute for Molecular Medicine Finland, HiLIFE, University of Helsinki, Finland

Henrike Heyne Institute for Molecular Medicine Finland, HiLIFE, University of Helsinki, Finland

Nina Mars Institute for Molecular Medicine Finland, HiLIFE, University of Helsinki, Finland

Kimmo Palin University of Helsinki, Helsinki, Finland

Javier Garcia-Tabuenca University of Tampere, Tampere, Finland

Harri Siirtola University of Tampere, Tampere, Finland

Tuomo Kiiskinen Institute for Molecular Medicine Finland, HiLIFE, University of Helsinki, Finland

Jiwoo Lee Institute for Molecular Medicine Finland, HiLIFE, University of Helsinki, Finland / Broad Institute, Cambridge, MA, United States

Kristin Tsuo Institute for Molecular Medicine Finland, HiLIFE, University of Helsinki, Finland / Broad Institute, Cambridge, MA, United States

Amanda Elliott Institute for Molecular Medicine Finland, HiLIFE, University of Helsinki, Finland / Broad Institute, Cambridge, MA, United States

Kati Kristiansson THL Biobank / The National Institute of Health and Welfare Helsinki, Finland

Mikko Arvas Finnish Red Cross Blood Service, Helsinki, Finland

Kati Hyvärinen Finnish Red Cross Blood Service, Helsinki, Finland

Jarmo Ritari Finnish Red Cross Blood Service, Helsinki, Finland

Miika Koskinen Helsinki Biobank / Helsinki University and Hospital District of Helsinki and Uusimaa, Helsinki

Olli Carpén Helsinki Biobank / Helsinki University and Hospital District of Helsinki and Uusimaa, Helsinki

Johannes Kettunen Northern Finland Biobank Borealis / University of Oulu / Northern Ostrobothnia Hospital District, Oulu, Finland

Katri Pylkäs Northern Finland Biobank Borealis / University of Oulu / Northern Ostrobothnia Hospital District, Oulu, Finland

Marita Kalaoja Northern Finland Biobank Borealis / University of Oulu / Northern Ostrobothnia Hospital District, Oulu, Finland

Minna Karjalainen Northern Finland Biobank Borealis / University of Oulu / Northern Ostrobothnia Hospital District, Oulu, Finland

Tuomo Mantere Northern Finland Biobank Borealis / University of Oulu / Northern Ostrobothnia Hospital District, Oulu, Finland

Eeva Kangasniemi Finnish Clinical Biobank Tampere / University of Tampere / Pirkanmaa Hospital District, Tampere, Finland

Sami Heikkinen Biobank of Eastern Finland / University of Eastern Finland / Northern Savo Hospital District, Kuopio, Finland

Arto Mannermaa Biobank of Eastern Finland / University of Eastern Finland / Northern Savo Hospital District, Kuopio, Finland

Eija Laakkonen Central Finland Biobank / University of Jyväskylä / Central Finland Health Care District, Jyväskylä, Finland

Csilla Sipeky University of Turku, Turku, Finland

Samuel Heron University of Turku, Turku, Finland

Antti Karlsson Auria Biobank / University of Turku / Hospital District of Southwest Finland, Turku, Finland

Dhanaprakash Jambulingam University of Turku, Turku, Finland

Venkat Subramaniam Rathinakannan University of Turku, Turku, Finland

Biobank directors

Lila Kallio Auria Biobank / University of Turku / Hospital District of Southwest Finland, Turku, Finland

Sirpa Soini THL Biobank / The National Institute of Health and Welfare Helsinki, Finland

Jukka Partanen Finnish Red Cross Blood Service / Finnish Hematology Registry and Clinical Biobank, Helsinki, Finland

Eero Punkka Helsinki Biobank / Helsinki University and Hospital District of Helsinki and Uusimaa, Helsinki

Raisa Serpi Northern Finland Biobank Borealis / University of Oulu / Northern Ostrobothnia Hospital District, Oulu, Finland

Johanna Mäkelä Finnish Clinical Biobank Tampere / University of Tampere / Pirkanmaa Hospital District, Tampere, Finland

Veli-Matti Kosma Biobank of Eastern Finland / University of Eastern Finland / Northern Savo Hospital District, Kuopio, Finland

Teijo Kuopio Central Finland Biobank / University of Jyväskylä / Central Finland Health Care District, Jyväskylä, Finland

FinnGen Teams

Administration

Anu Jalanko Institute for Molecular Medicine Finland, HiLIFE, University of Helsinki, Finland

Risto Kajanne Institute for Molecular Medicine Finland, HiLIFE, University of Helsinki, Finland

Mervi Aavikko Institute for Molecular Medicine Finland, HiLIFE, University of Helsinki, Finland

Manuel González Jiménez Institute for Molecular Medicine Finland, HiLIFE, University of Helsinki, Finland

Analysis

Mitja Kurki Institute for Molecular Medicine Finland, HiLIFE, University of Helsinki, Finland / Broad Institute, Cambridge, MA, United States

Juha Karjalainen Institute for Molecular Medicine Finland, HiLIFE, University of Helsinki, Finland / Broad Institute, Cambridge, MA, United States

Pietro della Briotta Parola Institute for Molecular Medicine Finland, HiLIFE, University of Helsinki, Finland

Sina Rüeger Institute for Molecular Medicine Finland, HiLIFE, University of Helsinki, Finland

Arto Lehistö Institute for Molecular Medicine Finland, HiLIFE, University of Helsinki, Finland

Wei Zhou Broad Institute, Cambridge, MA, United States

Masahiro Kanai Broad Institute, Cambridge, MA, United States

Clinical Endpoint Development

Hannele Laivuori Institute for Molecular Medicine Finland, HiLIFE, University of Helsinki, Finland

Aki Havulinna Institute for Molecular Medicine Finland, HiLIFE, University of Helsinki, Finland

Susanna Lemmelä Institute for Molecular Medicine Finland, HiLIFE, University of Helsinki, Finland

Tuomo Kiiskinen Institute for Molecular Medicine Finland, HiLIFE, University of Helsinki, Finland

Communication

Mari Kaunisto Institute for Molecular Medicine Finland, HiLIFE, University of Helsinki, Finland

Data Management and IT Infrastructure

Jarmo Harju Institute for Molecular Medicine Finland, HiLIFE, University of Helsinki, Finland

Elina Kilpeläinen Institute for Molecular Medicine Finland, HiLIFE, University of Helsinki, Finland

Timo P. Sipilä Institute for Molecular Medicine Finland, HiLIFE, University of Helsinki, Finland

Georg Brein Institute for Molecular Medicine Finland, HiLIFE, University of Helsinki, Finland Oluwaseun A. Dada Institute for Molecular Medicine Finland, HiLIFE, University of Helsinki, Finland

Ghazal Awaisa Institute for Molecular Medicine Finland, HiLIFE, University of Helsinki, Finland

Anastasia Shcherban Institute for Molecular Medicine Finland, HiLIFE, University of Helsinki, Finland

Genotyping

Kati Donner Institute for Molecular Medicine Finland, HiLIFE, University of Helsinki, Finland

Timo P. Sipilä Institute for Molecular Medicine Finland, HiLIFE, University of Helsinki, Finland

Sample Collection Coordination

Anu Loukola Helsinki Biobank / Helsinki University and Hospital District of Helsinki and Uusimaa, Helsinki

Sample Logistics

Päivi Laiho THL Biobank / The National Institute of Health and Welfare Helsinki, Finland

Tuuli Sistonen THL Biobank / The National Institute of Health and Welfare Helsinki, Finland

Essi Kaiharju THL Biobank / The National Institute of Health and Welfare Helsinki, Finland

Markku Laukkanen THL Biobank / The National Institute of Health and Welfare Helsinki, Finland

Elina Järvensivu THL Biobank / The National Institute of Health and Welfare Helsinki, Finland

Sini Lähteenmäki THL Biobank / The National Institute of Health and Welfare Helsinki, Finland

Lotta Männikkö THL Biobank / The National Institute of Health and Welfare Helsinki, Finland

Regis Wong THL Biobank / The National Institute of Health and Welfare Helsinki, Finland

Registry Data Operations

Hannele Mattsson THL Biobank / The National Institute of Health and Welfare Helsinki, Finland

Kati Kristiansson THL Biobank / The National Institute of Health and Welfare Helsinki, Finland

Susanna Lemmelä Institute for Molecular Medicine Finland, HiLIFE, University of Helsinki, Finland

Tero Hiekkalinna THL Biobank / The National Institute of Health and Welfare Helsinki, Finland

Teemu Paajanen THL Biobank / The National Institute of Health and Welfare Helsinki, Finland

Sequencing Informatics

Priit Palta Institute for Molecular Medicine Finland, HiLIFE, University of Helsinki, Finland

Kalle Pärn Institute for Molecular Medicine Finland, HiLIFE, University of Helsinki, Finland

Trajectory Team

Tarja Laitinen Pirkanmaa Hospital District, Tampere, Finland

Harri Siirtola University of Tampere, Tampere, Finland

Javier Gracia-Tabuenca University of Tampere, Tampere, Finland

6 References

1. Biobank TU. UK Biobank: Protocol for a Large-Scale Prospective Epidemiological Resource. 2007. http://www.ukbiobank.ac.uk/wp-content/uploads/2011/11/UK-Biobank-Protocol.pdf.

2. UK Biobank. UK Biobank Axiom® Array: Datasheet. 2018. http://www.ukbiobank.ac.uk/wp-content/uploads/2014/04/UK-Biobank-Axiom-Array-Datasheet-2014-1.pdf (accessed 26/06/2020.

3. Bycroft C, Freeman C, Petkova D, et al. The UK Biobank resource with deep phenotyping and genomic data. *Nature* 2018; **562**(7726): 203-9.

4. Biobank TU. Genotyping and Quality Control of UK Biobank, a Large-Scale, Extensively Phenotyped Prospective Resource. 2015. http://biobank.ctsu.ox.ac.uk/crystal/docs/genotyping_qc.pdf.

5. Tsukiyama SI, M; Ariyoshi, H; Shirawaka, I. A new algorithm for generating all the maximal independent sets. . *SIAM J Computing* 1977; **6**: 505-17.

6. Bowden J, Davey Smith G, Haycock PC, Burgess S. Consistent Estimation in Mendelian Randomization with Some Invalid Instruments Using a Weighted Median Estimator. *Genet Epidemiol* 2016; **40**(4): 304-14.

7. Verbanck M, Chen CY, Neale B, Do R. Detection of widespread horizontal pleiotropy in causal relationships inferred from Mendelian randomization between complex traits and diseases. *Nat Genet* 2018; **50**(5): 693-8.

8. Burgess S, Thompson SG. Multivariable Mendelian randomization: the use of pleiotropic genetic variants to estimate causal effects. *Am J Epidemiol* 2015; **181**(4): 251-60.

9. Barban N, Jansen R, de Vlaming R, et al. Genome-wide analysis identifies 12 loci influencing human reproductive behavior. *Nat Genet* 2016; **48**(12): 1462-72.

10. Wootton RE, Richmond RC, Stuijfzand BG, et al. Evidence for causal effects of lifetime smoking on risk for depression and schizophrenia: a Mendelian randomisation study. *Psychol Med* 2019: 1-9.

11. Karlsson Linnér R, Biroli P, Kong E, et al. Genome-wide association analyses of risk tolerance and risky behaviors in over 1 million individuals identify hundreds of loci and shared genetic influences. *Nat Genet* 2019; **51**(2): 245-57.

12. Purcell S, Neale B, Todd-Brown K, et al. PLINK: a toolset for whole-genome association and population-based linkage analysis. *American Journal of Human Genetics* 2007; **81**.

13. Buniello A, MacArthur JAL, Cerezo M, et al. The NHGRI-EBI GWAS Catalog of published genome-wide association studies, targeted arrays and summary statistics 2019. *Nucleic Acids Res* 2019; **47**(D1): D1005-D12.

14. Kamat MA, Blackshaw JA, Young R, et al. PhenoScanner V2: an expanded tool for searching human genotype-phenotype associations. *Bioinformatics* 2019; **35**(22): 4851-3.

15. Haeussler M, Zweig AS, Tyner C, et al. The UCSC Genome Browser database: 2019 update. *Nucleic Acids Res* 2019; **47**(D1): D853-D8.

16. Sherry ST, Ward MH, Kholodov M, et al. dbSNP: the NCBI database of genetic variation. *Nucleic Acids Res* 2001; **29**(1): 308-11.

17. Davis CA, Hitz BC, Sloan CA, et al. The Encyclopedia of DNA elements (ENCODE): data portal update. *Nucleic Acids Res* 2018; **46**(D1): D794-D801.

18. Gong J, Mei S, Liu C, et al. PancanQTL: systematic identification of cis-eQTLs and trans-eQTLs in 33 cancer types. *Nucleic Acids Res* 2018; **46**(D1): D971-D6.

19. The Genotype-Tissue Expression (GTEx) project. *Nat Genet* 2013; **45**(6): 580-5.

20. Forbes S, Clements J, Dawson E, et al. COSMIC 2005. *Br J Cancer* 2006; **94**(2): 318-22.

21. Ward LD, Kellis M. HaploReg v4: systematic mining of putative causal variants, cell types, regulators and target genes for human complex traits and disease. *Nucleic Acids Res* 2016; **44**(D1): D877-81.

22. Astle WJ, Elding H, Jiang T, et al. The Allelic Landscape of Human Blood Cell Trait Variation and Links to Common Complex Disease. *Cell* 2016; **167**(5): 1415-29 e19.

23. Kelemen LE, Lawrenson K, Tyrer J, et al. Genome-wide significant risk associations for mucinous ovarian carcinoma. *Nat Genet* 2015; **47**(8): 888-97.

24. Wang Y, Wang Y, Li J, et al. PAX8: a sensitive and specific marker to identify cancer cells of ovarian origin for patients prior to neoadjuvant chemotherapy. *Journal of Hematology & Oncology* 2013; **6**(1): 60.

25. Wang W, Yue Z, Tian Z, et al. Expression of Yin Yang 1 in cervical cancer and its correlation with E-cadherin expression and HPV16 E6. *PLoS One* 2018; **13**(2): e0193340.

26. Tian C, Hromatka BS, Kiefer AK, et al. Genome-wide association and HLA region fine-mapping studies identify susceptibility loci for multiple common infections. *Nat Commun* 2017; **8**(1): 599.

27. Lesseur C, Diergaarde B, Olshan AF, et al. Genome-wide association analyses identify new susceptibility loci for oral cavity and pharyngeal cancer. *Nat Genet* 2016; **48**(12): 1544-50.

28. Bei JX, Su WH, Ng CC, et al. A GWAS Meta-analysis and Replication Study Identifies a Novel Locus within CLPTM1L/TERT Associated with Nasopharyngeal Carcinoma in Individuals of Chinese Ancestry. *Cancer Epidemiol Biomarkers Prev* 2016; **25**(1): 188-92.

29. Rafnar T, Sulem P, Stacey SN, et al. Sequence variants at the TERT-CLPTM1L locus associate with many cancer types. *Nat Genet* 2009; **41**(2): 221-7.

30. Chitnis N, Clark PM, Kamoun M, Stolle C, Brad Johnson F, Monos DS. An Expanded Role for HLA Genes: HLA-B Encodes a microRNA that Regulates IgA and Other Immune Response Transcripts. *Front Immunol* 2017; **8**: 583.

31. Bierl C, Karem K, Poon AC, et al. Correlates of cervical mucosal antibodies to human papillomavirus 16: results from a case control study. *Gynecol Oncol* 2005; **99**(3 Suppl 1): S262-8.
